# SCTS annual meeting 2023 abstracts

**DOI:** 10.1186/s13019-023-02473-1

**Published:** 2024-06-03

**Adult Cardiac Aortic Valve**

## A1 Patient open descending and thoracoabdominal aortic replacement: operative steps for patients with prior endovascular treatment

### Robert Pruna-Guillen; Ana Lopez-Marco; Benjamin Adams; Aung Oo

#### Barts Heart Centre/St Bartholomew's Hospital, London, UK

**Correspondence**: Robert Pruna-Guillen

*Journal of Cardiothoracic Surgery 19(1):* A1

**Objectives:** Open surgery remains the gold standard for the treatment of the thoracoabdominal aorta (TAA). The rising number of endovascularly treated patients comes with an increase in the number of patients who require secondary open interventions due to the complex nature of the aortic disease or to treat endovascular complications.

**Methods:** We describe our current approach to secondary open extent II TAA repair in patients with prior endovascular repair. We also describe our experience with this cohort of patients in our institution between 2017 and 2022 (n = 22).

**Results:** We routinely use left heart bypass with mild passive hypothermia (34 °C), cerebrospinal fluid drainage, sequential aortic cross-clamping, motor evoked potentials monitoring, oxygen saturations and selective visceral perfusion with blood and renal protection with Custodiol. We advocate for reimplantation of intercostal arteries.

Between September 2017 and August 2022 a total of 22 patients underwent secondary open TAA repair after previous endovascular therapy in our institution.

Mean age was 56.5 years (20–78), and 77% were male.

Previous endovascular treatment consisted of TEVAR (16, 73%), EVAR/FEVAR (5, 23%) and FET (1, 4%). Indications included aneurysmal expansion (12, 54%), endoleak (4, 18%), stent misplacement (3, 14%) and infection (3, 14%).

Extent of the open TAA repair included: DTA (6, 27%), Crawford I (1, 4%), II (11, 50%), III (3, 14%) and IV (1, 4%). 14 patients (64%) were elective, while 8 cases (36%) were urgent/emergencies.

Median time to open TAA surgery from endovascular treatment was 31 months (0.5–264 months). In-hospital mortality was 32% (7 patients). Postoperative complications include haemofiltration (11, 50%), tracheostomy (3, 14%) and paraplegia (3, 14%).

**Conclusions:** Favourable early outcomes and a durable repair can be achieved at experienced high-volume centers, with pre-operative selection and multidisciplinary team based intraoperative and postoperative management of these patients.

## A2 Retrograde type A aortic dissection: a different evil?

### Robert Pruna-Guillen; Ana Lopez-Marco; Benjamin Adams; Aung Oo

#### Barts Heart Centre/St Bartholomew's Hospital, London, UK

**Correspondence**: Robert Pruna-Guillen

*Journal of Cardiothoracic Surgery 19(1):* A2

**Objectives:** Retrograde type A aortic dissection (RTAAD) can be spontaneous or secondary to instrumentation of the descending and thoracoabdominal aorta. It has anatomical differences compared to antegrade TAAD that impact of the management and prognosis. Treatment is not standardised.

**Methods:** We report our current approach to spontaneous RTAAD, and describe our experience with this cohort of patients in our institution between 2018 and 2022 (n = 15).

**Results:** A total of 15 patients experienced a spontaneous RTAAD in our centre between January 2018 and July 2022. Mean age was 60.1 years (30–82) and 93% were male (14/15). In 80% of the cases valve, coronary arteries and supra-aortic trunks were spared by the dissection. Antegrade distal extension to iliacs was common and lower limb malperfusion was present in four cases (27%). The ascending aorta was dilated (> 40 mm) at presentation in 60% of the cases. Emergency surgery with arch/FET replacement was offered to 11 patients (73%); 3 patients (20%) received a limited proximal aortic repair (ascending and hemiarch replacement); and one patient was treated conservatively due to short life expectancy. In all cases, aorta was explored with deep hypothermic cardiac arrest and antegrade cerebral perfusion was used. Overall mortality was 47% (7 patients). Respectively, 100% mortality for cases treated with limited proximal repair and 22% for those who received arch/FET replacement. Other postoperative complications included stroke n = 3 (20%), spinal cord injury n = 2 (13%), tracheostomy n = 7 (47%), hemofiltration for renal failure n = 5 (33%). All eight survivors (53%) remain alive with median follow-up 4.8 months (3.7–11.2 months).

**Conclusions:** Based on our experience, we advocate for aggressive treatment of RTAAD excluding the primary entry tear to prevent immediate and mid-term complications.

## A3 Early and long-term outcomes in treatment of NANB aortic dissection in a single centre

### Michelle Lee; Ottavia Borghese; Ana Lopez-Marco; Benjamin Adams; Aung Oo; Tara Mastracci

#### Barts Heart Centre / St Bartholomew's Hospital, London, UK

**Correspondence**: Michelle Lee

*Journal of Cardiothoracic Surgery 19(1):* A3

**Objectives:** Evaluate early and long-term outcomes in treatment of NANB AAD in a single centre.

**Methods:** Retrospective observational study between 1/01/2016 and 30/06/2022. Early and long-term morbidity and mortality of a strategy of best medical treatment with early symptoms-based intervention was analyzed.

**Results:** Out of 514 AAS (n311, 60.5% type A AAD; n164, 31.9% type B AAD; n19, 3.7% IMH/PAU and n3, 0.6% iatrogenic dissection), n17 (3.3%) NANB were detected (n13, 76.5% men, mean age 64y, range 41–87). Best medical treatment was immediately successful in 90% of cases. Emergent/urgent operation was needed in 47.1% of patients and consisted in FET.

At a mean follow-up of 14 months, the overall survival rate was 94.1%; 17.6% of patients needed an aortic reintervention. CTA showed FL thrombosis in 85.7% of operated vs 64.7% medically treated patients. Patients underwent intervention had significantly higher preoperative diameter of zone 3–4 (mean 33.01 vs. 43.23 mm, p.005) and 5–6 (mean 29.51 vs. 35.04 mm, p.046).

**Conclusions:** There is a continuing controversy over the best treatment for NANB AAD. The benefit to patients of medical/surgical treatment depends critically upon selection criteria. Higher preoperative diameter of DTA seems to be related to a higher need for immediate intervention.

## A4 Assessment of distal aorta using ambuscope to aid decision making during major aortic surgery

### Martin Yates; Karan Rai; Tobin Mangal; Damian Balmforth; Ana Lopez-Marco; Aung Oo

#### Barts Heart Centre/St Bartholomew's Hospital, London, UK

**Correspondence**: Martin Yates

*Journal of Cardiothoracic Surgery 19(1):* A4

**Objectives:** During aortic surgery, the intended extent of aortic replacement is often confirmed intraoperatively by inspection of the aortic arch. The ambuscope is a single use bronchoscope which we have used to accurately and more extensively inspect the arch and proximal descending aorta during circulatory arrest. We describe our initial experience of ambuscope to assist decision making during major aortic surgery.

**Methods:** All patients undergoing aortic surgery at a single institution from Oct 2020 until September 2022 we analysed. Those in whom ambuscope was used were included. Operation notes and imaging was reviewed for demographics, diagnosis, findings and outcomes. Primary outcomes were the use of ambuscope and its impact on intraoperative decision making.

**Results:** From October 2020 until September 2022 the ambuscope was used in 39 major aortic cases. Mean age was 61 years (21–82) and 28 (72%) were male. Thirteen (33%) were elective, 5 (13%) urgent and 21 (54%) emergency. Thirteen (33%) had aneurysm, 18 (46%) Type A dissection and 8 (21%) retrograde Type B dissection. All cases underwent circulatory arrest with ambuscope inspection of the arch and proximal descending aorta. Utilisation of ambuscope included measuring length of frozen elephant trunk required 21 (54%), expansion of ascyrus medical dissection stent (AMDS) 6 (15%), confirming no further tears in arch or descending aorta 9 (23%), unexpected arch tear requiring frozen elephant trunk (FET) 3(8%). Recording of images may be useful for documentation and subsequent review.

**Conclusions:** We describe novel use of ambuscope and its benefits in decision making during major aortic surgery.

## A5 Aprotinin and type 'A' dissection repair: a single centre experience

### Daniel Sitaranjan; Ibrahim Talal Fazmin; Jason Ali; Dan Aston; Florian Falter; Ravi De Silva

#### Royal Papworth Hospital, Cambridge, UK

**Correspondence**: Daniel Sitaranjan

*Journal of Cardiothoracic Surgery 19(1):* A5

**Objectives:** Patients suffering Type A Aortic dissection (TAAD) can develop severe coagulopathy, particularly in the post-operative period. Antifibrinolytics such as Aprotinin have been shown to reduce blood loss and reduce the transfusion burden in cardiac surgical patients. We aim to identify the benefit of Aprotinin over Tranexamic acid (TXA) by investigating patients undergoing surgery for TAAD.

**Methods:** Patients undergoing TAAD repair were retrospectively identified using a hospital database. 32 patients were identified receiving aprotinin and 105 patients received TXA. Patients undergoing complete arch replacement and those requiring mechanical support for excluded from our study. The volume of blood product transfusion in both theatre and in the intensive care unit after surgery was compared as our primary outcome.

**Results:** The demographics and pre-operative risk factors between the two groups are well matched. There were significantly more patients in the Aprotinin group undergoing deep hypothermic circulatory arrest (p = 0.034). There was a significant difference between the groups in intra-operative platelet transfusion, with TXA patients needing 1.48 (± 1.28) units vs those on aprotinin needing 0.94 (± 0.96) units (p = 0.012). This increased platelet transfusion in the TXA group was not replicated in the ICU or in composite transfusion data. When comparing bleeding rates, there was no significant difference in 12-h blood loss and return to theatre between the groups.

There were no differences in incidence of renal replacement therapy. There were also no differences in incidence of stroke, ICU and hospital stays or 30d and 1 year mortality.

**Conclusions:** Based on the current findings—within the limits of a retrospective observational study—aprotinin does not appear to confer any benefit when used during acute Type A aortic dissection repair.

## A6 Remote monitoring for aortic dissection at an aortic referral centre

### Laerke Ghosh^1^; Ottavia Borghese^1^; Debs Das^2^; Aung Oo^3^; Vikas Kapil^4^; Tara Mastracci^5^

#### ^1^St. Bartholomew's Hospital; ^2^St. Bartholomew's Hospital and Barts Heart Centre and Ortus I-Health, London, UK; ^3^St. Bartholomew's Hospital and William Harvey Research Institute, Centre for Cardiovascular Medicine and Devices, London, UK; ^4^Barts BP Centre of Excellence, William Harvey Research Institute, Centre for Cardiovascular Medicine and Devices, London, UK; ^5^St. Bartholomew's Hospital and University College London Department of Surgical and Interventional Sciences, London, UK

**Correspondence**: Laerke Ghosh

*Journal of Cardiothoracic Surgery 19(1):* A6

**Objectives:** Aortic dissection impacts 4.5/100,000 in the UK, and blood pressure [BP] control is critical. There has been no reliable method for monitoring patients’ BP after discharge to assess adherence to MDT developed antihypertensive guidelines. We describe the implementation and use of a novel digital remote monitoring tool for the post-discharge dissection patient.

**Methods:** From September to November 2022 all patients presenting with aortic dissection were enrolled onto a virtual ward. The pathway was developed with the expertise of vascular surgery, cardiothoracic surgery, a clinical hypertension specialist and nursing. Enrolment involved nursing delivered education, and daily monitoring by the clinical team. Adjustment of medications is based on antihypertensive guidelines and are communicated through both phone appointments and correspondence.

**Results:** To date, 22 patients have enrolled. Twelve patients underwent proximal surgery prior to enrolment, and 10 were monitored for distal dissection with or without endovascular intervention. Four enrolled patients did not engage, and four did not initiate registration. Six patients used the tool to communicate with the clinical team and four required hypertension intervention. Overall, 597 BP measurements were recorded; the average systolic BP was 128 mmHg (SD 20.36 mmHg). On average, patients recorded their BP 40 times (range 3–151 recordings); the longest enrolment was for three months.

**Conclusions:** After three months use of a digital tool for remote BP monitoring, we have found high patient uptake, engagement and an impact on antihypertensive management. Implementation of this tool requires a multidisciplinary effort to be successful, but has potential to change the management of dissection.

## A7 Robotic transcranial doppler ultrasound can ensure adequate cerebral perfusion during aortic arch surgery: a-proof-of-concept feasibility study

### Damian Balmforth^1^; Carlos Corredor^2^; Benjamin Adams^2^; Ana Lopez-Marco^2^; Danielle Blackie^2^; Aung Oo^2^

#### ^1^Royal Sussex County Hospital, Brighton, UK; ^2^St Bartholomew's Hospital, London, UK

**Correspondence**: Damian Balmforth

*Journal of Cardiothoracic Surgery 19(1):* A7

**Objectives:** Open surgery to the aortic arch is associated with a significant stroke risk. Neurological injury can occur from inadequate cerebral blood flow (CBF) during deep hypothermic circulatory arrest (DHCA). By directly measuring flow in the middle cerebral artery (MCA), robotic transcranial doppler (TCD) provides a more accurate and real time assessment of CBF than currently established monitoring of cerebral oxygen saturations. The routine use of robotic-TCD for aortic arch surgery has yet to be described in the scientific literature. We describe our early experience of its use.

**Methods:** Over a 4-week period commencing in February 2022, robotic-TCD was used in all patients undergoing elective aortic arch surgery. Robotic-TCD was used to measure cerebral blood flow (CBF) during deep hypothermic circulatory arrest (DHCA). The rate of CBF just prior to commencing DHCA was set as the minimal therapeutic target (MTT) to be achieved during antegrade cerebral perfusion (ACP). ACP flow rates at the MTT were recorded.

**Results:** Seven patients underwent aortic arch surgery with robotic-TCD over the study period. Once the flow in the MCA had been acquired by the robotic-TCD machine, the signal strength remained reliable throughout all seven cases. MTT flows within the MCA were achieved at significantly lower pump flow rates (mean of 5.62 ml/kg/min) compared to standard ACP flow rates (8–10 mls/kg/min). In addition, TCD allowed early identification of any issues with ACP cannula position and increased the use of a singular cannula for ACP as it was often possible to demonstrate good bilateral flow. No post-operative stokes were seen in any of the 7 patients.

**Conclusions:** Robotic-TCD was able to reliably provide continuous monitoring of cerebral blood flow for the duration of an aortic arch surgery case. Its use resulted in a significant reduction in ACP flow rates when compared to standard protocols.

## A8 Cerebrospinal fluid drainage complications following open distal thoracic and thoracoabdominal aortic surgery: a single centre retrospective study

### Adeyemi Olayiwola; Osazuwa Ighodaro; Ana Lopez-Marco; Aung Oo

#### St Bartholomew's Hospital, London, UK

**Correspondence**: Adeyemi Olayiwola

*Journal of Cardiothoracic Surgery 19(1):* A8

**Objective:** Cerebrospinal fluid (CSF) drainage reduces spinal cord ischemia associated with distal aortic surgery. CSF drainage is an invasive technique that poses complications. This study aims to report the complication rate associated with CSF drainage in patients undergoing open surgery of the distal thoracic (DTA) and thoracoabdominal aorta (TAA).

**Methods:** A retrospective single-centre study of prospectively collected data from the local aortic database from 2017 to 2022. Patients who died in the perioperative period (< 24 h) were excluded. The primary outcome was CSF drainage complications; secondary outcomes included spinal drain insertion success rate and duration of drainage. The population was grouped into DTA and TAA groups. Data were analyzed using SPSS and statistical significance was checked using Fisher’s Exact and Mann–Whitney tests.

**Results:** Median age was 56 years with 31% of the cohort having connective tissue disease (CTD). Over 60% of patients underwent TAA surgery. 124 out of 126 patients (98%) had successful spinal drain insertion with an average attempt of 1.47 ± 0.94 and a median drainage duration of 4 days. Drainage duration was significantly shorter in the DTA group (P = 0.011). The overall complication rate was 28.6%, including the following: Non-functional drain or fractured drain (42.9%), CSF leak (31.4%), spinal headache (11.4%), intraventricular hemorrhage (2.9%), retained catheter tip (2.9%), site infection (5.7%). Three patients (8.6%) had more than one complication. 41% of CTD patients had complications as compared to 23% in those with no CTD (p = 0.05). 22 (17.9%) patients had post-operative spinal ischemia with 17.3% in TAAA and 17.8% in the DTA group.

**Conclusions:** CSF drainage is a relatively safe procedure if done by a trained expert with a standardized protocol. Its importance in reducing the rate of spinal cord ischemia cannot be overemphasized (Fig. 1).

**Fig. 1:**
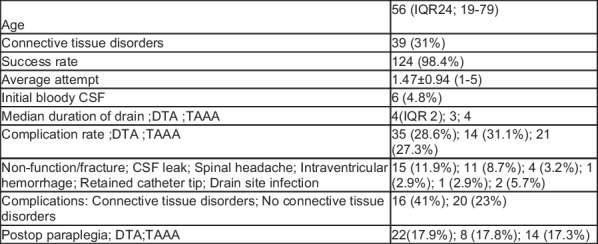
Complication of cerebrospinal fluid drainage in aortic surgery from a single center database collected retrospectively over a 5-year period. The success rate of insertion was 98% with a median drain duration of 4 days. Patients who had surgery for DTA had more complications and non-functional drain was the most complication recorded

## A9 Early results of single centre experience with a novel hybrid prosthesis for type A acute aortic dissection

### Myat Soe Thet; Sarvananthan Sajiram; Benjamin Adams; Kulvinder Lall; John Yap; Camelo Di Salvo; Rakesh Uppal; Ana Lopez-Marco; Aung Y Oo

#### St Bartholomew's Hospital, London, UK

**Correspondence**: Myat Soe Thet

*Journal of Cardiothoracic Surgery 19(1):* A9

**Objectives:** Acute type A aortic dissection is a surgical emergency requiring highly complex surgical repair with high mortality and morbidity rates. We report early results of single-centre experience using the Ascyrus Medical Dissection Stent (AMDS) hybrid prosthesis and its effect on aortic remodelling.

**Materials & Methods:** This retrospective study includes 10 patients with AMDS prostheses between June 2021 to July 2022. Aortic measurements (overall diameter, true lumen, false lumen, false lumen thrombosis) were obtained using computed tomography (CT) two-dimensional multiplanar reconstruction (MPR) from preoperative, postoperative and follow-up scans at 4–6 months. Statistical analysis was performed using IBM SPSS version 28.0.

**Results:** In-hospital mortality was 22.2% (n = 2/9), excluding one salvage procedure. The AMDS-mediated true lumen expansion is significant in the postoperative CT (zone 5; 20.7 ± 3.1 mm, *p* = 0.017) and follow-up CT (zone 5; 21.5 ± 3.3 mm, *p* = 0.046) compared to the preoperative CT (zone 5; 15.5 ± 3.4 mm). The descending aortic diameter remains stable during the follow-up period without further expansion of the false lumen.

**Conclusions:** The AMDS hybrid prosthesis offers effective, reproducible repair in acute type A aortic dissection, and provides early positive remodelling of the aorta. However, long-term follow-up data is required for determination of its role in aortic dissection surgery.

## A10 Using hospital episode statistics data to investigate health inequalities in the treatment and outcomes of patients with thoracic aortic dissection

### Joanne Miksza; Gavin Murphy; Florence Lai; Riccardo Abbasciano

#### University of Leicester, Leicester, UK

**Correspondence**: Joanne Miksza

*Journal of Cardiothoracic Surgery 19(1):* A10

**Objectives:** The use of the Hospital Episode Statistics (HES) dataset allows us to access information on patients typically underrepresented in research and examine whether there are variations in care associated with age, sex, social deprivation or geography in patients with thoracic aortic dissection (TAD).

**Methods:** Patients with a TAD diagnosis between 2013 and 2017 were identified from HES. Survival models were fitted adjusted for patient demographics and comorbidities with all-cause mortality as the outcome. An additional survival model was fitted to investigate the effect of receiving a TAD procedure on all-cause mortality with TAD procedure as a time varying covariate. A logistic regression model was fitted with patient demographics and comorbidities as explanatory variables to calculate the standardised TAD procedure rate by postcode area.

**Results:** The final cohort was 33,793 patients. There was an increased risk of death for women with HR: 1.09 (CI: 1.05–1.14) and an increasing trend in social deprivation quintiles with the most socially deprived having HR: 1.15 (CI: 1.08–1.22) compared to the least socially deprived. Asian patients showed a decrease in risk compared to White patients HR: 0.85 (CI: 0.75–0.97), with Black patients showing no significant difference. Emergency rather than elective admission had the greatest effect on all-cause mortality with HR: 2.52 (CI: 2.40–2.64). There was no significant difference in the effect of TAD procedure on survival after adjustment for patient level characteristics. 12 out of 98 postcode areas had a standardised ratio outside the 99.8% limits.

**Conclusions:** Being a woman and social deprivation are both associated with poorer survival, however an emergency admission provided the greatest increase in risk. There is variability in standardised revascularisation rates by postcode area not explained by patient level characteristics.
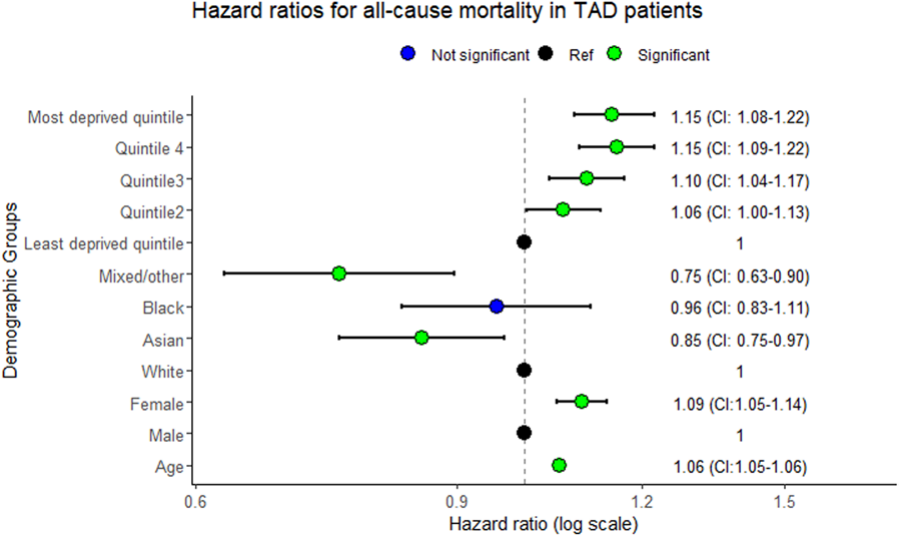


## A11 Peri-operative results and long-term survival in octogenarians with acute type A aortic dissection repair

### Hannah Masraf^1^; Laura Viola^2^; Anna Zingale^2^; Davorin Sef^2^; Szabolcs Miskolczi^2^; Pietro G Malvindi^2^; Theodore Velissaris^2^; Suvitesh Luthra^2^

#### ^1^Kingston Hospital, London, UK; ^2^University Hospital Southampton, Southampton, UK

**Correspondence**: Hannah Masraf

*Journal of Cardiothoracic Surgery 19(1):* A11

**Objectives:** As the population ages, the proportion of octogenarians experiencing acute aortic dissection will inevitably rise. We aimed to assess the peri-operative morbidity, mortality and long-term survival outcomes in octogenarians undergoing acute type A aortic dissection repair.

**Methods:** This was a single centre retrospective study (2007–2021). Demographic data and operative strategies of open versus closed distal anastomosis repair were compared including 26 variables. Univariate and multivariate logistic regression analysis was used for predictors of inpatient mortality. Kaplan–Meier and Cox proportional hazards methods were used to compare long-term survival.

**Results:** A total of 50 octogenarians underwent surgery for spontaneous Type A aortic dissection (22: open anastomosis; 28: closed anastomosis). The mean age was 82.3 ± 1.6 years. In-patient mortality was 18% (open; 14.2% versus closed; 22.7%, p = 0.44) and post-operative cerebrovascular accident (CVA) was 32% (open; 39.3% versus closed; 22.7%, p = 0.21). There was no difference in operative strategies for composite endpoint of hemofiltration, CVA, length of stay (LOS) ≥ 30 days, re-exploration, and inpatient mortality (open 45.5% versus closed 50.0%, p = 0.75). LVEF < 30, NHYA Class 3/4, cross-clamp time, LOS, pre-operative creatinine, cardiopulmonary bypass time, concomitant cardiac procedure and gender were independent predictors of inpatient mortality on univariate regression but not multivariate regression. Survival was similar for both open and closed strategies (open: 7.2 ± 1.3 years versus closed: 10.6 ± 3.1 years, p = 0.35; Fig. 1). Composite end point (HR: 3.65 95%, CI: 1.47–13.9, p = 0.008) and hypertension (HR 0.24, 95% CI: 0.08–0.78, p = 0.02) were predictors for long-term survival.

**Fig. 1:**
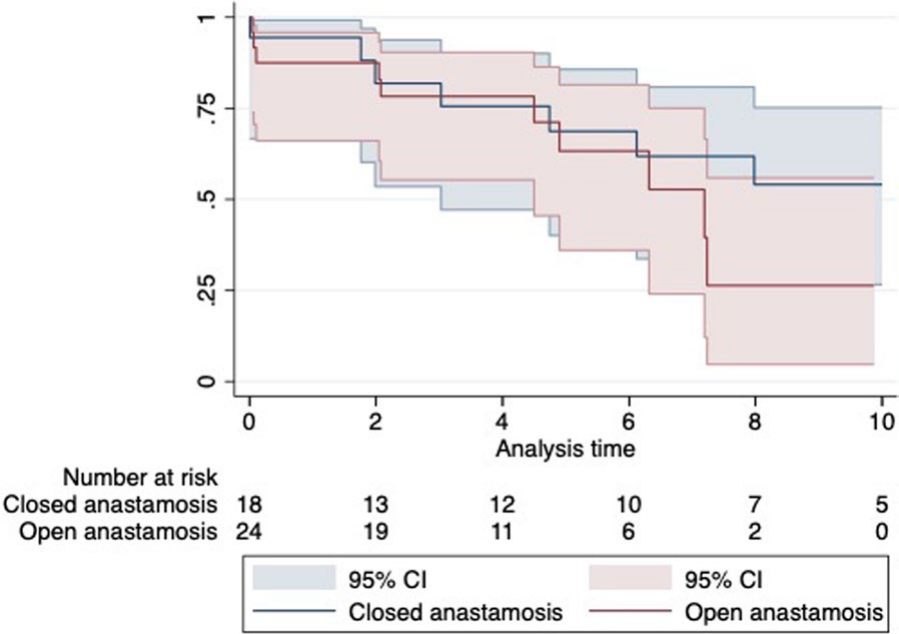
Kaplan–Meier survival curves comparing open vs. closed distal anastomosis technique

**Conclusions:** Aortic dissection repair in octogenarians is associated with high morbidity and mortality. Our study suggests there is no difference between open versus closed anastomosis strategies.

## A12 Percutaneus repair of an early ascending aortic pseudoaneurysm from cardioplegia cannulation site: a rare complication: a case report

### Monica Mittal; Giridhara Goli; Michael Mullen; Kulvinder Lall; John Yap

#### St. Bartholomew's Hospital, London, UK

**Correspondence**: Monica Mittal

*Journal of Cardiothoracic Surgery 19(1):* A12

**Objectives:** To present a percutaneous approach in the management of an early postoperative ascending aortic pseudoaneurysm (AAP) from cardioplegia cannula site, an aetiology that was not previously suggested for the development of AAPs.

**Methods:** We present a case of symptomatic AAP from cardioplegia cannulla that was closed endovasculary with an occluder device in patient post mechanical aortic root replacement, ascending aorta and hemiarch replacement for type A aortic dissection.

**Case presentation:** A 52-year male patient complained of three days history of intermittent chest pain and attended local A&E department 5 weeks after emergency repair of Type A aortic dissection with mechanical aortic root replacement (25 mm St Jude Medical Master Valsalva conduit), ascending aorta and hemiarch replacement. CT scan raised possibility of pseudoaneurysm at the distal anastomosis site. Patient was transferred urgently to our tertiary care centre for further investigation and management. Gated CT aortogram confirmed contrast leak from aortic graft anteriorly with otherwise stable appearances of aortic repair. A multidisciplinary meeting consensus was in favour of percutaneous closure of the pseudoaneurysm.

**Results:** Via radial approach, an AVP4 5 mm device was deployed to occlude the neck of the AAP and an aortogram confirmed the isolation of pseudoaneurysm. The patient was discharged home the same day and a follow up call confirmed that patient is asymptomatic.

**Conclusions:** We have presented a case of an early pseudoaneurysm formation from what we believe was cardioplegia site that was treated successfully with a percutaneously deployed occluder, which is a reasonable approach in highly dedicated centres. It is important that we acknowledge that the insertion and removal of the cardioplegia cannula can be a cause for pseudoaneurysm development. Therefore, we recommend meticulous closure after cardioplegia cannula is removed by reinforcement sutures if needed.

Patient gave informed consent for their information to be published in an Open access journal.

## A13 Mid-term outcomes of an alternative remodelling technique for aortic root replacement without coronary ostial mobilisation or reimplantation

### Leonidas Hadjinikolaou^1^; Metesh Acharya^1^; Carmelo Dominici^2^; Fausto Biancari^3^; Furqan Raheel^1^; Aamer Ahmed^1^; Giovanni Mariscalco^1^

#### ^1^Glenfield Hospital, Leicester, UK; ^2^Campus Bio-Medica University of Rome, Rome, Italy; ^3^Helsinki University Hospital, Helsinki, Finland

**Correspondence**: Metesh Acharya

*Journal of Cardiothoracic Surgery 19(1):* A13

**Objectives:** We compare the early and late outcomes of a modified aortic root remodelling (ARR) technique for aortic root replacement without mobilisation or reimplantation of the coronary ostia, with those of the modified Bentall-de Bono procedure.

**Methods:** A retrospective observational study was performed comprising 181 consecutive patients who underwent aortic root replacement with a modified Bentall-de Bono procedure (104 patients) or ARR (77 patients) between January 2013 and December 2019. Primary endpoints included hospital mortality and late survival. Secondary endpoints included incidence of post-operative complications and freedom from late re-operation.

**Results:** ARR procedures were performed with shorter cross-clamp times and comparable cardiopulmonary bypass times to modified Bentall-de Bono procedures. The incidence of early post-complications was comparable between groups. 30-day mortality was numerically lower with ARR than the modified Bentall-de Bono procedure. Over 7-year follow-up, 4 patients (3.8%) required repeat aortic surgery after a modified Bentall-de Bono procedure, and none after ARR. Long-term mortality after ARR and after modified Bentall-de Bono procedures was 17.1% and 22.7%, respectively. The cumulative incidence of reintervention on the aortic root/valve was 3.2% after a modified Bentall-de Bono procedure and 0% after ARR. When adjusted for other independent risk factors, late mortality was not influenced by the procedure performed, although competing risk adjusted for age showed that the modified Bentall-de Bono procedure was associated with an increased risk of aortic root/aortic valve re-operation.

**Conclusions:** The modified ARR technique is associated with reduced myocardial ischaemia time, lower post-operative mortality and aortic re-intervention rates compared to a modified Bentall-de Bono procedure. It may be considered a safe and feasible procedure for aortic root/ascending aortic replacement offering good long-term outcomes.
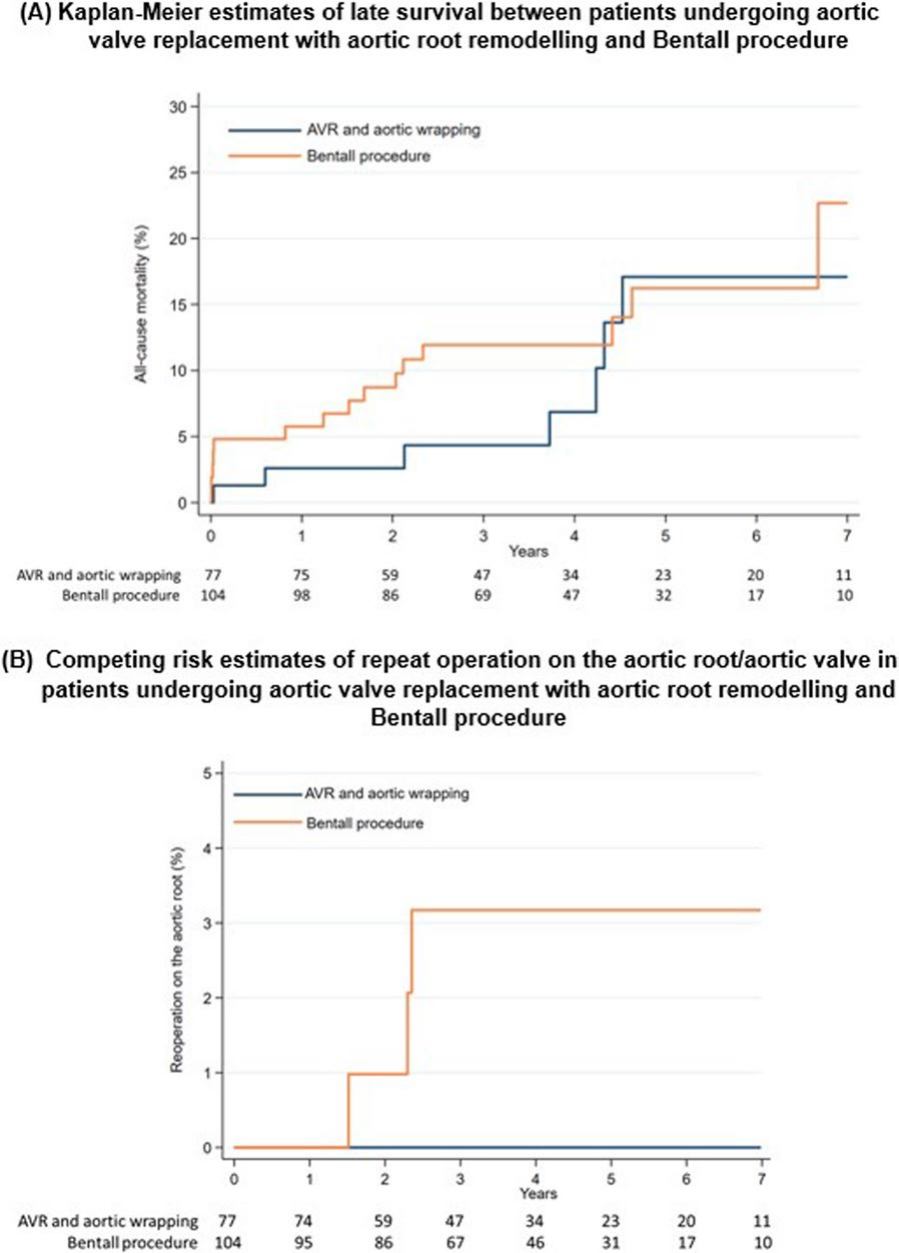


## A14 Normothermic arch replacement without circulatory arrest

### Danilo Verdichizzo; Iakovos Ttofi; Charles Chan; Samail Shahjahan; Amar Keiralla; Ian Harvey; Maurizio Renna; Elaine Hill; George Krasopoulos

#### John Radcliffe Hospital, Oxford University Hospitals, Oxford, UK

**Correspondence**: Danilo Verdichizzo

*Journal of Cardiothoracic Surgery 19(1):* A14

**Objectives**: Aortic arch surgery is traditionally performed on circulatory arrest with or without cerebral perfusion and mild to deep hypothermia. Normothermic cardiopulmonary bypass (NCPB) for arch surgery can reduce operating time, associated coagulopathy, systemic inflammatory response, multi-organ dysfunction and cerebro-spinal injuries.

We present the technique and results of performing aortic arch procedures using NCPB without circulatory arrest.

**Methods**: Four consecutive patients underwent normothermic aortic arch procedures at our Institution by a single aortic surgeon, over 12 months. CPB was established via right (1) or bilateral (3) axillary artery and right atrium. Transcutaneous cerebral oximetry was used. One patient had sickle cell anaemia.

The aortic cross clamp was applied obliquely from zone-I to the proximal descending aorta (pDTA) and separately at the base of the innominate artery. Systemic and left carotid antegrade perfusion was maintained via the left axillary artery and right carotid antegrade cerebral perfusion via right axillary artery. In 1 case a single aortic arch cross-clamp was applied obliquely from zone-0 to pDTA and a full hemi-arch replacement was performed, with the right axillary artery used for both systemic and cerebral perfusion. No circulatory arrest was required.

**Results**: The patients had a mean age of 58.2(36–68), 3 were male and 1 female. Mean CPB time was 142.7 min, cross-clamp 107.5 min. Two were extubated within 6 h and two had prolonged intubation (> 48 h). No patient required re-exploration for bleeding. Mean blood loss was 512.5 ml/12 h. Mean peak blood lactate was 3.3 mmol/L. One patient developed non-oliguric acute kidney injury. One patient (with chronic thrombus in the innominate artery – intraoperative finding) had a stroke from which recovered fully.

**Conclusions:** Normothermic arch replacement can be safely performed without circulatory arrest, in selected cases and by a dedicated, specialist Aortic Team.
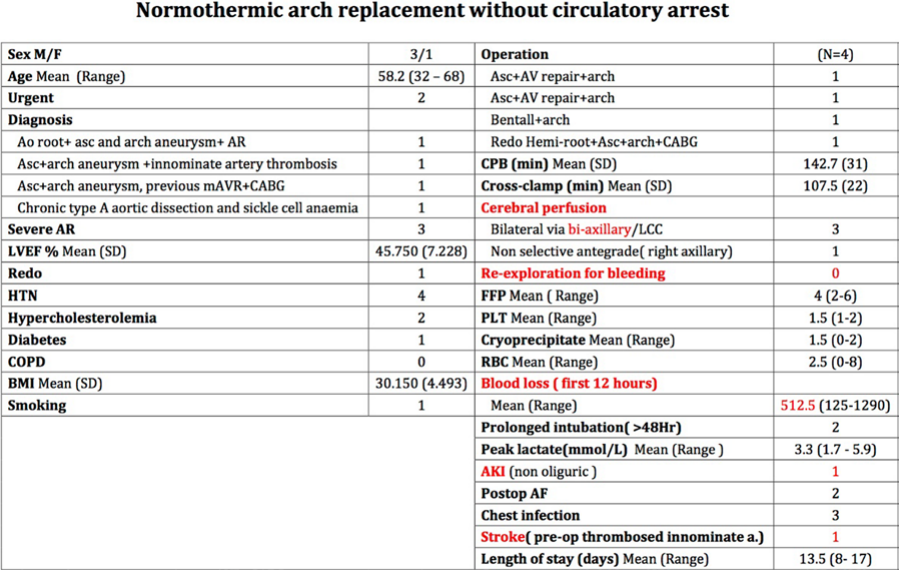


## A15 Type A acute aortic dissection (TAAD) repair: a day time affair or night time?

### Hiral Jhala; Kasra Shaikhrezai; Zahid Mahmood; Nawar Al-Attar; Fraser Sutherland; Rashmi Birla

#### Golden Jubilee National Hospital, Glasgow, UK

**Correspondence**: Hiral Jhala

*Journal of Cardiothoracic Surgery 19(1):* A15

**Objectives:** We assess the impact of time of operation (in-hours vs out-of-hours) on post operative outcomes in patients undergoing surgery for Type A acute aortic dissection (TAAD) in a single, tertiary cardiothoracic referral centre. Comparison is also made with published results from a specialised aortic centre in UK^1^.

**Methods:** Patients who underwent surgery for TAAD between Jan 2012 and Dec 2021 at a single generalised cardiothoracic centre were included. Data was collected prospectively. Retrospective subgroup analysis was performed where patients were grouped according to surgery in-hours (IH) or out-of-hours (OOH), defined as 08:00–17:59 and 18:00–07:59 respectively. Primary outcomes were mortality and cerebrovascular accident (CVA). Statistical significance was determined by Chi-squared and T-tests and defined at p < 0.05.

**Results:** 49 (38.6%) cases were operated IH and 78 (61.4%) cases OOH. The results are tabulated (Table 1). In the IH group, 47 (95.9%) had interposition grafts, 14 (28.6%) had aortic root procedures, and 7 (14.3%) had aortic arch interventions compared to the OOH group 76 (97.9%), 26 (33.3%), 16 (20.5%) (p = 0.47, p = 0.57, p = 0.38) respectively. The in-hospital mortality for IH group was 11 (22.4%) compared to 16 (20.5%) for the OOH group (p = 0.8). Postoperative CVA occurred in 6 (12.2%) patients IH compared to 18 (23%) patients OOH (p = 0.13). At a mean follow up of 5.7 years, 33 (67.3%) patients are alive in the IH group vs 47 (60.3%) in the OOH group (p = 0.42).

**Conclusions:** The neurological and mortality outcomes and the extent of the operation seem unaffected by the time of the day when the operation is undertaken. The outcomes in this tertiary cardiothoracic centre appear similar to the ones reported from a specialist aortic centre in the UK.

^1^Harky A, Mason S, Othman A et al. Outcomes of acute type A aortic dissection repair: Daytime versus nighttime. JTCVS Open. 2021;7:12–20.
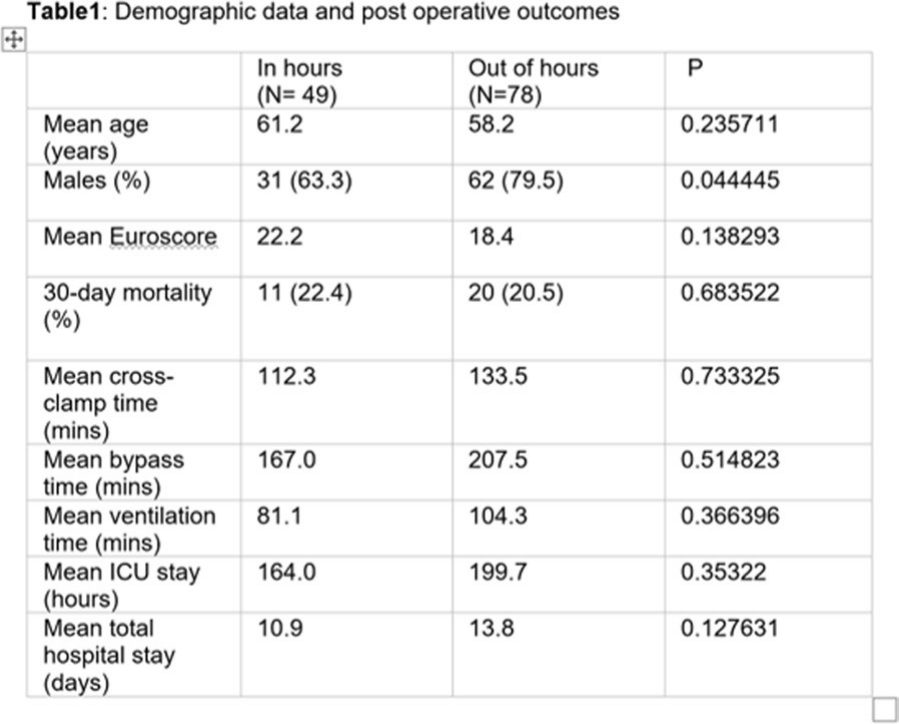


## A16 Improving tertiary pathways for aortic dissections: a novel algorithm

### Ryan Bashir Mohamed; Alicja Zientara; Amina Khalil; Yousuf Salmasi Mohammed; Ahmed Elfadil; Nick Cheshire; George Asimakopoulos; Mario Petrou; Cesare Quarto; Ulrich Rosendahl

#### Royal Brompton Hospital, London, UK

**Correspondence**: Ryan Bashir Mohamed

*Journal of Cardiothoracic Surgery 19(1):* A16

**Objectives:** Acute aortic dissection (AAD) is a time-critical condition with multiple disease factors and clinical processes surrounding its management. Patients are referred to specialist centres with varying symptom severity, malperfusion syndromes and overall critical illness. Additionally, logistical parameters, includingavailability of cross-sectional imaging and transport availability, play an important role for the time-to-treatment.

We aimed to develop an algorithm to improve efficiency and ensure a timely and safe handover from the peripheral hospital to accepting tertiary centre.

**Methods:** Two clinical tools were implemented: (1) handover sheet, and (2) survive-dissection-mini-pathway, that are available online or as hard copy for the on-call cardiothoracic registrar.

The content has been approved by senior staff physicians to be used routinely for every AAD referral and uploaded on the patients’ electronic chart.

**Result:** Preliminary data collection is underway and shows:

1. The handover sheet systematically covers all patient details and history in less than five minutes. Its practical design facilitates easy pick up of red flags and set early primary intervention plan. (Fig. 1).

**Fig. 1:**
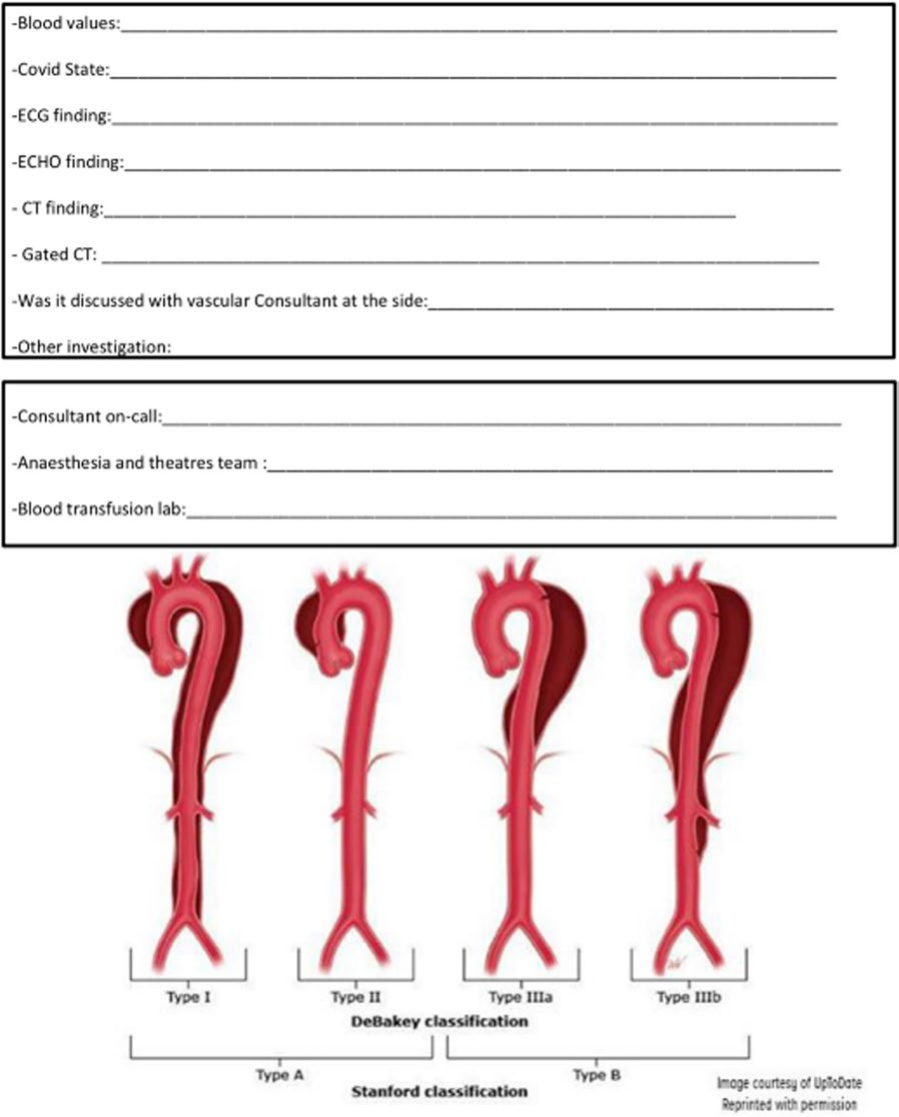
Aortic dissection handover sheet
2. The survive-dissection-mini-pathway is a small card the size of a credit card containing 11 steps of communication with all contact numbers to specifically guide new doctors safely without delay through the process of accepting a type A dissection.

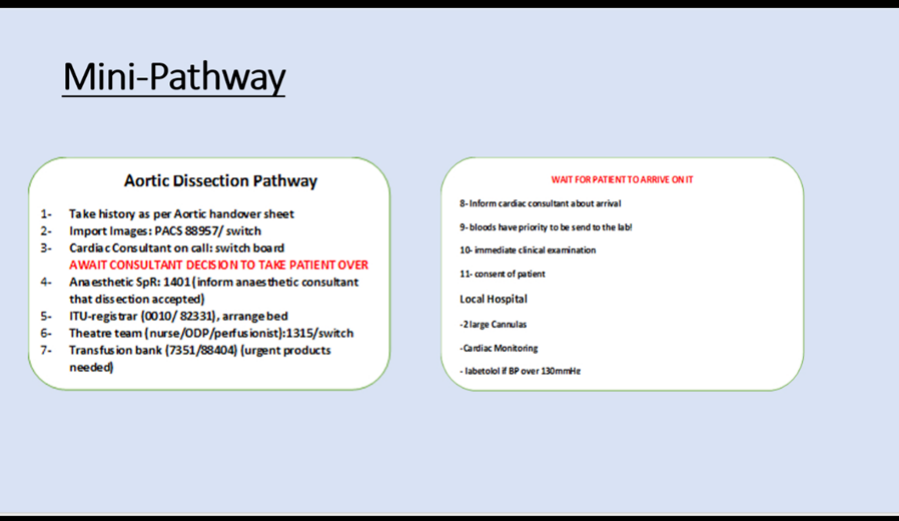


**Conclusions:** This Quality Improvement intervention aims to establish standardised good practice and minimise delay for critically sick patients. Data collection and analysis will identify gaps in the task-specific processes of patient management and help improve patient safety and outcome.

## A17 Minimally invasive david procedure: a single-center experience with a review of literature

### Davorin Sef^1^; Toufan Bahrami^2^; Tomislav Klokocovnik^3^

#### ^1^University of Southampton NHS Foundation Trust, Southampton, UK; ^2^Royal Brompton and Harefield Hospitals, Harefield, UK; ^3^University of Ljubljana, Slovenia, EU, Ljubljana, Slovenia

**Correspondence**: Davorin Sef

*Journal of Cardiothoracic Surgery 19(1):* A17

**Objectives:** There is growing evidence that minimal access cardiac surgery can reduce postoperative morbidity with no difference in mortality. This stimulated enthusiasm in the use of upper ministernotomy for valve-sparing aortic root replacement. We aimed to present our initial experience with David procedure via upper ministernotomy, and a review of all the available literature on this topic.

**Methods:** We reviewed 16 patients (12 male) with isolated aortic root aneurysm selected for minimally invasive David procedure over a 4-year period at our tertiary cardiac centre. A comprehensive literature search from 1992 to 2022 was performed on David procedure via upper ministernotomy.

**Results:** No conversion to full sternotomy or re-exploration due to bleeding was observed. 30-day postoperative mortality and stroke were 0%. Simultaneous aortic valve repair was performed in 8/16 (50%) and coronary artery bypass graft in one patient. Literature search identified 6 retrospective nonrandomized studies (NRSIs) from five different centers, including a total of 250 patients operated via minimal access. Recent NRSIs and comparative studies demonstrated excellent clinical outcomes of minimally invasive David procedure in selected patients with comparable perioperative mortality to the conventional technique.

**Conclusions:** David procedure via upper ministernotomy can be performed with excellent early postoperative results. Meticulous hemostasis is of paramount importance during minimal access David procedure. This approach is particularly beneficial for younger patients as it allows faster recovery with improved cosmesis. Further large randomized studies with long-term follow-up are still required to confirm durability of minimal access approach (Fig. 1).

**Fig. 1:**
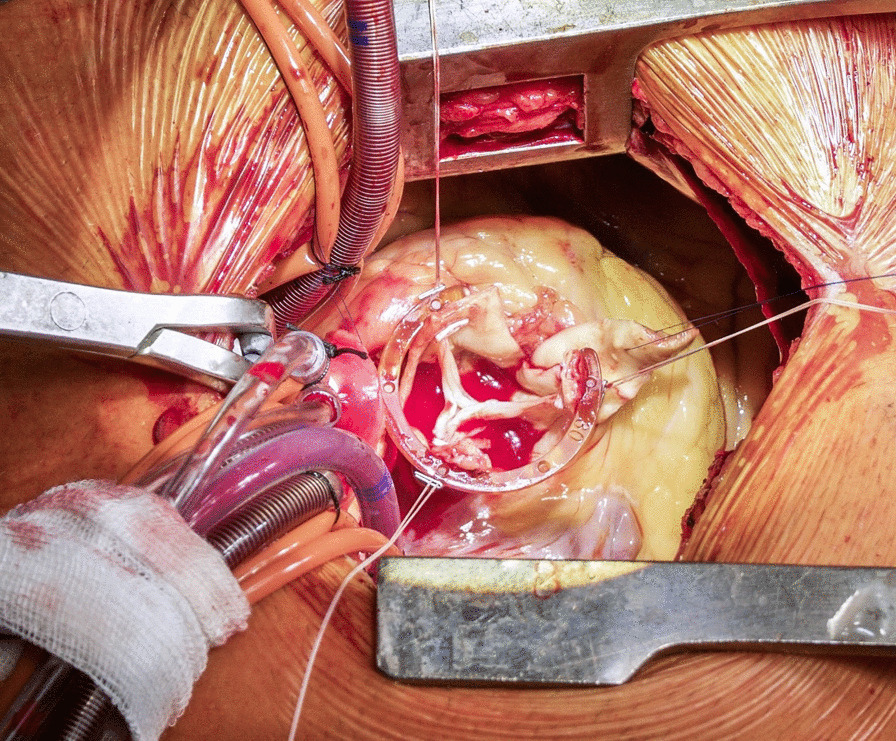
Intraoperative view of minimally invasive David procedure via ministernotomy

## A18 Case of tuberculous mycotic aortic aneurysm

### Batool Almoosawi^1^; Christopher Bayliss^2^; Jorge Mascaro^1^; Eshan Senanayake^1^

#### ^1^University Hospitals Birmingham/Queen Elizabeth Hospital, Birmingham, UK; ^2^Newcastle Upon Tyne NHS Foundation Trust, Newcastle Upon Tyne, UK

**Correspondence**: Batool Almoosawi

*Journal of Cardiothoracic Surgery 19(1):* A18

**Introduction:** Infection remains a rare cause of thoracic aortic aneurysms, with mycobacterium tuberculosis being one of the least commonly reported causative organisms. We present a case of a 31-year-old patient who was diagnosed with tuberculous mycotic aortic aneurysm affecting the aortic arch.

**Case presentation:** A 31-years-old man, previously diagnosed with, and treated for, tuberculous cervical lymphadenopathy in 2014, was investigated for a new onset hoarseness of voice, when a CT scan of the thorax revealed a necrotic nodal mass likely secondary to TB in the AP window/left hilum, involving the aortic arch with evidence of a pseudoaneurysm measuring 7.6 cm × 4 cm.

Multiple serial CT angiograms taken afterwards showed gradual increase in the size of the haematoma surrounding the aneurysmal sac. Echocardiogram studies showed an unaffected aortic valve and normal biventricular function.

Surgical intervention was done via a median sternotomy and involved a total arch and frozen elephant trunk replacement to reduce the risk of rupture.

**Comments:** TB mycotic aortic aneurysms are extremely rare, fatal, and can present with variable clinical features. The incidence of mycotic aortic aneurysms has been reported as 0.6–2% of all aortic aneurysms, with Mycobacteria accounting for only 2% of that [1].

Literature recommends that deterioration of patients already diagnosed with TB should prompt an emergent investigation of the aorta. [2] Treatment options include open-surgical or endovascular interventions, usually in combination with anti-TB therapy.
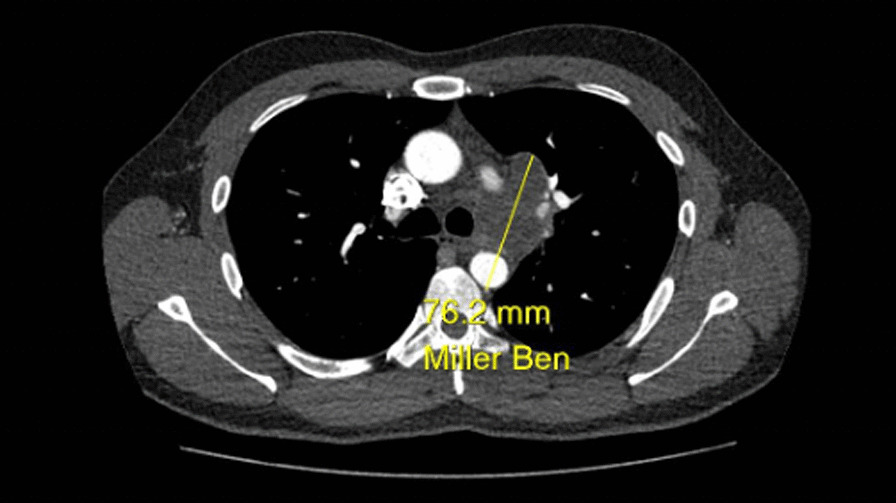


[1] Chen, M., Chung, C., Ke, H., Peng, C., Chien, W. and Shen, C., 2021. Risk of Aortic Aneurysm and Dissection in Patients with Tuberculosis: A Nationwide Population-Based Cohort Study. *International Journal of Environmental Research and Public Health*, 18(21), p.11075.

[2] Choudhary, S., Bhan, A., Talwar, S., Goyal, M., Sharma, S. and Venugopal, P., 2001. Tubercular pseudoaneurysms of aorta. *The Annals of Thoracic Surgery.*

*The patient has consented to this information being published in an open access journal.

## A19 Outcomes of re-do aortic root replacements: a contemporary single centre experience of 81 patients

### Daniel Fudulu; Roberto Natalia; Thomas Sullivan; Eltayeb Mohamed Ahmed; Cha Rajakaruna

#### Bristol Heart Institute, Bristol, UK

**Correspondence**: Daniel Fudulu

*Journal of Cardiothoracic Surgery 19(1):* A19

**Objectives:** There is limited data on the clinical outcomes of redo root replacement (rARR) and the clinical predictors for adverse outcomes. We report contemporary series from a single institution of rARR over the past decade.

**Methods:** In the period 2011–2022, 81 patients underwent rARR. Salvage procedures were excluded. Continuous variables were summarised as mean and standard deviation (SD) or median and interquartile range. A backward stepwise regression approach was used to select 8 final predictors which were included in the logistic regression model for mortality (Table 1).

**Results:** Median age was 54 (IQR: 34, 67) years and 59 (27%) patients were female. Urgent and emergency procedures were made up 39(48%) and 6 (7.4%) patients respectively. LV function was impaired in 26 (33%) cases. The indications were root aneurysm 36(44.4%), pseudoaneurysm 17 (20.9%), endocarditis 16 (19.5%), Chronic dissection 3 (3.7%), Valve failure 3(3.7%), PAU 1(1.2%) and acute aortic dissection 1 (1.2%). Median CPB and cross clamp time was 226(IQR: 177, 320) mins & 158 (IQR: 124,184) mins respectively. Types of operations were Composite ARR, Valve sparing ARR and homograft ARR in 72%, 12% and 16% cases respectively. In-hospital morality was 12(15%) patients. Aortic cross clamp time (OR: 1.02, CI 1.00–1.03, P = 0.025) was an independent predictor for in-hospital mortality (Table 1).

**Conclusions:** Redo aortic root replacement is associated with significant in-hospital mortality. In our analysis the only predictor for hospital morality was the prolonged aortic cross clamp time. Long term outcomes need to be described.
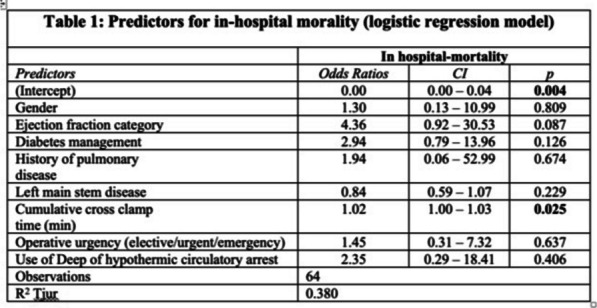


## A20 Initial experience with total aortic arch replacement: early to mid-term outcomes

### George Gradinariu; Sanjeet Singh; Darja Clinch; Rachael Forsythe; Charilaos-Panagiotis Koutsogiannidis; Kelvin Lim; Renzo Pessotto; Orwa Falah; Vincenzo Giordano

#### Royal Infirmary of Edinburgh, Edinburgh, UK

**Correspondence**: George Gradinariu

*Journal of Cardiothoracic Surgery 19(1):* A20

**Objectives:** This study reports our initial single-centre experience with total aortic arch replacement.

**Methods:** Consecutive patients undergoing elective and emergency total arch replacement were included. Demographic, perioperative data, 30-day and 1-year mortality alongside postoperative complications were retrospectively analysed.

**Results:** Between July 2018 and July 2022, 36 consecutive patients underwent total arch replacement in our institution. Indication for surgery was chronic type B dissection (14 patients), chronic type A (7 patients), arch aneurysms (11 patients) and acute aortic syndromes (4 patients). 6 patients (17%) had emergency surgery and 10 (28%) had concomitant aortic root surgery. Selective antegrade cerebral perfusion was used in 35 patients (97%). 10 patients (28%) had redo surgery. Frozen elephant trunk grafts were used in 28 patients (78%), while the conventional elephant trunk was used in 7 (19%). In-hospital or 30-day mortality in the entire cohort was 8.3% (3 patients) and 1-year survival was 78%.

Chest infections were the most common complication affecting 16 patients (44%) followed by atrial fibrillation in 10 (28%) and CVA/TIA in 5 (14%). Reoperation for bleeding occurred in 7 patients (19%). 5 patients (14%) required temporary haemofiltration or dialysis. The median LOS was 14.5 (IQR 9–21) days.

20 patients (56%) subsequently underwent a 2nd stage operation for the descending aorta (8 TEVAR and 12 open repairs). The median time between the 1st and 2nd stage procedure was 187 days (IQR:73–294). Overall survival at 3.5 years was 66%. Survival curves did not differ between chronic dissection vs aneurysms (p = 0.86) (Fig. 1), emergency vs elective surgery (p = 0.549) or redo vs first-time surgery (p = 0.132).

**Fig. 1:**
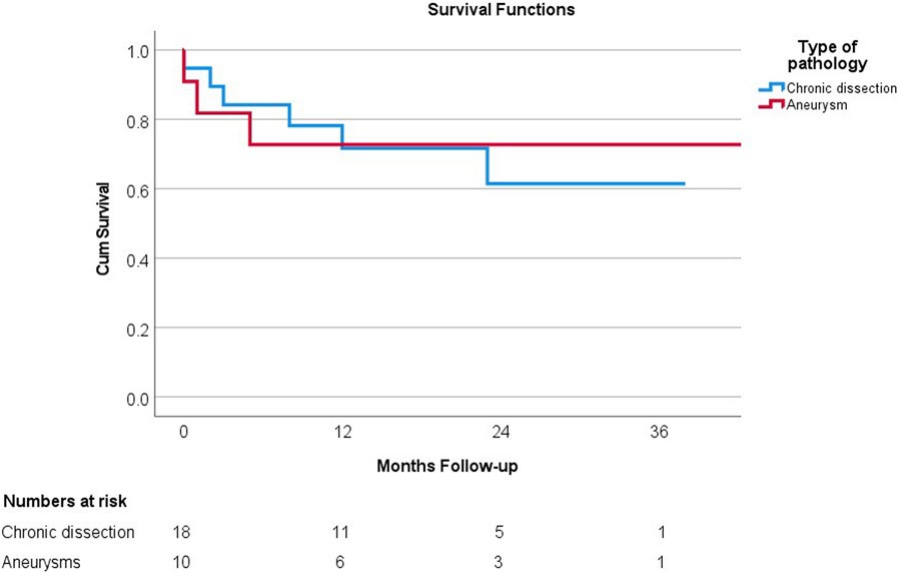
Kaplan–Meyer survival curves comparing total arch replacement outcomes in chronic dissection and aneurysmal pathology

**Conclusions:** Our initial experience shows comparable results with those reported in the literature for early and mid-term survival after total aortic arch replacement. Long-term follow-up and dedicated aorto-vascular MDT surveillance is needed.

## A21 Early experience in valve sparing aortic root replacement

### Ahmed Shazly; Arvind Singh; Hasnat Khan; Mohamed Osman; Pleasant Sunny; Sudhir Bhusari; Alberto Albanese; Alessia Rossi

#### Essex Cardiothoracic Centre, Basildon, Basildon, UK

**Correspondence**: Ahmed Shazly; Arvind Singh

*Journal of Cardiothoracic Surgery 19(1):* A21

**Objectives:** Valve sparing aortic root replacement became widely accepted and even favoured above the standard aortic root replacement for the treatment of Aortic root dilatation with healthy valve leaflets.

**Methods:** We are presenting our early experience of valve sparing root replacement in a low flow centre. Patients’ data was retrospectively collected and analysed. Operative data, post-operative complications and mortality were also included. All patients had immediate post-operative, 6 month, 1-year and 2-year follow up echocardiogram.

**Results:** From 2017–2020 a total of 34 cases of David procedure were done. 28 patients are males, Age ranged 23- 77 years (56 ± 8), BMI ranged 19–34 (30+/5.3). Preoperative NYHA class was I in 14 patients, seven cases were in NYHA II and 13 patients in NYHA class III.

Pre-operative EF ranged 23%-60% (53% ± 7.7%), Aortic annulus measurement ranged 18–45 mm (31 ± 5.3), Sinuses of Valsalva were 34–74 mm (49.8 ± 9.1 mm), STJ measured at 27-61 mm (42 ± 8.1 mm) and ascending aorta ranged 28-79 mm (46.8 ± 10.9). Circulatory arrest was used in 2 patients (5.8%). 14 patients had Bicuspid aortic valve while 20 patients had Tricuspid valve. Aortic valve leaflets height ranged 25–36 mm (30 ± 2.5), Coaptation length ranged 6–11 mm (8.3 ± 1.6 mm) and tubes used ranged 28–34 mm (30.5 ± 1.7 mm). Bypass time was between 143 and 487 min. (279.7 ± 77.3), Aortic clamp ranged 121–360 min (231.9 ± 55.5).

One patient died in ITU and 1 patient died 3 months post discharge.

Post-operative ventilation time ranged 2–39 h (9.7 ± 7.4 h).

ITU stay 10–980 h (78.3 with the exception of one patient who spent 147 days and died in ITU.

Post-operative complications included Multi-organ failure in 2 patients, 1 patient reopened for bleeding, 1 patient had stroke and 6 patients had PPM.

Follow up at 6-month one patient had severe AR another patient had moderate AR, 1-year echo showed severe AR in one patient with EF of 31% who needed redo AVR.

## A22 Perioperative outcome of urgent and emergency descending thoracic and thoracoabdominal aortic replacement

### Agni Salem; Jakub Marczak; Ayman Kenawy; Ahmed Othman; Omar Nawaytou; Deborah Harrington; Manoj Kuduvalli; Francesco Torella; Mark Field

#### Liverpool Heart and Chest Hospital, Liverpool, UK

**Correspondence**: Agni Salem

*Journal of Cardiothoracic Surgery 19(1):* A22

**Objectives:** Our goal is to describe our outcomes of urgent and emergency thoracic and thoracoabdominal aortic replacements from 1998 to 2022.

**Methods:** We retrospectively reviewed 516 patients. Our database was analysed for anyone who underwent a descending thoracic (DTA) or thoracoabdominal (TAAA) repair on urgent and emergency basis. Variables associated with 30-day mortality were identified among preoperative factors (age > 70, Female gender, NYHA class > 3, smoking, diabetes, hypercholesterolemia, hypertension, previous stroke, respiratory disease, TAAA Extent 2, peripheral artery disease, chronic renal disease, poor ejection fraction, previous aortic intervention, emergency surgery, use of CPB), on univariate analysis first, then on multivariate analysis.

**Results:** Out of 516 patients, 174 underwent emergency or urgent operation. Mean age was 58 years (± 14.52) and 37.9% (N = 66) patients were women. 58 patients underwent DTA replacement, 45 Extent 2 TAAA replacement and 29 Extent 1 TAAA replacement. 30-day mortality for urgent cases was: isolated DTA replacement—urgent 3.3% (n = 1) and emergency 31.6% (n = 6). For those with Extent 2 repairs, 30-day mortality was 28.1% (n = 10) for urgent patients and 50% (n = 4) for emergency operations. Patients operated on due to Extent 1 TAAA on urgent basis had survival with 0% 30-day mortality and those who were operated on emergency basis had mortality rate of 25% (n = 3).

Predictors of mortality were extent 2 replacement (OR of 4.0 [CI 1.6–10.0]), respiratory disease (OR 4.0 [1.2–13] and poor LVEF OR 24 [2.17–270.0]) and emergency status (OR 5.4 [CI 1.8–15.9]).

**Conclusions:** In selected cases urgent and emergency thoracoabdominal aortic repairs can be performed with acceptable risk. Extent of repair and its urgency along cardiovascular and pulmonary condition should be taken into consideration in the preoperative patient selection and decision-making process.

## A23 Acute type (A) aortic dissection repair with complex vascular ring: a rare case report

### Karim R Moawad; Muntashir Mahboob; Thomas Barker; Uday Dandekar; Sendhil K Balasubramanian

#### University Hospital Coventry and Warwickshire, Coventry, UK

**Correspondence**: Karim R Moawad

*Journal of Cardiothoracic Surgery 19(1):* A23

**Introduction:** Acute Aortic dissection with intramural hematoma (ADD-IMH) and impending rupture needs emergency surgery. Reporting a rare case of surgery of ADD-IMH with complex vascular ring and highlighting crucial perioperative strategies.

**Case report:** A 79-year-old patient had chest and back pain, CT aorta revealed acute intramural hematoma from ascending aorta to renal arteries with dissection flap, aberrant right subclavian arising from distal arch coursing retro-tracheal and common carotid trunk with an anomalous course of a right common carotid artery (RCCA) (Fig. 1).

**Fig. 1:**
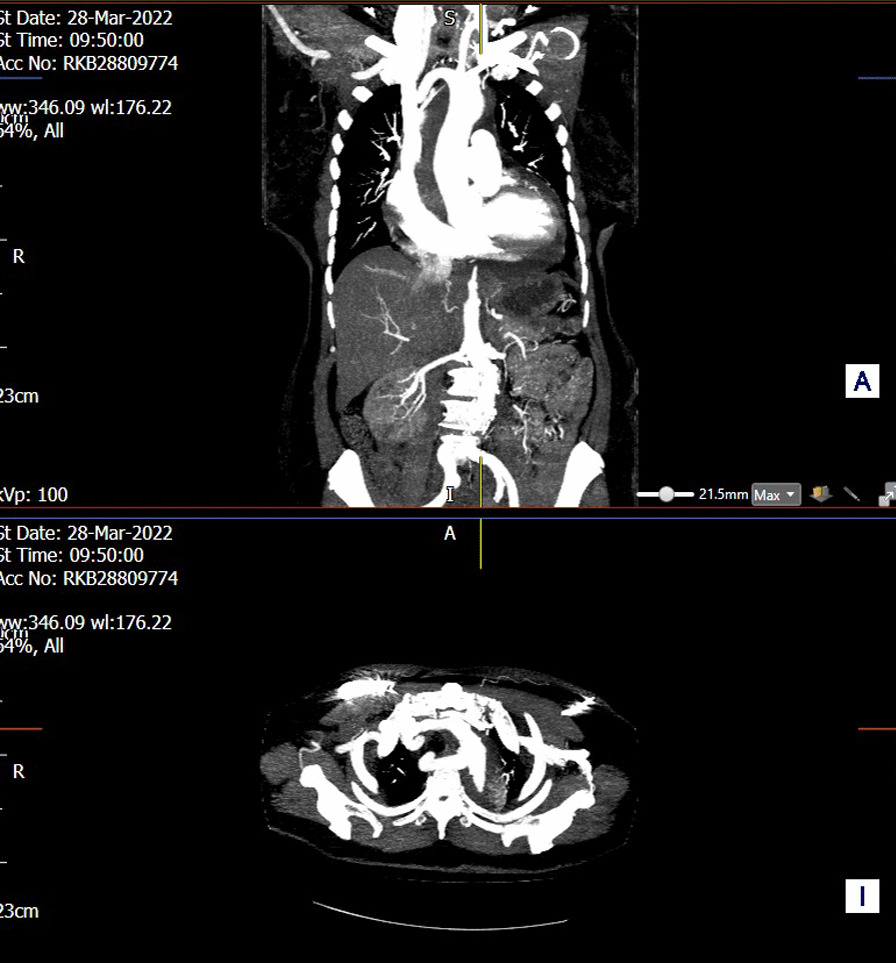
CT scan showing aberrant right subclavian artery arising from the distal arch coursing retro-tracheal forming a vascular ring and right common carotid artery taking an anomalous course with a common carotid trunk that was giving both carotids

ECHO revealed pericardial effusion, moderate AR & MR. Preoperatively developed paraplegia. Comorbidities: Hypertension, AAA repair, Permanent pacemaker.

Intraoperative findings: dilated ascending aorta 7 cm, contained hematoma partially ruptured, dissection tear at mid ascending aorta with adherent LA & RA, tricuspid aortic valve & root morphology preserved.

Procedure: CPB with right common carotid artery (arterial) with 8 mm graft and RA (Venous) while cooling to 23 ºC, aorta cross-clamped. Ante-grade cardioplegia given. Dissection flap, intramural hematoma in the ascending aorta and root removed. Ascending Aorta and proximal arch replaced with Valsalva graft. With DHCA continuous ante-grade cerebral perfusion. The distal anastomosis was performed on the proximal arch.

Patient had persistent paraplegia postoperatively and was referred to the regional spine center. Post-op CT scan and followed up 6 months satisfactory.

**Conclusions:** Rare case of type A acute aortic dissection repair with intramural haematoma in the context of the complex vascular ring so far not reported in literature. We recommend continuous antegrade cerebral perfusion strategy through arch vessels. Recent onset paraplegia is not a contraindication for this life-threatening condition.

Acknowledgements

The patient gave informed consent for their information to be published in an Open access journal.

This poster was possible because of the work of the cardiothoracic team at University Hospital Coventry and Warwickshire NHS Trust.

Authors have no financial disclosures to report.

References

1. De León-Luis J, Gámez F, Bravo C, Tenías JM, Arias Á, Pérez R, et al. Second-trimester foetal aberrant right subclavian artery: original study, systematic review and meta-analysis of performance in detection of Down syndrome. Ultrasound Obstet Gynecol. 2014 Aug;44(2):147–53.
2. Freed K, Low VH. The aberrant subclavian artery. AJR Am J Roentgenol. 1997 Feb;168(2):481–4.
3. Stone WM, Brewster DC, Moncure AC, Franklin DP, Cambria RP, Abbott WM. Aberrant right subclavian artery: varied presentations and management options. J Vasc Surg. 1990 Jun;11(6):812–7.
4. Weinberger G, Randall PA, Parker FB, Kieffer SA. Involvement of an aberrant right subclavian artery in dissection of the thoracic aorta: diagnostic and therapeutic implications. AJR Am J Roentgenol. 1977 Oct;129(4):653–5.

## A24 The complementary roles of open and endovascular repair of extent I–III thoracoabdominal aortic aneurysms in a United Kingdom aortic centre

### Eshan Senanayake; Martin Claridge; Maciej Juszczak; Jorge Mascaro; Donald Adam

#### University Hospitals Birmingham (UHB), Birmingham, UK

**Correspondence**: Eshan Senanayake

*Journal of Cardiothoracic Surgery 19(1):* A24

**Objectives:** To report the early and mid-term outcomes of open (OSR) and complex endovascular repair (cEVAR) for extent I-III TAAA in a UK aortic centre.

**Methods:** Single-centre retrospective study of consecutive patients with extent I-III TAAA treated between January 2009 and December 2021. Primary endpoint was 30-day/in-hospital mortality. Secondary end point was Kaplan–Meier estimates of mid-term survival. Data are presented as median (IQR). A P-value of < 0.05 was considered significant.

**Results:** 296 patients [176 men; median age 71 (65–76) years; median diameter 66 (61–75) mm] underwent elective (n = 222) or non-elective (n = 74) repair. OSR patients (n = 66) were significantly younger with a higher incidence of heritable thoracic aortic disease and chronic dissection, while cEVAR patients (n = 230) had a significantly higher incidence of coronary, pulmonary and kidney disease. Overall, in-hospital mortality after elective and non-elective repair was 3.2% (n = 7/222) and 23% (n = 17/74) with no significant difference comparing treatment modalities after elective [OSR 6.5% vs. cEVAR 2.3%; p = 0.142] or non-elective repair [OSR 25% vs. cEVAR 20.3%; p = 0.801]. Major non-fatal complications occurred in 15.7% (43 of 274) of operative survivors [OSR 39.7% vs. cEVAR 9.3%; p < 0.0001]. Median follow-up was 52 months (23–78). Estimated (± SE) survival at 1, 3 and 5 years was 89.6% (± 2.0%), 76.6% (± 2.9%) and 69.0% (± 3.2%) after elective repair, and 67.6% (± 5.4%), 52.1% (± 6.0%) and 41.0% (± 6.2%) after non-elective repair. 5-year survival after elective and non-elective repair in patients < 70 years was 85.6% (± 3.9%) and 65.4% (± 9.2%) compared to 58.0% (± 4.9%) and 23.7% (± 7.0%) in those aged > 70 years.

**Conclusions:** A multi-disciplinary team offering both OSR and cEVAR can deliver comprehensive care for extent I–III TAAAs with low early mortality and good mid-term survival. Further studies are required to determine the optimal relative roles of the treatment modalities.

## A25 Outcomes of Major aortic surgery in the elderly patient

### Ahmed Shazly; Arvind Singh; Sudhir Bhusari; Alberto Albanese; Hasnat Khan; Alessia Rossi; Younus Qamar

#### Essex Cardiothoracic Centre, Basildon, Basildon, UK

**Correspondence**: Ahmed Shazly

*Journal of Cardiothoracic Surgery 19(1):* A25

**Objectives:** Major aortic surgery is one of the most complex challenges a cardiac surgeon can undertake. High mortality is a feature of both elective and emergent cases with the elderly patient considered to be at higher risk than their younger counterpart.

**Methods:** We performed a retrospective analysis of all patients undergoing major aortic surgery between October 2014 and September 2016 inclusive. The patients were divided into 2 groups, those under the age of 70 years (group A), and those 70 years and above (group B). Demographic, admission length, complication and mortality data was collected for each group and statistically analysed.

**Results:** The 22 patients in group A had an average additive EuroSCORE of 7.45, with those is group B having a significantly higher score of 11.21 (P < 0.001). Similar number of patients underwent emergent surgery in each group (32% in group A vs 35% in group B). Group B had more complex aortic surgery with four patients requiring deep hypothermic circulatory arrest for aortic arch surgery compared to no patients in group A (P = 0.019). There was no significant difference between, mortality, major complication rates or length of admission between the 2 groups. Mortality in group B was actually lower than that of group A at 4% and 18% respectively but no statistical significance could be attributed to this.

**Conclusions:** There was no significant difference in post-operative outcomes for patients aged over 70 compared with their younger counterparts, despite this group having more complex aortic surgery and having higher predictive risk. Both elective and emergent major aortic surgery should be considered in the older patient, and age itself should not be used as the sole exclusion criteria.

## A26 Distal repair after total aortic arch replacement with frozen elephant trunk in patients with chronic multi-level thoracic aortic disease

### Eshan Senanayake^1^; Robert-James Doonan^2^; Martin Claridge^1^; Maciej Juszczak^1^; Francesco Torella^3^; Jorge Mascaro^1^; Mark Field^2^; Donald Adam^1^

#### ^1^University Hospitals Birmingham (UHB), Birmingham, UK; ^2^Liverpool Heart and Chest Hospital, Liverpool, UK; ^3^Liverpool University Hospitals, Liverpool, UK

**Correspondence**: Eshan Senanayake

*Journal of Cardiothoracic Surgery 19(1):* A26

**Objectives:** To examine the management of distal disease after total arch replacement with frozen elephant trunk (TAR + FET) in patients with chronic degenerative or post-dissection multi-segment thoracic aortic disease.

**Methods:** Two-centre retrospective study of consecutive patients treated between January 2010 and December 2019. Primary endpoint was 30-day/in-hospital mortality. Secondary end point was Kaplan–Meier estimates (± SE) of mid-term survival. Data are presented as median (IQR). A P-value of < 0.05 was considered significant.

**Results:** 158 patients [72 men, median age 70 (64–75), median descending thoracic aortic diameter 58 mm (46–68)] underwent elective (n = 107) or non-elective (n = 51) repair. Peri-operative mortality was 8.4% (n = 9) after elective, and 13.7% (n = 7) after non-elective repair. Median follow-up was 46 months (26–75). There were 7 early deaths in 74 (46.8%) patients with a primary distal seal, and 9 (13.4%) survivors underwent distal aortic repair at median 25 months (15–48). There were 9 early deaths in 84 patients with no primary distal seal, and 42 (56%) underwent distal repair at median 7 months (4–22). Of the remaining 33 patients, 23 reached size threshold: 7 were unfit, 8 were fit but died before repair, 1 declined repair, 4 were awaiting repair, and 3 were lost to follow-up. Survival at 1, 3 and 5 years was 89.7% (± 2.9%), 80.0% (± 3.9%) and 70.6% (± 4.7%) after elective, and 58.8% (± 6.9%), 46.1% (± 7.1%) and 43.2% (± 4.3%) after non-elective repair. There was no significant difference in survival comparing patients with or without a primary distal seal (p = 0.38); or those who had and did not have distal repair (p = 0.09).

**Conclusions:** TAR + FET provided a durable one-stage repair in 40% of patients. Late distal repair was indicated in over 50% of patients, 20% of whom were considered unfit or died in the interval. Further work is required to reduce the proportion of patients who fail to proceed to distal repair.

## A27 Long-term survival after urgent and elective aortic root, ascending and aortic arch surgery

### Kathryn Fisher^1^; Ulrich Von Oppell^2^; Mesbah Rahman^2^; Dheeraj Metha^2^; Michail Koutentakis^2^; Indu Deglurkar^2^

#### ^1^Cardiff University, Cardiff, UK; ^2^Cardiff and Vale University Health Board, Cardiff, UK

**Correspondence**: Kathryn Fisher

*Journal of Cardiothoracic Surgery 19(1):* A27

**Objectives:** Analyse 10-year survival for urgent and elective major aortic surgery at University Hospital of Wales from 2012 to 2022. Identify significant risk factors for post-operative all-cause mortality and evaluate their relevance over time.

**Methods:** 391 patients (237 male, 118 female) underwent open surgery (236 elective, 155 urgent) involving the ascending aorta (335/391), aortic root (207/391) and/or aortic arch (32/391) between 1st April 2012 and 31st March 2022. Kaplan–Meier survival curves were generated to calculate the probability of survival at various intervals post-operatively. Chi-squared tests identified significant risk factors for all-cause mortality in-hospital, at 30-days, 1-year, 3-years, 5-years, and 8-years.

**Results: ​**Overall non-risk adjusted survival at 10-years was 89.5%. Survival for elective procedures ranged from 98.3% (30-days) to 90.4% (10-years). Survival for urgent procedures ranged from 98.1% (30-days) to 87.4% (10-years). 14/32 factors were statistically significant (p < 0.05) at one or more intervals post-operatively. Short-term survival is not independent of previous cardiac surgery, hypertension, GI disease, VT/VF, heart rhythm, or infective endocarditis. Intermediate-term survival is not independent of angina, dyspnoea, heart failure, diabetes, previous cardiac surgery, renal impairment, or infective endocarditis. Long-term survival is not independent of angina, previous MI, heart failure, diabetes, previous DVT, or severity of aortic stenosis.

**Conclusions:** 89.5% survival at 10-years following urgent and elective major aortic surgery compares favourably with international publications of 68.7–80.9%. Risk of mortality is non-linear and is highest in the immediate post-operative period. Individual patient survival is multifactorial and influenced by presenting symptoms, established cardiac risk factors and disease severity. Further investigations can determine causal relationships and compare life expectancy to the general population.

**Adult Cardiac Aortic Valve**

## A28 A semi-continuous suture technique is not inferior to an interrupted suture technique in aortic valve implantation: a propensity matched analysis

### Alex Smith; Philip Hartley; Adeyemi Olayiwola; Andrew Selvaraj; Aung Oo; Rakesh Uppal; Damian Balmforth

#### St. Bartholomew's Hospital, London, UK

**Correspondence**: Philip Hartley

*Journal of Cardiothoracic Surgery 19(1):* A28

**Objectives:** It is a common held belief that an interrupted suture technique is superior to a semi-continuous technique for valve implantation due to lower rates of para-valvular leak and reoperation. Our aim was to investigate whether one implantation suture technique was superior.

**Methods:** A retrospective cohort study was conducted using a prospectively maintained single institutional database. All patients undergoing aortic valve replacement, with or without coronary artery bypass were included. A propensity matched analysis was performed for the primary outcomes of re-operation rate for valvular dysfunction and in-hospital mortality, matched on baseline EuroSCORE II, age and procedure performed. A multivariate cox-proportional hazards model was constructed to explore long-term survival adjusted for age, procedure, EuroSCORE II and gender.

**Results:** Between July 2017 and September 2021, 1474, patients met the inclusion criteria. The mean age was 65 and 411 (28%) patients were female. 1016 (69%) underwent isolated aortic valve replacement and 458 (31%) concomitant coronary artery bypass grafts. An interrupted suture technique was used in 709 (48%) cases and semi-continuous in 765 (52%). In the propensity matched analysis there was no significant difference between the cohorts in re-operation rate for valvular dysfunction [semicontinuous n = 8, interrupted n = 5, p = 0.25]. Neither was there any significant difference seen in in-hospital mortality between the two cohorts [4.7% semicontinuous, 3.7% interrupted, p = 0.32]. However, mean cross-clamp duration and cardiopulmonary bypass duration were significantly reduced in the semi-continuous group. The adjusted cox-proportional hazards model showed no difference in overall long-term survival between the cohorts (p = 0.65).

**Conclusions:** A semi-continuous suture technique for aortic valve implantation is not associated with increased reoperation rates or reduced mortality compared to the commonly employed interrupted technique.

## A29 Evaluation of three generations of tissue aortic valve haemodynamics: a propensity matched study

### Rickesh Karsan^1^; Dorina Roy^2^; Reuben Jeganathan^1^

#### ^1^Department of Cardiothoracic Surgery, Royal Victoria Hospital, Belfast, UK; ^2^Royal Victoria Hospital, Belfast, UK

**Correspondence**: Rickesh Karsan

*Journal of Cardiothoracic Surgery 19(1):* A29

**Objectives:** Recent data suggests 2nd generation rapid deployment aortic valves have superior haemodynamic profiles compared to conventional bioprostheses. The German Aortic Valve registry suggests post-operative haemodynamic profiles are comparable to transcatheter valves. We aimed to analyse the haemodynamic performance of three generations of aortic bioprosthetic valves including 1st generation conventional tissue prosthesis, 2nd generation rapid deployment valve, and 3rd generation bioprosthesis with advanced tissue technology.

**Methods:** Patients who underwent tissue aortic valve replacement from 2018 to 2020 for native valve aortic stenosis were identified. Pre and 1-year post-operative haemodynamic data including peak gradient (PG), mean gradient (MG), peak velocity (PVel), velocity time integral ratio (VTI) and aortic valve area (AVA) were collected. 1:1 propensity matching based on based on age, gender, body surface area, EuroSCORE II, pre-operative rhythm, left ventricular function was performed. Pre and post-operative haemodynamic data was compared via unpaired T-test and ANOVA.

**Results:** 234 cases were identified: Conventional bioprosthesis = 131, rapid deployment valve = 74 and bioprosthesis with advanced tissue technology = 29. Mean age was 69(± 9.43); mean EuroSCORE II was 4.17(± 0.42). Post-operative haemodynamics were significantly improved for all 3 valves at 1 year follow-up (p < 0.05). Propensity matched analysis showed significantly improved post-operative peak and mean gradients and peak velocities when comparing, 2nd generation rapid deployment valve to 1st generation (p < 0.001 PG; p < 0.001 MG; p < 0.001 PVel), and 3rd generation valves (p = 0.006 PG; p = 0.002 MG; p = 0.004 PVel). VTI and AVA were found to be significantly improved (p < 0.001 and 0.005) compared to 1st generation valves (Fig. 1).

**Fig. 1:**
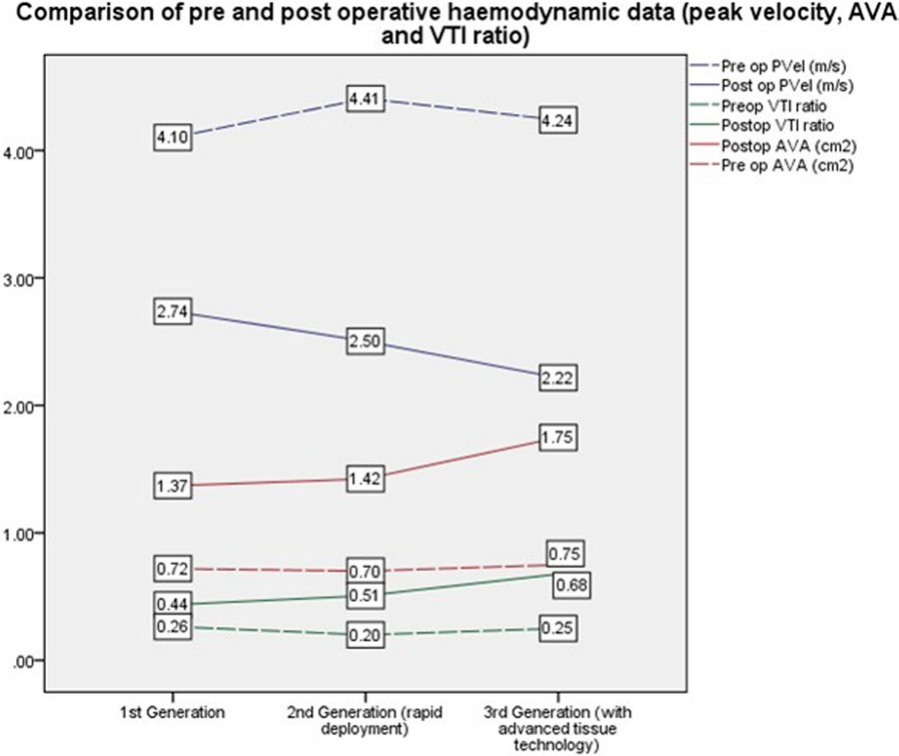
Comparison of pre and post-operative haemodynamic profiles for 3 generation of tissue aortic valve in patients with isolated aortic stenosis. 2nd generation rapid deployement valve bioprosthesis shows the most marked improvement in haemodynamic profile at one year follow up for, aortic valve area (AVA), mean gradient (MG) and peak gradient (PG) (P < 0.05) when compared to 1st and 3rd generation

**Conclusions:** Rapid deployment aortic valves are shown to give the greatest post-operative haemodynamic performance at 1 year. This is likely due to sub-annular remodelling of the LVOT.

## A30 Single centre outcomes after biological stented and sutureless AVR

### Roberto Marsico; Riccardo Abbasciano; Rachel Chubsey; Tavid Westerhoff-Mason; Hiwa Sherzad; Viktor Zlocha

#### Glenfield Hospital; University Hospital Leicester NHS Trust, Leicester, UK

**Correspondence**: Roberto Marsico

*Journal of Cardiothoracic Surgery 19(1):* A30

**Objectives:** Several recent series support the implant of sutureless valve for aortic valve stenosis treatment usually describing the outcome of one of the two most utilised sutureless valves. Our study aims at comparing early post-operative clinical outcomes and long-term follow up in patients undergoing aortic valve replacement (AVR) using conventional vs sutureless valves (Perceval-LivaNova or Edwards-Intuity).

**Methods:** We report a single unit experience between 2015 and 2022 of a total of 1937 consecutive patients who underwent an AVR. To minimize bias, propensity score matching was conducted and two groups of 275 patients with similar preoperative characteristics were matched: Standard aortic valve replacement (AVR) and Sutureless (SUAVR). Early outcome assessed included: CVA, re-exploration for bleeding, low cardiac output, deep sternal wound infection, dialysis, length of hospital stay and long-term survival.

**Results:** Our cohort characteristics were: age (74.5 ± 6.4), gender (F: 56%), BMI (29.2 ± 5.32). AVR group showed increased time (minutes) of: CBP (107.5 ± 46.9 vs 92.91 ± 38.7; p < 0.001) and cross-clam time (75.7 ± 28.4 vs 56.3 ± 24.1; p < 0.001). In early outcome AVR shown higher incidence of: CVA (8, 2.9% vs. 1, 0.4%; p < 0.001), re-exploration for bleeding (20, 7.3%, vs 10, 3.6%; p = 0.05) and dialysis (16, 5.8% vs. 7, 2.5%; p = 0.05). Postoperative permanent pacemaker (PPM) implantation at 30 days was increased in SUAVR (20, 7.2% vs. 9, 3.3%; p = 0.03).

No difference between the groups were found in terms of: hospital length of stay, deep wound infection and in-hospital mortality as well as overall six years survival for both unmatched (p = 0.12) and matched (p = 0.42) population.

**Conclusions:** Our analysis in keeping with literature showed that SUAVR offering overall reduced surgical time and associated complications with no differences observed in length of stay, short- and long-term survival but is associated with increased rate of PPM implantation (Table 1).

**Table 1:** Peri-operative and Post-operative Outcomes: AVR had a higher incidence of: CVA (8, 2.9% vs 1, 0.4%; p < 0.001), re-exploration for bleeding (20, 7.3%, vs 10, 3.6%; p = 0.05) and dialysis (16, 5.8% vs 7, 2.5%; p = 0.05)

	Total Cohort n = 550	AVR n = 275	SUAVR n = 275	P
In-hospital Mortality (%)	16 (2.9%)	10 (3.6%)	6 (2.2%)	0.31
Cross-clamp time Mean (SD)	77.4 (32.2)	75.7 (28.4)	56.3 (24.1)	< 0.001
CPB time Mean (SD)	110.1 (47.5)	107.5 (46.9)	92.91 (38.7)	< 0.001
IABP (%)	28 (5.1%)	13 (4.3%)	15 (5.4%)	0.69
New CVA (%)	9 (1.6%)	8 (2.9%)	1 (0.4%)	< 0.001
Length of stay Mean (SD)	13.1 (9.9)	13.7 (11.1)	12.5 (8.5)	0.13
Bleeding (re-exploration) (%)	30 (5.4%)	20 (7.3%)	10 (3.6%)	0.05
Dialysis (%)	23 (4.6%)	16 (5.8%)	7 (2.5%)	0.05
PPM in 30 days (%)	29 (5.3%)	9 (3.3%)	20 (7.2%)	0.03

No significant difference was seen with regards to hospital length of stay, deep wound infection and in-hospital mortality. However, there was an increased incidence in rates of permanent pacemaker insertion (PPM) at 30-days in the SUAVR cohort (9,3.3% vs 20, 7.2% p = 0.03)

## A31 Ten year outcomes of trifecta and perimount magna ease aortic valve bioprostheses: a propensity score matched comparative study

### Mukesh Karuppannan; Rebecca Taylor; Antony Walker; Amal Bose

#### Blackpool Victoria Hospital, Blackpool, UK

**Correspondence**: Mukesh Karuppannan

*Journal of Cardiothoracic Surgery 19(1):* A31

**Objectives:** This study compared the early and ten-year outcomes of Trifecta and Perimount Magna Ease aortic prosthesis.

**Methods:** Patients who underwent aortic valve replacement using the Trifecta and Perimount Magna Ease valves between May 2011 and December 2021 in our centre were included. Patients with concomitant valvular/aortic procedures and reoperations were excluded. Propensity score based matching and comparison was done between the two groups. The primary endpoints were 10-year survival and reoperation rate and secondary endpoints were perioperative complications and 30-day mortality.

**Results:** Out of the 1684 patients (mean age, 72.5 ± 8.5 years; Males 65.3%), 438 received the Trifecta and 1246 received the Perimount valve. After propensity scoring, 406 patients in each group were matched. There was no significant difference in the survival rate at 10 years (50.3% for Perimount vs 43.9% for Trifecta, p = 0.73). At 10 years, the Trifecta cohort had a significantly higher risk of repeat aortic valve replacement for all cause (6.3% vs 0.5%, p = 0.011) and the median time to reoperation was 4.75 years (IQR-4.96) vs 3.66 years (IQR-3.12) for Perimount. The median time to reoperation for structural valve failure in the Trifecta group was 5.6 years (IQR-4.92). There were no instances of reoperation for structural valve failure in the Perimount group. The 30-day mortality was similar between both the groups (96.8%, p = 0.73). Patients receiving Trifecta had significantly shorter bypass (p = 0.04) and cross-clamp times (p < 0.001), less likely to need blood products (p = 0.04), more likely to develop atrial fibrillation and require a permanent pacemaker (p = 0.05).

**Conclusions:** The Trifecta valve is associated with a higher occurrence of repeat operation due to structural valve failure compared with the Perimount Magna Ease valve. Further comparative studies with echocardiographic data on structural valve deterioration are needed to confirm these findings.

## A32 Automatic knot fastener in valve interventions: insights from a multicentre retrospective study

### George Gradinariu^1^; Rashmi Birla^2^; Hussein El-Shafei^3^; Adam Szafranek^4^; Ulrich Von Oppell^5^; Stephen Clark^6^

#### ^1^Royal Infirmary of Edinburgh, Edinburgh, UK; ^2^Golden Jubilee National Hospital, Glasgow, UK; ^3^Aberdeen Royal Infirmary, Aberdeen, UK; ^4^Nottingham University Hospitals, Nottingham, UK; ^5^University Hospital of Wales, Cardiff, UK; ^6^Freeman Hospital, Newcastle upon Tyne, UK

**Correspondence**: George Gradinariu

*Journal of Cardiothoracic Surgery 19(1):* A32

**Objectives:** The Cor-Knot automated knot fastener was designed as an adjunct to valve surgery to reduce cardio-pulmonary bypass (CBP) and cross clamp (XC) times and facilitate minimally invasive access. The aim of this study is to explore the safety of Cor-Knot in a large multi-centre real-life cohort of patients.

**Methods:** All patients undergoing valve interventions between January 2014 and February 2020 using the Cor-Knot device at four UK Cardiothoracic Units were included. Conventional sternotomy, minimal access, redo surgery and combined procedures (CABG, aortic surgery) were included. The primary outcome was severity of paravalvular leak (PVL) at discharge and during follow-up. Secondary outcomes were in-hospital and medium-term mortality and reoperations.

**Results:** 613 patients were included. 731 valves were operated using Cor-Knot: 404 (55%) aortic valves, 253 (35%) mitral valves (52 repairs with ring) and 73 (10%) tricuspid valves. 145 patients (20%) had concomitant CABG, 28 patients (5%) redo surgery, 57 (9%) surgery of the aorta and 34 patients (6%) had minimal-access surgery.

Pre-discharge echo was available for 416 patients (68%) and long-term follow-up data for 449 patients (73%). No cases of severe PVL pre-discharge were seen (Table 1). Median CBP and XC times for the entire cohort were 108 and 74 min. At a mean follow-up of 28 months, four mitral prostheses had severe PVL and three of them had subsequent reoperations. Endocarditis during follow-up was 2.9%. New permanent pacemaker rate was 5%. In-hospital mortality was 4.6% and medium-term survival at 28 months was 86%.

**Table 1:** Early and late rates of paravalvular leak after using CorKnot for aortic and mitral valve procedures

Valve intervention	No PVL	Trace PVL	Mild PVL	Moderate PVL	Severe PVL
Aortic—pre-discharge	95%, 280/296	3%, 9/296	2%, 6/296	0.3%, 1/296	0%, 0/296
Mitral—pre-discharge	81%, 136/148	2%, 3/148	2.7%, 4/148	3.4%, 5/148	0%, 0/296
Aortic—long-term	90%, 264/290	5.2%, 15/290	2.4%, 7/290	1.4%, 4/290	0%, 0/290
Mitral—long-term	83%, 133/145	2.8%, 4/145	1.4%, 2/145	1.4%, 2/145	2.8%, 4/145

**Conclusions:** This is the largest study of Cor-Knot yet reported and its use appears safe with rates of PVL similar to those reported in the literature. Cor-Knot can be successfully used in complex multivalve procedures where a reduction in CPB and XC times may impact patient outcomes or when access is challenging and knot tying is technically difficult. Long term, prospective trials are required.

## A33 Early follow-up of patients after elective aortic root enlargement for prevention of patient-prosthesis mismatch

### Mohamed Sherif; Mohamed Shoeib; Yama Haqzad; Massimo Capoccia; Walid Elmahdy

#### Leeds General Infirmary, Leeds, UK

**Correspondence**: Mohamed Sherif

*Journal of Cardiothoracic Surgery 19(1):* A33

**Objectives:** To evaluate the outcomes of patients who underwent aortic root enlargement (ARE) electively to prevent Patient-Prosthesis Mismatch (PPM). These patients were discussed pre-operatively in a specific MDT for small valves and planned for elective AVR+ aortic root enlargement. We report early follow-up outcomes of up to two years.

**Methods:** This is a retrospective study of 16 patients with a preoperative predicted moderate to severe PPM (between Feb 2020-Aug 2022). After discussion at the valve MDT, they underwent planned AVR + aortic root enlargement (ARE). Pre and post-operative data were collected from the cardiothoracic database. IBM SPSS statistics version 27 software was used for data analysis.

**Results:** The mean age of 68.8 years (61-77 years), mean EuroSCORE II of 10.6 (3.43–39.62), SD 8.5. Preoperative mean aortic valve area of 0.59 ± 0.30 cm2, a mean gradient of 60 ± 23 mmHg and peak gradient of 94 ± 35 mmHg. The mean aortic annulus size was 17 (15–20) mm.

The mean valve size implanted was 23 ± SD 1.15 (19–23). The mean bypass time and aortic clamp times were 138 (88–205) min, and 96 (70–132) min, respectively. All patients had improvement in the ejection fraction with mean EF of 58 ± 4.8.

There was a statistically significant increase in mean valve size post aortic root enlargement 5.3 mm (SD 1.9 mm) (p < 0.001). On postoperative echocardiography, there were statistically significant drops in mean pressure gradients across the aortic valve (MPG) (from 57 ± 21 mmHg to 9.8 ± 2.56 mmHg, (p < 0.001) and peak pressure gradient (from 92 ± 33 to 19.5 ± 4.4 mmHg, p < 0.001). No patients following ARE had moderate/severe PPM.

At up to two years follow-up, there is no mortality. The rate of the paravalvular leak 0%. Only one patient required a permanent pacemaker for a complete heart block. There were no cerebrovascular accidents or renal and respiratory failure.

**Conclusions:** Our series suggests that elective aortic root enlargement is safe.

## A34 Incidence and clinical outcomes of infective endocarditis post-transcatheter aortic valve implantation (TAVI): a tertiary centre experience

### Omar Zibdeh; Nader Moawad; William Carr; Clinton Lloyd

#### Derriford Hospital, Plymouth, UK

**Correspondence**: Omar Zibdeh

*Journal of Cardiothoracic Surgery 19(1):* A34

**Objectives:** Infective endocarditis (IE) is now a recognised complication after transcatheter aortic valve implantation (TAVI). However, the data remains scarce. We have therefore conducted a retrospective analysis of its incidence, microbiological profile and the clinical outcomes at our centre over a 3-year period with the aim of formulating a contemporary profile of its epidemiological trends.

**Methods:** We have retrospectively analysed data of all patients that have undergone a TAVI between October 2019 and October 2022 (n = 663). The endpoints assessed were the incidence of IE, its microbiological profile, route of management, and clinical outcomes including surgical intervention.

**Results:** The incidence of IE was 1.66% (11 patients). Only 54.5% of which had radiological evidence of IE (6 patients). The mean time of presentation was 362 days (± 258 days) from the TAVI operation date. Enterococcus faecalis and Streptococcus species (3 strains) were the most common causative organisms (36.4% each), followed by Staphylococcus aureus (18.2%), and one case of Candida parapsilosis (9.1%). 100% of cases were medically managed with prolonged intravenous antibiotics. The in-hospital mortality was 27.3%.

**Conclusions:** We have demonstrated significant incidence of TAVI endocarditis (1.66%) with significant in-hospital mortality (27.3%). Enterococcus faecalis was the most isolated organism. TAVI endocarditis can be managed medically with surgical intervention to be considered on an individual case basis if required.

## A35 Minimal access aortic valve replacement is a safe alternative to a conventional full sternotomy approach

### Asmita Singhania; Gokul Raj Krishna; Marcus Taylor; Rajamiyer Venkateswaran

#### Wythenshawe Hospital, Manchester, UK

**Correspondence**: Asmita Singhania

*Journal of Cardiothoracic Surgery 19(1):* A35

**Objectives:** Conventional aortic valve replacement via median sternotomy (con-AVR) is a safe and well-established surgical procedure. More recently, minimally invasive approaches for AVR (mini-AVR) have been undertaken with increased frequency. The aim of this study was to compare our short and mid-term outcomes for con-AVR versus mini-AVR.

**Methods:** All consecutive patients undergoing isolated AVR performed by a single surgeon at Manchester University NHS Foundation Trust between 2010 and 2019 were included. Primary outcomes were 90-day, 1-year and 3-year mortality. Secondary outcomes were cardiopulmonary bypass (CPB) and cross-clamp times, post-operative complications and post-operative length of stay (PLOS). Univariable analysis was undertaken to assess the difference in variables and outcomes between the con-AVR and mini-AVR groups. Statistical analysis was performed using SPSS version 28.

**Results:** In total, 169 patients underwent surgery, of whom 55% received mini-AVR (n = 93). There was no significant difference between groups for mean age, gender, comorbidities, ventricular dysfunction and mean implanted valve size. Whilst there was no significant difference in cross-clamp duration, the CPB time was significantly shorter in the mini-AVR group (101.0 ± 19.0 min vs 111.6 ± 40.4 min, p = 0.038). Overall, 90-day, 1-year and 3-year mortality were 1.2% (n = 2), 3.6% (n = 6) and 10.1% (n = 17), respectively. There was no significant difference in mortality between groups. There was also no significant difference in the rate of complications (re-exploration, atrial fibrillation and permanent pacemaker implantation) or PLOS.

**Conclusions:** This study has demonstrated acceptable short and mid-term outcomes after AVR and supports the use of mini-AVR as a safe alternative to con-AVR.

## A36 Aortic valve replacement with or without simultaneous coronary artery bypass in octogenarians: long-term survival

### Davorin Sef^1^; Vladimir Trkulja^2^; Sirr Ling Chin^3^; Szabolcs Miskolczi^3^; Theodore Velissaris^3^; Suvitesh Luthra^3^

#### ^1^University of Southampton NHS Foundation Trust, Southampton, UK; ^2^Medical School, University of Zagreb, Croatia, EU, Zagreb, Croatia; ^3^University Hospital Southampton NHS Foundation Trust, Southampton, UK

**Correspondence**: Davorin Sef

*Journal of Cardiothoracic Surgery 19(1):* A36

**Objectives:** Despite the fact that transcatheter approach in octogenarian patients with aortic stenosis has been increasing, impact of concomitant coronary artery bypass grafting (CABG) to aortic valve replacement (AVR) is still debated. We analysed the characteristics and long-term survival of octogenarians undergoing isolated AVR and AVR + CABG.

**Methods:** All elderly patients (> 80 years) who underwent AVR with or without concomitant CABG at our tertiary cardiac centre between 2000 and 2022 were included, and their characteristics and outcomes analysed. Redo cases were excluded.

**Results:** A total of 1040 patients who underwent AVR (83 ± 3 years, 42.0% males, body mass index [BMI] 26.7 ± 4.8) and 1140 with AVR + CABG AVR (84 ± 3 years, 65.8% males, BMI 26.7 ± 4.4) were included in our study. Perioperative mortality in AVR and AVR + CABG group was 2.6% and 4.39% (RD 1.41%; 95%CI -0.09 to 2.93), respectively. There was no significant difference in cumulative 10-year postoperative mortality (HR = 1.01; 95% CI 0.90–1.13) when adjusted for age, gender and BMI.

**Conclusions:** There was no significant difference in long-term survival of octogenerians who underwent AVR or AVR and CABG. However, CABG added to AVR was associated with higher perioperative mortality.
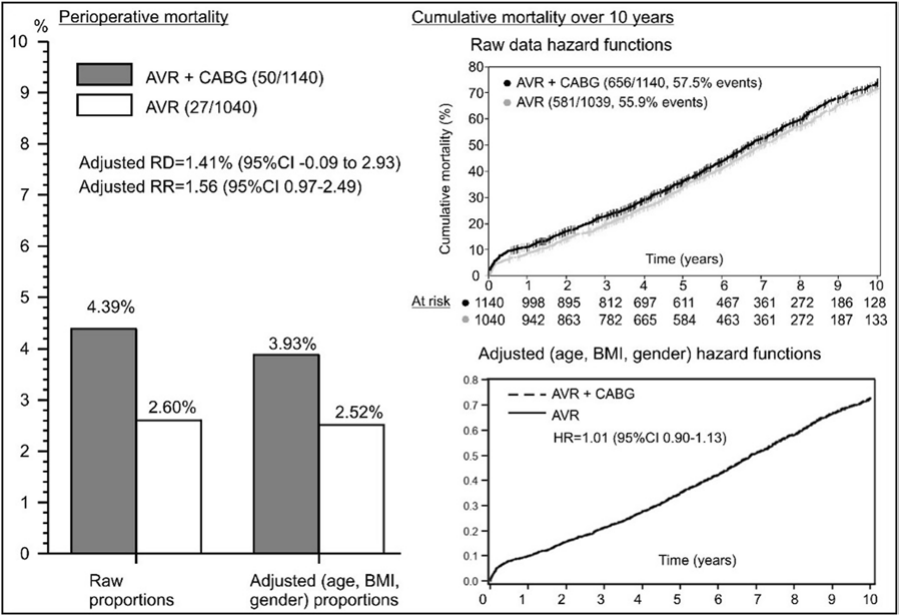


## A37 Development of a risk model for isolated surgical aortic valve replacement for severe aortic stenosis

### Khurum Mazhar; Bazegha Qamar; Ali Mohamed; Hosam Ahmed; Mohsin Uzzaman; Prakash Nanjaiah; Marko Raseta; Lognathen Balacumaraswami

#### Royal Stoke Hospital, Stoke on Trent, UK

**Correspondence**: Khurum Mazhar

*Journal of Cardiothoracic Surgery 19(1):* A37

**Objectives:** Predictive risk models help with stratification of patients for cardiac surgery. However, prognostication of pre-operative variables’ impact on post-operative outcomes is poorly understood for disease specific models. We designed a risk model for predicting mortality and morbidity after isolated Surgical Aortic Valve Replacement (SAVR) for severe aortic stenosis (sAS) and compared its performance to EuroSCORE (ES).

**Methods:** Prospective data collection of patients who underwent isolated SAVR for sAS at a single institution June 2004–May 2018 (inclusive). Exclusion criteria: SAVR for severe aortic regurgitation/mixed disease, incomplete data fields, previous cardiac surgery, endocarditis or pre-operative cardiogenic shock. 12-month mortality data was obtained from the Office for National Statistics. Predictive models were developed for 5 outcomes of interest; 30 day: mortality, Stroke / Transient Ischaemic Attack (TIA), new renal failure requiring Continuous Veno-Venous Haemofiltration (CVVH), insertion of new Permanent Pacemaker and 12 month mortality. Application of the ‘rule of 10’ was used to reduce overfitting. All models were cross validated 1000 times.

**Results:** 1049 patients analysed. Median age 72 Inter-Quartile Range (64–78). Cardiopulmonary Bypass and Aortic Cross Clamp time was not associated with adverse outcome. Table 1 shows performance of ES in comparison to our calculated models. ES had good discriminatory value in all outcomes, but our model proved to be a better predictor of 12-month mortality and CVVH.
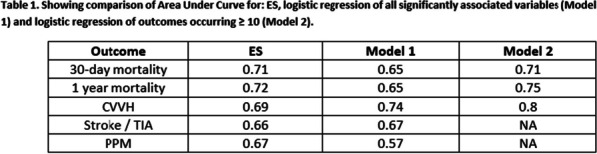


**Conclusions:** We developed a risk prediction model for SAVR in sAS using readily obtainable variables. The risk prediction model performs well outperforming ES for one year survival. A similar disease specific model for transcatheter aortic valve technology would allow clinicians and patients alike to make an informed choice about therapeutic options for isolated sAS.

## A38 Outcome predictors of surgical aortic valve replacement with concurrent coronary artery bypass grafting: insights from a large UK registry

### Daniel Fudulu^1^; Georgia Layton^2^; Bao Neyuen^3^; Shubhra Sinha^1^; Arnaldo Dimagli^1^; Gustavo Guida^1^; Gianni D Angelini^1^; Mustafa Zakkar^2^

#### ^1^Bristol Heart Institute, University of Bristol, Bristol, UK; ^2^Department of Cardiovascular Sciences, University of Leicester, Leicester, UK; ^3^Department of Cardiac Surgery, Derriford Hospital, Plymouth, UK

**Correspondence**: Daniel Fudulu

*Journal of Cardiothoracic Surgery 19(1):* A38

**Objectives:** Concomitant revascularisation of coronary artery disease at the same time as treatment for aortic valvopathy favourably impacts survival. However, combined surgery is associated with increased mortality compared to AVR or CABG in isolation. We aimed to identify patient or procedural predictors associated with increased postoperative mortality after AVR plus CABG.

**Methods:** We performed a retrospective analysis of all patients who underwent AVR with CABG between February 1996 and March 2019, using data from the National Adult Cardiac Surgery Audit obtained from the National Institute of Cardiovascular Outcomes Research (NICOR) central cardiac database. We used a generalised mixed-effects model to adjust for the relevant EuroSCORE 2 covariates and to assess the effect of the number and type of bypass grafts on clinical outcomes of AVR plus CABG.

**Results:** Fifty-one thousand two hundred seventy-two patients were included in the study. Patients receiving two or more bypass grafts demonstrated more significant disease severity and pre-operative comorbidity; they exhibited greater left main stem disease, more recent MIs, more significant LV impairment and more insulin-dependent diabetes (p < 0.001).

Patients receiving two or more bypass grafts experienced more frequent post-operative strokes, dialysis, and increased mortality (p < 0.001). There was an association between an increasing number of grafts and poorer short and long-term clinical outcomes. A single arterial conduit was associated with a reduction in mortality (p < 0.001), but this association was lost with more than one arterial conduit.

**Conclusions:** Morbidity and mortality of combined AVR and CABG increased with the number of grafts performed. A single arterial graft is protective and should be considered whenever practical. Patients with high pre-operative morbidity should be considered for complete FFR pre-operative evaluation and hybrid intervention when there is a low burden of coronary disease

## A39 Outcomes for SAVR in low-risk aortic stenosis patients in a tertiary care centre

### Salman Arif^1^; Azar Hussain^2^; Walid Elmahdy^3^

#### ^1^Castle Hill Hospital / Hull University Teaching Hospital, Hull, Cottingham, UK; ^2^Castle Hill Hospital, Cottingham, UK; ^3^Leeds General Infirmary, Leeds, UK

**Correspondence**: Salman Arif

*Journal of Cardiothoracic Surgery 19(1):* A39

**Objectives**

- TAVR has revolutionised the treatment of prohibitive/high risk symptomatic severe aortic stenosis patients.
- Nonetheless, SAVR is the standard treatment strategy in patients at low surgical risk (EuroSCORE II < 4% with no other risk factors like frailty, porcelain aorta, sequelae of chest radiation).
- Recently, PARTNER3 and Evolut trials have evaluated the outcome of TAVR in low-risk patients also.
- Our objective of this study was to benchmark the outcome of SAVR in comparison with the TAVI and review the PARTNER3 and Evolut low risk trials results in the light of the outcome of SAVR in a high-volume tertiary care centre.

**Methods**

- All high or intermediate risk, re-do patients and patients with renal failure were excluded.
- Low risk elective patients who underwent SAVR were included from 2013 to 2020.
- Retrospective analysis of patient notes was performed in two groups of patients i.e.; A < 70 and B > 70 years of age.
- The Primary outcomes were In hospital Mortality, Permanent Stroke, and Need for permanent pacemaker.

**Results**

- 944 patients met our inclusion criteria. Age ranging from 20 to 87 years. 53% were below 70 years. Mean age was 67 ± 10.
- Mean EuroSCORE II was 1.4 ± 0.8.
- In hospital mortality in group A was 0.2% and 1.1% in group B.
- Permanent stroke was 0.2% in group A and 0.9% in group B.
- 2.6% of group A patients required permanent pacemaker insertion postoperatively as compared to 3% in group B.

**Conclusions**

- We have found that in low-risk group of younger patients the outcomes of SAVR in terms of mortality, permanent stroke, and the need for permanent pacemaker insertion are much better than TAVR outcome quoted in PARTNER3 and Evolut Trials,
- Limitation of our study is that it was a retrospective study.
- Further studies are required to evaluate the outcomes of SAVR and TAVR in subgroup of patients < 70 years in the real world looking at more variables of outcome like hospital stay, need for transfusion, etc.,

## A40 Redo surgical aortic valve replacement versus valve-in-valve transcatheter aortic valve replacement for the treatment of failed aortic bioprostheses

### Bella Milan-Chhatrisha; Mohammad Yousuf Salmasi; Anan Daqa; George Asimakopoulos; Cesare Quarto; Ulrich Rosendahl; Simon Davies

#### Royal Brompton Hospital, London, UK

**Correspondence**: Bella Milan-Chhatrisha

*Journal of Cardiothoracic Surgery 19(1):* A40

**Objectives:** Studies comparing valve-in-valve transcatheter aortic valve replacement (ViV-TAVR) and redo surgical aortic valve replacement (redo-SAVR) have largely been limited to early outcomes. This study compared late survival, longitudinal remodelling (using echocardiographic parameters) and perioperative procedure-related outcomes between cohorts.

**Methods:** Institutional databases were retrospectively analysed to identify patients undergoing reintervention for failing aortic bioprostheses between January 2011-December 2020. Kaplan–Meier estimates and multivariate Cox regression were used to evaluate survival. Echocardiographic parameters were compared at stipulated post-operative timepoints using appropriate statistical tests. Procedural complications were compared using a logistic regression model. p < 0.05 was considered significant.

**Results:** Between 131 redo-SAVR and 128 ViV-TAVR patients, no significant differences were observed in early or late survival (log-rank p = 0.171); > 1 previous sternotomy was the only significant predictor of mortality (p = 0.047). Remodelling patterns were comparable, excepting left ventricular length and number of patients with paravalvular regurgitation. Redo-SAVR patients experienced significantly higher incidences of new-onset atrial fibrillation (odds ratio [OR]: 3.49 [CI: 1.43–8.49]) and pulmonary complications (OR: 6.95 [CI: 2.34–20.67]). Conversely, redo-SAVR patients experienced fewer vascular access-related complications (OR: 0.23 [CI: 0.08–0.63], p = 0.004), but less frequently underwent routine home discharge (OR: 0.20 [CI: 0.06–0.72], p = 0.013).

**Conclusions:** Comparable late survival outcomes and longitudinal remodelling patterns suggest equivalence of ViV-TAVR and redo-SAVR for high-risk surgical candidates; risk of potentially prohibitive morbidity numerators may better guide treatment selection. Paravalvular regurgitation remains an unfavourable sequela of ViV-TAVR, and clinical judgement remains prudent for determining treatment modality.

## A41 Outcome and structural valve dysfunction of bioprosthetic aortic valve replacement: Does recent literature reflect current results?

### Mahmoud Abdelaziz; Giuseppe Rescigno; Patrick Yiu; Maciej Matuszewski; Nicolas Nikolaidis; John Billing; Alina Budacan

#### Heart and Lung Centre, New Cross Hospital, Wolverhampton, UK

**Correspondence**: Alina Budacan

*Journal of Cardiothoracic Surgery 19(1):* A41

**Objectives:** Recent published data comparing surgical bioprosthetic AVR and TAVI eluded to the higher structural valve dysfunction in the surgical arms compared to TAVI. Data in the literature made use of various older generation bioprosthetic valves that are known to have higher incidence of Structural Valve Dysfunction (SVD). We present our results of Bioprosthetic AVR over an 8-year period.

**Methods:** 248 patients underwent single bioprosthetic aortic valve replacement over the study period were analysed. Survival, functional status and echo data were analysed and presented.

**Results:** Mean age was 71 (SD ± 7.8) years, 551% were males. 31 (12.5%) and 7 (2.8%) had moderate and severe LV dysfunction with 92 (37%) and 105 (42%) of patients presenting with class II and III NYHA respectively. The most common valves used were Trifecta (32%), Medtronic Hancock (22.5%), Carpentier-Edwards (CE) Perimount (12%) and CE Magna-ease (22%). Mean follow-up in months were 63 (median 59.4) with total mortality over the study period of 14%. In-hospital mortality was 0.8%, 1 year of 1%, and at 2-year mortality of 2.2%. Severe SVD occurred in 2 patients (0.84%) and moderate SVD in 38 patients (16%). Valve endocarditis rate was 4.4% and severe trans-prosthetic regurgitation was 1.6%. 9 Patients underwent re-operation (3.6%). Stroke rate was 3.6%. Death occurred in 16%, 20%, 12% and15% of Hancock, CE perimount, CE Magna-ease, and Trifecta valves implanted.

**Conclusions:** Surgical AVR has good outcomes and the rate of SVD and failure are significantly lower than that proposed by recent publications when compared with older generations of surgical valves as well as TAVI valves. Types of Bioprosthesis in comparative studies between TAVI and SAVR should be taken into account in further literature.

## A42 Does the severity of patient prosthesis mismatch have a survival impact in elderly patients after aortic valve replacement?

### Benjamin Omoregbee; Francesca Leone; Hind Elhassan; Alex Cale; Martin Jarvis; David Zicho; Mubarak Chaudhry; Mahmoud Loubani; Dumbor Ngaage

#### ^1^Castle Hill Hospital / Hull University Teaching Hospital, Hull, UK

**Correspondence**: Benjamin Omoregbee

*Journal of Cardiothoracic Surgery 19(1):* A42

**Objectives:** Patient prosthetic mismatch (PPM) is not uncommon and has an adverse effect on survival. However, in elderly patients where survival benefit is not often the indication for aortic valve replacement, the impact of PPM severity is unclear. We therefore, sought to understand the effect of PPM severity on survival in the elderly.

**Methods:** Since PPM is uncommon with aortic valve sizes ≥ 23 mm, we conducted a retrospective review of 358 patients ≥ 65 years of age, who had isolated AVR with ≤ 21 mm sized valves in our centre between January 2010 and January 2022. Using the standard definition of Effective Orifice Area Indexed (EOAI), we graded PPM as: moderate (0.65–0.85 cm^2^/m^2^) and severe (< 0.65 cm^2^/m^2^).

**Results:** 332 had estimated EOA of the implanted valves. We excluded 95 with no PPM. PPM was severe in 14.8% (n = 49) and moderate in 56.6% (n = 188). The groups clinical profiles are on Table 1. Severe PPM patients were younger, and often females. In-hospital outcomes were similar for both groups.

**Table 1:**
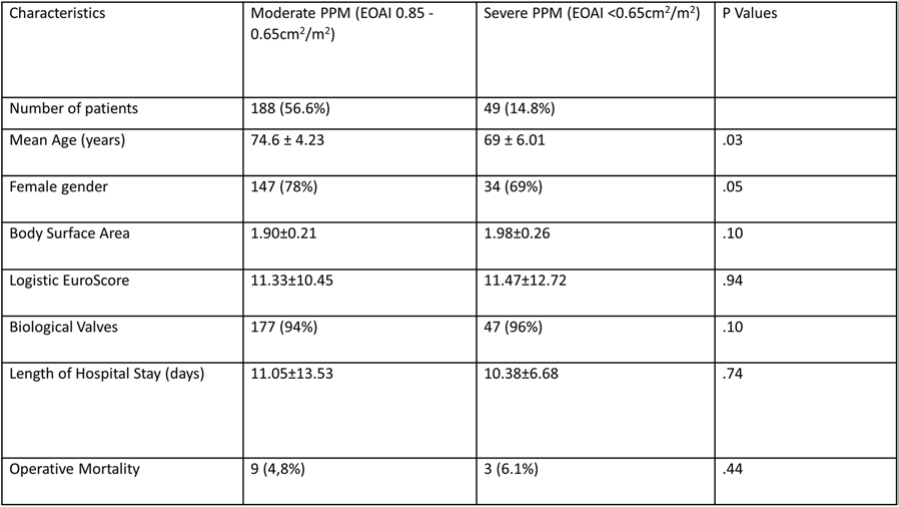
Data summary

The Kaplan Meier survival at 10 years (Severe, 43% vs. Moderate, 44%, p = 0.92) were comparable, after correcting for age.

**Conclusions:** The severity of PPM after AVR does not alter its influence on short- and long-term survival in the elderly.

## A43 Postcode health inequalities in aortic valve surgery: disparities in presentation and hospital outcomes

### Benjamin Omoregbee; Hind Elhassan; Dumbor Ngaage

#### Castle Hill Hospital / Hull University Teaching Hospital, Hull, UK

**Correspondence**: Benjamin Omoregbee

*Journal of Cardiothoracic Surgery 19(1):* A43

**Objectives:** Variations and avoidable differences in health care between different geographical regions cause health inequality. Postcode lottery in population health could have implications for the management of aortic valve disease, for which surgery is the standard of care. We examine disparities in the presentation and outcome of AVR across 3 postcode towns in North East England.

**Methods:** Clinical data for all patients who had AVR ± other procedures at our institution between February 1999 and October 2022, were reviewed. Excluding those from outside our catchment area, we grouped patients according to their postcodes towns (Table 1): Group 1 (HU), group 2 (YO), and group 3 (DN).

**Results:** Compared to other postcode towns, HU postcode town patients were more likely to present with advanced symptoms, congestive cardiac failure, significant left ventricular systolic dysfunction, active smoking, and higher predicted operative risk. They also often underwent urgent or emergency operations, and complex procedures, with a longer postoperative hospital stay.

**Conclusions:** Health inequalities resulting from postcode lottery leads to late presentation and increases operative risk and complexity of surgery for aortic valve disease. Although, we observed no significant difference in operative survival, health inequality imposes operative challenges and needs addressing to reduce operative risk and postoperative morbidity.

**Table 1:**
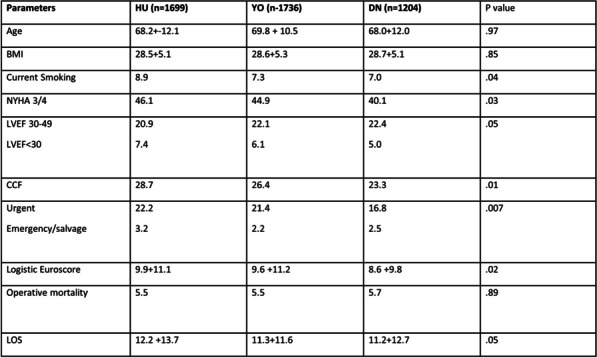
Patient characteristics

**Adult Cardiac Coronary**

## A44 Comparing CABG and PCI across the globe based on current regional registry evidence

### Tulio Caldonazo; Hristo Kirov; Leoni Riedel; Mahmoud Diab; Gloria Färber; Torsten Doenst

Department of Cardiothoracic Surgery – Jena University Hospital, Jena, Germany

**Correspondence**: Tulio Caldonazo

*Journal of Cardiothoracic Surgery 19(1):* A44

Comparing CABG and PCI across the globe based on current regional registry evidence | Scientific Reports (nature.com)

## A45 A scope of practice review: current use of vein graft preservation solutions across the UK

### Georgia Layton^1^; Shameem Ladak^1^; Liam McQueen^1^; Riccardo Abbasciano^2^; Mustafa Zakkar^1^

#### ^1^Department of Cardiovascular Sciences, University of Leicester, Leicester, UK; ^2^Department of Cardiothoracic Surgery, Imperial College Healthcare NHS Trust, Leicester, UK

**Correspondence**: Georgia Layton

*Journal of Cardiothoracic Surgery 19(1):* A45

**Objectives:** Intra-operative handling of the long-saphenous vein during and after harvesting, as well as the composition of storage and flushing solutions, impacts its endothelial integrity and function. This impacts graft patency and patient outcomes. There is no accepted standard for vein graft preservation during harvesting and there remains equipoise within the specialty as to which solutions are best. We aimed to investigate the current UK scope of practice and preferences.

**Methods:** A cross-sectional, electronic survey was distributed online to perform convenience sampling of all adult cardiac surgery professionals in the UK. A combination of open and closed questioning was used to explore current practices and beliefs regarding vein graft integrity.

**Results:** 120 responses were received from 35 (100%) UK NHS/HSC adult cardiac surgery units (median responses per unit 2). There are eight commonly used preservation fluids used during and after vein harvest. Most units performed both conventional and endoscopic vein harvest in a surgeon-dependent fashion, except for six units who do not utilise endoscopic vein harvest.

21.8% and 42% of respondents do not believe preservation fluid choice can impact vein graft integrity or postoperative outcomes. More than a third of respondents do not believe vein integrity can impact graft patency or short and long-term clinical outcomes demonstrating that current UK-wide clinical practice is significantly incongruent with newly emerging evidence in this field.

**Conclusions:** There is gross heterogeneity in practice with more than 80% of respondents reporting variation of consultant practice within their unit and only six units have a departmental protocol for the use of preservation fluids. The detrimental impact of some vein preservation solutions on graft integrity and clinical outcomes is recognised but not fully characterised, and so should be addressed through robust prospective clinical trials.

## A46 Should all CABG patients receive an arterial conduit? Defining the use of total venous revascularisation in the UK

### Georgia Layton^1^; Shubhra Sinha^2^; Riccardo Abbasciano^3^; Daniel Fudulu^2^; Gustavo Guida^2^; Gianni D Angelini^2^; Mustafa Zakkar^1^

#### ^1^Department of Cardiovascular Sciences, University of Leicester, Leicester, UK; ^2^Bristol Heart Institute, University of Bristol, Bristol, UK; ^3^Department of Cardiothoracic Surgery, Imperial College Healthcare NHS Trust, Leicester, UK

**Correspondence**: Georgia Layton

*Journal of Cardiothoracic Surgery 19(1):* A46

**Objectives:** The long-term patency of arterial grafts has long been proven resulting in the LIMA being the gold-standard choice of conduit for all patients. However, the LSV remains one of the most utilised conduits for CABG and in some circumstances, is the only suitable conduit option. We aimed to examine the outcomes of CABG in the UK over the last two decades; specifically, patients receiving only venous conduits compared to those receiving at least one arterial conduit.

**Methods:** Retrospective analysis of all consecutive patients undergoing cardiac surgery including CABG, in the UK, between February 1996 and 2018, using data from the prospectively managed NACSA and NICOR databases. We analysed the variance of the mean to compare clinical outcomes between patients receiving total venous revascularisation versus all other conduit choices.

**Results:** 459,390 patients were included in the study. Patients receiving total venous revascularisation were older and more likely to be female (p < 0.001). They demonstrated greater pre-operative symptom severity as well as more baseline morbidity and operative complexity. The data suggest that total venous conduits were preferred for patients undergoing multiple procedures or who were haemodynamically unstable.

Mortality was greater in patients receiving TVR compared to those receiving at least one arterial graft (6.9% versus 2.1%, p < 0.001) although other post-operative complications such as MI and stroke were similar between groups despite significant variations of critical pre-operative status and number of on-pump surgeries.

**Conclusions:** We have established a correlation between high-risk operative patient profiles and total venous revascularisation at time of CABG. TVR retains a critical role for certain patient groups and its utility should not be dismissed, particularly for high-risk or unstable patients where myocardial revascularisation by any safe conduit should be prioritised.

## A47 The impact of pre-operative diabetic control on short-term outcomes following coronary artery bypass

### Fadi Al-Zubaidi^1^; Manoraj Navaratnarajah^1^; Jean-Luc Duval^2^; Ravi De Silva^1^

#### ^1^Royal Papworth Hospital NHS Foundation Trust, Cambridge, UK; ^2^Oxford University Hospitals NHS Foundation Trust, Oxford, UK

**Correspondence**: Fadi Al-Zubaidi

*Journal of Cardiothoracic Surgery 19(1):* A47

**Objectives:** Examine the impact of diabetic control on short-term outcomes following coronary artery bypass.

**Methods:** We collected local data on 379 patients undergoing isolated CABG between August 2020 and October 2021. The cohort was split by blood glucose levels (BMs) in the anaesthetic room into three groups: BMs < 7 mmol/L (n = 263), BMs 7.0–11.1 mmol/L (n = 86) and BMs > 11.1 (n = 30). In a retrospective analysis we compared background characteristics, intraoperative variables and postoperative outcomes. Our primary outcome was in-hospital mortality; secondary outcomes included post-operative pneumonia (HAP), prolonged postoperative inotrope requirements, superficial wound infections and units of blood transfused. We planned multivariable regression models assessing the association between pre-operative BMs and mortality, HAP, prolonged inotropes and delayed discharge. Variables differing with p < 0.25 were included as covariates.

**Results:** Univariable comparisons demonstrated that pre-induction BMs > 11.0 mmol/L were associated with significantly higher rates of in-hospital mortality (7%, p = 0.025), post-operative prolonged inotrope requirements (20%, p = 0.002), mean units of blood transfused (2 units, p = 0.018), and rates of delayed-discharge (20%, p = 0.037). Multivariable analyses demonstrated that pre-induction BMs > 11.0 mmol/L independently predict prolonged postoperative inotrope requirements (OR: 5.02; 95% CI: 1.29–19.50, P = 0.020); pre-induction BMs were not found to independently predict mortality, post-operative HAP or delayed discharge.

**Conclusions:** Real world data demonstrate significantly worse outcomes in patients presenting with poor diabetic control. There is a strong independent association between raised pre-operative blood glucose levels and prolonged post-operative inotrope requirements. Blood glucose levels should be optimised carefully prior to surgery in order to improve outcomes.

## A48 More grafts, more risk?

### Gareth Hooks; Alastair Graham

#### Royal Victoria Hospital, Belfast, UK

**Correspondence**: Gareth Hooks

*Journal of Cardiothoracic Surgery 19(1):* A48

**Objectives:** Coronary artery bypass grafting aims to revascularise epicardial territories supplied by diseased native vessels. It has been suggested that there is a plateau effect with no further benefit beyond one graft to each of the 3 major epicardial territories, exceeding this may even prove detrimental. What constitutes complete revascularisation is debated and a higher number of bypass grafts may simply be a marker of more aggressive coronary disease. Implicit in a more ‘comprehensive’ revascularisation is an increase in complexity and operation length. This may place patients at greater perioperative risk.

**Methods:** 842 consecutive patients with TVD who underwent isolated, first time CABG were retrospectively reviewed and categorised as having either undergone:

- Conservative revascularisation—***Maximum*** of 1 bypass graft per epicardial territory (n = 416)
- Non-conservative revascularisation revascularisation—***At least*** 1 bypass graft per epicardial territory (n = 426)

Perioperative outcomes were investigated using Chi square, Fishers exact test or Independent T-test calculations as appropriate. Subgroup analysis was performed for: Patients ≤ 74 years, ≥ 75 years and impaired LVSF (EF < 50%).

Peri-operative outcome.

**Results**:

**Table:** 

Peri-operative Outcome	Conservative Revascularisation	Non-Conservative Revascularisation	P Value
Average Length Of Cross-clamp	59.8 (SD = 18.50)	74.3(SD = 19.40)	< 0.001
Average Postoperative Length Of Sta	10.2 (SD = 8.93)	10.2 (SD = 7.14)	0.970
Required Convalescence Prior To Discharge Home	8.6% (n = 35)	9.5% (n = 40)	0.643
In Hospital Mortality	1.9% (n = 8)	1.1% (n = 5)	0.378
New Post-Operative IABP Requirement	5.3% (n = 21)	4.5% (n = 18)	0.628
Newly Dialysis Dependent	1.2% (n = 5)	1.6% (n = 7)	0.589
Newly Diagnosed Postoperative CVA	0.5% (n = 2)	0.5% (n = 2)	0.981
75 years: New Post-Operative IABP Requirement	14.3% (n = 10)	4.3% (n = 4)	0.024
75 years: Newly Dialysis Dependent	0% (n = 0)	5.4% (n = 5)	0.071

**Discussion:** No significant difference in perioperative outcomes was identified when the 2 revascularisation strategies were directly compared. Subgroup analysis demonstrated no significant difference in patients ≤ 74 years or patients with impaired LVSF.

Interestingly there was a significant difference in the requirement for IABP in conservatively revascularised patients aged ≥ 75 with the potential implication of incomplete revascularisation, but an increase in new dialysis dependency with the non-conservative revascularisation group which approached significance.

What constitutes complete coronary revascularisation remains disputed. This study suggests pursuing a non-conservative revascularisation strategy does not adversely affect perioperative outcomes of patients ≤ 75 years.

## A49 Left internal thoracic artery versus saphenous vein grafting to the left anterior descending artery after isolated coronary artery bypass surgery

### Hannah Masraf^1^; Mostafa Mohamed^2^; Anna Zingale^2^; Davorin Sef^2^; Szabolcs Miskolczi^2^; Theodore Velissaris^2^; Suvitesh Luthra^2^

#### ^1^Kingston Hospital, London, UK; ^2^University Hospital Southampton, Southampton, UK

**Correspondence**: Hannah Masraf

*Journal of Cardiothoracic Surgery 19(1):* A49

**Objectives:** Left internal thoracic artery (LITA) grafting to the left anterior descending artery (LAD) is the gold standard in coronary artery bypass surgery (CABG). This study assesses peri-operative and long-term outcomes of LITA versus saphenous vein graft (SVG) to the LAD for primary isolated CABG.

**Methods:** We reviewed 8237 patients (1602 SVG-LAD/6725 LITA-LAD) with isolated primary CABG between 2000 and 2020. Propensity score matching (PSM) was performed for LITA vs SVG to the LAD with 1:1 matching (nearest neighbour). Survival was compared with Kaplan–Meier and Cox proportional hazards methods.

**Results:** 1,270 pairs were propensity matched to SVG versus LITA to LAD. Survival was worse for SVG-LAD in the unmatched group (SVG-LAD; 12.9 ± 0.60 years versus LITA-LAD; 16.5 ± 0.26 years; p < 0.001, Fig. 1A) but was comparable in the propensity matched group (SVG-LAD; 13.0 ± 0.60 years versus LITA-LAD; 13.7 ± 0.47 years; p = 0.35 Fig. 1B). Age, gender, renal dysfunction, diabetes, hypertension, pulmonary disease, cross-clamp time, triple vessel disease, hemofiltration and logistic EuroSCORE were independent predictors of adverse long-term survival. SVG-LAD did not adversely impact long-term survival (HR 0.90 (95% CI: 0.88–1.16 p = 0.91). There was no difference in survival between propensity matched groups when stratified by age (< 70 yrs versus ≥ 70 years); logistic EuroSCORE (< 10 versus ≥ 10) or diabetes.

**Fig. 1:**
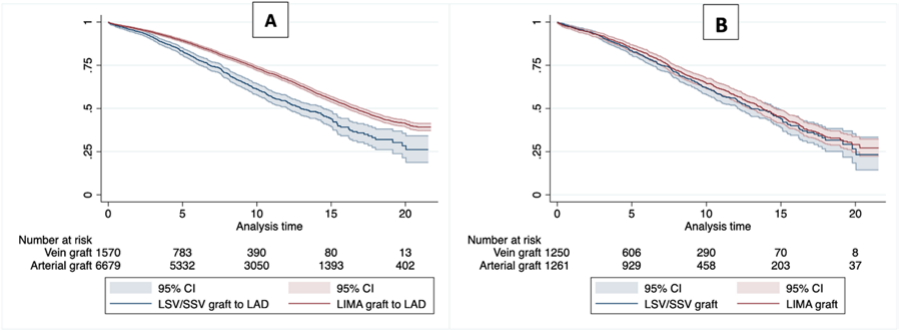
**A** Unmatched survival between SVG-LAD vs LITA-LAD. **B** Survival in propensity matched groups SVG vs LITA

**Conclusions:** Appropriate use SVG-LAD in selected patients may offer equivalent survival to LITA-LAD.

## A50 Assessment of the use of Bart's surgical infection risk (BSIR) tool in clinical practice

### Charlotte Christian^1^; Rosalie Magboo^1^; Jackie Cooper^2^; Bev Blair^1^; Angelica Tambal^1^; Alex Shipolini^1^; Heather Byers^1^; Julie Sanders^2^

#### ^1^Barts Health NHS Trust, London, UK; ^2^Queen Mary University London, London, UK

**Correspondence**: Charlotte Christian

*Journal of Cardiothoracic Surgery 19(1):* A50

**Objective:** The Barts Surgical Infection Risk (BSIR) tool is a pre-operative risk assessment tool to predict surgical site infection. It was locally developed and internally validated (1). We sought to audit the efficacy of the BSIR tool in identifying patients who are at risk of developing SSI after cardiac surgery.

**Methods:** A prospective audit of cardiac surgical patients over a 6-week period in a single center was conducted. The BSIR score for each patient was calculated and stratified according to low, moderate or high-risk categories by the clinical team as part of routine practice. An estimated risk for each category was determined using the SSI risk estimate from the developmental study (1) and compared with the actual risk based on the number of SSI’s in the audit conducted. Chi-square test was performed to assess the difference between the observed and expected frequencies of patients with and without SSI from the audit.

**Results:** Over the audit period 180 patient underwent cardiac surgery. All had a clinically obtained BSIR score. Of these, 50% were scored as low—39% as moderate—and 11% as high-risk for developing SSI. These proportions did not significantly differ with the percentage of patients for each category from the pilot study (44%, 41% and 15%) (p = 0.18). Four (2.2%) patients developed SSI. From this, the expected numbers with SSI were calculated at 1.5, 2.7 and 1.7 for low, moderate and high-risk. These numbers were not significantly different with the observed numbers with SSI (p = 0.89).

**Conclusions:** This audit found that the stratification scoring of the BSIR tool correctly identifies patients who are at risk of developing SSI. Further research will be needed to recalibrate BSIR to account for the reduction of SSI rates due to targeted interventions.

Magboo R, Drey N, Cooper J, Shipolini A, Byers H and Sanders J (2020) Predicting SSI: Development and Validation of B-SIR Tool. *J Clin Epidemiology*; 128: 57–65.

## A51 Aprotinin in cardiac surgery: safety and efficacy in isolated CABG

### Rishab Makam; Mahmoud Loubani

#### Castle Hill Hospital, Hull, UK

**Correspondence**: Rishab Makam

*Journal of Cardiothoracic Surgery 19(1):* A51

**Objectives:** Aprotinin was reported to increase morbidity and mortality in some nonrandomized observational studies. It was usually administered to patients who are high risk which was not adequately considered in the evidence. Aprotinin was reintroduced for isolated CABG in 2019 at our department. Our study reports our centre’s data on the use and evaluates the outcomes related to the safety of Aprotinin in adult patients.

**Methods:** We prospectively collected data relating to all cases using aprotinin since its approval in 2019 and compiled into a data base. We gathered all case data including patient demographics, outcomes, and complications for analysis.

**Results:** Aprotinin was used in 67 cases since 2019 which represents 3.16% of total patients, 4.48% were elective, 61.19% urgent, 28.36% emergency and 5.97% salvage. 74.63% of cases were Isolated CABG (iCABG), only 4% of which were elective. In-hospital mortality in iCABG patients was 12% with average EUROSCORE II of 14.4 ± 16.67. The use in our department contrasts the data from the Nordic Aprotinin patient registry (NAPAR) where only 34.53% were iCABG cases, 58.6% of which were elective cases and a significantly lower EuroSCORE, 4.6 ± 6.3, and in-hospital mortality of 1.3%.

Post operative stroke occurred in two (4%) patients while three (6%) had new renal impairment that required dialysis or filtration and four patients (8%) underwent re-exploration for bleeding or tamponade. Average preoperative stay was 3.94 ± 4.05 days and post-operative ITU stay was 2.70 ± 3.67 days. Furthermore, 28% of iCABG patients required transfusion iCABG with the average patient receiving 731.29 mL (2.09 units).

**Conclusions:** We demonstrate the safe use of Aprotinin in a high-risk group of patients with shortening of preoperative stay in iCABG patients and reduction of blood products usage. Although Aprotinin is only licenced for a limited number of indications, our data shows that it still forms a vital part of the cardiac surgeon’s arsenal.

## A52 Unplanned lima to diagonal grafting in the context of non-graftable lad; an observational outcome analysis

### Karishma Chandarana; Mayooran Nithiananthan; Niki Nicou; Anas Boulemden; Adam Szafranek; Saqib Qureshi

#### Department of Cardiac Surgery, Nottingham City Hospital, Nottingham, UK

**Correspondence**: Karishma Chandarana

*Journal of Cardiothoracic Surgery 19(1):* A52

**Objectives:** Surgeons often face challenging situations when finding planned LIMA-LAD is not feasible due to several reasons. We explored the consequences of this scenario on perioperative and long-term outcomes.

**Methods:** This was a retrospective exploratory analysis of a surgical database plus patients’ operative records.

**Results:** More than 4,500 patients underwent isolated CABG between 1999 and 2021 with LIMA-LAD anastomosis carried out in 99% of patients. We identified 51 patients (1%) where LAD was deemed not graftable. Median age was 70 (range 47–84). Twelve (23%) had poor LV with n = 10 (20%) having sustained an MI within 30 days. Five patients (10%) were on pre-operative intravenous nitrates and eleven (22%) required a pre-operative IABP. Median logistic EuroSCORE was 3.5 (0.88–65). All patients had triple vessel disease with or without left main stem involvement (25%). The reasons for not grafting the LAD were; diffuse calcified disease n = 14 (27%), small vessel n = 19 (37%), intramyocardial vessel n = 13 (26%), extensive stenting n = 1 (2%) and other reasons n = 4 (8%). In all cases revascularization was undertaken to three major territories. Postoperatively, three patients (6%) were re-explored for bleeding, two (4%) required new renal dialysis, no patients had a stroke, four (8%) died before discharge, whilst forty-seven (92%) are still alive, median survival 1,872 days (range 402–3938). Postoperatively, three (6%) patients re-presented with NSTEMI, two (4%) required PCI and four (8%) required hospitalisation for heart failure.

**Conclusions:** Inability to graft LAD at the time of surgical revascularization is certainly inferior but still portends a favourable perioperative and longer-term outcome, as long as the anterior territory is revascularized using a suitable diagonal artery (Fig. 1).

**Fig. 1:**
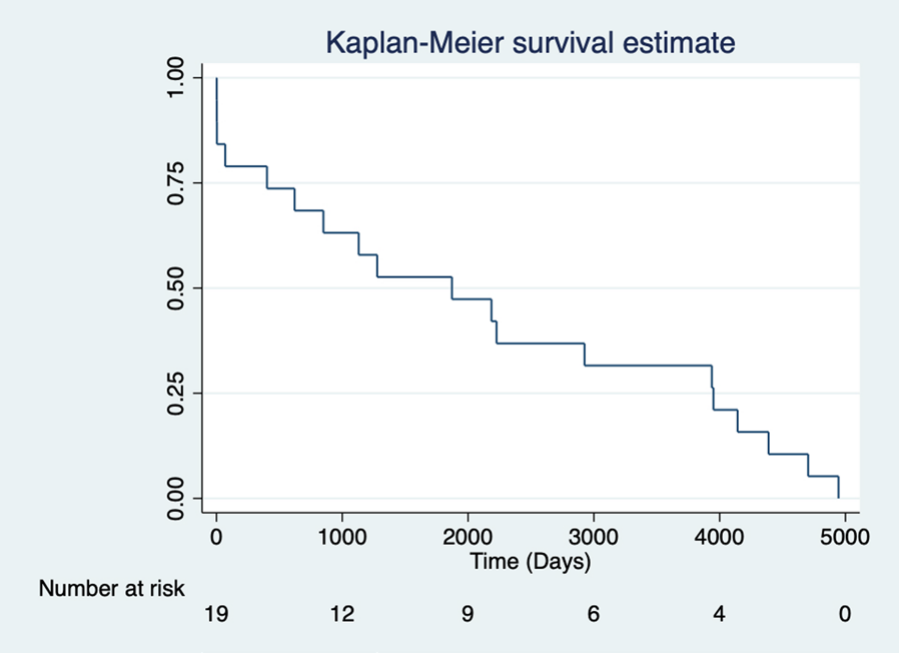
Kaplan–Meier survival curve for LIMA-Diagonal graft recipients

## A53 Surgical repair of post-infarction ventricular septal defects: a 10-year, single-centre experience

### Mohamed Sherif; Hamza Rehman; Betsy Evans

#### Leeds General Infirmary, Leeds, UK

**Correspondence**: Hamza Rehman

*Journal of Cardiothoracic Surgery 19(1):* A53

**Objective:** To evaluate short and long-term outcomes for surgical repair of the post-infarction ventricular septal defect. This study evaluates a 10-year, single-centre experience.

**Methods:** Sixteen patients underwent surgical repair of an ischaemic VSD in Leeds General Infirmary between 2010 and 2021. Pre- and post-operative data, including long-term follow-up, were collected from these patients' electronic health records and analysed using IBM SPSS Statistics 27.

**Results:** The mean patient age was 64 (range 46–77). 69% (11 patients) were male, and 31% (5 patients) were female. 56% of cases were posterior VSDs, and 43% were anterior VSDs. In over half (56%) of patients, an intra-aortic balloon pump was inserted perioperatively.

The mean time from referral to surgery was 2.8 days (range 0–18 days). 50% of patients only had patch repair, while the other half had a patch repair along with coronary artery bypass grafting (CABG).

In-hospital mortality post-ischaemic VSD repair was 37.5% (6 patients). VSD repair with the patch alone was associated with a statistically significant (p < 0.001) higher mortality than patch repair and concurrent CABG, with mortality rates of 62.5% and 12.5%, respectively. Five-year survival was only 44% (7 patients); most patients (71%, five patients) had a patch repair with CABG.

In univariant and multivariant analysis, the type of operation (patch repair with CABG) and early surgery were associated with higher in-hospital and long-term survival.

**Conclusions:** The surgical repair of post-infarct VSD is associated with high in-hospital and long-term mortality. Performing CABG alongside VSD closure could be associated with a relatively favourable outcome. Our experience has echoed findings already known in the literature. A more extensive study with a larger cohort of patients is needed to analyse further factors associated with short and long-term survivability post-ischaemic VSDs.

## A54 Coronary surgery for patients with poor left ventricular function: 12-year real-world data

### Giuditta Coppola; Fabio De Robertis; Nandor Marczin; Sunil Bhudia; Shahzad Raja

#### Harefield Hospital, London, UK

**Correspondence**: Giuditta Coppola

*Journal of Cardiothoracic Surgery 19(1):* A54

**Objectives:** Coronary artery bypass graft surgery (CABG) is still the gold standard for patients with complex multiple-vessel disease. Patients with poor left ventricular ejection fraction (LVEF) who require CABG remain a big challenge in cardiac surgery. We analysed the short-term outcomes and long-term mortality in off-pump (OPCAB) and on-pump (ONCABG) CABG with poor LVEF.

**Methods:** We retrospectively analysed prospectively collected data from institutional database from January 2007 to December 2019. During the study period 381 patients with poor LVEF underwent a CABG procedure.

**Results:** Mean age of study population was 65.79 years (± 10.12) and 341 patients (89.5%) were male. OPCAB was performed in 160 (42%) patients. Fewer patients in OPCAB group required postoperative IABP (3.1% vs 11.3%; p = 0.01) and in-hospital stay was significantly shorter in this group (15.51 vs. 21.06 days, p = 0.01). All other outcomes including in-hospital mortality were similar for the two cohorts.

**Conclusions:** CABG in poor LVEF is still a major challenge. No differences in terms of major post-operative outcomes have been found between the OPCAB and ONCABG procedures. However, the decreased risk of post-IABP insertion and length of in-hospital stay support OPCAB as the preferred surgical revascularisation strategy for this high-risk cohort.

*Table: Baseline characteristics of the patients and postoperative results.*

*CVA* = *cerebrovascular accident*

*IABP = intra-aortic balloon pump*

*CABG = Coronary artery bypass grafting*

The patients were divided in two groups: Off pump CABG (OPCAB, 42%) and On pump CABG (ONCABG, 58%). The baseline characteristics of the patients have been evaluated. The age has been found significantly higher in the OPCAB group compared to the ONCABG one. The other characteristic were similar between the groups. Fewer patients in the Off Pump group required postoperative IABP (3.1% vs 11.3%; p = 0.01) and in-hospital stay was significantly shorter in this group (15.51 vs. 21.06 days, p = 0.01). All other outcomes including in-hospital mortality were similar for the two cohorts.

**Table:** 

	Off pump CABG 160 pts (42%)	On pump CABG 221 pts (58%)	P-value
Age, mean (± SD)	67.15 (± 10.75)	64.80 (± 9.55)	**0.01**
Male, n (%)	142 (88.8%)	199 (90%)	0.81
Diabetes, n (%)	69 (43.1%)	94 (42.5%)	0.99
Hypertension, n (%)	112 (70%)	135 (61.1%)	0.09
Renal impairment, n (%) (GFR < 60 mL/min)	48 (30%)	72 (32.6%)	0.67
Previous CVA, n (%)	16 (10%)	17 (7.7%)	0.54
Preoperative Inotropes, n (%)	8 (5%)	17 (7.7%)	0.40
Previous MI, n (%)	116 (72.5%)	174 (78.7%)	0.19
Preoperative IABP, n (%)	27 (16.9%)	30 (13.6%)	0.45
Euroscore, mean (± SD)	7.63 (± 3.51%)	7.16 (± 3.76%)	0.15
Urgency, n (%)	79 (49.4%)	132 (59.7%)	0.06
Postoperative renal failure, n (%) (GFR < 60 mL/min)	8 (5%)	18 (8.1%)	0.31
Tracheostomy, n (%)	4 (2.5%)	14 (6.3%)	0.13
Postoperative IABP, n (%)	5 (3.1%)	25 (11.3%)	**0.01**
Mean Hospital Stay (days), mean (± SD)	15.51 (± 14.71)	21.06 (± 17.68)	**0.01**
In-hospital mortality, n (%)	12 (7.5%)	17 (7.7%)	1
Follow-up mortality, n (%)	53 (33.1%)	77 (34.8%)	0.93

## A55 Evaluation of saphenous vein graft patency using wall shear stress and geometry post-coronary artery bypass grafting surgery: a literature review

### Hashem Abdel-Kader^1^; Panagiotis G. Kyriazis^2^; Prakash P. Punjabi^2^

#### ^1^Peninsula Medical School, Plymouth, UK; ^2^Imperial College Healthcare NHS Trust, Hammersmith Hospital, Department of Cardiothoracic Surgery, London, UK

**Correspondence**: Hashem Abdel-Kader

*Journal of Cardiothoracic Surgery 19(1):* A55

**Objectives:** Although saphenous vein graft (SVG) patency is inferior to arterial alternatives, SVGs remain the most frequently chosen conduits in coronary artery bypass grafting (CABG). Current literature attributes this inferiority to the effects of wall shear stress (WSS) and geometry on intimal hyperplasia and atherosclerosis. Computational fluid dynamics (CFD) suggest a potential objective method of evaluating the effects of these metrics on graft patency. This review discusses the effects of WSS and geometry on graft remodelling and explores the feasibility of CFD as an evaluation tool of SVG patency.

**Methods:** PubMed, Cochrane Library and EMBASE databases were used to search the terms "wall shear stress", "geometry", "computational fluid dynamics" and "saphenous vein graft patency". Out of 374 papers, 82 were selected based on experimental exploration of WSS and/or geometry in relation to SVG patency in patients with coronary vessel disease. Studies were also excluded due to isolated qualitative domains used to assess graft patency or if they were solely based on animal models, reducing reviewed studies to 53.

**Results:** Low WSS and/or a turbulent WSS environment is associated with excessive intimal hyperplasia, atherosclerosis and ultimately graft failure. The geometrical features of a SVG influencing its patency include larger SVG diameters, a target coronary diameter > 2.0 mm, graft wall thickness > 1.5 mm, SVGs with significant curvature at the graft-host junction and anastomotic angles > 15–20 degrees. The functional assessment of SVG patency using CFD is effective and provides results coinciding with literature.

**Discussion:** The pathophysiology of SVG failure correlated with WSS and geometry is well established in the literature. CFD provide a potential method of functionally assessing grafts by analysing their geometry and flow patterns. With further enhancements, CFD can provide great clinical utility for surgeons and significantly improve CABG patient outcomes.

## A56 Coronary artery imaging in the pre-operative assessment of patients undergoing cardiac surgery

### Eduardo Urgesi^1^; Ciro Amodio^1^; Mai Shehab^2^; Nada Al Yasen^3^; Marsioleda Kemberi^3^; Wael Ibrahim Awad^4^

#### ^1^Barts Heart Centre. St Bartholomew's Hospital, London, UK; ^2^Queen Mary University of London; King's College London Medical School, London, UK; ^3^Barts and The London, University of London; Barts Heart Centre. St Bartholomew's Hospital, London, UK; ^4^Barts Heart Centre. St Bartholomew's Hospital; William Harvey Research Institute, QMUL, London, UK

**Correspondence**: Eduardo Urgesi

*Journal of Cardiothoracic Surgery 19(1):* A56

**Objective:** To investigate the utilisation and selection of coronary imaging modality in the pre-operative assessment of patients undergoing cardiac surgery.

**Methods:** All patients undergoing cardiac surgery at our Institute between Sept 2021 and Apr 2022 were classified into four groups according to the pre-operative coronary imaging modality performed: CT coronary angiography (CTCA) only, invasive coronary angiography (ICA) only, Both (CTCA and ICA) and None. Chi-squared and Mann–Whitney U tests were used to compare characteristics between the CTCA and ICA only groups.

**Results:** Of the 969 patients included in our study, 201 (20.7%) underwent CTCA only, 625 (64.5%) ICA only, 101 (10.4%) Both and 42 (4.3%) None. In the Both group, 81 (80.2%) had CTCA first and 20 (19.8%) patients had ICA first, due to the inability to clearly assess coronary artery disease severity on first investigation. Patients in the CTCA group vs ICA group are younger (< 60 years: 106 (52.7%) vs. 183 (29.3%)), have no history of angina (144 (71.6%) vs. 139 (22.2%)), have LVEF > 50% (171 (85.1%) vs. 427 (68.3%)), undergone previous cardiac surgery (32 (15.9%) vs. 12 (1.9%)), have higher EuroSCORE II (2.22 vs. 1.54), pre-operative infective endocarditis (25 (12.4%) vs 7 (1.1%)) and considered for major aortic procedures (64 (31.8%) vs. 19 (3%); p < 0.001 for all comparisons). Patients in the ICA group were more likely to have CCS class II, III or IV angina, (435 (69.6%) vs 44 (21.9%), extracardiac arteriopathy (25 (4%) vs. 2 (1%), p: 0.037), previous MI (335 (53.6%) vs. 3 (1.5%)) and undergoing urgent (317 (50.7%) vs. 49 (24.4%) or planned isolated CABG procedures (p < 0.001 for all comparisons bar extracardiac arteriopathy).

**Conclusions:** Our results illustrate the utilisation of CTCA and ICA in the pre-operative assessment of coronary anatomy in patients undergoing all types of cardiac surgery and may suggest a greater role for non-invasive CTCA in future, provided there are no negative consequences on outcomes.

## A57 LIMA in octogenarians: Is it best medical practice?

### Ahmed Mohamed Abdel Shafi; Manoraj Navaratnaraj; Muhammad Umar Rafiq

#### Royal Papworth Hospital, Cambridge, UK

**Correspondence**: Ahmed Mohamed Abdel Shafi

*Journal of Cardiothoracic Surgery 19(1):* A57

**Objectives:** There are a number of challenges facing cardiac surgery as a result of an ageing population. Elderly patients presenting for consideration of surgery often have multiple co-morbidities, increased severity of disease, and reduced physiological reserve.

The use of the internal mammary artery conduit for grafting in CABG has been the preferred choice with studies showing long term prognostic value. However, the question arises, is the prognostic valve seen by utilising LIMA in the historical study cohorts translated into elderly patients.

We aimed to assess the long-term survival of Octogenarians at out institution undergoing isolated CABG with and without the use of the left internal mammary artery.

**Methods:** We performed a retrospective cohort study, in which all patients undergoing isolated coronary artery bypass surgery at our Centre from January 2010 to July 2022 age 80 or over where identified.

Variables and survival outcomes were examined.

**Results:** A total of 965 patients over the age of 80 underwent isolated CABG at our institution, of which 631 (65.4%) had a LIMA graft compared to 334 (34.6%) who only had vein grafts. The median age of the two groups was 82 ± 2 years. The median EuroSCORE was 7 ± 2.37 for the LIMA group compared to 8 ± 3.23 for the no LIMA group. Elective operations were performed in 346 (54.8%) of the LIMA group compared to 119 (35.6%) in the no LIMA group, p < 0.0001. Survival at hospital discharge was 611 (96.8%) in the LIMA group and 321 (96.1%) in the no LIMA group, p = 0.5568. Long-term survival with a median follow-up period of 7.4 ± 3.2 years, for the LIMA group was 292 (46.3%) compared to 206 (61.6%) in the no LIMA group, p < 0.0001.

**Conclusions:** Our results show that the prognostic value of the LIMA conduit in CABG does not translate to longer term survival outcomes. These results raise an interesting point in terms of surgical practice and requires further research.

## A58 Female gender is not a predictor of in-hospital mortality after off-pump coronary artery bypass grafting: an analysis of 5900 cases

### Joy Edlin; Fabio de Robertis; Toufan Bahrami; Nandor Marczin; Sunil Bhudia; Shahzad Raja

#### Harefield Hospital, Harefield, UK

**Correspondence**: Joy Edlin

*Journal of Cardiothoracic Surgery 19(1):* A58

**Objectives:** Female gender is independently associated with a worse prognosis in the postoperative period after coronary artery bypass grafting (CABG). This aspect is acknowledged by the many risk stratification models, including the most commonly used European System for Cardiac Operative Risk Evaluation (EuroSCORE). We evaluated our institutional database to determine the impact of female gender on in-hospital mortality and early complications after off-pump coronary artery bypass grafting.

**Methods:** From January 2007 to December 2019, a total of 5900 patients underwent off-pump CABG. Their data were prospectively entered into the cardiac surgery database (Patients Analysis and Tracking System; Dendrite Clinical Systems, Ltd, Oxford, England, United Kingdom) and analysed retrospectively. Outcome measures included in-hospital mortality, major complications, and length of stay. Multivariate analysis was performed to identify predictors of in-hospital mortality after off-pump CABG.

**Results:** The study cohort was divided into two groups: female (n = 1085; 18.4%) and male (n = 4815; 81.6%). Females were older (68.1 years vs. 64.8 years; p = 0.003), suffered more from diabetes (41.0% vs. 31.7%; p = 0.02) and had more renal dysfunction (40.9% vs. 28.3%; p = 0.001). There was no statistically significant difference in in-hospital mortality (2.0% vs. 1.2%; p = 0.69), major complications, or length of stay (11.3 vs. 10.2; p = 0.07) between the two groups. Female gender did not emerge as an independent predictor of in-hospital mortality in the multiple logistic regression analysis (OR = 0.6; 95%, CI 0.4–0.9).

**Conclusions:** Females have comparable in-hospital mortality and morbidity to males following off-pump CABG. Female gender is not an independent risk factor for in-hospital mortality after off-pump CABG. Further larger studies are required to validate the findings of this study.

## A59 Intermittent cross clamp fibrillation vs cardioplegia for isolated coronary artery surgery: a propensity matched analysis of in-patient outcomes

### Bazegha Qamar^1^; Khurum Mazhar^1^; Ali Mohamed^1^; Mohsin Uzzaman^1^; Marko Raseta^2^; Qamar Abid^1^

#### ^1^Royal Stoke Hospital, Stoke on Trent, UK; ^2^Erasmus MC Dept. of Molecular Genetics, Rotterdam, Netherlands

**Correspondence**: Bazegha Qamar

*Journal of Cardiothoracic Surgery 19(1):* A59

**Objectives:** There are varying practices of myocardial protection during coronary artery surgery depending upon a surgeon’s preference, experience, and beliefs. We sought to investigate differences in short term outcomes between Intermittent cross clamp fibrillation (XCF) and the cardioplegia (CP) technique.

**Methods:** Retrospective observational study of all patients who underwent isolated coronary artery bypass graft surgery (CABG) between April 2003 and Oct 2022 at a single tertiary referral centre. This identified 8,399 patients who either had XCF (n = 2149) or CP (n = 6250) technique. One-to-one propensity score matching based on pre-operative variables was performed to compare 30-day: mortality, re-sternotomy for thoracic bleeding, new renal failure requiring Continuous Veno-Venous Haemofiltration (CVVH), Stroke or Transient Ischaemic Attack (TIA), and Post Operative Inotropic requirements (POI).

**Results:** There were 2015 matched pairs with no significant difference between baseline characteristics. Mean age was 65.8 (XCF) and 65.9 (CP) with a median number of three distal anastomosis in each group. There were no significant differences in 30-day mortality (0.7% vs. 0.7%, p = 1.0), re-sternotomy for bleeding (3.7% vs. 4.7%, p = 0.12), CVVH (0.9% vs. 0.8%, p = 0.86) and stroke/TIA (1.1% vs. 0.7%, p = 0.24) for XCF and CP, respectively. XCF showed a reduction in POI requirements (17.6% vs. 28.2%, p < 0.01).

**Conclusions:** XCF produces a reduction in POI requirements and maybe a useful technique for the ventricle with poor ejection fraction. Concerns about increased cerebrovascular events due to repetitive aortic cross clamping / manipulation have not been borne out in this study.

## A60 A novel advanced grading tool assessing the quality of the saphenous vein, used in coronary artery bypass grafting surgery: a QI project

### Antonia Gerontati; Benjamin Adams

#### Barts Heart Centre / St Bartholomew's Hospital, London, UK

**Correspondence**: Antonia Gerontati

*Journal of Cardiothoracic Surgery 19(1):* A60

**Objectives: **The way that saphenous veins (SV) conduits’ quality is currently described in literature is as good, moderate or poor, lacking accuracy and specificity. Endoscopic and open vein harvesting are often compared as harvesting techniques in regards to saphenous vein graft failure (SVGF), without thoroughly evaluating the state of the SV at the time of surgery, which cannot lead to safe conclusions. The intrinsic characteristics of the SV and harvesting mishaps can affect grafts patency, yet there is no tool to date grading the vein’ quality. This gap in literature has been identified and initiated a QI project. We created a novel tool (AG score) for assessing vein’ quality intraoperatively, that allows a retrospective evaluation and quality benchmarking.

**Methods:** A clinical audit evaluated the graft failure rates in our Trust and showed that no definite conclusions can be drawn on how to reduce SVGF rates, unless the quality of the SV can be analysed as well. A thorough literature review revealed seven elements that can contribute to vein’s quality and are the body of the AG score. A quasi experimental design, with two clinical audits allowed a comparison before and after tool’s implementation. Bespoke surveys evaluated the users’ satisfaction.

**Results:** Documentation rates of SV quality in operation notes increased from 57 to 100%. Bespoke surveys revealed 100% Surgeons’ satisfaction with the change. The AG score is part of the operation notes and highlights which problems of the SV were present, if any and a numerical result accordingly.

**Discussion:** This tool allowed fast review of intraoperative findings, translated qualitative classification to quantitative and created a new language when referring to SV quality. In the event of SVGF, the AG score can be insightful if used in combination with the patient's comorbidities and surgical findings. Utilising this tool at a greater scale, can produce higher quality research in the field of SVGF.

## A61 Ten years outcome of SVG to LAD: a single centre study

### Azar Hussain^1^; Salman Arif^1^; Walid Elmahdy^2^

#### ^1^Castle Hill Hospital, Cottingham, UK; ^2^Leeds General Infirmary, Leeds, UK

**Correspondence**: Salman Arif

*Journal of Cardiothoracic Surgery 19(1):* A61

**Objectives:** Left internal mammary artery (LIMA) is the conduit of choice to revascularize left anterior descending artery (LAD) due to its proven long-term patency and survival benefits.

- However other conduit materials like long saphenous vein (LSV) need to be consider for patients where LIMA cannot be used.
- Little evidence exists about long-term survival after the use of Saphenous Vein graft (SVG) for LAD anastomosis.
- The purpose of this study is to evaluate the survival for those patients who received SVG instead of LIMA to revascularize the LAD.

**Methods**

1. Patients who underwent isolated CABG with SVG to LAD, between January 2011 and March 2021 were identified from retrospective analysis of a tertiary care centre database.
2. Patients who had other concomitant cardiac procedure, previous cardiac surgery, cardiogenic shock, and those with chronic renal failure on dialysis were excluded from the study.
3. The primary outcome was all cause mortality, and the patients were divided into < 70 and > 70 years age groups.
4. A P value < 0.05 was considered statistically significant.

**Results**

- 368 patients were included.
- The mean age of cohort was 68.97 ± 9.55 years.
- The 30 days mortality was 0.68% (two patients) and the 90 days mortality was 1.03% (three patients).
- The 10-year all-cause mortality rate by Kaplan Meier Analysis was 21.8% and the mean duration of survival was 6.92 ± 0.15 years.
- Pre-op neurological dysfunction, diabetes mellitus and poor LV function resulted in worsened 10-year overall mortality.

**Conclusions**

- The study confirmed that the survival in our patients who received SVG to LAD was 78% at 10 years post-surgery.
- Although the strategy of LIMA–LAD grafting is the gold standard, these findings may provide an alternative strategy where in situ LIMA cannot be used to bypass LAD.
- Several limitations of our study should be recognized including its descriptive nature, using a relatively small cohort of patients at a single institution and the outcome is limited to survival only.

## A62 To wait or not to wait? Impact of timing of M.I. on postoperative outcomes in patients with reduced LVEF after CABG: a propensity score analysis

### Ettorino Di Tommaso^1^; Vito Domenico Bruno^2^; Lauren Kari Dixon^3^; Anil Sankanahalli Annaiah^4^; Gustavo Guida^4^; Roberto Natali^4^; Raimondo Ascione^5^

#### ^1^Bristol Heart Institute, University Hospitals of Bristol and Weston NHS Foundation Trust, UK; ^2^Bristol Medical School – Translational Health Science, Bristol, UK; ^3^Bristol Medical School – Public Health Science, Bristol, UK; ^4^Bristol Heart Institute, Bristol, UK; ^5^Bristol Medical School – Translational Health Science, Bristol, UK

**Correspondence**: Ettorino Di Tommaso

*Journal of Cardiothoracic Surgery 19(1):* A62

**Objectives:** We aim to evaluate the impact of Myocardial Infarction (MI) timing on short- and long-term outcomes after isolated coronary artery bypass grafting (CABG) in patients with reduced Left Ventricular Ejection Fraction (LVEF).

**Methods:** This is a retrospective single-centre analysis of 3026 patients with reduced LVEF (< 50%) and previous records of myocardial infarction undergoing isolated CABG over a period of 11 years. The primary outcomes were short- and long-term mortality rates. We used propensity score (PS) analysis to compare Acute (≤ 24 h), Subacute (1–30 days) and Chronic (> 30 days) MI.

**Results:** Before PS there were 59 patients in the acute group, 1,021 patients in the subacute and 1946 in the chronic group. Mortality was higher in the acute group (22%) followed by the subacute (4.1%) and the chronic group (2.8%, p < 0.01). These differences were confirmed after PS (22% vs. 4.3% vs 0%, p < 0.01). Mortality rates were not significantly different between subacute and chronic groups. There were no other differences in post-operative complications except a prolonged length of stay in the acute group (11.93 ± 10.07 vs. 10.11 ± 7.74 vs. 8.23 ± 6.86, p = 0.04). Acute MI had the worst long-term survival (Fig. 1), although this did not reach significance (p = 0.06).

**Fig. 1:**
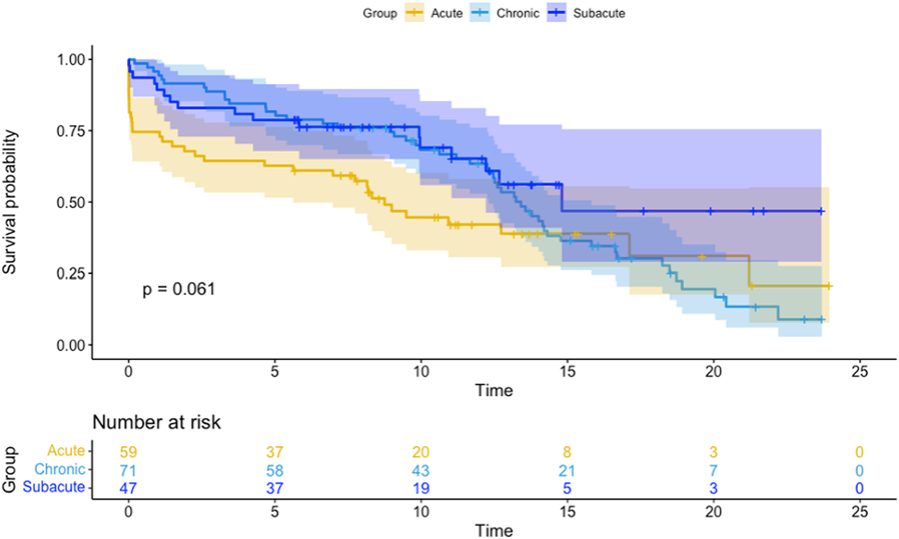
Kaplan–Meier survival curve for patients following coronary artery bypass grafting comparing timing of surgery after myocardial infarction

**Conclusions:** The timing of MI before CABG in patients with reduced LVEF has a direct impact on both short- and long-term survival rates and prolonged length of stay after surgery. Therefore, operation in acute settings (within 24 h) should be deferred as long as the patient remains clinically stable, as this is associated with better early and long-term outcomes.

## A63 Outcomes of patients presenting with non-ST elevation myocardial infarction undergoing surgical revascularization

### Marsioleda Kemberi^1^; Eduardo Urgesi^2^; Jing Yong Ng^3^; Mai Shehab^4^; Emma McEwen^3^; Wael Ibrahim Awad^5^

#### ^1^Barts and the London Medical School. Imperial College London. Bart's Heart Centre, London, UK; ^2^Barts Heart Centre. St Bartholomew's Hospital, London, UK; ^3^Barts and the London Medical School and Bart's Heart Centre, London, UK; ^4^Queen Mary University of London. King's College London Medical School, London, UK; ^5^Barts Heart Centre. St Bartholomew's Hospital; William Harvey Research Institute, QMUL, London, UK

**Correspondence**: Marsioleda Kemberi

*Journal of Cardiothoracic Surgery 19(1):* A63

**Objective:** To evaluate the in-hospital outcomes of patients undergoing CABG following a non-ST elevation MI (NSTEMI).

**Methods:** A retrospective analysis of all patients undergoing isolated CABG following NSTEMI from Sept 2017 to Sept 2022 at our Centre. Mann–Whitney U test and Chi-squared were used to compare patient characteristics, operative details, and in-hospital outcomes between patients that survived and those that died. Logistic regression analysis adjusting for age, gender and operative urgency was conducted to identify risk factors predicting mortality.

**Results:** 928 patients, mean age 63.53 ± 10.68 years, 782 (84.3%) male, undergoing 685 (73.8%) urgent, 210 (22.6%) elective, 33 (4.6%) emergency CABG procedures were studied. In-hospital mortality was 1.6% (15 patients). Patients who died had more co-morbidities: NYHA class III/IV (8 (53.3%) vs 216 (23.7%), p = 0.008), LVEF < 21% (1 (6.7%) vs 7 (0.8%), p = 0.014), poor mobility (5 (33.3%) vs 45 (4.9%), p = 0.042), PVD (6 (40%) vs 48 (5.3%), p < 0.0001) and severe renal impairment (8 (53.3%) vs 134 (14.7%), p < 0.001). The incidence of the following were higher among the patients that died vs those who survived: emergency operations (2 (14.3%) vs 30 (3.2%), p = 0.034), pre-op ventilation (2 (13.3%) vs 0, p < 0.0001), on inotropic support (2 (13.3%) vs 2 (0.2%), p < 0.0001) and IABP use (1 (6.7%) vs 10 (1.1%), p = 0.048). Logistic regression analysis confirmed the following variables as independent predictors of mortality: poor mobility (OR = 6.35, 95% CI 1.82–22.11, p = 0.004), PVD (OR = 7.78, 95% CI 2.42–25.01, p = 0.001), LVEF < 21% (OR = 11.35, 95% CI 1.07–120.26, p = 0.044), new postoperative stroke (OR = 20.25, 95% CI 3.47–118.24, p = 0.001), new haemodialysis (OR = 16.51, 95% CI 4.77–57.17, p < 0.0001).

**Conclusions:** NSTEMI patients undergoing isolated CABG have an increased risk of mortality if they have poor mobility, PVD, very poor LV function, suffered a new stroke or required dialysis. Larger studies are required to validate these findings.

## A64 Impact of high BMI in patients with reduced left ventricular function undergoing coronary artery bypass grafting

### Ettorino Di Tommaso^1^; Vito Domenico Bruno^2^; Lauren Kari Dixon^3^; Anil Sankanahalli Annaiah^4^; Gustavo Guida^4^; Roberto Natali^4^; Raimondo Ascione^2^

#### ^1^Bristol Heart Institute, University Hospitals of Bristol and Weston NHS Foundation Trust, Bristol (UK), UK; ^2^Bristol Medical School – Translational Health Science, Bristol, UK; ^3^Bristol Medical School – Public Health Science, Bristol, UK; ^4^Bristol Heart Institute, Bristol, UK

**Correspondence**: Ettorino Di Tommaso

*Journal of Cardiothoracic Surgery 19(1):* A64

**Objectives:** Obesity represents an independent predictor of mortality and morbidity following cardiac surgery. The recently developed concept of "Obesity Paradox" has not been described in high-risk patients with reduced left ventricular function ejection fraction (LVEF). The aim of this study is to investigate the short and long-term mortality of overweight patients with reduced LVEF after coronary artery bypass grafting (CABG).

**Methods:** A single-centre retrospective analysis based on 20 years of experience. 4,184 patients with reduced LVEF underwent CABG: 80.1% with moderate LV impairment (30–49%), 19.9% with severe LV impairment (< 30%). Patients were divided into groups: 1074 in Normal weight (BMI 18.5–24.99 kg/m^2^) and 3110 in Overweight (BMI ≥ 25 kg/m^2^). Our outcomes were short and long-term survival (≤ 20 years).

**Results:** Mean age was 68.6 ± 9.47 years, 84% male. Overweight patients were younger and had a better preoperative risk profile (Log EuroSCORE: 0.08 ± 0.09 vs 0.12 ± 0.13). In-hospital mortality rates were 4.9% Normal BMI vs 3.3% High BMI, and survival rates were 91% vs 93%, 54% vs 58% and 21% vs 25% (at 1, 10, 20 years), respectively (Fig. 1). The univariable regression model showed a protective effect of high BMI on mortality, although this was not confirmed in the adjusted model.

**Fig. 1:**
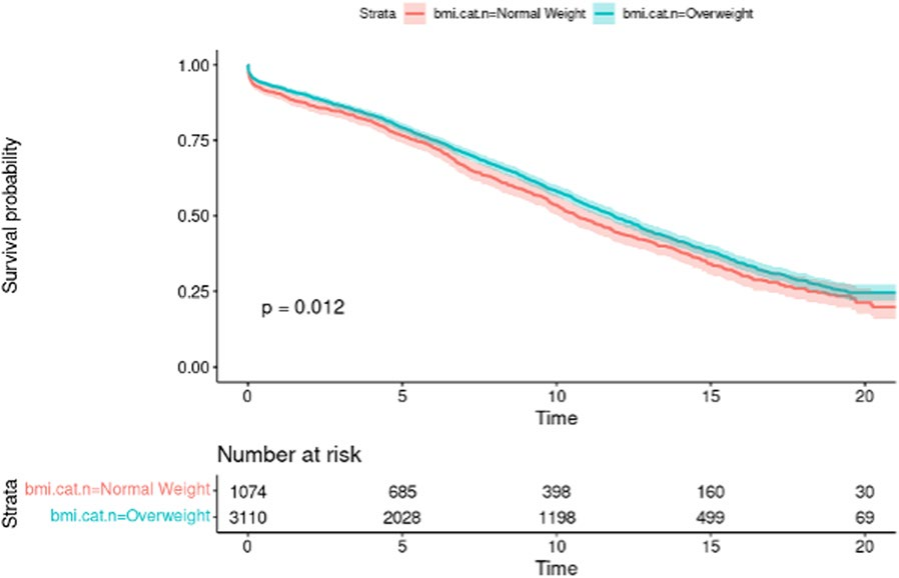
Kaplan–Meier survival curve for patients following coronary artery bypass grafting comparing patients with normal BMI to patients with high BMI

**Conclusions:** Overweight patients had better short and long-term survival compared to normal-weight patients but with a better preoperative risk profile. When adjusted for risk profile, the potential beneficial effect of the elevated BMI is nullified. The ‘Obesity Paradox’ doesn’t apply to patients undergoing CABG with reduced LVEF.

## A65 Impact of preoperative atrial fibrillation in patients with reduced left ventricular function undergoing coronary artery bypass grafting

### Vito Domenico Bruno^1^; Ettorino Di Tommaso^2^; Lauren Kari Dixon^3^; Anil Sankanahalli Annaiah^4^; Gustavo Guida^4^; Roberto Natali^4^; Raimondo Ascione^1^

#### ^1^Bristol Medical School – Translational Health Science, Bristol, UK; ^2^Bristol Heart Institute, University Hospitals of Bristol and Weston NHS Foundation Trust, Bristol, UK; ^3^Bristol Medical School—Public Health Science, Bristol, UK; ^4^Bristol Heart Institute, Bristol, UK

**Correspondence**: Vito Domenico Bruno

*Journal of Cardiothoracic Surgery 19(1):* A65

**Objectives:** Pre-operative atrial fibrillation (preAF) has a negative impact on postoperative outcomes after coronary artery bypass grafting (CABG), however its impact in patients with reduced LVEF has not been investigated. We aim to evaluate the impact of preAF on short- and long-term outcomes after isolated CABG.

**Methods:** This is a retrospective single-centre analysis of 4133 consecutive patients with reduced LVEF (< 50%) undergoing CABG at our institution over a period of 11 years. The primary outcome was short- and long-term mortality.

**Results:** 256 patients had preAF and they were older (71.41 ± 7.24 vs 66.70 ± 9.45 years, p < 0.01) and more frequently in NYHA class III/IV (48.8 vs 39.0%, p < 0.01) compared to those in Sinus Rhythm (SR). In-hospital mortality was almost double in preAF patients (5.9 vs 3.1%, p = 0.03), while there were no differences in post-operative complications nor in hospital length of stay. Long-term survival rates were as follow: at 5 years 63% vs 80.4%, at 10 years 34.3% vs 60.4% and at 20 years 7.8% vs 26% (preAF vs SR respectively, p < 0.01, Fig. 1). After adjustment for preoperative characteristics, preAF was an independent predictor for long-term mortality (HR 1.46, 95% CI: 1.26–168, p < 0.01).

**Fig. 1:**
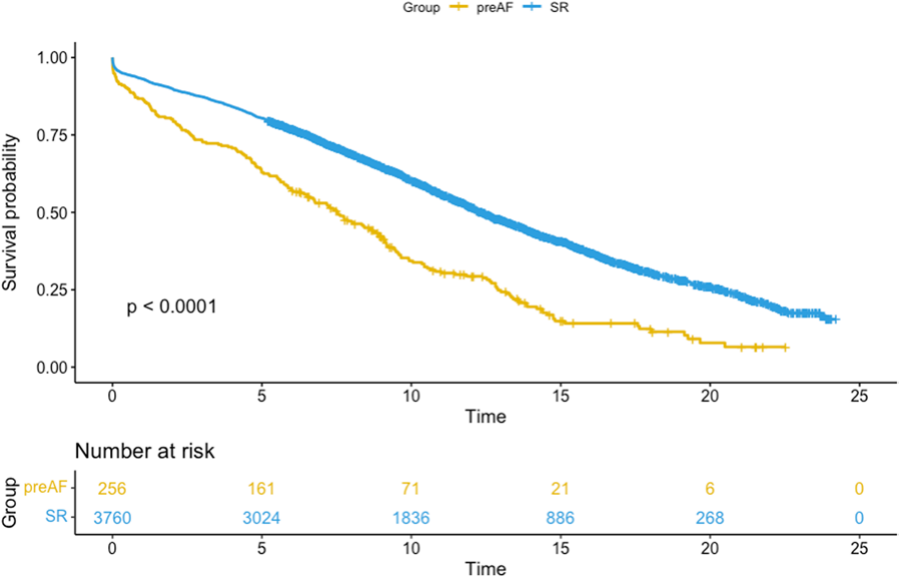
Kaplan–Meier survival curve for patients following coronary artery bypass grafting comparing patients with pre-operative atrial fibrillation to patients in sinus rhythm

**Conclusions:** Preoperative atrial fibrillation directly affects short- and long-term outcomes after coronary artery bypass in patients with reduced left ventricular function.

## A66 Early and medium-term outcomes in patients undergoing CABG with left ventricular dysfunction and left main stem disease; single centre experience

### Umair Aslam; Sam Poon; James Jones; Yasir Ahmed; Sobaran Sharma; Fabio Falconeri; Farhan Husain; Afzal Zaidi; Saeed Ashraf; Saleem Mujtaba

#### Morriston Hospital, Swansea, UK

**Correspondence**: Umair Aslam

*Journal of Cardiothoracic Surgery 19(1):* A66
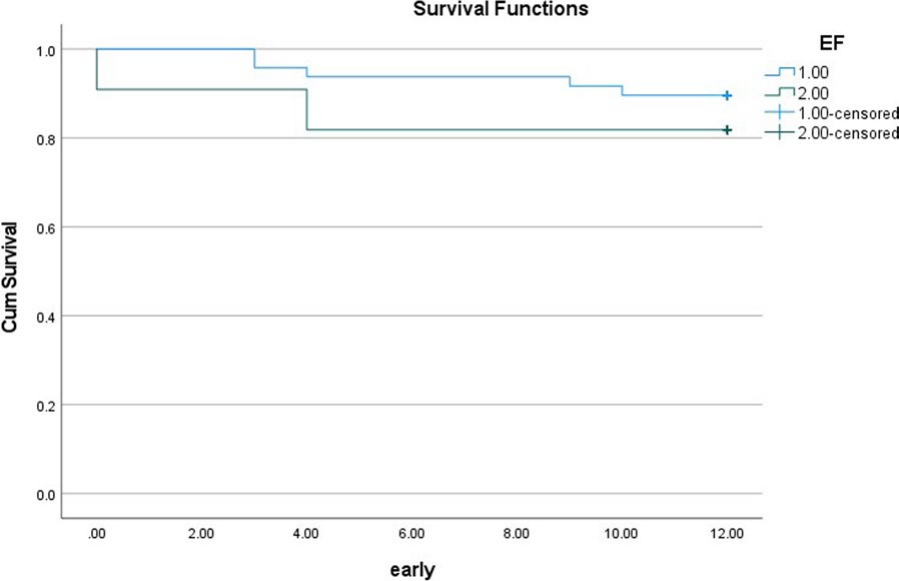


**Objectives:** To assess the early and medium-term outcomes for patients undergoing CABG with LMS and poor ventricular function.

**Methods:** We analysed 59 consecutives patients operated from January 2015 till August 2022. We divided the patients into I of II EF group: I group 21–30% (48 patients) and II group < or = 21% (11 patients). The ventricular function was assessed by transthoracic echocardiogram pre-operatively. Patients who required concomitant procedures were excluded. The group included patients who had elective, urgent and emergency surgery. KM survival analysis at 1 and 3 year used. Mean age 68 ± SD 8.8, Male: 81% (48/59), Smoker: 79% (47/59), Diabetes 46% (27/59), Elective 22%, urgent 71%, emergency/salvage 7%, Pre-op IABP = 36% (21/59), Pre-op cardiogenic shock = 6% (4/59), LV function = 48 poor (81%), 11 very poor (19%), Average EuroSCORE II = 12 ± SD 4.5, Bypass time: 114 min ± SD 33 min, Cross clamp time: 70 min ± SD 26 min.

**Results:** Group II had more post op complications as compared to group I. There were no in hospital and 30 day deaths in Group I. Operative mortality = 1.7% (1/59), Stroke = 0, Renal failure requiring dialysis = 5% (3, Group II), Re-exploration for bleeding = 3.3% (1 in each group), Average length of hospital stay = 12 days ± SD 5.8 days, 1 repeat PCI post-op (1.7% Group I), New ICD implantation = 8.4% (5/59). Some patients were lost to follow-up so echocardiogram not performed in those patients. The rest who had Echocardiogram at 6, 12, and 36 months showed as below.

Improvement in LV; (1) From Poor to moderate (30–50%): **68%** (21/31 patients), (2) Very poor to moderate: 12% (4/31), (3) Very poor to poor: 12% (4/31), (4)LV unchanged (poor to poor) 6% (2/31).

year = Overall 88%, Significantly better in Poor LV compared to Very Poor LV (90% vs. 82%, p = 0.04), 3 years = Overall 74%.

**Conclusions:** Our data suggests that CABG be undertaken safely in poor LV patients with LMS and with good outcomes.

## A67 CABG in a case with coronary arteries from a single coronary ostium in the right coronary sinus

### Ahmed Shazly; Mohamed Osman; Hasnat Khan; Nickolaos Charokopos; Arvind Singh; Alessia Rossi; Sudhir Bhusari; Alberto Albanese; Younus Qamar

#### Essex Cardiothoracic Centre, Basildon, Basildon, UK

**Correspondence**: Ahmed Shazly; Younus Qamar

*Journal of Cardiothoracic Surgery 19(1):* A67

**Introduction:** Coronary arteries originating from a single coronary ostium in the aorta are rare, occurring in less than 0.03% of the general population. The first report of a single coronary artery was by Hyrtl in 1841.

**Case report:** We are presenting a 79-year-old gentleman who was referred for CABG.

His coronary angiogram showed left main stem originates from the right coronary sinus adjoining the right coronary artery origin. The left main continues as a single vessel passing anterior to the pulmonary artery and then divides into LAD and circumflex branches. At the origin of left main, it gives off a small septal branch that supplies the proximal part of the interventricular septum. The circumflex is a smaller vessel continuing as an inferior obtuse marginal. RCA is a large dominant vessel originating from the right coronary sinus and giving rise to the sinus node artery proximally. Distally it divides into a long PDA and a good sized posterolateral artery. Intra operatively, the LM was found to be very calcified along its course. Vein graft was anastomosed distally before its bifurcation. The graft top end was anastomosed to mid aorta.

**Discussion:** Shirani and colleagues classified single coronary ostium into 20 categories on the basis of the ostium’s location and the path of any aberrantly coursing coronary artery.

Among 97 instances of solitary ostium, they described the cases of four patients in whom the right coronary artery gave rise to an LAD and continued to travel in the AV groove past the crux that supplied the LCx territory. Our patient’s case has the characteristics of type RIIA of this classification (see the Fig. 1).

**Fig. 1:**
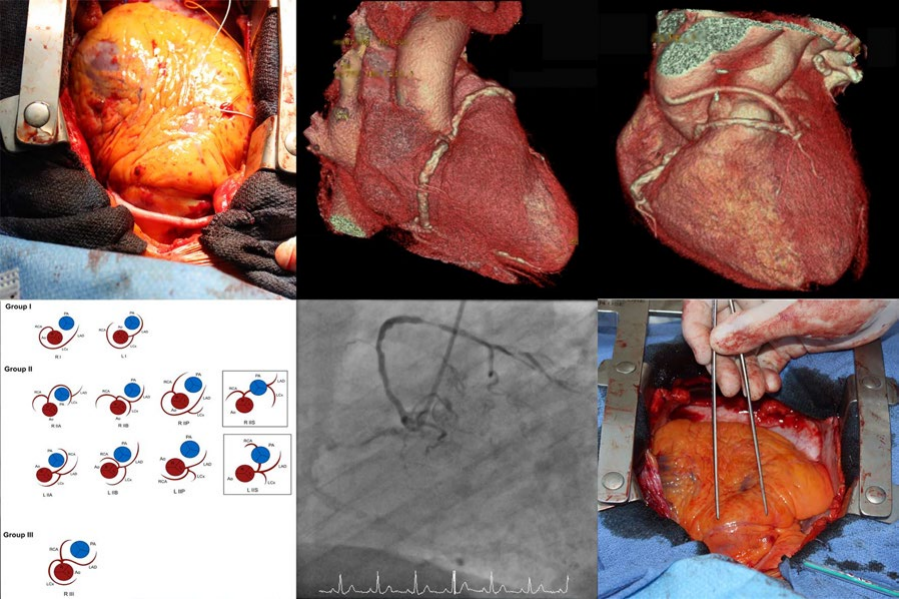
Intra-operative, 3D reconstructed CTCA and coronary angiography images and classification of single coronary ostium. *Images were taken and published after obtaining the patient’s consent

## A68 The effects of raised BMI in patients undergoing isolated coronary artery surgery: a single centre experience

### Shahan Shahid; Alice Mines; Stavroula Tata; Azhar Hussain; Gentjan Jakaj; Irfan Rasheed; Ranjit Deshpande; Habib Khan

#### Kings College Hospital, London, UK

**Correspondence**: Shahan Shahid

*Journal of Cardiothoracic Surgery 19(1):* A68

**Objectives:** Increasing levels of obesity are associated with ischaemic heart disease and systemic comorbidities. A rising proportion of patients undergoing surgery for occlusive atherosclerotic disease have a raised BMI. This retrospective study from a single UK centre, aims to investigate the effect of obesity on short term outcomes following CABG surgery.

**Methods:** Patients treated with isolated CABG between 2012 and 2022 were grouped according to their BMI; < 30 not obese, 30–35 mild obesity, 35–40 moderate obesity and > 40 morbid obesity. Baseline characteristics and outcomes were recorded after retrospective review of case notes. The primary outcome was 30-day mortality. Secondary outcomes were sternal wound infection and stroke.

**Results:** There were 3278 patients identified; 1762 patients < 30 BMI, 1192 patients 30–35 BMI, 253 patients 35–40 BMI, 71 patients > 40 BMI. The 30-day post-operative mortality was 0.78% in the under 30 BMI, 0.69% in the 30–35 BMI group, 0% in both the 35–40 and > 40 BMI group. The incidence of stroke was 0.93% in the under 30 BMI, 1.23% in the 30–35 BMI group, 0.79% in the 35–40 and 0% in the > 40 BMI group. The rate of sternal wound infections was 0.47% in the under 30 BMI group, 0.84% in the 30–35 BMI group, 1.58% in the 35–40 and 4.22% in the > 40 BMI group.

**Conclusions:** Patients with a raised BMI undergoing isolate CABG have a similar early mortality rate and incidence of sternal wound infection as compared to those with a normal BMI. However, patients with extreme BMI’s suffer a slightly higher rate of sternal complications, although this is not significant.
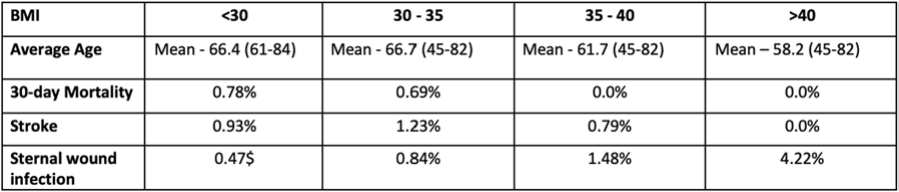


## A69 25 Year outcomes of patients undergoing minimally invasive direct coronary artery bypass surgery: a single-centre experience

### Oluwanifemi Akintoye; Aabha Divya; Shagorika Talukder; Clare Lau; Shakil Farid; Ravi De Silva

#### Royal Papworth Hospital NHS Foundation Trust, Cambridge, UK

**Correspondence**: Oluwanifemi Akintoye

*Journal of Cardiothoracic Surgery 19(1):* A69

**Objectives:** Minimally invasive direct coronary artery bypass (MIDCAB) is a safe alternative for revascularisation of proximal left anterior descending artery (LAD) disease. MIDCAB may lead to faster recovery without affecting freedom from revascularisation or mortality and morbidity following coronary artery bypass surgery (CABG) compared to open techniques. The objective of this study was to evaluate the 25-year outcomes of patients who underwent this surgical technique of CABG.

**Methods:** This was a retrospective, observational study of patients who underwent MIDCAB (with lower midline sternotomy approach only) between December 1996 and June 2021. Data was collected using electronic medical records, telephone follow-up conversations and community records. The primary outcome measure was mortality while secondary outcomes included revascularisation, stroke, myocardial infarction (MI) and wound infection. Survival analysis was performed using the Kaplan–Meier method.

**Results:** A total of 215 patients were identified in the study period undergoing MIDCAB at our centre. The median age was 77 years with 83.3% being male patients. The median follow-up period was 16 years. At follow-up, freedom from repeat LAD revascularisation and from other vessels revascularisation was 96.7% and 89.1% respectively. Wound infection occurred in 11 (5.1%) and MI in 6 (2.8%) patients. Survival rates were 99.5%, 81.0%, and 45.2% survival at 1-year, 10-year, and 25-year respectively. Univariate analysis showed increasing age (p < 0.01, HR 1.08 CI 1.05–1.11) and LVF (p < 0.01, HR 2.40, CI 1.66–3.45) as factors associated with mortality.

**Conclusions:** Our single-centre experience of MIDCAB demonstrated excellent long-term freedom from revascularisation and other complications over 25 years of review. Although limited by the retrospective nature, the study showed a trend towards MIDCAB as a safe procedure for definitive revascularisation.

**Adult Cardiac Miscellaneous**

## A70 Focused liaison service for the elderly: expanding an established service into a tertiary centre for cardiac surgery

### Ben Wildblood^1^; R Davies^1^; Cha Rajakaruna^2^; C Huang^1^

#### ^1^Bristol Royal Infirmary, Bristol, UK; ^2^Bristol Heart Institute, Bristol, UK

**Correspondence**: Ben Wildblood

*Journal of Cardiothoracic Surgery 19(1):* A70

**Objectives:** The Patient focUsed Liaison Service for the Elderly (PULSE) was established in the Bristol Heart Institute in 2020, following a pilot study estimating a potential saving of 700 bed days per annum through reduced readmissions. This consultant led service provides holistic care for cardiology patients in inpatient and outpatient settings.

Interest from the cardiac surgery team has led to consideration of expanding into this department. Here we report on an assessment of frailty in cardiac surgery inpatients in general ward and cardiac intensive care (CICU) settings.

**Methods:** Cardiac surgery inpatients were assessed for frailty using the clinical frailty scale on a given day in May 2022. The presence of any typical geriatric-related pathology was also noted, in addition to demographic data. Comparative data was also collected for all medical cardiology inpatients.

**Results:** Data were collected for 36 patients in cardiac surgery. Ward-based patients showed low levels of frailty (CFS ≥ 5 = 0%), although many were deemed to be vulnerable due to underlying disease (CFS4 = 47%). Cardiac surgery patients in the CICU setting were more likely to be frail (CFS ≥ 5 = 41%, p = 0.002) when compared with those outside of CICU. All with at least moderate frailty had presented acutely. The number of major geriatric-related pathologies was similarly higher in CICU patients (1[0–2]) compared to those on the general cardiac surgery wards (0[0–0], p = 0.02).

When compared with 51 medical cardiology inpatients, cardiac surgery inpatients were significantly younger (60[53–77] vs 71[59–80], p = 0.04), but displayed similar rates of frailty (CFS 4[3–4] vs 3[3–5], p = 0.99) and major geriatric pathology (p = 0.48).

**Discussion:** Care of the elderly liaison services to cardiac surgery are likely to be most effective when targeted to the intensive care setting and to patients admitted acutely. The authors would encourage the development of similar cardiology liaison services in other UK tertiary cardiac.

## A71 Timing and outcomes of surgery for active infective endocarditis in general population and patients with preoperative neurological complications

### Dora Pestotnik Stres^1^; Antony Walker^2^

#### ^1^Lancaster University, Lancaster, UK; ^2^Blackpool Teaching Hospitals NHS Foundation Trust, Blackpool, UK

**Correspondence**: Dora Pestotnik Stres

*Journal of Cardiothoracic Surgery 19(1):* A71

**Objectives:** The incidence of infective endocarditis (IE) is increasing. The role of and indications for surgery are well established. Controversy remains regarding the optimal timing of surgery, especially after preoperative neurological complications (PNC). This study aimed to investigate the effects of timing of surgery for active IE on short and long-term outcomes in general population and in patients with PNC.

**Methods:** Single centre retrospective study of patients undergoing surgery for active IE between May 1996 and July 2021. Patients were divided into very early (≤ 2 days between admission and surgery), early (3–7 days) and late surgery (8–42 days) groups and their preoperative risk factors assessed using EuroSCORE and its components. Short and long-term outcomes were compared between the groups and subgroups with PNC. Appropriate statistical analysis was performed with p < 0.05 taken as significant.

**Results:** 216 patients (38 with PNC) were included. Mean age of patients was 55.8 years, 77.3% were male. Effected valves were aortic (53%) and mitral (17%). Very-early surgery group had higher additive (p = 0.002) and logistic (p = 0.003) EuroSCOREs than other groups. No significant differences were found in EuroSCOREs between the PNC subgroups (p = 0.815, p = 0.547). ICU and postoperative stays, short- and long-term (up to 10 years) mortalities did not differ significantly between the groups or subgroups. 30-day mortality ranged from 6.7%-12.3% (p = 0.429) between the groups, 10-year mortality from 21.0%-24.4% (p = 0.886).

**Conclusions:** Timing of surgery in general population showed no apparent impact on the outcomes following valve surgery for IE. Despite a higher risk profile of the very-early surgery (≤ 2 days), there was no difference in outcomes. This indicates a possible protective effect of earlier surgery. In presence of PNC, timing of surgery. In presence of PNC, timing of surgery does not seem to affect the outcomes (Fig. 1).

**Fig. 1:**
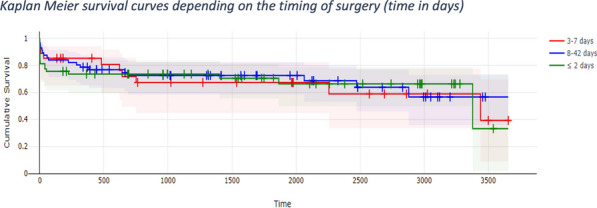
Kaplan Meier survival curves depending on the time of surgery (time in days)

## A72 Current practice in beta-blocker prescribing in post-operative cardiac surgery: a single-centre experience

### Ann Cheng; Oliver Dixon; Norman Briffa

#### Northern General Hospital, Sheffield, UK

**Correspondence**: Ann Cheng

*Journal of Cardiothoracic Surgery 19(1):* A72

**Objectives:** We aimed to evaluate the current practice in beta(β)-blocker prescription in post-operative cardiac surgery patients and the development of post-operative atrial fibrillation (POAF) at a tertiary cardio-thoracic centre in the UK.

**Methods:** The local hospital database was searched retrospectively for 100 consecutive patients undergoing cardiac surgery up to 12/31/2021. Patients undergoing open cardiac surgery in sinus rhythm pre-operatively were included; patients deceased in hospital or with incomplete data were excluded. Basic demographic and case urgency were also recorded. Data extracted included: (1) pre-operative medication (2) post-operative day (POD) and dose of β-blocker prescribed in the immediate post-operative period, and (3) presence of atrial fibrillation defined as a post-operative complication on discharge letter or prescription of amiodarone on discharge. Statistical analysis, where applicable, was performed with chi-squared test for categorical data.

**Results:** 100 patients (71 male, 29 female) underwent cardiac surgery over a period of three months. The average age was 64 years (SD = 13, range 22–87). The case urgency were representative of the general caseload at the authors' institution.

The average time from the operation to receiving the first dose of β-blocker post-operatively was 2.3 days (mean 2.3, mode 1, median 2; range 0–13). Table 1 demonstrates the rate of POAF development against prescription of pre-operative β-blocker. 32% (17/53) of patients on pre-operative β-blocker developed POAF, versus 45% (21/47) of patients not on pre-operative β-blocker. Whilst the difference may be clinically significant, it was not statistically significant (X2 = 1.68, p = 0.195).

**Table 1:**
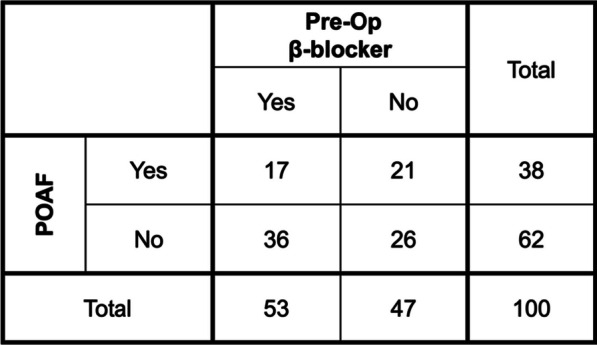
Rate of POAF against pre-op β-blocker prescription

**Conclusions:** Routine prescription of β-blocker pre-operatively should be considered in adult cardiac patients, however, more research may be required to inform best peri-operative practice in preventing POAF.

## A73 Waiting well for cardiac surgery; using digital technology to ensure patients are safe and prepared for pre-operatively

### Cathy Walters^1^; Hillary Schrauwers^1^; Ana Alves^2^; Lepalele Sepa^1^

#### ^1^Guys and St Thomas’ NHS Trust, London, UK; ^2^St Thomas Hospital, London, UK

**Correspondence**: Cathy Walters; Hillary Schrauwers; Ana Alves; Lepalele Sepa

*Journal of Cardiothoracic Surgery 19(1):* A73

**Background:** The COVID-19 pandemic caused global disruption to the provision and delivery of cardiac surgery. Cognisant of our responsibility to safeguard the health and wellbeing of our patients who are at risk of deterioration whilst waiting for surgery we set about finding a solution.

**Objectives:** To ensure early detection of deterioration and swift action to prevent/reduce mortality and morbidity of patients waiting for surgery.

**Methods:** We worked closely with Ortus to develop a platform for our patients. All patients were onboarded onto the platform as they were added to the waiting list. The inputted information about their symptoms. The clinical team monitored this information remotely, prompting intervention for those patients who needed it.

**Results:** 71% of patients on the waiting list are actively using the app, 109 of these that were identified as having a red flag and were identified as at risk of deteriorating based on data captured via the platform and subsequently escalated to their respective consultant. 20 of those patients have had their surgery completed.

**Conclusions:** The use of the app has been part of a transformation change in the way we work with patients. It has the potential to benefit all patients who are at risk of deterioration for any wait for surgery.

## A74 Apply ERAS protocol for patients undergoing cardiac surgery 2nd cycle of an audit

### Bassem Gadallah; Aisling Kinsella; Sona Harbish

#### Mater Misericordiae University Hospital, Dublin, Ireland

**Correspondence**: Bassem Gadallah

*Journal of Cardiothoracic Surgery 19(1):* A74

**Objectives:** This is the 2nd Cycle of an Audit to apply ERAS protocol for patients undergoing Cardiac surgery.

In the previous cycle the overall median duration of mechanical ventilation is 14.5 h [11.25–17.75] and for the ERAS cohort 12.75 h [10.5–16]. In this cycle, we aimed to shorten ventilation aiming to Extubate within six hours of arrival to ICU as per ERAS guidelines.

**Methods:** Post-Operative Strategies Targeted include Glycaemic control, Pain management, post-operative normothermia and avoiding hyperthermia, Delirium management, Monitoring renal function and preventive strategies for AKI, Goal-directed fluid therapy, extubation within 6 h of ICU arrival.

**Results:** 62 patients were included, the demographics of patients in both cycles are very similar. More than 50% of patients underwent CABG in both cycles. In the 2nd Cycle, there is a reduction of the overall median duration of mechanical ventilation from 14.5 h to nine hrs. The median time to extubation in our ERAS-appropriate candidates improved from 12.75 h to seven hrs. Also, there is a reduction of fluid resuscitation before extubation from a median of 3.1 L to 1.1L. Atrial Fibrillation was the most common post-op complication (42%), Failed extubation rate was 3.2%, and Delirium occurred in 4.8%. ICU stay for the ERAS Group was close to 1 day (23 h) and two days for the non-ERAS group (45 h). The median hospital stay was 10 days in both cycles of the Audit.

**Conclusions:** Shortening MV is feasible and can lead decrease in ICU stay. Improving the median time to extubation close to six hrs is feasible with an acceptable rate of postoperative complications.

## A75 Outcomes following convergent procedure for long-standing persistent atrial fibrillation utilising implantable loop recorder analysis

### Hannah Jesani; Faizel Osman; Thomas Barker

#### University Hospital Coventry and Warwickshire, Coventry, UK

**Correspondence**: Hannah Jesani

*Journal of Cardiothoracic Surgery 19(1):* A75

**Objectives:** To evaluate the outcomes of hybrid atrial fibrillation (AF) ablation with the Convergent procedure utilising implantable loop recorders (ILR) for continuous rhythm analysis.

**Methods:** This prospective study analysed all patients who underwent the Convergent procedure. Patient demographics, operative details, and ILR rhythm analysis was undertaken. Conversion and sustainability of sinus rhythm, and procedural complications were recorded.

**Results:** In total, 23 patients underwent the Convergent procedure from December 2017 to December 2021. Eighteen patients had completed both surgical and percutaneous ablations in this timeframe. Twenty (87%) were male with a mean age of 62 years (range 44–78). Prior percutaneous catheter ablations had been undertaken in 16 (70%). All patients were discharged following epicardial ablation in sinus rhythm. Analysis of ILR revealed freedom from AF at three months in 11/15 (73%), six months 11/14 (79%), and 12 months 10/13 (77%) patients. At 18 months freedom from AF was 56% (5/9). There were two complications which resolved with conservative management.

**Conclusions:** This prospective study has shown the Convergent procedure to be a safe and effective method to convert patients to sinus rhythm. Further follow up is being undertaken to assess efficacy over longer timeframes with an increased number of patients.
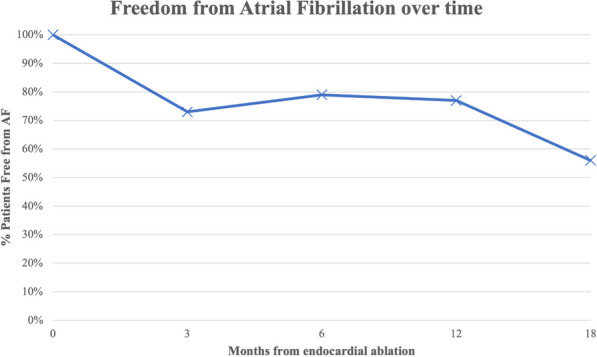


## A76 Hybrid epicardial and endocardial ablation for long-standing persistent atrial fibrillation: a systematic review and meta-analysis

### Hannah Jesani; Saifullah Mohamed; Faizel Osman; Thomas Barker

#### University Hospital Coventry and Warwickshire, Coventry, UK

**Correspondence**: Hannah Jesani

*Journal of Cardiothoracic Surgery 19(1):* A76

**Objectives:** To undertake a systematic review and meta-analysis of hybrid atrial fibrillation (AF) ablation procedures (Convergent procedure and thoracoscopic hybrid ablation) to evaluate the outcomes and complication rates.

**Methods:** A literature search using MEDLINE and EMBASE databases for Convergent procedure and hybrid thoracoscopic ablation was undertaken. The Preferred Reporting Items for Systematic Reviews and Meta-Analysis (PRISMA) guidelines were followed. Following removal of duplicate studies, 102 full text manuscripts were reviewed by two independent reviewers. Included studies were analysed for the reported success rates in conversion to sinus rhythm and complication rates.

**Results:** In total 32 studies fulfilled the inclusion criteria. This incorporated 15 studies on Convergent procedure and 17 on thoracoscopic hybrid ablation. Comparing these methods of hybrid ablation for persistent AF, there was no significant difference in freedom from AF at 12 months (74% vs. 80%; p = 0.14), complication rates (10% vs. 12%; p = 0.77) or mortality rates (0.9% vs. 0.5%; p = 0.24). There was a wide variability in reported success rates among these studies.

**Conclusions:** The Convergent procedure and thoracoscopic hybrid ablation procedures provide similar efficacy in restoration of sinus rhythm, and complication rates. Randomized control trials are required to compare these differing hybrid ablation techniques for long-standing persistent atrial fibrillation.
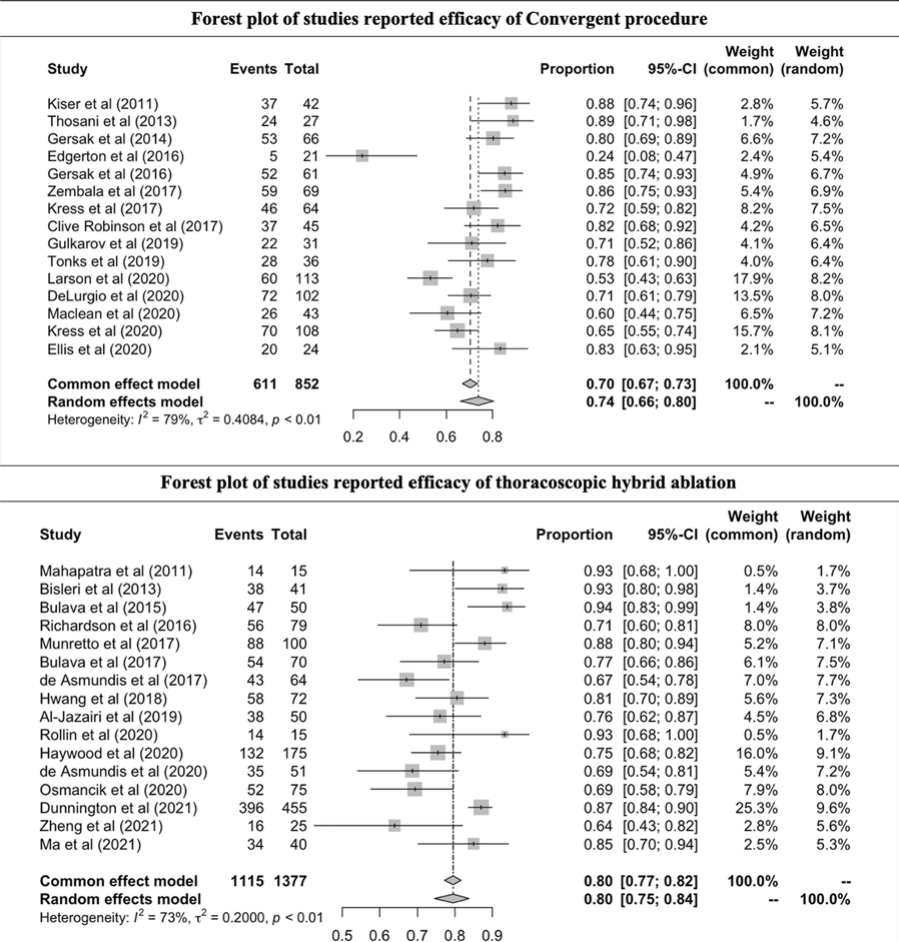


## A77 Adequacy of discharge information after cardiac surgery: a quality improvement project

### Daniel Nie^1^; Vinci Naruka^1^; Adeyemi Olayiwola^1^; Minahil Khan^2^; Kerin May Borer^2^; Laerke Ghosh^1^; Shirish Ambekar^1^

#### ^1^Barts Health NHS Trust, London, UK; ^2^Barts and the London School of Medicine and Dentistry, London, UK

**Correspondence**: Daniel Nie

*Journal of Cardiothoracic Surgery 19(1):* A77

**Objectives:** Good discharge information following surgery is essential for optimising outcomes, aiding information retention and improve transition to home. An initial study looked at the adequacy of discharge information in March 2022 revealing a large proportion of patients (38.5%) not retaining key information. The aim of our study was to determine whether additional resources such as specialist nurse-led discharge talks, providing handouts for these talks and a pictorial presentation of wound red flags would improve retention of key information.

**Methods:** We conducted a first round of patient interviews in March 2022 followed by a second round in September 2022. These were completed via telephone at 6 weeks after their discharge. The 29 patients identified in each round underwent either Coronary Artery Bypass Graft or Aortic Valve Replacement surgery. We asked the identical 23 questions in both cycles, designed from the discharge talk which included nine testing questions. Patient data was anonymised.

**Results:** 29 patients were collected from each time period. The age distribution was: < 50 (3.4%); 50–70 (48.3%); > 70 (48.3%) in March 2022 and < 50 (10.3%); 50–70 (65.5%); > 70 (24.2%) in September 2022. 72.4% were male in the first round compared to 86.2% in the second. Questionnaire results are in Table 1.

**Table:** 

Parameter	March 2022	September 2022
Number of patients	29	29
Operation	CABG: 75.9% AVR: 24.1%	CABG: 86.2% AVR: 13.8%
Patients given discharge information (%)	89.7	96.6
Patient knowing best sleeping position (%)	57.7	67.9
When to drive or lift light objects (%)	53.6	75.9
Identifying wound red flags (%)	61.5	65.5
First point of contact for wound concerns (%)	39.3	62.1
Identifying concerning symptoms (%)	42.9	81.5
Returning to work (%)	19.2	20.7

**Conclusions:** We demonstrate an improvement in knowledge retention as a result of our interventions. Resources such as pictorial demonstrations of wound healing should be included to aid discharge information and increase quality of educational content for patients from diverse backgrounds, thereby aiding successful self-management at home.

## A78 The use of high-flow nasal oxygen (*HFNO*) for post-operative cardiothoracic surgical patients: a review of an intensive care unit (*ICU*) experience

### Ujjawal Kumar; Nicola Jones; Daniel Aston

#### Royal Papworth Hospital, Cambridge, UK

**Correspondence**: Ujjawal Kumar

*Journal of Cardiothoracic Surgery 19(1):* A78

**Objectives:** Hypoxia and respiratory failure are amongst the commonest complications following cardiothoracic surgery and prolonged respiratory failure is associated with adverse outcomes. HFNO is a non-invasive ventilation modality that is commonly used in our ICU but good evidence for the superiority of HFNO over more established oxygen therapies is lacking. We reviewed how HFNO is used in our unit to help safeguard best practice and facilitate enhanced recovery for postoperative patients.

**Methods:** A retrospective observational study was undertaken on all patients who received HFNO during their postoperative admission to our ICU between January–June 2022 using the electronic medical record system. Data collected included risk factors for postoperative pulmonary complications (PPCs), indication for HFNO use, ventilation strategy prior to and following HFNO, as well as arterial blood gas values prior to HFNO initiation, after one hour on HFNO, and following HFNO cessation.

**Results:** 107 patients were included in this study; mean age of 60 years (range 22–82). The modal risk factor for PPCs was a smoking history of greater than 20 pack-years (54%). CABG+ other cardiac procedures were the modal cause of ICU admission. Postoperative atelectasis was the modal indication for HFNO. Poor LVEF (< 30%) was associated with longer duration of HFNO therapy. Patients extubated directly onto HFNO had a shorter ICU admission than those extubated onto other ventilation mechanisms.

**Conclusions:** Our study supports HFNO as a useful tool to aid recovery in patients with risk factors for PPCs, or patients who are hypoxemic but with appropriate progression. We additionally find that HFNO is used for patients who are unable to tolerate facemasks and/or where removal of the facemask results in rapid desaturation. HFNO may also benefit patients with increasing oxygen demands to achieve suitable ABG values, and lastly for patients with stable-high oxygen requirements immediately post-extubation.

## A79 A case report of right anterior thoracotomy for RCA aneurysm repair: Is it the best approach?

### Youssef Abouelela; Arvind Singh; Hasnat Khan; Sudhir Bhusari; Alberto Albanese; Alesia Rossi; Mohamed Osman; Arun Venkitaramanan; Swamy Gedela

#### Essex Cardiothoracic Centre, Basildon, Essex Cardiothoracic Centre, Basildon, UK

**Correspondence**: Youssef Abouelela

*Journal of Cardiothoracic Surgery 19(1):* A79

**Introduction:** Patients with coronary artery aneurysms often undergo conservative management, sometimes interventional techniques or surgery is planned especially with compression or shunt symptoms. With coronary artery aneurysms, a variety of non-specific presenting symptoms including angina-like pain and shortness of breath exist.

**Case Presentation:** A 79-year-old gentleman with a background of a coronary artery bypass graft (CABG), abdominal aortic aneurysm (AAA), hypertension, hernia repair and an ex-smoker for 40 years presented with a six-month history of progressive shortness of breath and was subsequently reviewed in community cardiology where he was found to have a giant expanding right coronary artery aneurysm measuring 18 cm × 10 cm × 9 cm on CTCA aorta and his echocardiogram showed a thrombus in situ. He was managed with a surgical approach via thoracotomy.

The patient gave explicit permission to publish their information and images in an open access journal.
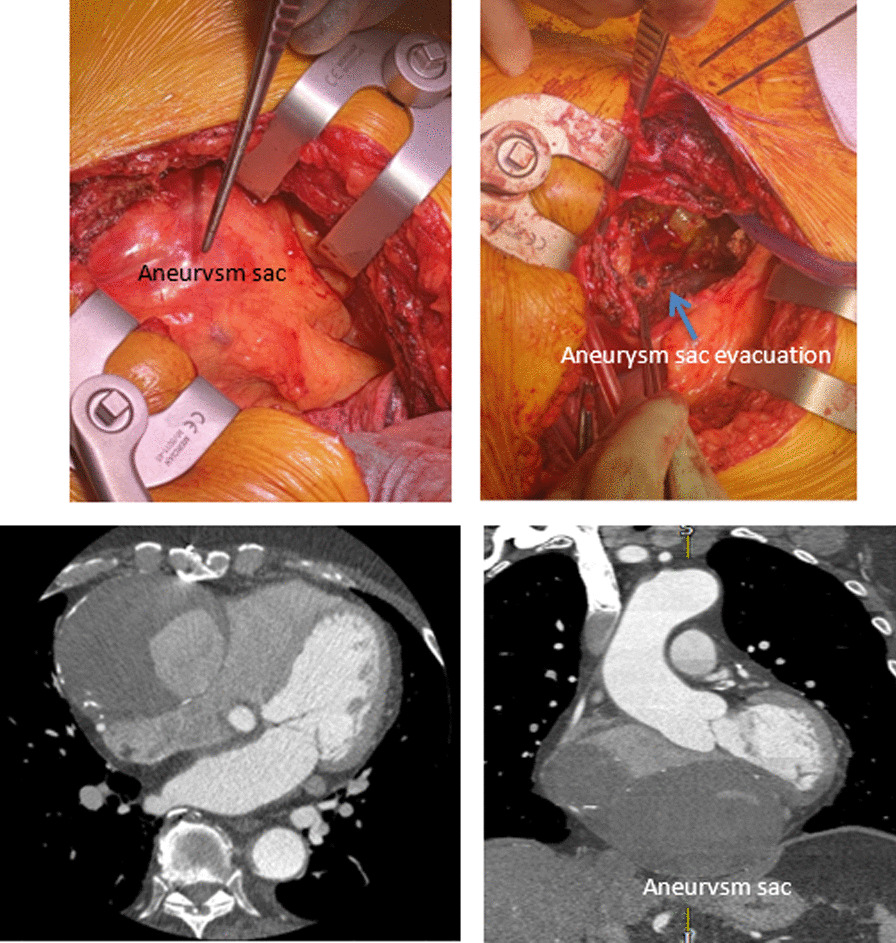


**Discussion:** In the event of coronary artery aneurysms, there are multiple approaches employed in managing. However, the evidence is still ill-defined and mostly consists of expert opinion and case reports.

**Conclusions:** We performed a repair of a right coronary artery aneurysm using an anterior thoracotomy approach in our case. There is a need for an evidence-based approach, but we recognize that randomized controlled trials are extremely difficult, given how seldom the disease is encountered.

## A80 The cardiothoracic anaesthesia partnership: collaborative education in cardiothoracics

### Anders Hulme^1^; Paul Balfour^1^; Martin Yates^2^; Roger Cordery^2^

#### ^1^Harefield Hospital, London, UK; ^2^Barts Heart Centre, London, UK

**Correspondence**: Anders Hulme

*Journal of Cardiothoracic Surgery 19(1):* A80

Born out of the Barts Heart Centre Fellows club, the Cardiothoracic Anaesthesia Partnership (CAP) is a free, monthly, multidisciplinary, multicentre educational programme providing advanced level cardiothoracic teaching primarily aimed at anaesthesic registrars and fellows. Through themed meetings we deliver high quality teaching from anaesthetists, surgeons, intensivists and cardiologists, to cover all aspects of the chosen topic for a given month. The teaching has evolved and developed into a regular international educational meeting delivered in person and on MS Teams. All CAP meetings feature at least one surgical talk as it is imperative for anaesthetists involved in cardiac and thoracic anaesthesia have a thorough grasp of the surgical aspects of the procedures in question. The close involvement of surgeons in our meetings allows for improved understanding of surgical techniques and decision making, thus facilitating the delivery of optimal levels of care for patients. CAP has also seen increasing attendance from cardiothoracic surgical trainees looking to gain further knowledge and experience of the perioperative journey of their patients, as well as develop greater understanding of the challenges faced by their anaesthetic and critical care colleagues.

CAP has adapted to the increased surgical presence at its meeting by appointing two surgical leads—one trainee and one consultant—to shape meetings, suggest topics and speakers and ensure that over the three-year cycle of topics, we have completely covered a given area of cardiothoracic surgery. Our website www.CardiothoracicAnaesthesia.com has had over 26,000 visitors and we host videos of all our presentations to allow retrospective viewing and reviewing.

We feel the CAP is a unique entity in medical education in the UK, possibly the world, due to our collaborative structure, regularity and accessibility.

## A81 Acute bowel ischemia is a rare but devastating complication following cardiac surgery: retrospective analysis of 41 patients over a period of 18 years

### Ammar Mustafa^1^; Jeni Palima^2^; Abdalmahmoud Elsiddig^2^; Jens Roggenbach^2^; Sanna Elgaddal^2^; Mahmoud Abdelaziz^2^

#### ^1^Heart and Lung Centre, New Cross Hospital, Wolverhampton, UK; ^2^New Cross Hospital / Heart and Lung Centre, Wolverhampton, UK

**Correspondence**: Ammar Mustafa

*Journal of Cardiothoracic Surgery 19(1):* A81

**Introduction:** Acute bowel ischemia (ABI) is a rare but life-threatening complication after cardiac surgery. The incidence is very low (less than 1%), however, very high mortality rates have been reported (between 50 and 100%). Unfortunately, no major advances have been shown to improve the dismal prognosis over the last few decades. The commonest mechanism of ABI is non-occlusive mesenteric ischemia (NOMI) which results from reduction in splanchnic blood flow secondary to low cardiac output.

**Objectives:** This is a retrospective analysis to assess: the incidence, perioperative risk factors and postoperative outcomes of all the patients who suffered ABI post cardiac surgery of any type in our institution between 2004 and 2022.

**Methods:** All our cardiac surgical patients were screened for GI complications. Patients with confirmed ABI were reviewed retrospectively for: patients’ characteristics, perioperative predictors of ABI and postoperative outcomes including in-hospital mortality.

**Results: O**ut of 15,472 patients, 41 (0.26%) suffered ABI postoperatively. 23 patients (56%) underwent CABG alone, 10 patients (24%) had CABG+ Valve and 7 (17%) were emergency operations. 38 (93%) developed leucocytosis prior to the diagnosis while all patients had metabolic acidosis with an average peak serum lactate level of 11.5 mmol/L. The in-hospital mortality rate was 76% (32 patients). All the 9 survivors underwent bowel resection within 24 h from reaching ABI diagnosis while the average waiting time to laparotomy or death for the 32 patients who died was 2.7 days.

**Table:** 

Characteristic/predictor	Mean ± 2SDs or number (%)
Mean Age	72.51 ± 9.63
Female Gender	14 (34%)
SCTS Logistic EuroSCORE	13.91 ± 4.27
Poor Left Ventricle (EF < 30%)	9 (22%)
Peri-operative IABP support	11 (27%)
Cumulative Cardiopulmonary Bypass time	124 min
New Hemofiltration/Dialysis	19 (46%)
Prolonged Postoperative Ventilation	15 (37%)
Significant Inotropic/Vasopressor Support	23 (56%)
Postoperative Atrial Fibrillation	17 (41%)
Re-exploration for Bleeding	9 (22%)

**Conclusions:** There are several predictors to identify high risk patients; using risk score models is a very useful predictive utility such as the well-validated GI Complications Score (GICS). Leucocytosis and metabolic acidosis are also sensitive indicators for early diagnosis and prompt intervention. Developing a common strategy in collaboration with the bowel surgeons might be helpful to achieve that goal.

## A82 Surgery for cardiac myxoma: 21-year outcomes analysis from a single centre in United Kingdom

### Maria Comanici; Fabio De Robertis; Jullien Gaer; John Dunning; Toufan Bahrami; Sunil Bhudia; Shahzad Raja

#### Harefield Hospital, London, UK

**Correspondence**: Maria Comanici

*Journal of Cardiothoracic Surgery 19(1):* A82

**Objectives:** The outcomes of surgical treatment of cardiac myxoma in the modern era from the United Kingdom have not been recently reported. We performed this study to review our 22-year institutional experience of surgical management of cardiac myxoma.

**Methods:** We retrospectively analysed prospectively collected data from the Patients Analysis and Tracking System database (Dendrite Clinical Systems, Oxford, UK) for all cardiac myxomas excised at our institution from January 2001 to September 2022. In addition, medical notes and electronic patient records of all the study patients were reviewed.

**Results:** A total of 78 patients (35 males, 43 females, mean age 61.2 ± 13.1, range 28–83 years) underwent surgical resection of a cardiac myxoma during the study period. The tumour was located in the left atrium in 73 patients (93.6%), right atrium in four patients (5.1%), and right ventricle in one patient (1.3%). The tumour was incidentally diagnosed in 25 asymptomatic patients (32.1%) with 27 patients presenting with dyspnoea (34.6%), eight with palpitations (10.3%), seven with heart failure (9%), six with neurological events (7.7%), three with syncope (3.8%), and two with fever of unknown origin (2.6%). Eighteen patients (23.1%) had concomitant coronary artery bypass grafting (CABG) or valvular procedures (6 CABG, three aortic, three mitral, four tricuspid, and two CABG+ valve). Excision was performed through median sternotomy in 71 patients (91%) with left atriotomy as the preferred approach (65.4%). There were five in-hospital deaths (6.4%) with 4 deaths in patients having additional procedures (80%). Two patients (2.6%) required haemofiltration, 17 developed new-onset atrial fibrillation (21.8%), and one needed permanent pacemaker (1.3%). There were no recurrences during the study period.

**Conclusions:** Surgical excision of isolated myxoma is safe with excellent outcomes. Presence of concomitant coronary artery disease or valvular disorder requiring intervention increases mortality risk.

## A83 Post-operative protamine infusion does not lead to reduced bleeding or transfusion following coronary artery bypass grafting

### Ahmed Shazly; Hasnat Khan; Sudhir Bhusari; Arvind Singh; Mohamed Osman; Alessia Rossi; Alberto Albanese; Younus Qamar

#### Essex Cardiothoracic Centre, Basildon, Basildon University Hospital, UK

**Correspondence**: Ahmed Shazly; Younus Qamar

*Journal of Cardiothoracic Surgery 19(1):* A83

**Objectives:** Post-operative bleeding remains a significant risk after cardiac surgery. Despite adequate protamine reversal of heparin intraoperatively, protein-bound heparin causes anticoagulant effect, leading to bleeding in the post-operative period. The aim of this study is to whether the use of a four-hour, low dose protamine infusion in intensive care would reduce post-operative bleeding and hence, blood transfusion requirements.

**Methods:** A retrospective cohort study of seven hundred and two patients, who underwent elective or urgent coronary artery bypass grafting from April 2014 and January 2017, were divided into two groups based on who received post-operative protamine infusion (Group A, 472 patients) versus those who did not (Group B, 230 patients). They were assessed for amount of post-operative mediastinal and pleural drainage for the first 24 h, use of post-operative transfusion of blood products, postoperative hospital stay, and re-exploration.

**Results:** We found no significant difference between the rate of bleeding in either of the groups. No significant difference was observed in blood product requirements as well. In the sub-group consisting of patients with high BMI (BMI ≥ 30), who received protamine infusion, post-operative platelets transfusion was found to be significantly less.

**Conclusions:** Our results suggest that a low dose protamine infusion given in the immediate postoperative period does not lead to any significant clinical benefits. Both patients receiving and not receiving the infusion had similar postoperative drainage, transfusion requirements, haemorrhagic morbidity, mortality and length of hospital stay (Table 1).

**Table 1:** Comparison between the two groups in total drainage and transfusions requirements

	Group A protamine	Group B no protamine	P-value
Number	203	75	
Total Drainage (mean (SD)	392.26 (277.50)	403.51 (209.49)	0.75
Blood transfusion in units (mean (SD)	0.65 (1.83)	0.48 (0.86)	0.439
FFP transfusion (mean (SD)	0.39 (1.21)	0.61 (1.36)	0.197
Platelets (mean (SD)	0.11 (0.42)	0.27 (0.70)	0.023
Indexed blood loss (mean (SD)	11.54 (8.50)	12.27 (6.76)	0.507

## A84 Exploring the changing clinical characteristics of infective endocarditis requiring surgical intervention during the Covid-19 pandemic

### Gráinne Keehan; Jack Whooley; Alan Soo

#### Galway University Hospital, Galway, Ireland

**Correspondence**: Gráinne Keehan

*Journal of Cardiothoracic Surgery 19(1):* A84

**Objectives:** Knowledge on the risk factors, modes of presentation and the microbiology of infective endocarditis (IE) remains central in risk stratification and triage of patients with infective endocarditis down the appropriate management pathway.

The aim of this study was to examine whether any significant differences existed between the clinical characteristics, bacteriology and post-operative outcomes in patients with confirmed IE requiring surgical intervention presenting around or after the onset of Covid-19 when compared to those presenting before the onset of Covid-19 pandemic.

**Methods:** This single-centre retrospective observational study was conducted between the time period of June 2016 and June 2022. Patients that met the study inclusion criteria (n = 51) were stratified into those that underwent surgery for IE between June 2016 and June 2019, and those that underwent surgery for IE between July 2019 and June 2022. Comparison between the two groups was then performed using the chi squared/fishers exact test, or two sample t-test where appropriate.

**Results:** Patients that required surgical intervention between July 2019-June 2022 tended to be older (mean age 68.5 vs. 59.86, p = 0.031), and have a higher cardiac operative predicted mortality (mean EuroSCORE II 10.53 vs. 7.33, p = 0.036) than those presenting between June 2016-June 2019. Presence of risk factors and microbiology did not differ significantly between the two groups. Despite the higher predicted operative risk and more prevalent post-operative AKI requiring renal replacement therapy (6 vs 0, p = 0.006) in the July 2019-June 2022 group, there was no significant difference seen between 30-day mortality rates in the two groups (14.29% vs. 9.09%).

**Conclusions:** This study provides an early insight into the changing characteristics and resultant post-operative course of patients with confirmed infective endocarditis managed surgically following the onset of Covid-19.

## A85 Acute stroke following cardiac surgery: a 16-year analysis of 24,618 patients in a large UK tertiary centre

### Mai Shehab^1^; Eduardo Urgesi^2^; Mahmoud Shehab^3^; Marsioleda Kemberi^4^; Ahmed Eldarwi^5^; Nada Al Yasen^4^; Bijendra Patel^6^; Wael I. Awad^6^

#### ^1^Queen Mary University of London, King's College London Medical School, London, UK; ^2^Barts and The London, University of London, London, UK; ^3^Hull York Medical School, York, UK; ^4^Barts and the London Medical School, Bart's Heart Centre, London, UK; ^5^Barts Heart Centre, London, UK; ^6^Barts Health NHS Trust, London, UK

**Correspondence**: Mai Shehab

*Journal of Cardiothoracic Surgery 19(1):* A85

**Objectives:** To quantify the incidence of stroke following cardiac surgery over a 16-year period and to evaluate the potential risk factors leading to post-operative stroke in these patients.

**Methods:** Data was collected from 24,618 patients undergoing cardiac and aortic surgery at our centre between Jan 2005 and Dec 2021. Patients who developed a post-operative stroke were compared to those who did not. Univariate logistic regression analyses were applied to identify independent risk factors for the occurrence of post-operative stroke.

**Results:** 353 (1.43%) patients developed post-operative stroke. Major aortic surgery resulted in stroke in 44/340 (12.9%), isolated valve surgery in 120/5727 (2.1%) and isolated CABG in 108/13249 (0.8%) patients. After adjusting for possible confounders, the following were significant risk factors for development of stroke: CPB time > 140 min (OR = 3.78, 95% CI = 1.80–7.94; *P* < 0.001), dramatically increasing at 200 min to an OR = 9.03, 95% CI = 4.45–18.31; *P* < 0.001), aortic cross clamp time > 120 min (OR = 2.49, 95% CI = 1.57–3.96; *P* < 0.001), extracardiac arteriopathy (OR = 1.51, 95% CI = 1.06–2.14; *P* = 0.023), angina class III/IV (OR = 1.42, 95% CI = 1.03–1.97; *P* = 0.033), pre-operative inotropic use (OR = 3.01, 95% CI = 1.78–5.09; *P* < 0.001), poor LVEF (OR = 1.65, 95% CI = 1.07–2.52; *P* = 0.022), urgent (OR = 1.45, 95% CI = 1.12–1.89; *P* = 0.005), emergency (OR = 4.87, 95% CI = 3.56–6.68; *P* < 0.001) and salvage procedures (OR = 7.88, 95% CI = 4.18–14.87; *P* < 0.001), IABP use (OR = 2.98, 95% CI = 1.87–4.73; *P* < 0.001). Patients who return to theatre for post-operative bleeding or required new haemodialysis also had higher post-operative stroke (OR = 2.87, 95% CI = 1.97–4.19; *P* < 0.001) and (OR = 3.97, 95% CI = 2.96–5.32; *P* < 0.001), respectively.

**Conclusions:** The incidence of stroke following cardiac surgery is low with many predisposing factors. Nevertheless, identifying patients at increased risk of stroke may allow shared decision making between clinicians and patients and enhance informed consent.

## A86 Huge left atrial myxoma on CTPA clinically presenting as pulmonary embolism

### Abdul Nadim Asil; Youssef Abouelela; Ahmed Shazly; Mohamed Osman; Maria Macaroni; Alberto Albanese; Charina Mammino

#### Essex Cardiothoracic Centre, Basildon University Hospital, UK

**Correspondence**: Abdul Nadim Asil

*Journal of Cardiothoracic Surgery 19(1):* A86

**Introduction:** Atrial myxomas represent the most common benign primary heart tumor with an incidence of 3 in 1000 patients. These tumors can increase both morbidity and mortality. The majority of atrial myxomas occur in the left atrium, familial myxoma syndromes are reported to happen in atypical locations. Around 50% of cases with myxomas may experience symptoms due to intracardiac obstruction, peripheral or central embolism.10% of these patients may be completely asymptomatic.

**Case Presentation:** A 76-year-old lady presented to the GP with shortness of breath and typical manifestation of pulmonary embolism. A computed tomography pulmonary angiogram (CTPA) revealed a large filling defect in the left atrium and a subsequent transthoracic echo confirmed the diagnosis of a large left atrial myxoma with attachment to the base. An intraoperative TOE highlights attachment to base. She was managed with a surgical approach via median sternotomy trans-septal approach.

**Discussion:** There are multiple modalities for diagnosing left atrial myxomas however the transoesophageal echo shows 100% sensitivity. We believe a CTPA scan has adequate sensitivity in detecting atrial myxomas with the additional advantage of being non-invasive.

**Conclusions:** We believe that although the transoesophageal echo is the best modality for diagnosis of left atrial myxomas showing a sensitivity of 100%, other radiological modalities like CTPA can be extremely useful for differentiating atrial myxomas from pulmonary embolism as discussed in the case with the added benefit of being a non-invasive modality.

This is to confirm that the patient gave explicit permission to publish their information and images in an open access journal.
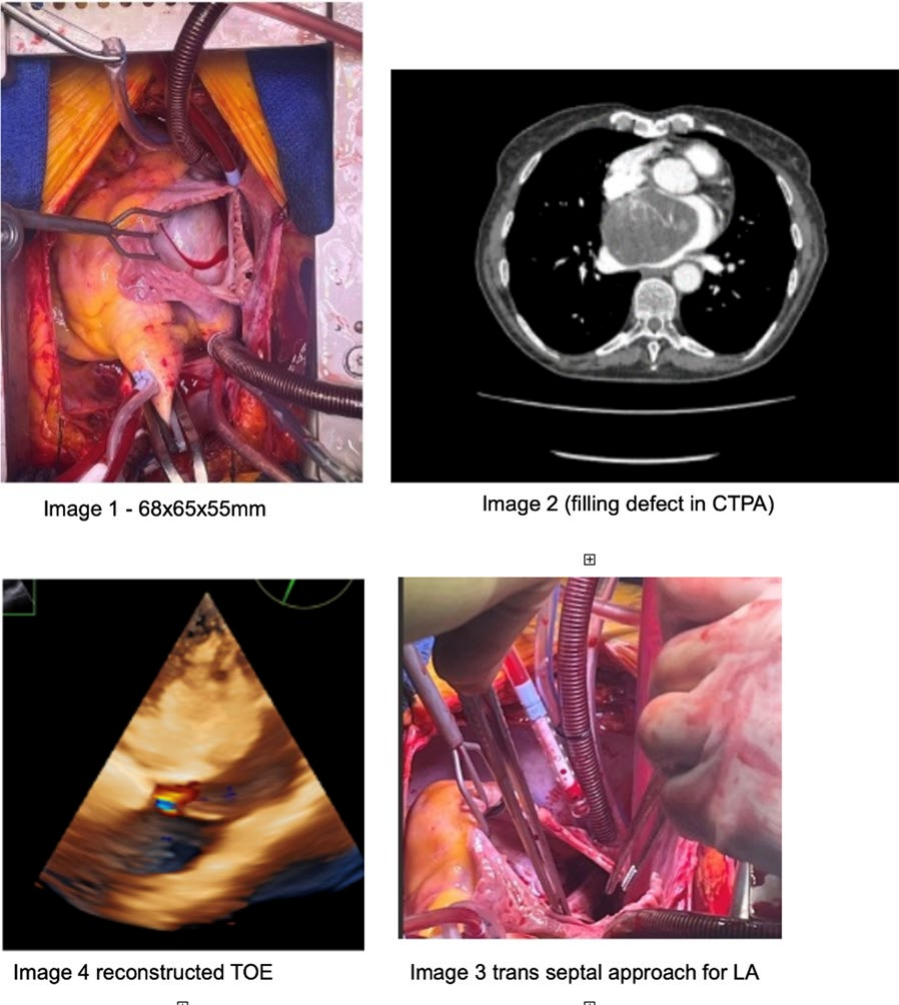


## A87 Epidemiological trends in infective endocarditis in an Irish population during the Covid era

### Gráinne Keehan; Jack Whooley; Alan Soo

#### Galway University Hospital, Galway, Ireland

**Correspondence**: Gráinne Keehan

*Journal of Cardiothoracic Surgery 19(1):* A87

**Objectives:** The aim of this study was to examine the distribution, determinants and microbiology of infective endocarditis in an Irish population, and explore whether any changes have been seen in these trends since the onset of the Covid-19 pandemic.

**Methods:** This single-centre retrospective observational study was conducted between the time period of June 2016 and June 2022. Patients that met the study inclusion criteria (n = 115) were stratified into those presenting with definitive infective endocarditis between June 2016 and June 2019, and those with confirmed infective endocarditis that presented between July 2019 and June 2022. Comparison between the two groups was then performed using the chi squared/fishers exact test, or two sample t-test where appropriate.

**Results:** Patients presenting with infective endocarditis during the July 2019-June 2022 period were noted to be older (*mean age 72.14 versus 66.54, p* = *0.021*), but more likely to require surgical intervention (*48.28% vs 38.59%)* when compared to those presenting between June 2016-June 2019. A statistically significant increase in the proportion of patients with aortic valve endocarditis was seen around or after the onset of Covid-19 (77.57% vs 59.65%, p = 0.037). This occurred is associated with a reduction in the proportion of patients presenting with mitral valve endocarditis *(31.52% vs. 15.51%, p* = *0.041).* With regards to implicated organisms, viridans streptococci were the most common causative agent in both groups, followed by staphylococcus aureus. All cases of coagulase negative staphylococci occurred in those with native valve endocarditis, with a p value of 0.0482 observed when performing a fishers exact test for coagulase-negative staphylococci native vs. prosthetic valve IE.

**Conclusions:** This study provides an early insight into the evolving epidemiology of infective endocarditis following the onset of Covid-19.

## A88 Minimally invasive surgical approach to left atrial ablation (convergent procedure) with left atrial appendage clip for persistent atrial fib

### Kathryn Mulryan^1^; James O Connor^2^; Jonathan Lyne^2^; Karen Redmond^2^

#### ^1^Mater Misericordiae University Hospital, Dublin, Ireland; ^2^Beacon Hospital, Dublin, Ireland

**Correspondence**: Kathryn Mulryan

*Journal of Cardiothoracic Surgery 19(1):* A88

**Objective:** Atrial fibrillation (AF) is a cause of structural heart disease, stroke, with increased hospitalizations. Patients can require multiple procedures and anti-arrhythmics to maintain sinus rhythm (SR). Patients with AF refractory to pharmacological and catheter ablations can be offered the Convergent Procedure. Traditional endocardial ablation is combined with a staged epicardial ablation via a sub-xiyphoid direct approach for radiofrequency lesion placement on the posterior left atrial wall. Left atrial appendage (LAA) occlusion is achieved with clip application (AtriClip LAA exclusion system, AtriCure, USA).

**Methods:** Data was gathered retrospectively between 2019 and 2022 from a single-surgeon database. The primary endpoint was freedom from AF post procedure and at 3-months. Secondary endpoints included 30-day mortality and complications.

**Results:** 13 patients underwent the Convergent/LAA clip procedure. All patients had long standing persistent AF, and an arrythmia burden of 7.23 years (2–19). 76.9% (9/13) of patients had freedom from AF post-surgery, with 53.8% in SR at three month follow up. 30-day mortality 0%. Post-operative complications requiring prolonged admission or re-admission included chyle leak (1) and pericardial tamponade (1). These resolved with administration of low-fat diet with octreotide and pericardotomy respectively.

**Conclusions:** Convergent procedure with LAA ligation is a safe and effective procedure and could be offered to patients with persistent AF and / or who have contraindications to anticoagulation.

## A89 Incidence and outcomes of surgical pulmonary embolectomy in the UK

### Amerikos Argyriou; Hunaid Vohra; Eltayeb Mohamed Ahmed; Cha Rajakaruna; Gianni Angelini; Daniel Fudulu

#### Bristol Heart Institute, Bristol, UK

**Correspondence**: Amerikos Argyriou

*Journal of Cardiothoracic Surgery 19(1):* A89

**Objectives:** Surgical pulmonary embolectomy is rarely used for the treatment of massive acute pulmonary embolism. The aim of our study was to assess the incidence and outcomes of this operation by retrospectively analysing a large UK registry (Fig. 1).

**Fig. 1:**
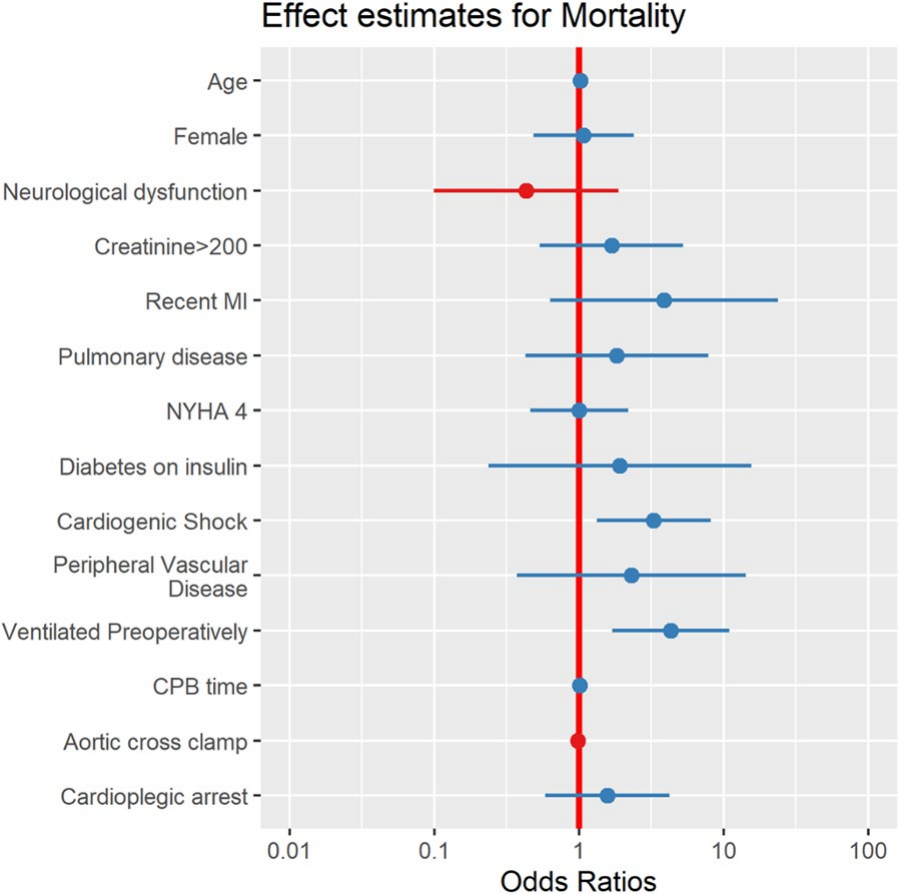
Forrest plot of surgical patient characteristics and peri-operative variables with relation to in-patient mortality

**Methods:** All UK acute pulmonary embolectomies performed between 1996 and 2018 were captured in the National Institute of Cardiovascular Outcomes Research (NICOR) database. The analysis was conducted in R Version 1.4.1106, packages: gt summary, and sj Plot. Categorical variables were summarised as counts with percentage. Continuous variables were summarised as median values with interquartile range. Multivariable logistic regression was performed to identify risk factors for in-hospital mortality.

**Results:** We identified 256 (0.039%) patients who underwent surgical pulmonary embolectomy out of 651,970 cardiac surgical procedures during the study period. At presentation, median age was 60, 56% of patients were male, 48% had NYHA class four heart failure symptoms and 38% had preoperative cardiogenic shock. Cardioplegic arrest was used in 53% of cases, with a median bypass time of 73 min and median cross-clamp time of 19 min. Median length of stay was 11 days. In-hospital mortality was 24%, post-op stroke was 5.9%, post-op dialysis required in 14% while the re-operation rate was 7.4%. Risk-adjusted multivariate analysis revealed cardiogenic shock (OR 1.2 CI 0.29–2.1, P = 0.01,), pre-op ventilation (OR 1.5 95%CI 0.54–2.4, P = 0.002,), bypass time (OR 0.01 CI 0.00–0.02, P < 0.001,) and cross-clamp time (OR − 0.02 CI − 0.03 to 0.00, P = 0.043) as significant independent risk factors for in-hospital mortality.

**Conclusions:** Surgical pulmonary embolectomy is associated with significant in-hospital mortality and morbidity. To our knowledge, this work represents the largest reported study in Europe. Preoperative ventilation, cardiogenic shock and increased by-pass and cross clamp times were significant predictors for in hospital mortality.

## A90 Bayesian networks via machine learning help identify determinants of short-term outcomes following cardiac surgery in a UK population

### Khurum Mazhar^1^; Bazegha Qamar^1^; Akshay Patel^2^; Ali Mohamed^1^; Mohsin Uzzaman^1^; Lognathen Balacumaraswami^1^; Marko Raseta^3^

#### ^1^Royal Stoke Hospital, Stoke on Trent, UK; ^2^University of Birmingham, Birmingham, UK; ^3^Erasmus MC Dept. of Molecular Genetics, Rotterdam, Netherlands

**Correspondence**: Khurum Mazhar

*Journal of Cardiothoracic Surgery 19(1):* A90

**Objectives:** Traditional risk stratification tools do not describe the complex causal relationships that exist amongst pre-operative and peri-operative factors and their influence on cardiac surgical outcomes. Our study reports on the use of Bayesian networks to investigate such outcomes.

**Methods:** Data were prospectively collected from 4776 adult patients undergoing cardiac surgery at a single UK institute between April 2012 and May 2019. Machine learning techniques were used to construct Bayesian networks for four key short-term outcomes: death, stroke, re-sternotomy for bleeding and renal failure.

**Results:** Duration of operation was the most important determinant of death (status at discharge) irrespective of EuroSCORE (Fig. 1). Duration of cardiopulmonary bypass was the most important determinant of re-operation for bleeding. EuroSCORE was predictive of new renal replacement therapy but not mortality. Univariate regression analysis was used to show the Markov Blanket from our initial results was indeed statistically significantly associated with all outcomes of interest.

**Fig. 1:**
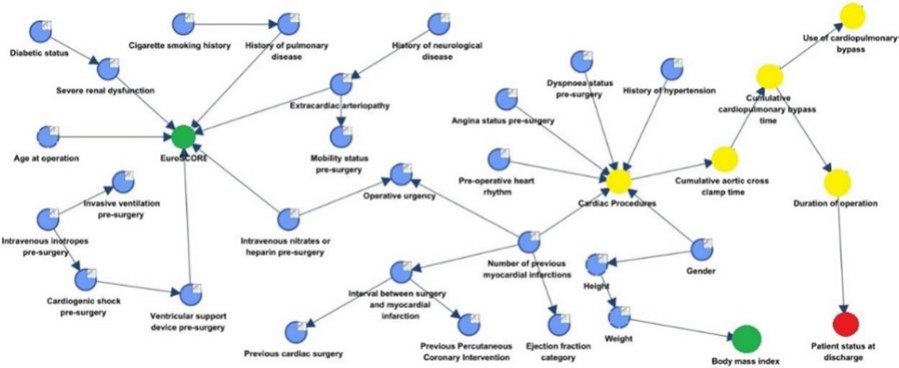
Bayesian network graph with MB for survival (status at discharge)

**Conclusions:** Machine-learning algorithms have allowed us to analyse the significance of dynamic processes that occur between pre-operative and peri-operative elements. Length of procedure and duration of cardiopulmonary bypass predicted mortality and morbidity in patients undergoing cardiac surgery in the UK. Bayesian networks can be used to explore potential causal mechanisms underlying outcomes and be used to help develop future risk models.

## A91 Designing an infection risk prediction tool for coronary artery bypass graft (CABG) surgery: what mapping the patient pathway tells us

### Lyn Brierley-Jones^1^; Judith Tanner^1^; Melissa Rochon^4^; Catherine Wloch^2^; Nigel Westwood^3^; Ricky Vaja^4^; Luke Rogers^5^; Gavin Murphy^6^; Theresa Lamagni^2^; Colin Brown^2^; Pauline Harrington^2^; Jeremy Dearling^3^; Keith Wilson^7^; Rosalie Renamagboo^8^; Sue Page^6^; Hardeep Aujla^6^; Penny Whiting^9^; Maureen Morgan^7^; Louise Bailes^5^; Fiona Fagan^4^; Angila Jawarchan^4^; Bil Kirmani^7^; Sunil Bhuddia^4^; Mario Petrou^4^; Cha Rajakaruna^5^

#### ^1^University of Nottingham, Nottingham, UK; ^2^UKHSA; ^3^PIP; ^4^Guys and St Thomas’ NHS Trust; ^5^University Hospitals Bristol and Weston NHS Trust, Bristol, UK; ^6^University of Leicester, Leicester, UK; ^7^Liverpool Heart and Chest Hospital NHS Trust, Liverpool, UK; ^8^Barts Health NHS Trust, London, UK; ^9^University of Bristol, Bristol, UK

**Correspondence**: Lyn Brierley-Jones

*Journal of Cardiothoracic Surgery 19(1):* A91

**Objective:** This NIHR programme development grant study aimed to identify candidate predictors for a surgical site infection risk prediction tool and strategies to target both infection prevention and surgical site infection (SSI) surveillance in adult cardiac surgery. The study included three work packages. This paper describes the work package designed to identify barriers and facilitators to implementing interventions that reduce the risk of SSI.

**Methods:** A qualitative study involving observations of 32 CABG patients at four cardiac centres as they progressed through the surgical pathway from admission, through theatres and until arrival on the post-surgical ward. Eligible patients were recruited from elective and urgent patient lists. Emergency cases were excluded. Sixteen semi-structured staff interviews (four per centre) and five patient interviews (one per centre plus a pilot interview) explored risk reduction implementation. Data were thematically analysed using the Consolidated Framework for Implementation Research (CFIR). Ethics approval was obtained from the East of Scotland Research Ethics Service (EoSRES).

**Results:** Findings indicate consistent adherence to some interventions to prevent SSIs and lack of adherence to others. Non-compliance was seen at the level of the individual and also by professional groups. Contextual factors influencing non-compliance included insufficient supplies (sutures, dressings) believed to be caused by Brexit, infrastructure funding (theatre size, condition), staffing levels and theatre culture (including power and hierarchy).

**Conclusions:** Facilitators and barriers to implementation involved all five CFIR domains. Risk responsibility, clustered (non) compliance and theatre culture were observed to influence the implementation of interventions to reduce the risk of SSI and warrant further investigation.

## A92 Barriers and facilitators to surgical site infection (SSI) surveillance in cardiac surgery in England: findings from surveys and interviews

### Lyn Brierley-Jones^1^; Judith Tanner^1^; Melissa Rochon^4^; Catherine Wloch^2^; Nigel Westwood^3^; Ricky Vaja^4^; Luke Rogers^5^; Gavin Murphy^6^; Theresa Lamagni^2^; Colin Brown^2^; Pauline Harrington^2^; Jeremy Dearling^3^; Keith Wilson^7^; Rosalie Renamagboo^8^; Sue Page^6^; Hardeep Aujla^6^; Penny Whiting^9^

#### ^1^University of Nottingham, Nottingham, UK; ^2^UKHSA; ^3^PIP; ^4^Guys and St Thomas’ NHS Trust, London, UK; ^5^University Hospitals Bristol and Weston NHS Trust, Bristol, UK; ^6^University of Leicester, Leicester, UK; ^7^Liverpool Heart and Chest Hospital NHS Trust, Liverpool, UK; ^8^Barts Health NHS Trust, London, UK; ^9^University of Bristol, Bristol, UK

**Correspondence**: Lyn Brierley-Jones

*Journal of Cardiothoracic Surgery 19(1):* A92

**Background:** Whilst national surveillance and audit programmes for cardiac surgery are in place in England, participation is variable with all 29 cardiac centres reporting deep sternal wound data to NICOR, 18 participating in the UKHSA SSI CABG module and eight submitting to the GIRFT SSI audit. As part of an NIHR funded programme development grant to develop an SSI risk prediction tool and increase surveillance, the barriers and enablers to SSI surveillance were explored.

**Methods:** Surveillance and cardiac consultant leads at all cardiac centres in England were asked to complete a surveillance awareness survey and distribute a survey to staff assessing perceptions of barriers and enablers to surveillance. All responders were invited to be interviewed. Data were analysed using descriptive statistics or thematically.

**Results:** All 29 centres responded to the surveillance survey with responses provided by surveillance staff (17) or by surgeons (12). In addition, barrier and enabler surveys were completed by 93 staff, 16 of which were interviewed.

Combined survey and interview data suggests a division between UKHSA and NICOR by professional groups, where UKHSA is seen to be led by surveillance staff while NICOR is seen as surgeon led, with little integration but some duplication. NICOR was embedded within the clinical pathway with surgeons collecting data as part of their daily activities. UKHSA surveillance was seen as more removed from the clinical pathway as data was collected by surveillance staff. Both systems were thought to have strengths and weaknesses.

Key facilitators for surveillance were reported as dedicated surveillance staff (88%), senior management buy in (63%) and surgeon champions (62%). Reported barriers included lack of resources, lack of standardised definitions and an unsupportive culture.

**Conclusions:** Interventions to maximise SSI reporting and engagement should be tailored to specific programmes.

## A93 10 year review of tricuspid valve repair outcomes using suture annuloplasty (P-repair)

### Nikhil Sahdev; Riccardo Abbasciano; Hashem Abdelkader; Rebecca Turton; Jacob Chacko; Muhammad Ashraf; Vinci Naruka; Guiqing Liu; Prakash Punjabi

#### Hammersmith Hospital, London, UK

**Correspondence**: Nikhil Sahdev

*Journal of Cardiothoracic Surgery 19(1):* A93

**Objectives:** Tricuspid Regurgitation (TR) is often a concomitant finding in patients undergoing cardiac surgery. This study evaluates the safety, effectiveness and mid-to-long-term outcomes of tricuspid valve repair performed via interrupted pledgeted sutures (P-repair) without using a conventional prosthetic ring.

**Methods:** Patients over a 10-year period receiving P-repair (6–10 pledgeted sutures as required to achieve optimal tricuspid annular reduction and competent repair) were compared with patients undergoing tricuspid repair using conventional ring annuloplasty. A propensity-matched analysis was performed to assess differences in perioperative morbidity, mortality, long-term survival and echocardiogram findings.

**Results:** 196 patients (average follow-up:1514 days) were included. Both groups were statistically similar in age, diabetes, baseline creatinine, ejection fraction and pulmonary artery systolic pressure. P-repair patients were significantly more symptomatic (NYHA 3) (59(70%) vs 39(35%)) (p < 0.001). Rate of concomitant surgical procedures i.e. CABG (p = 0.886), aortic (p = 0.890) and mitral valve interventions (p = 0.586) was comparable between the two groups. No statistical difference was observed in rate of re-operation (p = 0.157), haemodialysis (p = 0.068) and pacemaker insertion (p = 0.726). Propensity-matched scoring did not reveal any statistical difference in hospital mortality (p = 0.293). No statistical difference was observed in long term survival on Kaplan–Meier analysis (p = 0.580). Echocardiography review at baseline (p = 0.369) and follow up (p = 0.55) showed no difference in presence and severity of TR. Significant difference was found in the cost analysis between the two groups: prosthetic ring ($1015) vs p-repair ($30).

**Conclusions:** P-repair is comparable to conventional ring annuloplasty in terms of safety profile and long-term results but importantly is considerably cheaper and easier to perform. We recommend widespread adoption of this safe, effective and low-cost technique (Fig. 1).

**Fig. 1:**
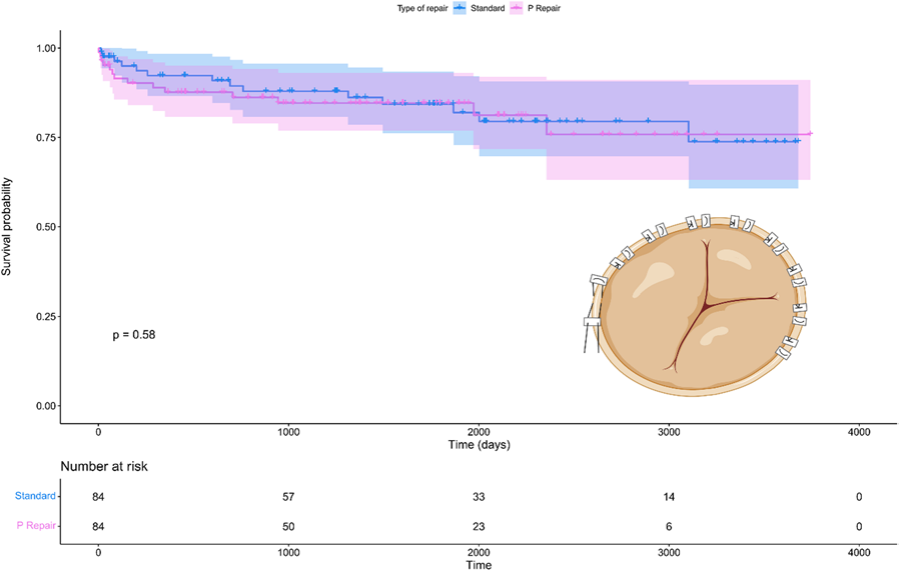
No statistical difference was observed in long term survival on Kaplan–meier analysis in both the matched and unmatched cohorts (p = 0.580)

## A94 Medium- and long-term follow-up of super obese patients after cardiac surgery: a ten years series from a single centre

### Ammar Mustafa; Jeni Palima; Michael Boateng; Hannah Jesani; Giuseppe Rescigno

#### Royal Wolverhampton NHS Trust, Wolverhampton, UK

**Correspondence**: Ammar Mustafa

*Journal of Cardiothoracic Surgery 19(1):* A94

**Objective:** Obesity is a worldwide pandemic that carries a significant burden for the National Health Services. The operative risk for obese patients in Cardiac Surgery is not completely elucidated. In a previous analysis performed on patients with a BMI =  > 40 kg/m^2^ operated in our Department from January 2010 to December 2019 we have shown that hospital results were not different from the EuroSCORE two predicted mortality. However, the long-term benefit in terms of quality of life is also matter of debate. Therefore, we performed a telephone follow-up to assess the clinical condition among the same population. Here we present our results.

**Methods:** Our initial cohort included 179 patients. Mean age was 61.2 ± 9.4 years; mean SCTS EuroSCORE was 1.6 ± 4.1. The types of operations were isolated CABG (45.8%), isolated valve surgery (34.0%), CABG + Valve (12.8%), major aortic (3.9%) and others (3.3%). There were 3 hospital deaths (1.6%). Therefore, the follow-up was attempted in the 176 survivors. Telephone calls were performed from December 2021 to June 2022. They were successful for 122 (89%) of the alive patients. The outcomes (alive or dead and date of death) for those who were not possible to contact, were obtained by using the NHS SPINE digital service.

**Results:** Thirty-two follow-up deaths were recorded. The actuarial survival is displayed in the Figure. Survival at 80 months was 85%. Clinical condition outcomes are summarised in the Table. Of the contacted patients 77% were in NYHA Class I or II. Five patients advised to have severe dyspnoea. Recovery time to normal life after surgery was 3.9 +−3.88 months.

**Table:** 

Recovery time from surgery (months)	3.9 3.88
NYHA Class	
I	70 (57.3%)
II	25 (20.4%)
III	22 (18.0%)
IV	5 (4.0%)
Angina (any severity)	4
Later admission for any cardiac condition	21 (17.2%)

**Conclusions:** This study shows that long-term results of cardiac surgery in super obese patients are good. Recovery time is acceptable and there is a good quality of live. The beneficial effects could be probably improved by appropriate weight loss programs. A propensity matching analysis with a similar population of non-obese patients is warranted.
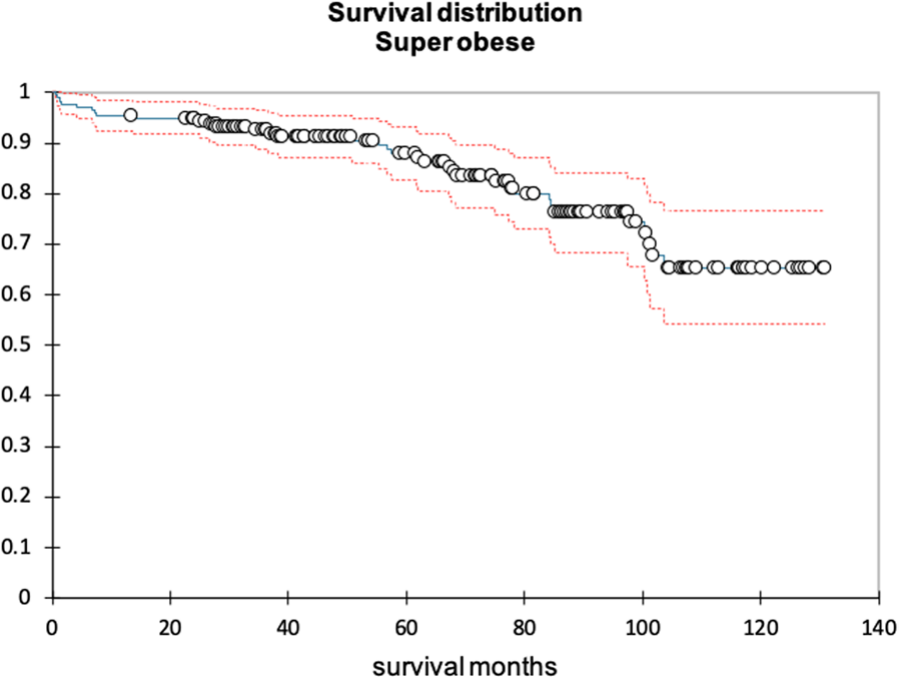


## A95 Left atrial appendage occlusion as a concomitant procedure in non-mitral cardiac surgery: a meta-analysis

### Lauren Kari Dixon^1^; Krishna Patel^2^; Sara Eltirafi^3^; Ettorino Di Tommaso^1^; Anil Sankanahalli Annaiah^1^; Sarah George^2^; Vito Domenico Bruno^1^

#### ^1^Bristol Heart Institute, Bristol, UK; ^2^University of Bristol, Bristol, UK; ^3^University of Cardiff, Cardiff, UK

**Correspondence**: Lauren Kari Dixon

*Journal of Cardiothoracic Surgery 19(1):* A95

**Objectives:** Left Atrial Appendage Occlusion (LAAO) during cardiac surgery is thought to reduce stroke incidence in patients with atrial fibrillation (AF). This meta-analysis aims to assess the benefits of concurrent LAAO in patients, with or without AF, undergoing non-mitral cardiac surgery.

**Methods:** A literature search was performed using PubMed, Web of Science and Cochrane Library. We included all studies comparing LAAO in non-mitral adult cardiac surgery. Our short-term outcomes were mortality, cerebrovascular accident (CVA), major adverse cardiovascular and cerebrovascular events (MACCE), postoperative AF (POAF) and readmission rate. Our mid-term outcomes were one-year mortality and thromboembolic events (TE). Odds Ratios (OR) and Confidence Intervals (CI) were created using the Mantel–Haenszel method.

**Results:** We reviewed 1,315 studies and included 17 studies with a total of 24,794 patients (9,742 LAAO, 15,052 control) in our final analysis. At 30-days, the LAAO cohort was associated with a lower incidence of CVA (OR = 0.88, 95% CI = 0.84, 0.93, p < 0.0001). The LAAO cohort had less one-year mortality (OR = 0.87, 95% CI = 0.81, 0.94, p = 0.0002) and TE (OR = 0.85, 95% CI = 0.76, 0.95, p = 0.005) compared to the control cohort. There was no difference in 30-day mortality (OR = 0.94, 95% CI = 0.68, 1.31, p = 0.72), MACCE (OR = 1.00, 95% CI = 0.84, 1.19, p = 0.97) or re-admission rate (OR = 0.70, 95% CI = 0.34, 1.44, p = 0.33). LAAO was associated with increased 30-day POAF (OR = 1.69, 95% CI = 1.17, 2.43, p = 0.005).

**Conclusions:** LAAO is a safe and effective concurrent procedure to non-mitral cardiac surgery to reduce short-term CVA incidence and mid-term mortality and TE. Despite an initial increase in POAF with LAAO, this does not translate to increased CVA or mortality. Further studies comparing long-term outcomes such as CVA and AF rates are required.

## A96 Intracranial haemorrhage in patients after pulmonary thromboendarterectomy: incidence and outcomes

### Rushmi Purmessur^1^; Winston Yao^2^; John Fouad Taghavi^1^; Choo Ng^1^; Steven Tsui^1^; David Jenkins^1^

#### ^1^Royal Papworth Hospital NHS Foundation Trust, Cambridge, UK; ^2^University of Cambridge Medical School, Cambridge, UK

**Correspondence**: Rushmi Purmessur

*Journal of Cardiothoracic Surgery 19(1):* A96

**Objective:** Pulmonary thromboendarterectomy (PTE) is the definitive treatment for chronic thromboembolic pulmonary hypertension (CTEPH). The procedure decreases pulmonary vascular resistance (PVR) and reduces right ventricle afterload, improving symptoms and prognosis. Patients require full anticoagulation in the early post-operative period to prevent thrombosis of the denuded pulmonary vasculature. However, a recent report on a small cohort of PTE patients suggested that 60% develop subdural haemorrhage (SDH) post-operatively (Yap et al. 2018). This alarmingly high figure has obvious implications for anticoagulation management. We sought to find out the incidence and outcomes of intracranial haemorrhage (ICH) in patients undergoing PTE at the UK national PTE centre.

**Methods:** This was a retrospective analysis of patients undergoing PTE at the UK national PTE centre from September 2020 to May 2022. Pre-operative, intra-operative and post-operative parameters as well as outcomes were analysed.

**Results:** During the study period, 13 out of 249 PTE patients suffered an ICH post-operatively, representing an incidence of 4.4%. Pre-operative, intra-operative and post-operative parameters for patients with and without post-operative ICH are summarised in Fig. 1. The most common presentation was headache and the commonest type of ICH confirmed by imaging was a SDH. 3 patients required emergency surgical management, while the rest were managed conservatively. The in-hospital mortality for affected patients was 18% (n = 2). Follow-up imaging performed in 8 patients showed stable or improved appearances, with a median follow-up time of 15 days.

**Conclusions:** The incidence of ICH is low in the early post-PTE period but this is a serious and potentially fatal complication. A high index of suspicion and early imaging allow early detection. The low incidence of this complication means that a much larger cohort of patient will need to be studied to identify potential risk factors.
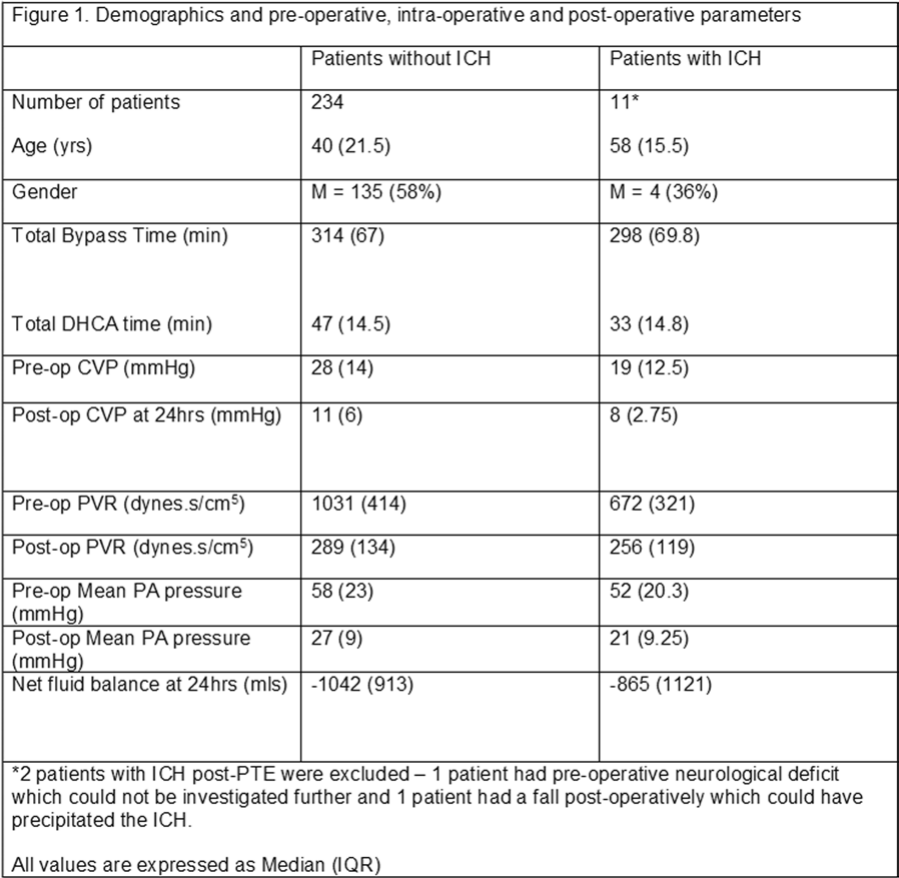


## A97 Outcomes of COVID-19 patients undergoing cardiac surgery during pandemic: ancestral variant compared to omicron variant

### Vinci Naruka; Selina Tsai; Martin Yates; Maria-Teresa Cutino-Moguel; Anna Riddell; Damian Balmforth; Ana Lopez-Marco; Aung Y. Oo; Rakesh Uppal

#### St. Bartholomew Hospital, London, UK

**Correspondence**: Vinci Naruka

*Journal of Cardiothoracic Surgery 19(1):* A97

**Objectives:** SARS-CoV-2 viral infection affected patient outcomes in cardiac surgery with significantly increased mortality during the initial outbreak. There is limited data on operative outcomes of vaccinated patients with PCR evidence of COVID-19 (CV19) infection in cardiac surgery. We report our outcomes for CV19 patients undergoing cardiac surgery and compared patients infected with ancestral variant to those with Omicron one.

**Methods:** A prospectively collated database was analysed for patients undergoing cardiac surgery with positive CV19 PCR within 3 days of their operation. Two cohorts were identified: cohort 1 (C1), operated in February to March 2020 during the ancestral variant outbreak, cohort 2 (C2) from September 2021 to August 2022 during the Omicron variant. Vaccination history was recorded. Samples were tested for SARS-CoV-2 RNA using different platforms with values as cycle threshold or relative light units. This made cycle thresholds and therefore mRNA load incomparable. The primary outcome was 30-day mortality. Secondary outcomes included stroke, atrial fibrillation, dialysis, resternotomy for bleeding and length of stay (LOS).

**Results:** Nine patients in C1 and 26 in C2 underwent cardiac surgery, mean age was 61 ± 17 years (C1) and 63 ± 11 years (C2). 88% were males in C1, 73% in C2. 88.9% were urgent/emergency cases in C1 and 65.4% in C2. C1 included: 44.4% coronary artery bypass graft (CABG) and 55.6% valves. In C2: 50% CABG, 34.6% valves, 11.5% aortic repairs and 3.8% pericardectomy. Table 1 summarises outcomes. Lower mortality (3.9% v 44.4%, p = 0.01) and LOS (8 v 14, p = 0.03) were reported in C2.

**Table 1:** Outcomes for COVID-19 patients undergoing cardiac surgery infected with Ancestral variant to Omicron one

Outcomes	Cohort 1 (n = 9) (February–March 2020)	Cohort 2 (n = 26) (September 2021–August 2022)
Vaccinated	0%	92% (23/25)
EuroSCORE II (median (IQR))	2.29 (1.03–4.91)	1.59 (0.88–2.86)
New post-operative stroke	0% (0/9)	7.7% (2/26)
New post-operative dialysis	22.2% (2/9)	3.9% (1/26)
New post-operative atrial fibrillation	0% (0/9)	15.4% (4/26)
New post-operative permanent pacemaker	11.1% (1/9)	0% (0/26)
Resternotomy for bleeding	11.1% (1/9)	3.9% (1/26)
Length of stay (days) (median (IQR))	14 (12–24)	8 (6–11.75)
30-day mortality	44.4% (4/9)	3.9% (1/26)

**Conclusions:** Despite similar risk profile and acuity, significantly lower mortality, morbidity and postoperative stay were observed in CV19 patients undergoing cardiac surgery in C2. It is likely that vaccination contributed to improved outcomes. Although rigorous CV19 protocols to reduce infection must continue, it is encouraging to see improved outcomes.

## A98 Percutaneous versus surgical closure of post-infarction ventricular septal defect: a single centre experience

### Alice Mines; Shahan Shahid; Azhar Hussain; Gentjan Jakaj; Irfan Rasheed; Ranjit Deshpande; John Byrne; Habib Khan

#### Kings College Hospital, London, UK

**Correspondence**: Alice Mines

*Journal of Cardiothoracic Surgery 19(1):* A98

**Objectives:** Post-infarction ventricular septal defect carries a poor prognosis. Although surgical repair is usually the first line treatment for post infarction ventricular septal defect, percutaneous options have become increasingly used in the acute setting. Our study looked at early outcomes in patients treated with either approach in a single UK centre.

**Methods:** Patients treated with either percutaneous or surgical repair between 2005 and 2021 were included in the study. Retrospective review of case notes was undertaken. The primary outcome was 30-day mortality. Patients were allocated into two groups based on their treatment modality (percutaneous vs surgical).

**Results:** Thirty-six patients were identified (31 surgery, 5 percutaneous). Average time from AMI to intervention were similar in both groups (10.1 vs 11 days, p = 0.22). Percutaneous patients were significantly older (66.7 vs 76.4, p = 0.048) and much more likely to be discussed in a formal MDT. There were no differences in in-hospital mortality between the two groups (58% vs 60%, p = 0.18). Repeat intervention was more commonly required in the surgical group (6% vs 0%, p = 0.12).

**Conclusions:** Both percutaneous and surgical treatments are viable management options in the setting of post infarction ventricular septal defects. Long term outcomes are still uncertain for percutaneous treatment options, but provide a useful treatment strategy in patients who may be unfit to undergo surgical repair with good short term outcomes.

**Table:** 

	Percutaneous (5)	Surgical (31)
Average Age	Mean—76.4 (61–84) Median—77	Mean—66.7 (45–82) Median—67
Risk Factors	DM—2/5 HTN—2/5 Lung disease—0/5 Smoking—1/5 CVA—0/5 Hyperchol—1/5	DM—6/31 HTN—24/31 Lung disease—6/31 Smoking—22/31 CVA—2/31 Hyperchol—4/31
Heart MDT	Formal—5/5 (100%) Informal—0/5	Formal—11/31 (35%) Informal—20/31 (65%)
Average Length of time between MI and VSD intervention	Mean 11 (5–21) Median 12	Mean 10.1 (1–37) Median 6
24-h Mortality	1/5 (cardiac causes intra-operatively) (20%)	4/31, (all cardiac causes, 1 intraoperatively) (13%)
In-Hospital Mortality	3/ 5 ? (60%) (2 MODS, 1 cardiac)	18/31 ? (58%) (6 MODS, 11 cardiac, 1 unknown)
30-day Stroke	0/5 (0%)	1/31 (3%)
30-day Patch Dehiscence	N/A	Partial 3/31 (10%) Complete 4/31 (13%)
Repeat Intervention	0/5 (0%)	2/31 (surgical closure for dehiscence) (6%)

## A99 Does routine outpatient post operative chest X-ray and electrocardiograph iin cardiac surgical patients make a difference and at what cost?

### Ali Mohamed^1^; Khurum Mazhar^1^; Zara Khan^1^; Bazegha Qamar^1^; Hosam Ahmed^1^; Marko Raseta^2^; Lognathen Balacumaraswami^1^; Prakash Nanjaiah^1^

#### ^1^Royal Stoke Hospital, Stoke on Trent, UK; ^2^Erasmus MC Dept. of Molecular Genetics, Rotterdam, Netherlands

**Correspondence**: Ali Mohamed

*Journal of Cardiothoracic Surgery 19(1):* A99

**Objectives:** There is variation of practice regarding follow up of post operative cardiac surgical patients, with a rise in the number of Remote Consultations (RC). Proponents of traditional ‘Face-to-Face’ method (F2F) cite the importance of post operative Chest X-Ray (CXR) and Electrocardiograph (ECG) in determining further management. We sought to determine: (1) if routine outpatient (OP) CXR and ECG following cardiac surgery determined further management and (2) the cost to the hospital and the patient of having these investigations.

**Methods:** Retrospective study of patients who underwent cardiac surgery between 1st April 2021–31st March 2022 at our institution. Inclusion criteria: All patients who had isolated coronary artery bypass graft (CABG), isolated aortic valve replacement (AVR) or CABG + AVR at first post-operative clinic follow-up. Exclusion criteria: duplicate appointments, death or re-admission prior to appointment.

**Results:** 333/450 patients met the eligibility criteria. 215 patients had F2F consultation with either CXR/ECG or both; results displayed in Fig. 1. 84.5% (n = 71) of RC were discharged and of the 15.5% offered follow-up 69.2% (n = 9) had no change in management with CXR and/or ECG. 30.8% (n = 4) had a further RC with no change in management. There was no difference in reporting of symptoms between RC and F2F (p = 0.95) but RC were more likely to be discharged after first follow up (p < 0.01). Mean cost of transportation was £4.50 per patient (excluding mileage costs) but mean duration of appointment was over 90 min.

**Fig. 1:**
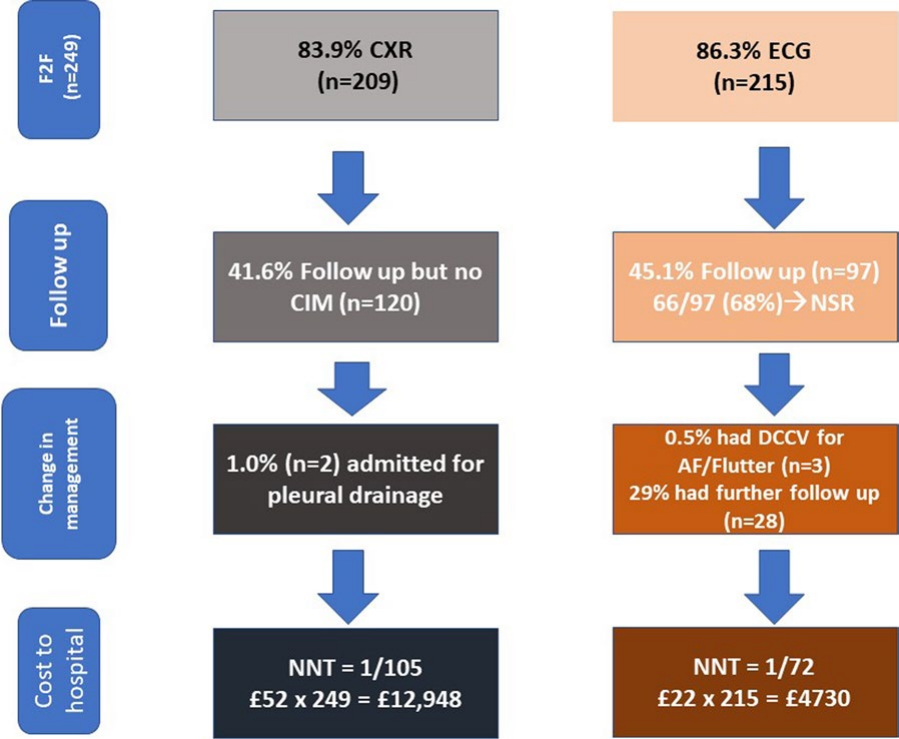
Results of F2F Consultation with CXR and/or ECG

**Conclusions:** This study shows that routine outpatient CXR and ECG following cardiac surgery does not alter management in the majority of patients. Furthermore, RC is cost effective from both a societal and health care perspective. We feel this topic merits further investigation to identify those patients most likely to benefit from a F2F appointment.

## A100 A 20-year experience of cardiac surgery and concomitant carotid endarterectomy

### Marcus Taylor; Paul Waterworth; Isaac Kadir; Rajamiyer Venkateswaran; Jim Barnard; Vipin Mehta

#### Wythenshawe Hospital, Manchester, UK

**Correspondence**: Marcus Taylor

*Journal of Cardiothoracic Surgery 19(1):* A100

**Objectives:** Patients with severe carotid artery disease undergoing cardiac surgery are at increased risk of peri-operative stroke. A proportion of these patients undergo combined cardiac surgery and carotid endarterectomy (CS-CEA) to try and mitigate the risk of stroke. The aim of this study was to review short and long-term outcomes of patients undergoing CS-CEA.

**Methods:** A single-centre retrospective review of all patients undergoing CS-CEA between September 2002 and September 2022 was undertaken. Patients undergoing emergency or salvage surgery were excluded. Primary outcomes were in-hospital mortality and overall survival. Secondary outcomes were incidence of peri-operative stroke and post-operative length of stay (PLOS). The impact of peri-operative stroke on survival was assessed using the log-rank analysis.

**Results:** A total of 20,204 patients underwent cardiac surgery over 20 years, out of which 0.5% (n = 102) had CS-CEA. Of those 102 patients the mean age was 67 years (SD ± 9.8) and 68% (n = 69) were male. Elective planned surgery was done in 58% (n = 59), while 42% (n = 43) underwent urgent surgery. 33% (n = 34) had left ventricular impairment. Concomitant cardiac surgery (with CEA) was coronary artery bypass grafting (CABG) in 58% (n = 59), valve surgery in 21% (n = 21) and valve & CABG in 22% (n = 22). Median additive and logistic EuroSCORE-I, were 7 (IQR 5–8) and 3.4 (IQR 1.8–7.1), respectively. In-hospital mortality was 3.9% (n = 4) and median PLOS was 8 days (IQR 6–11 days). The incidence of peri-operative stroke was 3.9% (n = 4). Median follow-up time was 43 months (IQR 16–108 months, range 0–240 months). Estimated median overall survival was 15 years (79–277 months). Peri-operative stroke was associated with reduced overall survival (log-rank analysis, p = 0.005) (Fig. 1).

**Fig. 1:**
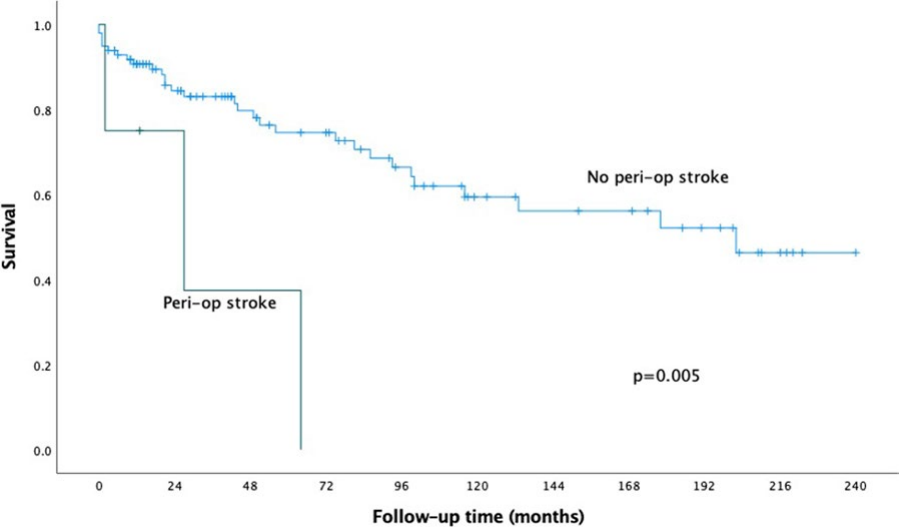
Kaplan–Meier curve comparing overall survival in patients who did and did not experience per -operative stroke after CS-CEA

**Conclusions:** This study has shown that in experienced centres, concomitant cardiac surgery and carotid endarterectomy can be performed safely with good short and long-term outcomes.

## A101 Changes in clinical practice with modified enhanced recovery after surgery (ERAS) programme in cardiac surgical patients

### Maria Rita Maccaroni; Youssef Abouelela; Ahmed Shazly

#### Essex Cardiothoracic Centre, Basildon, Basildon University Hospital

**Correspondence**: Youssef Abouelela

*Journal of Cardiothoracic Surgery 19(1):* A101

**Objectives:** ERAS is a worldwide programme implemented to improve clinical outcomes, cost effectiveness and patient satisfaction. Our aim is to assess whether implementing the ERAS guidelines to our practice in cardiac surgical patients would accelerate recovery and facilitate faster turnover, thereby reducing length of stay in ITU and hospital.

**Methods**: Data collection has been ongoing since 2021 at our cardiac tertiary centre and thus far includes 758 patients. We have included all patients scheduled for elective and urgent CABG and/or valve surgery. The exclusion criteria are any patients in heart failure, BMI > 35, poor respiratory function and end stage renal disease. To ensure adequate hydration, all patients received clear carbohydrate fluids. The anaesthetic technique is geared towards rapid recovery using short-acting anaesthetic and analgesic agents with bispectral index monitoring throughout. The final decision to continue the enhancement protocol is reassessed at the end of surgery based on haemodynamics and bleeding. Following this, the aim is to stop sedation one hour after arriving in ITU (if bleeding < 100 ml/h, normal TEG and acceptable ABG) and then extubate within six hours. The outcome measured was percentage of patients extubated within 6 h and length of stay in hospital.

**Results:** Before implementing the modified ERAS programme, only 40% of patients were extubated within 6 h and following these interventions this has significantly improved to 65%. The average ITU stay was 1.5 days with 72% of patients discharged home in less than a week. Only five out of the 758 patients were re-intubated within 12 h.

**Conclusions**: The modified ERAS has considerably transformed best practice in our unit. With appropriate protocols, training and regular audits, this protocol can be adapted in other cardiac units to support fast-tracking patients, enhance recovery and patient satisfaction.

## A102 Impact of implementation intra-operative haemostasis checklist to reduce re-operation rates for bleeding and blood transfusion after cardiac surgery

### Sobaran Sharma; Yasir Ahmed; Sam Poon; Umair Aslam; Afzal Zaidi; Pankaj Kumar

#### Morriston Hospital, Swansea, UK

**Correspondence**: Sobaran Sharma

*Journal of Cardiothoracic Surgery 19(1):* A102

**Introduction:** Re-exploration for bleeding or tamponade is an adverse clinical outcome. National databases and the published literature reports a re-exploration rates after cardiac surgery in the range of 2% to 8%. We evaluated the impact of introducing an intra-operative haemostasis checklist prior to chest closure in our unit.

**Method:** Reducing the rates of re-exploration for bleeding following cardiac surgery has been a key priority for patient care in our unit. On 1st August 2021, Intraoperative haemostasis Checklist was introduced in a systematic manner. All consecutive patients undergoing cardiac surgery in the 14-month period, either side of the service change were included in the study. Our study compared the outcomes in Group A (pre-checklist) and Group B (post checklist). Prospectively collected data from the PATS registry and local databases were collated and compared.

**Results:** In Group A, n = 388 and Group B, n = 533 patients underwent cardiac surgery. The Groups were similar in the pre-operative demographics, risk profile by EuroSCORE II 3.81% vs 3.77% or case mix between Group A and Group B respectively.

There was 76% reduction in re-exploration of bleeding or tamponade with 2.3% vs 0.56% of patients in group A and B respectively. We also observed a marked reduction in the use of RBC and FFP transfused per patient in the ratio 3:1 and 2:1 respectively which represents a reduction of 66% and 55% respectively. There was no difference in the use of platelets between the two Groups. There was also a tendency towards lower blood loss in the first 12 h in ITU (Group A 399 mls and Group B 350 mls).

**Conclusions:** The implementation of a checklist has resulted in significant reduction in re-exploration rates for bleeding or tamponade and in addition there has also been a reduction in the blood transfusion requirements. Consideration should be given to a similar approach on a wider level in services delivering cardiac surgery to improve the clinical outcomes.

## A103 Bloodletting in post-operative cardiac surgical patients: a 5-year follow-up study

### Najeeba Lallmahomed; Muslim Mustaev; Paolo Bosco

#### St. Thomas' Hospital, London, UK

**Correspondence**: Najeeba Lallmahomed

*Journal of Cardiothoracic Surgery 19(1):* A103

**Objectives:** The most common blood tests requested in post-operative cardiac surgical patients include FBC, U&E, Coagulation Screen and CRP. An initial study carried out in 2017 at our centre demonstrated that a mean of 121 mL (SD = 45 mL) of blood per patient was drawn as part of post-operative investigations, with a mean length of stay of 8.8 days. A local recommendation was subsequently implemented, stating that stable patients should only be bled on Days 0, 1 and 5 in order to decrease the likelihood of iatrogenic anaemia. Our aim was to audit this recommendation after 5 years of implementation, and to evaluate its cost effectiveness.

**Methods:** Data was collected from 50 randomised cardiac surgical patients undergoing CABG and single valve replacements between the 1st June 2022 to the 1st August 2022, and only those whose post-op lengths of stay were between 5 to 15 days were included. Our data was analysed using the IBM SPSS Statistics software.

**Results:** A new mean of 90 mL (SD = 23 mL) was the amount of blood drawn from patients by venepuncture post-operatively (p = 0.025). There was a statistically significant drop in the total number of blood tests per patient (p = 0.022), with a mean length of stay of 7.2 days (p = 0.591). There was consequently a 6.9% reduction in the cost of blood tests.

**Conclusions:** Although the new local recommendation set five years ago was not being followed to the letter, there has been a statistically significant decrease in both the number of blood tests and the total amount of blood let, in addition to a decreased associated cost.

Comparative results

**Table:** 

	2022	2017	p
LENGTH OF STAY POST CARDIAC SURGERY, Days	7.2 ± 2.2	8.8 ± 2.9	0.591
NUMBER OF TESTS PER PATIENT	21.3 ± 5.5	28 ± 10	0.022
TOTAL BLOODLETTING PER PT, mL	89.7 ± 22.7	121 ± 44	0.025
FBC PER PT	7.2 ± 1.7	8 ± 2.7	0.753
U&E PER PT	7.5 ± 1.9	8 ± 3.0	0.903
CRP PER PT	4.9 ± 1.7	4 ± 2.3	0.847
COAGS PER PT	6.5 ± 2.4	7 ± 3.5	0.476


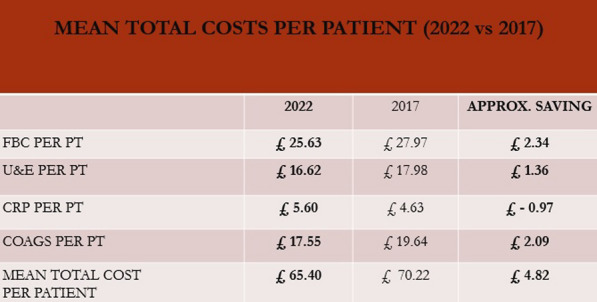


## A104 Reporting of postoperative complications in clinic letters after cardiac surgery: a complete loop audit

### William Crawford^1^; Vinci Naruka^1^; Sara Volpi^2^; Philip Hartley^1^; Martin Yates^1^

#### ^1^St Bartholomew's Hospital, Barts NHS Trust, London, UK; ^2^Guy's and St Thomas' Hospital, London, UK

**Correspondence**: William Crawford

*Journal of Cardiothoracic Surgery 19(1):* A104

**Objectives:** Communication with General Practitioners and other teams is essential in post-operative Cardiac Surgery patients. GMC Good Medical Practice states that clinicians should share all relevant information with colleagues involved in the patients’ care. This complete audit cycle aims to assess whether surgical teams effectively communicate post-operative complications to General Practitioners and other specialties via clinic letters.

**Methods:** Consecutive patients undergoing cardiac surgery at a large tertiary unit were enrolled for two cycles: January and July 2022. First outpatient clinic letters at 6-week after surgery were reviewed against discharge summary. Operative urgency and postoperative complications were collected. An intervention was introduced which included education and poster advertisement for including this data in clinic letters. Significant complications identified included: dialysis, stroke, tamponade, resternotomy, tracheostomy, reintubation, new atrial fibrillation, pacemaker insertion and wound infection. Re-audit of patients undergoing surgery during July 2022 was performed to assess compliance to documentation guidance.

**Results:** Urgency of the operation was mentioned in 30.6% (11/36) in the first cycle and 26.9% (7/26) in the second. In the first audit cycle, 41.6% (15/36) commented on presence or absence of postoperative complications in clinic letters; compared to 38.5% (10/26) in the second cycle. Of those that had complications, 50% (6/12) were mentioned in the clinic letters at the first cycle but only 33.3% (4/12) in the second. The most common omitted complication was postoperative atrial fibrillation (50% (3/6) in first cycle and 62.5% (5/8) in the second).

**Conclusions:** Documentation of postoperative complications in clinic letters remained overall poor. Improving this practice proved challenging, potentially due to doctors changeover during this period. Nonetheless this is vital for continuity of care in postoperative patients.

## A105 Development and validation of multivariable logistic regression models for predicting return to theatre for bleeding following cardiac surgery

### Alexander Smith^1^; Paulo De Sousa^1^; Aw Tuan Chen^1^; Petrou Mario^1^; Tim Collier^2^; Stuart Pocock^2^; Eric Lim^1^

#### ^1^Royal Brompton and Harefield NHS Foundation Trust, London, UK; ^2^London School of Hygiene and Tropical Medicine, London, UK

**Correspondence**: Alexander Smith

*Journal of Cardiothoracic Surgery 19(1):* A105

**Objectives:** Post-operative bleeding requiring reoperation is a common complication following cardiac surgery. In this paper we construct predictive models for reoperation for bleeding at three-time points; pre-operatively, immediately post-operatively, and at the 3-h post-operative time point.

**Methods:** Data were obtained from a prospectively maintained single institution database. All patients over 16 years old undergoing bypass, valve or aortic surgery were included. Variables associated with reoperation were identified through univariate analysis. Predictors were combined into multivariable logistic regression models. Patients were stratified into risk groups and predicted and observed proportions of reoperation compared. Models were validated using an external dataset.

**Results:** A total of 11,846 patients met the eligibility criteria and were included in the study. 2944 (24.9%) were female. 327 (2.75%) patients required re-operation for bleeding.

The pre-operative model included gender, procedure, pre-operative anaemia, operative urgency, poor ejection fraction, use of dual antiplatelets and low body mass index. The only post-operative factor which added significance to the baseline model were intra-operative units of packed red cells. The post-operative model included 3-h peak lactate, cumulative chest drain output, and pre-operative anaemia. The pre-operative, operative and post-operative models performed with areas under the curve of 0.666, 0.701 and 0.898 respectively. External validation was carried out successfully; all three multivariable models performed well compared to the observed reoperation proportion.

**Conclusions:** The models showed good predictive capabilities for reoperation. The post-operative model has particularly good performance and could be used as a clinical tool to aid identification of patients requiring reoperation on the intensive care unit three hours post-operatively.

## A106 5 year service evaluation: the RECOVER project: restoration of sinus rhythm following elective cardiac surgery using VERnakalant

### Monica Mittal; Srinivasan Perumal; Sadeer Fhadil; Ian Rowlands; Sibtain Anwar; Martina Buerge

#### St. Bartholomew's Hospital, London, UK

**Correspondence**: Monica Mittal

*Journal of Cardiothoracic Surgery 19(1):* A106

**Objectives:** To assess the effectiveness and to evaluate the framework required to implement the use of Vernakalant for patients that developed postoperative atrial fibrillation (POAF).

**Methods:** We have included all patients post cardiac surgery that developed new AF ≤ 3 days duration based on inclusion and exclusion criteria.

**Table:** 

Inclusion criteria	Exclusion criteria
Not on multiple inotropes/vasopressors	Hypotension (systolic BP < 100 mmHg)
No other clear triggers for AF	Bradycardia/sinus node dysfunction/second- or third-degree heart block in the absence of pacemaker
Corrected aortic stenosis	Decompensated heart failure
	ACS in last 30 days
	Uncorrected severe aortic stenosis
	On class I and III antiarrhythmics 4 h prior and after Vernakalant administration
	Preoperative paroxysmal or permanent atrial fibrillation

From December 2017 to March 2022 a prospective cohort of 50 patients were enrolled. These patients received one or two doses of Vernakalant based on an approved internal protocol. If cardioversion was not achieved, Amiodarone or electric cardioversion was introduced. The data was collected from the electronic health records.

**Results**: Of the 50 patients identified, 20% were females and 80% were males. 34 (68.8%) patients required second dose of Vernakalant. The time to revert to SR after second dose was 97.5 min. The rate of conversion to SR following Vernakalant alone at discharge was 40%, the remaining patients required either Amiodarone, DCCV or a combination of both and anticoagulation. In our group, 10 (20%) patients were in persistent or paroxysmal atrial fibrillation at discharge irrespective of the cardioversion modality used and were anti coagulated. There was no need for further central line access insertion. The length of stay in acute critical was three days. there were four mortalities that were not related to Vernakalant or atrial fibrillation. Our main hurdles during this service evaluation were COVID pandemic that slowed the enrolment process, anecdotal evidence that contributed to barriers in changing organisational practices, difficulties in information-sharing as well as collecting the data.

**Conclusions:** Although there was an important, but not significant, rate of cardioversion to sinus rhythm with this agent, there is a need for further research in larger samples to define stricter inclusion criteria and to identify how Vernakalant can be best used for the benefits of the patients that are affected by POAF.

## A107 Remote monitoring of over 1000 patients awaiting cardiac surgery across London: the initial experience

### Martin Yates^1^; Sunil Bhudia^2^; Vasilios Avlonitis^3^; Vias Markides^2^; Cathy Walters^2^; Hillary Schrauwers^2^; Justine States^2^; Debashish Das^1^; Stephen Edmondson^1^

#### ^1^Barts Heart Centre / St Bartholomew's Hospital, London, UK; ^2^Guys and St Thomas’ NHS Trust; ^3^St Thomas Hospital, London, UK

**Correspondence**: Martin Yates

*Journal of Cardiothoracic Surgery 19(1):* A107

**Objectives:** Undetected deterioration at home whilst awaiting cardiac surgery can result in unplanned admission or death. The Covid 19 pandemic and subsequent backlog has elevated the frequency and risk of this. Remote monitoring is one solution to expedite treatment in those patients with worsening symptoms at home. The aim of this project is to describe the initial experience and outcomes of elective patients recruited onto virtual ward monitoring whilst awaiting cardiac surgery.

**Methods:** Initial recruitment occurred at five out of eight tertiary centres across London with the other centres in a second phase. All patients awaiting cardiac surgery on 19th September 2022, were invited onto Virtual Ward monitoring using Ortus iHealth platform. Patients completed weekly structured questionnaires assessing symptoms. Those with significant or deteriorating symptoms, highlighted through the software algorithm, were reviewed and treatment expedited.

**Results:** In the first 6 weeks, n = 1134 patients were invited of which 873 (77%) participated. Mean age was 66 years (Range 22–86) and 328 (29%) were Female. 56% of activated patients completed a weekly questionnaire, of which 57% flagged as red, 10% amber and 33% grey. Red & Amber alerts were reviewed through a combination of asynchrounous messaging through the platform or Tele-health consultation. There were no unplanned admissions and no deaths.

**Conclusions:** Remote monitoring of elective cardiac surgery patients has been successfully implemented across London with high patient participation and surgery expedited in a small but significant number of patients within the first month.

## A108 Is it time to use objective assessment methods to evaluate technical skills in cardiothoracic surgery?

### Nabil Hussein; Mahmoud Loubani

#### Castle Hill Hospital, Cottingham, UK

**Correspondence**: Nabil Hussein

*Journal of Cardiothoracic Surgery 19(1):* A108

**Objectives:** As modern cardiothoracic surgery (CTS) training moves towards a competency-based approach there is a need to develop and integrate objective assessment methods to measure technical skill progression and competence. This systematic review explored available objective assessment tools in CTS and whether they have been incorporated into training programmes.

**Methods:** Databases were searched for relevant articles that used objective assessment methods in the evaluation of technical skills in CTS. Data extracted included the task and assessment performed, the level of assessor(s), study outcome and incorporation into training. The Medical Education Research Study Quality Instrument (MERSQI) was used to score the studies’ methodological rigour.

**Results:** Fifty-four studies were included. Sixty-one percent of studies used a Likert method of assessment with the majority (85%) performed in a simulation setting. Cardiac surgery utilised objective assessments most with coronary anastomosis being the commonly assessed task. Evaluations were mainly performed by expert surgeons (78%) with 46% adopting blinding. Fifty-six percent (30) of studies showed changes in technical performance following repetition with 97% demonstrating improved performance. The remaining studies used assessments for validation purposes. The mean MERSQI score for the studies was 13.5 ± 1.5 demonstrating high validity. Thirty-nine percent of studies had incorporated these assessment tools into training programmes.

**Conclusions:** Objective assessments are available in CTS and have shown to assist technical skill development. With a growing emphasis on competency-based training there is an emerging need to incorporate such methods into modern training.

## A109 Pre-operative diabetic control in adult cardiac surgery as evidenced by glycosylated haemoglobin levels

### Bertie Harrington; Gemma Sowky; Ishtiaq Ahmed; Johnathon Hyde; Amit Modi; Uday Trivedi; Michael Lewis

#### Royal Sussex County Hospital, Brighton, UK

**Correspondence**: Bertie Harrington

*Journal of Cardiothoracic Surgery 19(1):* A109

Intraoperative control of glucose has long been recognised as important in achieving good patient outcomes, following cardiac surgery. More recently, the OCTOPUS trial is focusing on the impact of long-term diabetic control on these same outcomes.

The aim of this study was to highlight how well diabetic patients are being monitored and optimised prior to elective cardiac surgery. The current consensus in cardiac surgery is for a target of diabetic control giving an HbA1c of < 69 mmol/l quoted by several advisory bodies including NICE, CPOC and EACTS.

The study includes all elective cardiac surgery patients for two years between October 2020 and October 2022 at the Royal Sussex County Hospital looking at patients with known and newly diagnosed diabetes. Patient demographics, type and timing of surgery, initial clinic or referring HbA1c and admission HbA1c, diabetic management and post-operative outcomes such as wound infections and arrhythmias were included in retrospective data collection and analysis. Statistics were calculated using IBM SPSS Statistics v29.

There were 303 elective patients who underwent cardiac surgery, 122 had diabetes. 13 were insulin controlled, 55 medication and 33 diet. The mean listing HbA1c was 55.88 with 12.4% > 69 mmol/l. On average there was an improvement of 2.58% (p = 0.17, 95%CI-0.6,3.50) in HbA1c between clinic and admission, 92.6% had an HbA1c measured at both of these timepoints. 11.40% diabetics were operated on with an HbA1c > 69. 20.66% of these patients went on to have recorded arrhythmias and 4.13% wound infections.

In conclusions, our current therapeutic efforts make only small impacts on the degree of diabetic control for these patients. The outcomes of the OCTOPUS trial are eagerly awaited – do small changes in HbA1C make any difference to patient outcomes? Is 69 mmol/l an appropriate target for therapy? Are there ways to improve blood glucose control further than we are currently achieving, in the time frame available?

## A110 Factors associated with adverse outcomes after pericardiectomy

### David Varghese; George Gradinariu; Stewart Craig; Kasra Shaikhrezai

#### Golden Jubilee National Hospital, Glasgow, UK

**Correspondence**: George Gradinariu

*Journal of Cardiothoracic Surgery 19(1):* A110

**Objectives:** Pericardiectomy is associated with high morbidity and mortality. Surgery can be challenging, but highly effective. The aim of this study was to identify preoperative risk factors associated with adverse outcomes after surgery.

**Methods:** We conducted a retrospective analysis of all patients that underwent isolated pericardiectomy between 2012 and 2021 in our unit. We collected patient demographics, laboratory and imaging results, operative details and post-operative outcomes. The primary outcome was in-hospital mortality. Data was analysed using SPSS and Unistat statistical software. Binary logistic regression was used to identify factors associated with the primary outcome.

**Results:** 43 consecutive patients who underwent isolated pericardiectomy were included. The median age was 56 years, median logistic EuroSCORE was 2.3% and median BMI 27. 38 patients (88.4%) were male. 28 patients (65.1%) were in NYHA 3 or 4, 31 (72.1%) had good left ventricular function and in 39 cases (90.7%) cardiopulmonary bypass was not used. In-hospital mortality was 4.7% (2/43). At a median follow-up of 44 months, survival was 72%. Univariate logistic regression identified albumin (OR 0.832 [0.737–0.939], p = 0.001) as significantly associated with in-hospital mortality after surgery. The modified MELD score was associated with increased length of stay post-operatively (p = 0.03).

**Conclusions:** This study suggests that low preoperative albumin level is a predictor of mortality after isolated pericardiectomy. Also, a high MELD score, suggestive of liver congestion or dysfunction was found to be associated with postoperative morbidity. These findings underline the importance of preoperative optimization of the nutritional status as well as maximising the heart failure treatment regimen before embarking for surgery. This often requires a multidisciplinary approach and involvement from all members of the heart team.

## A111 MINI BYPASS: utility in modern cardiac surgical practice

### Brianda Ripoll; Mahmoud Loubani; Martin Jarvis; Alexander Cale; Dumbor Ngaage; Andrew Wallhead; Robert Bennet; Mubarak Chaudhry

#### Castle Hill Hospital, Hull, UK

**Correspondence**: Brianda Ripoll

*Journal of Cardiothoracic Surgery 19(1):* A111

**Objectives:** Mini-Extracorporeal technology (MIECT) was shown to have short-term advantages over Conventional Bypass (CB) and Prime Displacement (PD) in coronary and aortic valve surgery. This observational study reports the long-term results of 15-year experience of a single centre of our cohort of 2952 patients that underwent Cardiac Surgery with MIECT.

**Methods:** A retrospective analysis of our cardiac surgical database was performed for patients operated on in the last 15 years. The description of the variables were analysed with SPSS 28.0.1.

**Results:** A total of 2952 patients underwent Cardiac Surgery with MIECT. This represents 23% of the total patients over the same period. The mean age of our cohort was 68 years old ± 18) with a mean BSA of 1.96 ± 0.20. 2309 were male (78.2%) and 643 females (21.8%). 1096 operations were urgent (37.1%), 1740 elective (59%) and 102 emergencies (3.48%).

Intraoperatively, 2284 required subsequent prime (77.4%) and 665 did not (22.5%). Most of them 2119 (71.9%) were single cardiac procedures, 540 (18.3%) were double (CABG + 1 valve) and 257 (8.7%) were triple (CABG + 2 valves). 814 (27.6%) were single valve replacements. 25 (8%) were major aortic cases. The mean cumulative cross clamp time in minutes was 58.7 ± 24. Only 67 (2.3%) required to go back onto bypass with PD and 105 (3.6%) required re-opening for bleeding.

Post operatively, 1528 (51.8%) presented postoperative arrhythmias while 55 of them (1.9%) developed a new post-operative stroke. Overall, 2878 (97.5%) patients are alive with a mortality of 64 patients (2.2%).

**Conclusions:** MIECT has short-term and long-term advantages in a larger cardiac community and is a safe regular practice in our unit with excellent survival results when performed by an experienced team of Perfusionists and Cardiac Surgeons.

## A112 Infective endocarditis profile of an 8 year experience

### Abdul Badran; Sunil Ohri; Henry Rowe; Cynthia Nwakou

#### University Hospital Southampton NHS Foundation Trust, Southampton, UK

**Correspondence**: Abdul Badran

*Journal of Cardiothoracic Surgery 19(1):* A112

**Objectives:** Endocarditis remains a disease associated with significant morbidity and mortality. Surgical management is reserved in failed medical therapy and from serious complications related to valve destruction or embolic phenomena. We reviewed the assessment and management of patients with endocarditis in our busy cardiothoracic surgical centre.

**Method:** We retrospectively reviewed patient data from 2012 to 2020 including demographics, clinical and follow-up details from patient notes in our tertiary services teaching hospital. 574 were included over the 8-year period.

**Results:** Of these 379 (66%) underwent an operation (Vs n = 195). 303 (80%) were males, 76 were females (20%) and mean age was 57 (18–95). Of the patients that underwent surgery, 300 (79%) were alive at latest follow up (Vs n = 67 (34%). Of those that had operations compared to those that did not on micro culture 32% (n = 120) had variants of Staph. bacteria (Vs 17%, n = 33, 0 were MRSA); 22% (n = 84) had variants of Strep. (Vs n = 25); 8% (n = 30) had enterococcus (Vs 7% (n = 14); and 7% (n = 25) had other organisms (vs. 7% (n = 14). Patient that had IE related to a pacing device or wires made up 4% (n = 23 patients) of the cohort. Of those undergoing surgery 9% (n = 34) had concomitant CABG with their infective endocarditis surgery. In the 22% (n = 129) of patients that died on last follow up 31% (n = 40) had SA, n = 17 had strep and, 15 had enterococcus, 3 had candida albicans. For those that were operated mean day to discharge was 33 Vs 35 in those non operated.

**Conclusions:** In our unit we operate on the majority of patients with endocarditis. Of the patients that were operated they had higher survival than those unoperated, although overall time to discharge did not seem to differ. This may be due to referral practices in our unit but does bear favourably for surgery in endocarditis.

**Adult Cardiac Mitral Valve**

## A113 The effectiveness of cadaveric simulation for training in minimal-access cardiac surgery

### Ali Mohammadi^1^; Joseph Zacharias^2^; Toufan Bahrami^3^; Hunaid Vohra^4^; Antonios Pitsis^5^; Dincer Aktuerk^6^; Ranjit Deshpande^7^; Ishtiaq Ahmed^8^

#### ^1^Brighton & Sussex Medical School, Brighton, UK; ^2^Lancashire Cardiac Centre, Blackpool, UK; ^3^Royal Brompton & Harefield, London, UK; ^4^University Hospitals Bristol, Bristol, UK; ^5^Eurobalkan Hospital, Athens, Greece; ^6^Barts Heart Centre, London, UK; ^7^King's College Hospital, London, UK; ^8^University Hospitals Sussex, Brighton, UK

**Correspondence**: Ali Mohammadi

*Journal of Cardiothoracic Surgery 19(1):* A113

**Objectives:** Multifactorial issues, such as increased concern for patient safety, reduced available didactic time, high workload, duty-hour restrictions, and the complexity of procedures have affected training in Cardiac Surgery. Cadaveric-simulation training has emerged as a useful adjunct to facilitate specialist operative skills. The goal of this study is to assess the effectiveness of using cadaveric labs in specialist training.

**Methods:** 13 candidates including consultants and senior trainees from two Minimal-Access Cadaveric workshops completed a detailed questionnaire based on their level of knowledge before and after the courses. The questionnaire was based on a 5-point rating scale in respect of Pre-Operative Assessment of patient, Patient Set up and Cannulation, and Surgical Procedures. This initiative was primarily funded by a charitable educational grant from Heart Research UK. In addition, one of the courses was supported by live streaming.

**Results:** Performance self-assessment demonstrated an improvement in each of the assessed components after the human cadaveric simulation. In the Pre-operative Assessment of patient, the level of the trainee’s understanding enhanced from a mean score of 11 to 17.3. Improvement in Patient Set up and Cannulation was achieved with pre- and post-feedback scores from 13.6 to 18.7 respectively. The degree of improvement appeared to be greatest in the Surgical Procedures from 33.8 to 48.9.

**Conclusions:** This study suggests that introducing cadaveric simulation exercises in technically challenging Minimal-Access cardiac surgery provides an effective means of enhancing trainees’ skills. Therefore, they can be an excellent means of transferring techniques to prepare the next generation of cardiac surgeons with minimal access ability, with the final goal of yielding optimised surgical results for patients’ well-being.

## A114 Sex-based differences in early outcomes following mitral valve surgery for degenerative disease

### Fadi Al-Zubaidi; Maria Pufulete; Hunaid Vohra

#### Bristol Heart Institute, Bristol, UK

**Correspondence**: Fadi Al-Zubaidi

*Journal of Cardiothoracic Surgery 19(1):* A114

**Objectives:** To determine whether sex-based differences exist following surgery for degenerative mitral valve disease.

**Methods:** Using a large national database we analysed data on mitral valve surgery for primary degenerative disease (n = 22,658) between January 2000 and March 2019 in the UK. We split the cohort by sex into males (n = 14,681) and females (n = 7977) and compared background characteristics, intraoperative variables and short-term postoperative outcomes. Our primary outcome was in-hospital mortality; secondary outcomes included takeback-to-theatre, prolonged admission (> 10 days) and mitral replacement. We planned binary logistic regression models for all outcomes. We used multiplicative interaction terms to determine the nature of any sex-based differences.

**Results:** Females presented older with more advanced disease and worse symptom profiles. They had lower repair rates (66% vs 76%, p < 0.001), higher mortality (3% vs 2%, p < 0.001) and more prolonged admissions (48% vs 40%, p < 0.001). Binary logistic regression modelling found female sex to be an independent predictor of mortality (OR:1.52, 95% CI:1.21–1.90, p < 0.001). The variables age and CCS score demonstrated significant interaction terms with sex. When stratifying by age group, female sex was only found to be an independent predictor of mortality in patients aged between 64 and 74 (OR:2.34; 95% CI:1.57–3.50). When stratifying by CCS score, female sex was a stronger predictor of mortality in patients with CCS scores III–IV (OR:2.49; 95% CI:1.20–5.20) compared to CCS I–II (OR:1.73; 95% CI:1.12–2.67) or CCS 0 (OR:1.30; 95% CI:0.97–1.73). Female sex was an independent predictor of prolonged admission and mitral replacement; it protected against take-back-to-theatre (Fig. 1).

**Fig. 1:**
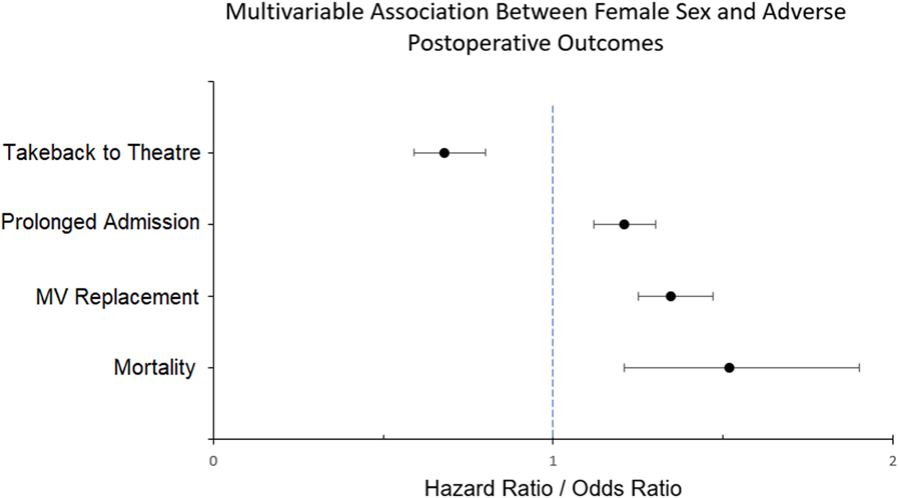
Forest plot displaying multivariable association between sex and outcomes

**Conclusions:** Female sex is an independent predictor of mortality, prolonged hospital admission and mitral replacement. The relationship between female sex and mortality is exacerbated by worsening CCS scores. Females have significantly lower repair rates.

## A115 Mitral repair versus replacement: twenty-year outcome trends in the UK (2000–2019)

### Fadi Al-Zubaidi; Maria Pufulete; Shubhra Sinha; Hunaid Vohra

#### Bristol Heart Institute, Bristol, UK

**Correspondence**: Fadi Al-Zubaidi

*Journal of Cardiothoracic Surgery 19(1):* A115

**Objectives:** Using a large national database, we sought to describe outcome-trends in mitral valve surgery (MVS) between 2000 and 2019.

**Methods:** The study cohort (n = 63,000) was split into MV repair (MVr) (n = 31,644) and MV replacement (MVR) (n = 31,356). Patients were grouped by four-year admission period into groups (A to E). Cochran-Mantel-Haenzsel test was used to investigate categorical variable trends; for continuous variables, simple linear regression was used. A multivariable binary logistic regression model for mortality was planned to assess the independent relationship between mortality and time. Cohorts were further stratified by sex and aetiology; regression analyses for mortality were performed inthese sub-groups. Secondary outcomes included return to theatre, post-operative stroke and admission length.

**Results:** Significant demographic shifts were observed. Aetiology has shifted towards degenerative disease; endocarditis rates in MVr dropped initially but are now rising period A = 6%, period C = 4%, period E = 6%; p < 0.001). The burden of comorbidities has increased over time. Ablation rates remain low in both MVr (13%) and MVR (6%) groups. In the latest time period, females have lower repair rates (49% vs 67%, p < 0.001) and higher mortality rates when undergoing repair (3% vs 2% < p = 0.001) than males. Unadjusted post-operative mortality dropped in MVr (5% vs 2%, p < 0.001) and MVR (9% vs 7%, p = 0.015). Secondary outcomes have improved. Time period was an independent predictor for reduced mortality in both MVr (OR: 0.41, 95% CI: 0.28–0.61, p < 0.001) and MVR (OR: 0.50, 95% CI: 0.41–0.61, p < 0.001) (Fig. 1).

**Fig. 1:**
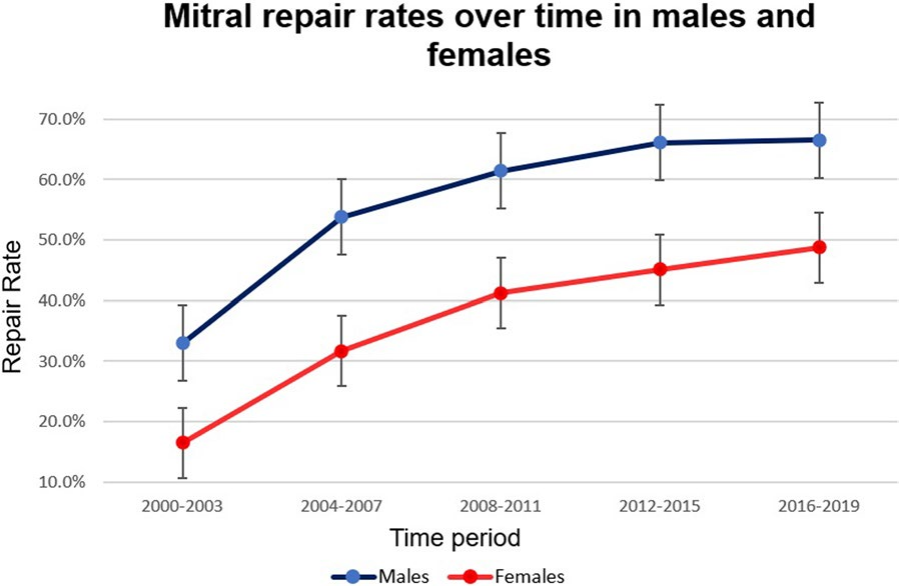
Sex-based differences in mitral valve repair rate trends (2000–2019)

**Conclusions:** In-hospital mortality has dropped significantly over time for mitral valve surgery in the UK. MVr has surpassed replacement to become the more common procedure. Sex-based discrepancies in repair rates and mortality require further investigation. Rates of concomitant AF ablation are low when compared to rates of preoperative AF.

## A116 Starting minimal invasive mitral valve surgery program, milestones and challenges

### Ahmed Shazly; Alessia Rossi; Hasnat Khan; Arvind Singh; Sudhir Bhusari; Alberto Albanese; Mohamed Osman; Ayman Asfour

#### Essex Cardiothoracic Centre, Basildon, Basildon University Hospital, UK

**Correspondence**: Ahmed Shazly

*Journal of Cardiothoracic Surgery 19(1):* A116

**Objectives:** Starting a minimal invasive program in cardiac centre is a challenge. We are presenting our experience in re-starting a minimal invasive mitral program. The program re-started in October 2021 by building a team of minimally invasive mitral valve including a dedicated heart team. The team had to go through the challenge of sharing different experiences and prospective among the different disciplines and with proctorship for the first four cases.

Like any program starting, the operation time was longer which reflected on the theatre efficiency. In addition, a protocol for emergencies during ITU course had to be generated and agreed among surgeons and ITU consultants.

**Methods:** Twenty-five patients underwent minimal invasive mitral valve surgery (MIMVS) between October 2021 and October 2022.

Short term (30 days) mortality was 0%, rate of conversion was 8% (2 cases) and most patients went home within one week.

The cases were preoperatively carefully assessed to ensure suitability for minimal invasive procedure. All patients had pre-operative CT scan, echo, PFT, bloods in addition to pre-operative anaesthetic review. Two consultants are allocated per each case complying with NICE guidelines to start a new clinical program in a Trust.

We are working on increasing the number of patients by more than double by next year, starting concomitant tricuspid valve surgery and AF ablation.

MIMVS provides an excellent outcome in case of carefully selected. The patients had a smooth post-operative course with early discharge and better recovery. A coordinated team-based structure was achieved during this year with more progress in learning curve and better results.

**Conclusions:** Our program started and run safely with no in-hospital mortality. Training curve is improving with every case, surgical and anaesthetic team are getting more familiar with the approach. It was a good exercise for the team to share opinions and contribute to safe conduction of the procedure.

## A117 Left ventricular dimensions as a predictor for mortality after combined CABG and MV surgery

### Walid Hammad^1^; Ashraf Zahra^2^

#### ^1^Al-Azhar Faculty of Medicine, Cairo, Egypt; ^2^Shebin El kom Teaching Hospital, Shebin El Kom, Egypt

**Correspondence**: Walid Hammad

*Journal of Cardiothoracic Surgery 19(1):* A117

**Introduction:** Left ventricular size is a factor independently associated with increased mortality in mitral surgery, particularly, in the setting of IMR. LV dimensions are an important predictor of reverse re-modelling. In some patients, reverse re-modelling does not occur, with a high risk of residual or recurrent MR and therefore associated with worse outcome (Table 1).

**Table 1:** Pre and postoperative LV dimensions in survival and mortality groups. In both genders, a statistically significant persistent elevated LV ESD dimensions postoperatively was associated with higher mortality rate. While a significant elevated LV EDD was only noted in female patient

Mortality group 26 (9.05%)		preop	postop	P value	
Male 15 (5.22%) ? EDD ? ESD	EDD	58.53	54.50	> 0.05	
	ESD	47.46	46.78	< 0.001	
Female 11 (3.83%)	EDD	50.18	52.00	< 0.001	
	ESD	38.90	40.91	< 0.001	
Survival group 261 (90.94%)					
Male 182 (63.41%)	EDD	59.39	55.98	> 0.05	
	ESD	46.76	44.34	> 0.05	
Female 79 (27.52%)	EDD	55.49	52.02	> 0.05	
	ESD	40.13	39.24	> 0.05	

**Objectives:** The aim of this study to corelate a relationship between preoperative and early postop LV dimensions to mortality rates after combined CABG and MV surgery.

**Methods:** A retrospective observational study was conducted on patients had combined CABG and MV surgery for moderately severe IMR between 2009 and 2022. Exclusion criteria was redo surgery, nonischemic mitral regurgitation, + 2 MR or less, and association of any other valve lesion. The normal reference value for the LV EDD and ESD dimensions were set at 59 and 42 mm for males while a 53 and 39 for females were considered.

**Results:** 287 patients had combined CABG and MV surgery. The mean age was 62.36 years. 68.6% males and 31.35% female were included. 65.5% has evidence of previous MI, with ECG confirmed of MI in 16.02%, 10.10%, 2.09% at the inferior, anterior, and lateral walls respectively. Symptoms of CHF with dyspnea on exertion dominated the presentation with 63.7% had NYHA III and 18.5% class IV. MVR was done in 37.63%, while mitral repair was possible in 62.37%. The average preoperative and early postoperative LVEF were 33.84% and 34.44 respectively. In both genders, a statistically significant persistent elevated LV ESD dimensions postoperatively was associated with higher mortality rate. While a significant elevated LV EDD was only noted in female patient.

**Conclusions:** Failure of early ventricular remodeling with persistent elevated ESD is a risk factor for postoperative death in both genders. Females gender will have an additional higher risk if the EDD remains elevated.

## A118 Simultaneous Cox-Maze in patients with AF undergoing mitral valve surgery is associated with superior 1-year freedom from AF

### Davorin Sef^1^; Vladimir Trkulja^2^; Joanne Hooper^3^; Shahzad Raja^4^; Marko Turina^5^

#### ^1^University of Southampton NHS Foundation Trust, Southampton, UK; ^2^Medical School, University of Zagreb, Croatia, EU, Zagreb, Croatia; ^3^University of Bristol, Bristol, UK; ^4^Royal Brompton and Harefield Hospitals, Harefield, UK; ^5^University of Zurich, Zurich, Switzerland

**Correspondence**: Davorin Sef

*Journal of Cardiothoracic Surgery 19(1):* A118

**Objectives:** While up to 50% of patients undergoing mitral valve (MV) surgery have preoperative atrial fibrillation (AF), data regarding the comparative effectiveness of simultaneous Cox-Maze and pulmonary vein isolation (PVI) procedures are still limited. We conducted a systematic review of randomized controlled trials (RCTs) and non-randomized studies (NRSIs) directly comparing the two procedures in this setting with additional meta-analysis of RCTs.

**Methods:** A systematic literature search was performed from 1987 to 2022 for studies comparing simultaneous Cox-Maze and PVI during MV surgery. A meta-analysis of RCTs was performed to compare the one-year clinical outcomes between these two surgical ablation techniques.

**Results:** Six studies with a total of 790 patients met the inclusion criteria. Pooled analysis found comparable odds of AF recurrence during one1-year follow-up (OR = 1.10, 95% CI [0.26, 4.62]), although the largest RCT (CTSN trial) clearly demonstrated higher risk of AF recurrence after the simultaneous PVI procedure during MV surgery. In two out of three NRSIs, one-year AF recurrence was higher in PVI when compared to the Cox-Maze group (estimated adjusted probabilities 35% vs. 17% and 11% vs. 8%, respectively). Adding a Cox-Maze procedure did not seem to affect the operative mortality of MV procedures.

**Conclusions:** Simultaneous Cox-Maze in AF patients undergoing MV surgery is associated with better one-year freedom from AF. Observational studies suggest that there is a mid-term survival benefit for patients who undergo simultaneous Cox-Maze. Further large RCTs with quality-of-life analysis are still required (Fig. 1).

**Fig. 1:**
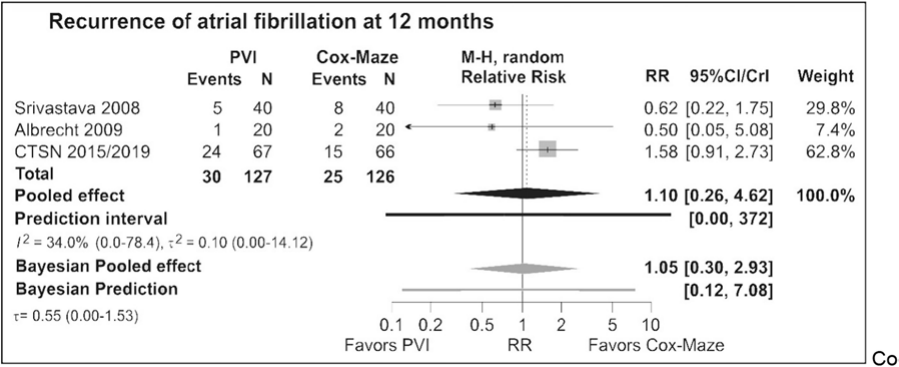
Forest plot showing the effect of pulmonary vein isolation (PVI) and Cox-Maze procedure for AF during the mitral valve surgery on 12-month mortality and recurrence of AF in randomized controlled trials

## A119 Long-term outcomes after rheumatic mitral valve replacement at a large tertiary centre

### Vinci Naruka; Selina Tsai; David Bleetman; Amir Sheikh; Shyamsunder Kolvekar; Kulvinder Lall; Kit Wong; John Yap; Rakesh Uppal; Dincer Aktuerk

#### St. Bartholomew Hospital, London, UK

**Correspondence**: Vinci Naruka

*Journal of Cardiothoracic Surgery 19(1):* A118

**Objectives:** Rheumatic mitral valve disease has traditionally been treated with replacement (MVR). Recently, single centre studies have advocated for repair in "selected" patients. We report MVR patient outcomes for rheumatic heart disease (RHD) at a large tertiary centre.

**Methods:** A prospective database was retrospectively analysed in 179 consecutive RHD patients undergoing MVR from January 2015 to March 2022. Redo sternotomy or concomitant coronary artery bypass were excluded. We report outcomes including mortality and postoperative adverse event rates.

**Results:** Average age was 57 ± 13 years. 80.4% (n = 144) were female. Median EuroSCORE II was 1.8% (IQR 1.13–2.86) and 15.1% (n = 27) underwent non-elective MVR. Valve-type was mainly mechanical (75.7%, n = 131). Concomitant tricuspid valve repair (TVr) was performed in 20.7% (n = 37) and 5% (n = 9) underwent concomitant aortic valve replacement with or without TVr. Mean cardiopulmonary bypass time was 117 ± 66 min with cross-clamp of 89 ± 44 min. Postoperative complications were: resternotomy for bleeding (3.4%, n = 6), cerebrovascular accident (1.1%, n = 2), dialysis (2.8%, n = 5), atrial fibrillation (20.1%, n = 36), pacemaker (8.4%, n = 15). Mean postoperative stay was 11.8 ± 8.6 days. Long term valuation of left ventricular (LV) function showed no significant postoperative deterioration. Freedom from prosthesis related complications was 97.7% (85/87) with 633 days mean follow up. Freedom from reintervention was 96.6% (173/179) with 50% (3/6) for endocarditis. 30-day mortality was as follows: isolated MVR (elective (0.9%, 1/107), urgent (0%, 0/20)) and MVR with concomitant procedures (elective (11.1%, 5/45), urgent (14.3%, 1/7)). Long-term survival was evaluated using Kaplan–Meier analysis (Fig. 1).

**Fig. 1:**
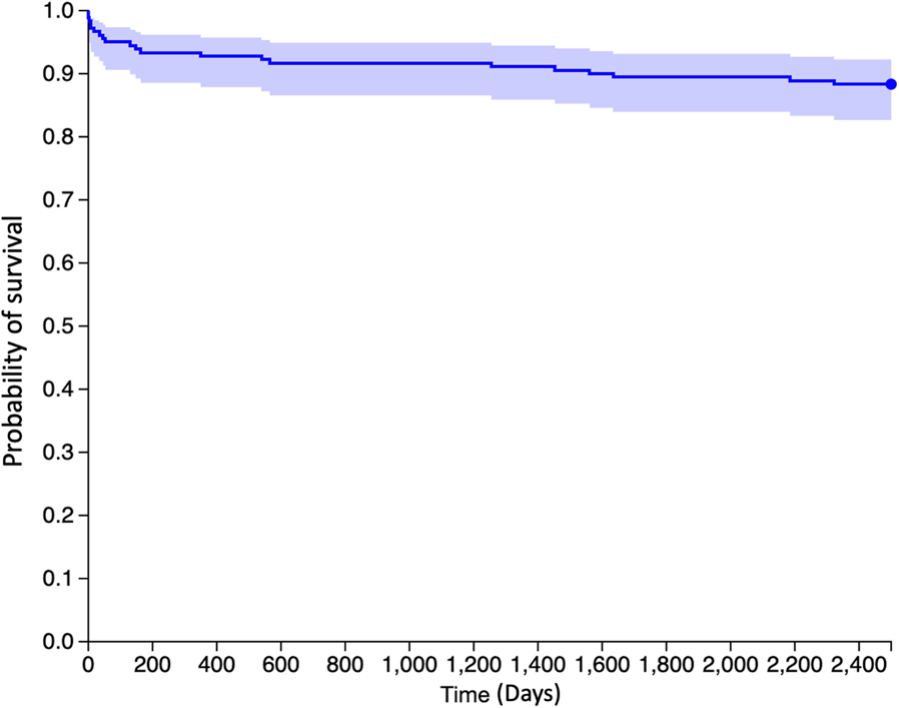
Long-term survival for patients with rheumatic mitral valve disease undergoing mitral valve replacement, evaluated using Kaplan–Meier analysis

**Conclusions:** MVR remains the gold-standard treatment for rheumatic mitral valve disease. 30-day and long-term mortality is excellent, valve related complications are minimal and with careful operative approach, LV function is preserved.

## A120 Did the Covid pandemic impact minimally invasive heart valve surgery outcomes?

### Ravi Ghatanatti; Palanikumar Saravanan; Andrew Knowles; William Simpson; Grzegorz Laskawski; Carmelo Raimondo; Joseph Zacharias

#### Blackpool Teaching Hospitals NHS Foundation Trust, Blackpool, UK

**Correspondence**: Ravi Ghatanatti

*Journal of Cardiothoracic Surgery 19(1):* A120

**Objective:** Globally, COVID-19 has had a considerable impact on the delivery of cardiac surgery. The study aims to compare impact of COVID-19 pandemic on isolated adult heart valve procedures to look for any impact on outcomes in minimal access valvular procedures at single cardiac surgery centre in the United Kingdom.

**Methods:** A retrospective cohort study was undertaken utilizing prospectively collected data of isolated heart valve procedures. Surgical approaches used during this period, were conventional sternotomy, hemi-sternotomy, right anterior thoracotomy, and fully endoscopic. Pre-pandemic data (Period 1) of 2018–19 was compared with pandemic period data (Period 2) of 2020–21. All the patients presenting with isolated valvular diseases and redo valvular procedures were included, whereas; combined procedures were excluded from the study. All covid positive patients were treated for the recommended duration before undergoing a procedure.

**Results:** A total of 862 procedures were performed, of which 496 were performed in period 1 and 366 in period 2, a decrease by 26.20% (Table 1) was noted. In period 1, minimal access valvular procedures accounted for 37.41% (group 1) and in period 2, it was 33.60% (group 2). The incidence of post operative stroke was 2.73% and 1.20% and pneumonia was reported at 13.70% and 16.66% in group 1 and 2 respectively. The median hospital length of stay for both groups was 7 days. Mortality rate was 2.21% for group 1 and 1.09% for group 2.

**Table 1:** Case volumes for periods 1 & 2 displayed for each surgery as total/MICS

Procedures	Period 1—Total 496, Sternotomy 311, MICS 185. Total/ MICS	Period 2—Total 366, Sternotomy 243, MICS 123. Total/MICS
Aortic valve replacement	297/ 96	199/66
Mitral valve replacement/repair	131/63	120/48
Double valve replacement	25/1	17/3
Mitral with tricuspid valve repair	30/17	22/8
Aortic with tricuspid valve	3/1	3/0
Tricuspid valve repair	7/7	2/0
Triple valve surgery	3/0	3/0

**Conclusions:** Overall, there was a reduction in the number of isolated valve cases operated on since the pandemic but the pandemic itself did not have any deleterious impact on outcomes in minimal access heart valve procedures in our institution.

## A121 Concomitant tricuspid valve repair for moderate tricuspid valve regurgitation in association with left-sided valve surgery: a meta-analysis

### Shubham Jain; Mohsin Uzzaman; Anupama Barua; Prakash Nanjaiah; Ravish Jeeji; Lognathen Balacumaraswami

#### Royal Stoke University Hospital / UHNM NHS Trust, Stoke-On-Trent, UK

**Correspondence**: Shubham Jain

*Journal of Cardiothoracic Surgery 19(1):* A121

**Objectives:** There is controversy over the optimal management of moderate tricuspid valve regurgitation during left-sided valve surgery due to the paucity of robust evidence for superior outcomes from concomitant tricuspid valve repair. Hence, we assessed the literature to compare the clinical outcomes in patients undergoing left-sided valve procedures with or without tricuspid valve repair surgery.

**Methods:** We performed a literature search using MEDLINE, EMBASE, Scopus and Web of Science. We identified articles to evaluate primary outcomes including overall mortality and cardiac mortality. Secondary outcomes were stroke, wound infection, reoperation, new-onset atrial fibrillation, renal failure, intensive care duration and permanent pacemaker. All analysis was done using the random effects model.

**Results:** There was no significant difference in overall mortality but very significant reduction in cardiac mortality (p < 0.0001) with concomitant tricuspid valve repair despite higher CPB (p < 0.00001) and X-Clamp times (p < 0.00001). There was lower postoperative tricuspid regurgitation (p < 0.00001) and reduced permanent pacemaker implantation rates (p < 0.05) with concomitant tricuspid repair. There was no difference in postoperative stroke, wound infection, atrial fibrillation, renal failure and intensive care duration of stay between the two groups.

**Conclusions:** Concomitant tricuspid valve surgery for moderate tricuspid regurgitation during left-sided valve surgery offers a definite survival benefit with a significant decrease in cardiac mortality. Additionally, this strategy results in significant decrease in both, permanent pacemaker implantation and late tricuspid regurgitation, with no increase in morbidity.

## A122 Safety, effectiveness, and long-term outcomes of mitral valve surgery performed via minimally invasive access: a 10-years, single-surgeon experience

### Riccardo Abbasciano; Mohd Shahbaaz Khan; Sanjay Chaubey; Antanas Macys; Peter McCartney; Farzana Aslam; Thanos Athanasiou; Roberto Casula

#### Imperial College Healthcare NHS Trust, London, UK

**Correspondence**: Riccardo Abbasciano

*Journal of Cardiothoracic Surgery 19(1):* A122

**Objective:** Minimally invasive approaches for mitral valve surgery have been developed for approximately 30 years, although their adoption is not systematic yet. Several contemporary studies show equal or improved outcomes when comparing minimally invasive surgery to the traditional approach via median sternotomy. Nonetheless, few series are exclusive to the activity of a single experienced minimally-invasive operator.

**Methods:** We reviewed retrospectively the cases, indications, and outcomes (both in-hospital and at follow-up) of the mitral valve surgeries performed through a minimally invasive approach in the last 10 years of activity of a single surgeon at the Hammersmith Hospital. A propensity-score matched cohort was selected to compare groups with similar pre-operative characteristics.

**Results:** A total of 919 patients were included. The average follow-up was 1874 days. The two groups differed for age (63.3 ± 13.3 years for median sternotomy vs 60.4 ± 14.1 years for minimally invasive, p = 0.013), baseline creatinine (79, IQR 70–96, µmol/L vs 71, IQR 65–80, µmol/L respectively, p < 0.001), ejection fraction (54.2 ± 8.5% vs 56.4 ± 6.8%, p = 0.006) and EuroSCORE II (4.58 ± 7.6% vs 2.3 ± 4.1%, p = 0.005). Gender representation (43% vs 41% female, p = 0.717), baseline haemoglobin (12.6 ± 2.1 g/dl vs 13.4 ± 1.8 g/dL, p = 0.418) and rates of comorbidities were comparable. After propensity score matching, no differences were recorded for in-hospital mortality (0% in both groups, p = 1). Reduced use of blood products was appreciated in the minimally invasive group (p = 0.048). A Kaplan–Meier analysis did not show significant differences in long-term survival, in both the unmatched or matched cohorts (p = 0.810).

**Conclusions:** Minimally invasive mitral valve surgery is as safe and effective as conventional approaches when performed by an experienced operator. Future works will expand the analysis to capture rates of reoperations and preoperative markers of prognosis.

**Adult Cardiac Scientific and Experimental**

## A123 Cardiomyocyte-specific inhibition of P16ink4 improves recovery following myocardial infarction with reperfusion

### Omowumi Folaranmi^1^; Rachael Redgrave^1^; Emily Dookun^1^; Anna Walaszczyk^2^; Laura Booth^1^; Simon Tual-Chalot^2^; Andrew Owens^3^; Enoch Akowuah^3^; Ioakim Spyridopoulos^2^; Helen Arthur^2^; Joao F Passos^4^; Gavin Richardson^2^

#### ^1^Newcastle University, Newcastle upon Tyne, UK; ^2^Newcastle University, Newcastle upon Tyne, UK; ^3^James Cook University Hospital; Middlesbrough, UK; ^4^Mayo Clinic, Rochester

**Correspondence**: Omowumi Folaranmi

*Journal of Cardiothoracic Surgery 19(1):* A123

**Objectives:** Recently we demonstrated in mice models, that following a myocardial infarction (MI), cardiomyocytes (CMs) obtained a senescent phenotype. Treatment with the Bcl-2 inhibitor navitoclax, eliminated senescent cells and improved clinical outcomes. In this study we aimed to 1) identify that the observed improvement was due to the senolytic activity of navitoclax and not off-target effects and 2) identify if senescence contributes to remodelling clinically.

**Methods:** We established a transgenic model of cardiomyocyte specific inhibition of senescence, via knockout of p16Ink4a, a key regulator of senescence. We also quantified senescence in human myocardial tissue and correlated this to biomarkers of remodelling.

**Results:** Following MI, mice lacking CM p16Ink4a expression demonstrated reduced cardiomyocyte senescence, reduced scar size and improved cardiac function compared to controls. In humans, myocardial expression of senescence-associated genes correlated with indicators of remodelling including heart size and pro-BNP expression.

**Conclusions:** Our data suggest that, in mice, MI-induced CM senescence promotes scar formation, leading to cardiac dysfunction. Data from human samples further suggest that myocardial senescence is associated with remodelling clinically. Together, this suggests that targeting elimination of the senescent cardiomyocyte population is a potential therapeutic strategy to improve outcomes from MI.

## A124 Solutions for the preservation of vein graft endothelium: a systematic review and narrative synthesis

### Georgia Layton^1^; Shameem Ladak^1^; Liam McQueen^1^; Riccardo Abbasciano^2^; Mustafa Zakkar^1^

#### ^1^Department of Cardiovascular Sciences, University of Leicester, Leicester, UK; ^2^Department of Cardiothoracic Surgery, Imperial College Healthcare NHS Trust, Leicester, UK

**Correspondence**: Georgia Layton

*Journal of Cardiothoracic Surgery 19(1):* A124

**Objectives:** Use of saphenous vein conduits for CABG is complicated by an increased risk of occlusion due to the development ‘vein graft disease’ (VGD). Endothelial dysfunction is a key driver of VGD and whilst its causation is multi-factorial, emerging evidence identifies conduit harvest technique and preservation fluids as critical culprits in its onset and propagation. This review aims to assess currently published data on the relationship between preservation solutions and endothelial cell (EC) integrity and function in human LSVs.

**Methods:** The review was registered with PROSPERO (CRD42022358828). Electronic searches of Cochrane Central Register, MEDLINE, and EMBASE databases were undertaken from inception until August 2022. A narrative synthesis was performed for all included studies. Quality assessment was evaluated using the ROBINS-I tool. Given the anticipated diversity of outcome measures, limited scope for statistical analysis was expected and as such, meta-analysis was not undertaken.

**Results:** Eleven prospective controlled studies were included. All studies used normal saline as a control solution. Intervention solutions included heparinised whole blood and saline, DuraGraft, TiProtec, Euro-Collins, University of Wisconsin, Ringer’s lactate, HTK and Pyruvate solutions. Studies investigated numerous outcomes synonymous with EC structure (ranging from immunohistochemistry to identify endothelial-derived markers such as CD31 and CD34, direct visual assessment of EC morphology to oxidative stress) or EC dependent relaxatory function. Only one paper reported clinical patient outcomes.

**Conclusions:** There is gross heterogeneity in practice and reporting. Current data does not facilitate generation of robust data to support changes to clinical practice at present. There is a need for prospective interventional studies to address this common feature of practice to improve long-term patency of venous conduits.

## A125 Towards the prevention of vein graft disease: defining gender differences in the genetic make-up of saphenous veins

### Georgia Layton; Shameem Ladak; Liam McQueen; Mustafa Zakkar

#### Department of Cardiovascular Sciences, University of Leicester, Leicester, UK

**Correspondence**: Georgia Layton

*Journal of Cardiothoracic Surgery 19(1):* A125

**Objectives:** The long-term effectiveness of CABG is limited by vein graft failure. It’s been noted that women have a higher incidence of MI and repeat revascularisation after CABG, compared to men. The reasons for this are not known. Up to 25% of the endothelial cell transcriptome is sex-specific and many genes with a key role in the development of vein graft disease have been shown to be differentially expressed in female cells in-vitro, suggesting genetic variance may account for this disparity.

**Methods:** Surplus segments of veins obtained from people undergoing CABG were retrieved immediately after harvesting then fresh-frozen, fixed, and segmented. Spatial cell RNA sequencing was performed using the 10X Visium Spatial Gene expression kit and protocol. Findings were validated with quantitative PCR and immunostaining. Analysis and figures were performed in R and RStudio. Patient consent was obtained pre-operatively.

**Results:** Vein segments from three female and three male patients were studied. 4.2% (482) of genes were significantly different at baseline between males and females, including differences in genes regulating the inflammatory response (chemokines, cytokines, AP-1, and regulators of MAPK and NF-KB), heat shock proteins, genes with a key role in the remodelling of the extracellular matrix (ADAMTS), and those modulating both endothelial and smooth muscle cell function, proliferation and phenotype switching (TGF, VEGFA and PDGF). A select profile of genes was validated using reverse transcriptase quantitative PCR to confirm our findings.

**Conclusions:** We have demonstrated, for the first time in humans, that there are significant genetic differences at baseline in vein grafts harvested from men and women. It is highly likely that the development of vein graft disease after CABG reflects different processes between genders. These differences may require different therapeutic approaches tailored by sex.

## A126 Dexamethasone pre-treatment inhibits OPN-activation associated with vein graft intimal hyperplasia and microcalcification

### Liam McQueen; Shameem Ladak; Georgia Layton; Mustafa Zakkar

#### University of Leicester, Leicester, UK

**Correspondence**: Liam McQueen

*Journal of Cardiothoracic Surgery 19(1):* A126

**Objectives:** The long saphenous vein (LSV), frequently utilised in CABG surgery, is known to develop intimal hyperplasia (IH) leading to graft failure. IH is correlated with vascular calcification (VC), to which the matricellular protein osteopontin (OPN) has been implicated at endothelial injury sites in animal studies. Haemodynamics are believed to drive inflammation facilitating VC, IH, and stimulate OPN expression, which could be mitigated by dexamethasone (DEXA), shown to inhibit murine IH. This work aims to assess the role of OPN on VC & IH in humans, whether DEXA attenuates this & whether detection of VC in situ could allow for graft patency monitoring.

**Methods:** LSV segments obtained from CABG patients were subjected to ex vivo perfusion using a bioreactor to mimic arterial conditions for four hours. Tissue culture up to 10 days was required to identify longer-term VC and OPN expression. In both models the tissue was untreated or pre-treated with DEXA (10 ng/mL) for one hour. Gene and protein expression was quantified using qRT-PCR, RNAScope and immunofluorescence. Calcium deposition was identified using Alizarin Red and McNeal’s Tetrachrome staining, visualised using light microscopy and ^18^F autoradiography.

**Results:** OPN expression was significantly upregulated over time and in response to flow, with DEXA pre-treatment suppressing expression by day 10 culture. Calcium staining identified significant calcification over time, predominantly in the intimal and medial layers. DEXA pre-treatment significantly attenuated OPN expression (p < 0.0001) and VC in both models, with day 10 calcification comparable to day 0. Validation via autoradiography confirmed significant reductions in VC in pre-treated samples.

**Conclusions:** DEXA pre-treatment inhibits OPN expression and VC following haemodynamic stimulation and long-term culture, presenting a potential pre-implantation strategy for IH mitigation. VC detection via autoradiography may offer a novel approach to graft patient monitoring in situ.
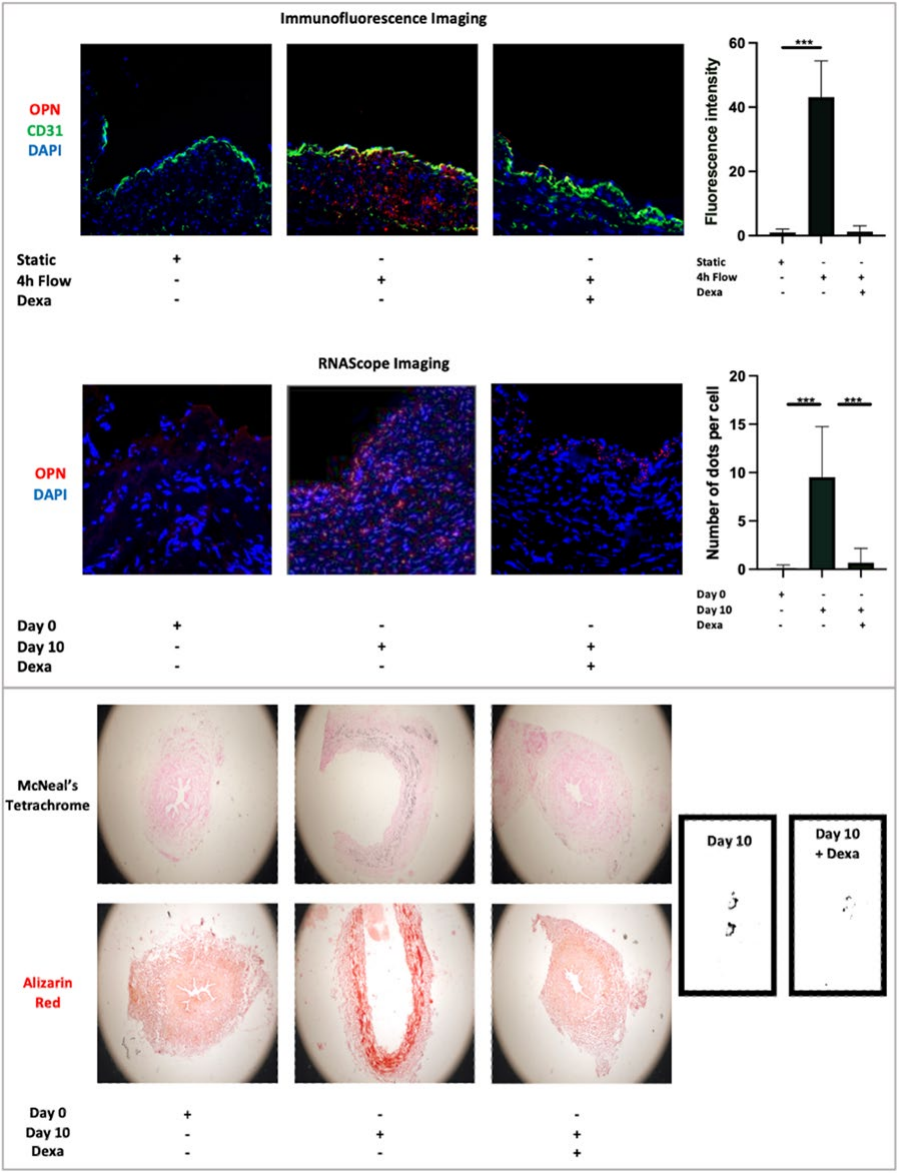


## A127 3D bioprinted hydrogel patches containing cardiac spheroids recover heart function in a mouse model of myocardial infarction

### Christopher Roche^1^; Haiyan Lin^2^; Charles de Bock^3^; Dominik Beck^4^; Meilang Xue^2^; Carmine Gentile^4^

#### ^1^University Hospital of Llandough, Cardiff, UK; ^2^University of Sydney, Sydney, Australia; ^3^University of New South Wales, Sydney, Australia; ^4^University of Technology Sydney, Sydney, Australia

**Correspondence**: Christopher Roche

*Journal of Cardiothoracic Surgery 19(1):* A127

The copyright holder agreed to their image being published under a CCBY 4.0 Open access licence (Fig. 1).

**Fig. 1:**
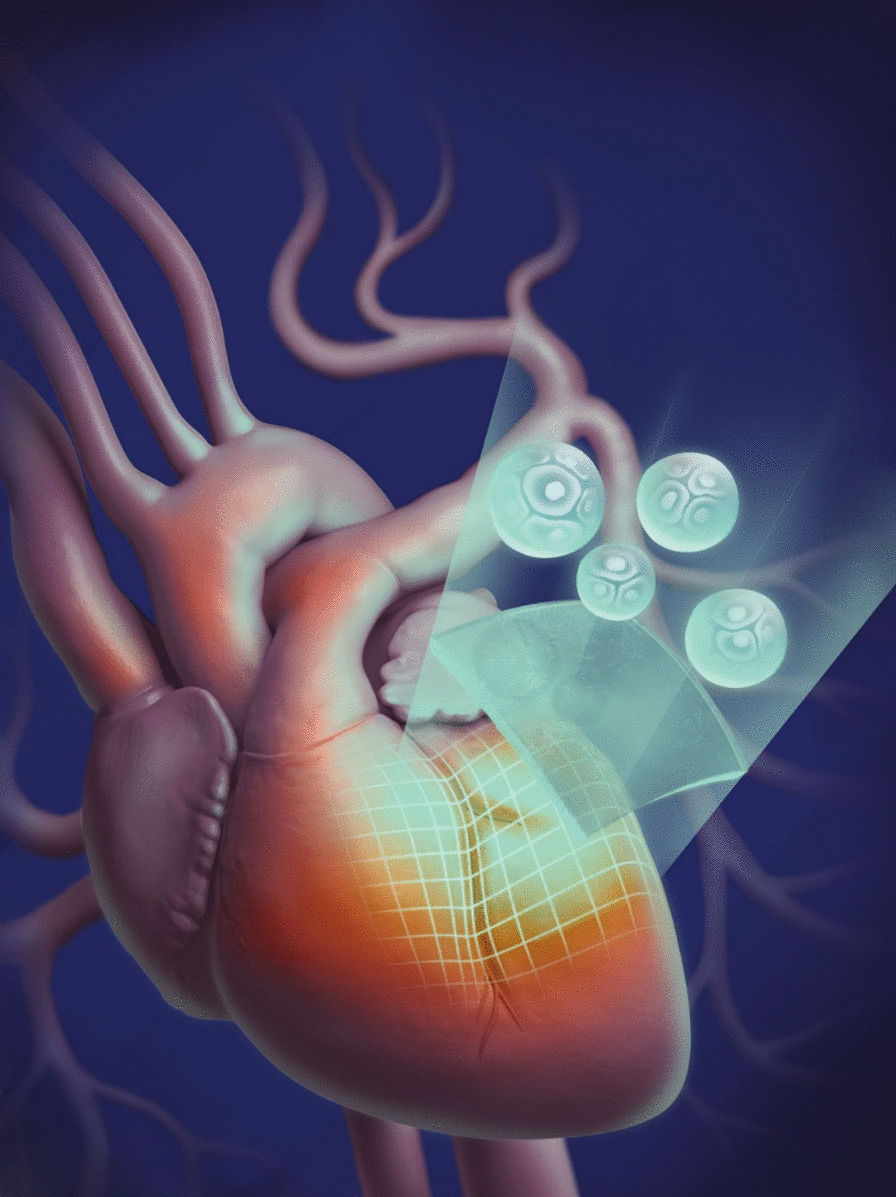
Representative illustration of 3D bioprinted heart patches transplanted to the epicardium for myocardial regeneration. Patches include “vascularised cardiac spheroids” within alginate-gelatin hydrogel. These are microtissues containing induced pluripotent stem cell-derived cardiomyocytes (iCMs), cardiac fibroblasts (CFs) and human coronary artery endothelial cells (HCAECs). Copyright Leo Herson (graphical artist)

Epicardial transplantation of 3D bioprinted patches represents a promising protective strategy against infarction-induced myocardial damage. We previously showed that 3D bioprinted tissues containing cardiac spheroids in alginate/gelatin (AlgGel) hydrogels promoted cell viability/function and endothelial cell tubular self-assembly. Here, we hypothesise that bioprinted cardiac spheroid patches improve cardiac function after myocardial infarction (MI). To determine treatment effects of hydrogel alone or with cells, MI mice were transplanted with: (1) AlgGel acellular patches, (2) AlgGel with freely suspended cardiac cells, (3) AlgGel with cardiac spheroids. We included control MI mice (no treatment) and mice undergoing sham surgery. We performed measurements to 28 days including echocardiography, flow cytometry and transcriptomic analyses. Our results measured median baseline (pre-surgery) left ventricular ejection fraction (LVEF%) for all mice at 66%. Post-surgery, LVEF% was 58% for sham (non-infarcted) and 41% for MI (no treatment) mice. Patch transplantation increased LVEF%: 55% (acellular; p = 0.012), 59% (cells; p = 0.106), 64% (spheroids; p = 0.010). Our flow cytometry analyses demonstrated that cardiac spheroid patches regulated host cardiac tissue immune cell populations. RNAseq transcriptomes demonstrated similar gene expression profiles for sham and mice treated with cardiac spheroid patches. Altogether, our findings identify for the first time which cellular and molecular targets may regulate the mechanisms controlling the recovery following cardiac spheroid patch epicardial transplantation.

## A128 Applied mixed-reality visualisation of patient-specific anatomy in congenital heart surgery

### Garima Bhag; Ahmed Abousteit; Ramesh Kutty; Attilio A Lotto; Ram Dhannapuneni; Phuoc Duong; Rafael Guerrero

#### Alder Hey Children's Hospital, Liverpool, UK

**Correspondence**: Garima Bhag

*Journal of Cardiothoracic Surgery 19(1):* A128

**Objectives:** Evaluation of use in cases when surgeons see and interact with high fidelity 3D anatomic datasets prior to operations. Complex cases included small neonates (smallest − 2.2 kg) with rare coronary abnormalities.

**Methods:** CT and MRI images were used to reconstruct 3D anatomic structures in fine details. Surgeons reviewed anatomic data using conventional case planning, followed by rehearsal in mixed reality (rotating, cropping & annotation) environment (HoloLens 2, Microsoft Inc., USA).

Feedback was gathered using Likert-scale scoring system. Objective bias acknowledged due to prior approval using conventional data analysis.

**Results:** Five surgeons at different seniority approved the use of technology, in assisting further spatial understanding of anatomic structures, in 10 cases. Junior surgeons spent more time interacting with datasets. No side effects (nausea, headache, disorientation) were reported. Junior surgeons appreciate senior surgeon’s perspective and seek mentorship simultaneously. Complex surgical manoeuvres such as coronary button translocation, especially in small neonates were better understood. Exposure experience in rare abnormalities, is rated highly. All surgeons valued ability to rehearse whilst not under stress, translating to the peri-procedural benefit.

**Conclusions:** Innovation using immersive mixed reality visualisation of anatomic structure is perceived as useful in Congenital Heart Surgery. The value is elevated in rare complex cases which may enable surgeons to reduce error and stress level.

Interacting with the heart in Augmented Reality
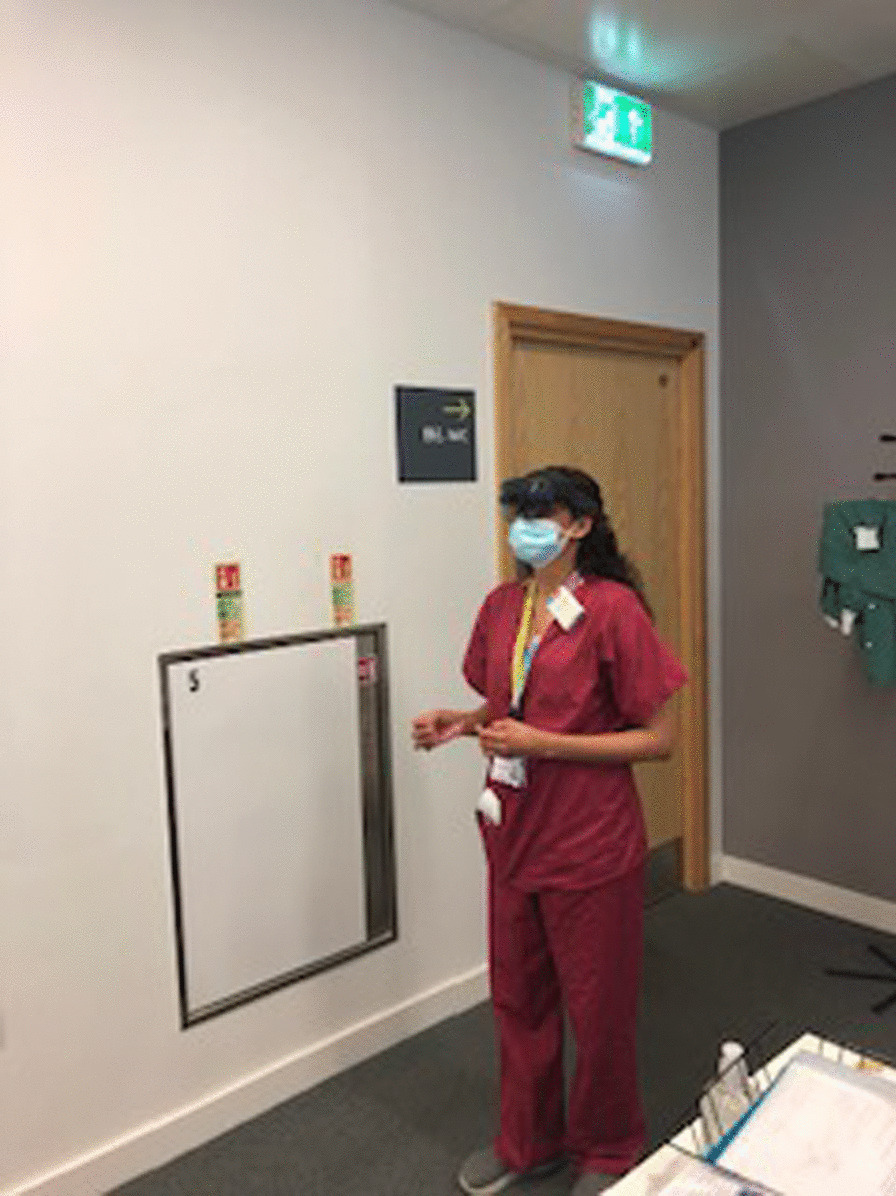


The author can confirm that the person in the figure agreed to publication of their image in an Open Access journal.

## A129 Whiteboard adding colour to the theatre team

### Huzaifa Ahmad; Hannah Merriman; Sridhar Rathinam

#### Department of Thoracic Surgery, Glenfield Hospital, Leicester, UK

**Correspondence**: Huzaifa Ahmad

*Journal of Cardiothoracic Surgery 19(1):* A129

**Objectives:** Communication and team working are integral in theatre environment. Breakdowns in communication are often contributory to medical error in the operating theatre. The WHO team huddle starts with introduction however most staff do not display badges and names are easily missed. We sought to assess whether writing team members' names on a surgical whiteboard, where they remained visible throughout the day improved the perceived quality of communication.

**Methods:** Six-item questionnaires were distributed to members of the thoracic surgical team in the theatre, over a period of one month. These were distributed amongst staff in theatres with names displayed on the whiteboard, as well as theatre without names being displayed. Data is reported summative below.

**Results:** There were 24 respondents. Twenty were regular staff, whilst four were bank staff who were not part of the established team. Overall, 83.3% expressed a preference to continue the practice of writing names on a visible board in future.

**Conclusions:** Majority of the staff agreed that displaying names on the whiteboard helped in overall communication, especially with bank staff who were new to the environment. Amongst the four who disagreed with whiteboard, suggested different means on identifying the staff, for instance, a name batch. However, everyone was in agreement with the concept of identification being displayed while in theatre – making communication more colourful.

## A130 Simulation practices and attitudes in cardiothoracic surgery

### Abdul Badran^1^; Umar Daniyal^2^; Aiman Alzetani^1^

#### ^1^University Hospital Southampton NHS Foundation Trust, Southampton, UK; ^2^University of Southampton, Southampton, UK

**Correspondence**: Abdul Badran

*Journal of Cardiothoracic Surgery 19(1):* A130

**Introduction:** Simulation-based training (SBT) in surgery is an educational activity utilising simulation aides to replicate real-life scenarios. Surgical specialties such as Cardiothoracic Surgery (CTS) can benefit from simulation considering the high-risk and delicate operative procedures and techniques that trainees are expected to learn.

**Aims:** This study aims to assess the feasibility and effectiveness of simulation in CTS, as well as attitudes towards incorporating more simulation into the specialty’s curriculum.

**Methods:** CTS trainees, foundation doctors and medical students were invited to simulation skills sessions. They performed critical skills on synthetic CTS simulation models. Measurements of objective elements such as surgical performance and user feedback were carried out. Pre-session and post-session questionnaires were distributed out within the sessions to each participant to gauge their perspectives on current simulation standards. Evaluations were mainly based on a five-point Likert scale. A national survey was generated and sent out to all trainees in the UK and Ireland.

**Results:** Nine CT trainees, ten medical students and one foundation doctor from across the country took part and completed the questionnaires. Eight CT trainees (88.9%) out of the nine agreed that simulators should be utilised more regularly in surgical training compared to the current standards (p < 0.05). There was also a general trend of participants who had graduated earlier, not necessarily performing better than those who graduated later despite the relationship not being statistically significant. Additional national survey results confirmed a lack of availability of resources and enthusiasm amongst the training cohort.

**Conclusions:** Overall, attitude towards SBT is positive and this specialty would welcome more of its incorporation in its curriculum. Its use, however, is currently limited and requires stronger evidence to assess its feasibility and effectiveness.

**Congenital**

## A131 Anomalous left or right coronary artery from the pulmonary artery: 22 years experience in a single centre

### Ahmed ElSherbini; Muhammad Mustafa; Minji Ho; Gianluca Lucchese; Conal Austin

#### Guy's and St Thomas' NHS Foundation Trust, London, UK

**Correspondence**: Ahmed ElSherbini

*Journal of Cardiothoracic Surgery 19(1):* A131

**Objectives:** Anomalous left or right coronary artery from the pulmonary artery (ALCAPA or ARCAPA) is a potentially life-threatening congenital coronary artery anomaly. This study aims to present the outcomes of surgical repair.

**Methods:** A retrospective analysis was performed for patients who underwent surgical repair between January 2000 and October 2022.

**Results:** A total of 37 patients aged from 26 days to 57 years, with a median age of 1.54 years (neonates = 2, Infants = 15, children < 18y = 13, adults = 7) underwent surgical repair; ALCAPA = 34 (91.9%); ARCAPA = 3 (8.1%). Median follow-up was 6.34 years (mean, 8.56 years; range, 5 months-20.45 years). 36 patients received coronary reimplantation, and one underwent Takeuchi repair. All three ARCAPA patients had concomitant congenital cardiac anomalies, two required VSD closure, and the 3rd had Ross-procedure with aortic coarctation repair. Two patients had a delayed ALCAPA repair after a missed diagnosis having undergone Mitral annuloplasty for a presumed diagnosis of isolated severe mitral regurgitation in infancy (one in childhood and one after 30 years). There was no early mortality. 34 patients (91.89%) were free from reoperation. Three patients required surgical re-intervention, two patients for RVOTO with pulmonary artery patch augmentation and one patient for left main coronary artery stenosis. At the last follow-up, four patients (10.81%) had mild to moderate mitral valve regurgitation, 32 patients (86.49%) had a normal left ventricular function, and five patients (13.51%) had mildly reduced left ventricular function.

**Conclusions:** Experience with coronary reimplantation in Arterial switch operations has popularised repair techniques in ALCAPA and ARCAPA. Our results confirm excellent late myocardial recovery with low levels of re-intervention and stable Mitral function. Generous Pulmonary artery patch augmentation at initial repair may lessen the chance of late RVOTO. Be aware of infantile isolated Mitral insufficiency masquerading ALCAPA.

## A132 Assessing change in left ventricular function following mitral valve replacement in children using global strain analysis

### Clare Stewart^1^; Mark Danton^1^; Emma Finlay^2^

#### ^1^University of Glasgow, Glasgow, UK; ^2^Royal Hospital for Children, Glasgow, UK

**Correspondence**: Clare Stewart

*Journal of Cardiothoracic Surgery 19(1):* A132

**Objectives:** This observational cohort study investigated whether children and adolescents experience a significant and non-recoverable decline in LV contractility function following MVR, a relationship that has been established in adults.

**Methods:** This study investigated 22 patients (14:8 M:F) mean age 56 ± 70 months (3 months–18 yrs) who underwent bileaflet mechanical MVR (commonest size 21 mm, range 16–31) from 2002 to 2021. Regurgitation was present in 17 (77%) of these patients, stenosis in three (14%), and mixed MV dysfunction in two (9%). Congenital aetiology was the most common pathology, present in 17 patients (77%). LV contractility pre and post MVR was evaluated using echo derived % global longitudinal strain (GLS); a measure of myocardial contractility that is relatively insensitive to loading conditions, where normal function: − 20% ± 2; mild impairment − 18 to − 15, moderate impairment − 15 to − 10, and severe − 10 to 0. GLS was obtained pre-op (15 days prior), early post-op (25 days after), and late post-op (11 months after). % GLS was expressed as mean ± SD, and hypothesis testing was used to determine significances in GLS change between these timepoints.

**Results:** Pre-MVR GLS was normal: − 19.0% ± 6.6. Post MVR demonstrated moderate impairment: Early post-MVR GLS − 13.3% ± 4.1, and late post-MVR GLS − 14.6% ± 3.5. The reduction in LV contractility was significant between pre- and early post-MVR echos (mean GLS decline: 5.7 ± 6.8, p = 0.001), and between pre- and later post-MVR (GLS decline: 4.4 ± 6.3, p = 0.014). At the later evaluation some contractile recovery was evident but non-significant in this cohort and timeframe (P = 0.094).

**Conclusions:** GLS analysis has shown that undergoing MVR leads to a moderate impairment of LV function in children after surgery. The trend to achieve functional recovery in the later analysis however suggests that there is potential for ventricular adaptation in children (Fig. 1).

**Fig. 1:**
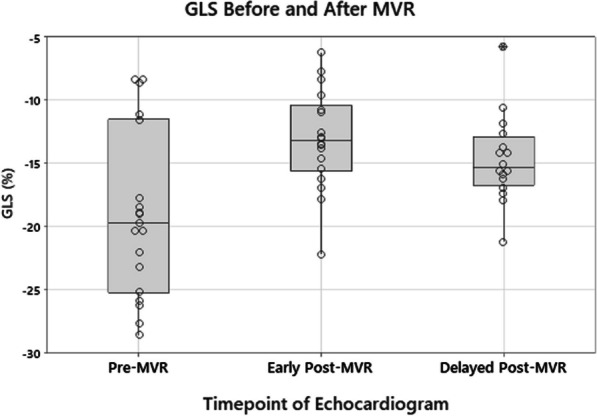
Global Longitudinal Strain on echocardiogram before and after Mitral Valve Replacement

## A133 Anomalous aortic origin of right coronary artery: outcomes of unroofing of the intramural segment

### Ahmed ElSherbini; Muhammad Mustafa; Minji Ho; Muhammad Hebala; Gianluca Lucchese; Conal Austin

#### Guy's and St Thomas' NHS Foundation Trust, London, UK

**Correspondence**: Ahmed ElSherbini

*Journal of Cardiothoracic Surgery 19(1):* A133

**Objectives:** Anomalous aortic origin of the right coronary artery with interarterial course is a risk factor for sudden death and significant cardiac complications. Different surgical repair techniques have been adopted with no superiority of one. We describe our experience with the unroofing technique results at midterm follow-up.

**Methods:** A retrospective review of all right coronary artery unroofing procedures performed in a single institution between 1st January 2007 and 31st October 2022 was performed. A cohort of six patients with anomalous origin of the right coronary artery from the opposite sinus was identified.

**Results:** The mean age was 42 (range, 17–61 years). Four patients presented with dyspnoea, and angina, and two with acute myocardial infarction. A definitive diagnosis was achieved with coronary CT angiography. In all cases, the anomalous origin of the right coronary artery from the left sinus had an intramural course. At the operation, the intramural segment of the right coronary artery was unroofed in all patients. After a median follow-up of 4.74 years (mean, 6.21 years; range, 2.26–15.83 years), all patients are asymptomatic and have returned to total exercise capacity. There was no early mortality. Freedom from reintervention was 100%. All patients had normal left ventricular function at the last follow-up.

**Conclusions:** Definitive surgery is indicated in the symptomatic anomalous aortic origin of the right coronary artery with interarterial course. The unroofing technique provides an excellent physiological and anatomical repair, eliminates a slit-like ostium, avoids compression of the coronary artery between the aorta and the pulmonary artery, and is associated with excellent midterm outcomes.

## A134 Mechanical mitral valve replacement in children: Is patient or prosthesis size the major determinant of risk?

### Ahmed Aboelenen; John Stickley; Nigel Drury; Milind Chaudhari; Vinay Bhole; Natasha Khan; Tim Jones; Phil Botha

#### Birmingham Children Hospital, Birmingham, UK

**Correspondence**: Ahmed Aboelenen

*Journal of Cardiothoracic Surgery 19(1):* A134

**Objectives:** Recent reports suggest that oversized mechanical mitral valve replacement (MVR) in children increases the risk of mortality and morbidity. We investigated whether risks are associated with patient age / somatic size, or the size of the prosthesis used.

**Methods:** All mechanical MVR (age < 18) between 1988 and 2022. Survival, freedom from replacement and thrombotic/ bleeding complications were compared between patients with first MVR in the first year of life and older children, and between annular and supra-annular implants using competing risks methodology.

**Results:** 77 children underwent 103 MVR; 2 neonates, 44 infants and 57 age 1–18 years at the time of implant. Median prosthesis size / patient weight ratio was 2.2 (Range 0.5–5.2 mm/kg) and 53% of implants were supra-annular. Median duration of follow-up was 6.2 years. Procedural 90-day mortality was 14% overall: 22% in infants and 7% in older children (p = 0.042). Ninety-day mortality was 22% from 1988–2006, and 6% since 2006, (p = 0.023). Survival was significantly lower in patients with Shone’s complex (n = 18) as compared to other diagnoses (Log-rank p = 0.038). Freedom from major bleeding / thrombotic events was 78% at 10 years post MVR. At 10 years, overall survival was 63%, and 95% of patients had either died or required repeat valve replacement by 17 years (Fig. 1). In competing risks analysis of redo-MVR and death, both the infant age group (HR 3.22, 95%CI 1.07–9.75, p = 0.038) and era of surgery (HR 2.12, 95%CI 1.01–4.44, p = 0.046) were significantly associated with these outcomes, but valve size (mm/kg body weight) and supra-annular position of implant were not.

**Conclusions**: The increased risk after MVR in small children is inherent in their somatic size and likely the severity of illness, not the size of the mechanical prosthesis used. Supra-annular implantation is a useful technique for children with a small annulus with good operative survival and infrequent adverse events.
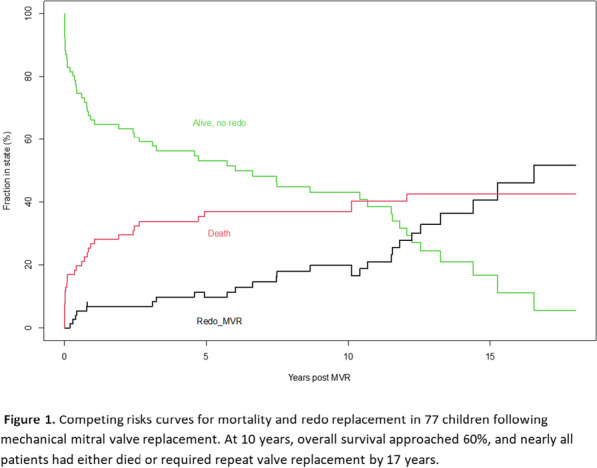


## A135 Inadequate ductal resection is a strong predictive factor for arch obstruction after norwood procedure, regardless of surgical technique

### Ahmed ElSherbini; Muhammad Mustafa; Caner Salih; Conal Austin

#### Guy's and St Thomas' NHS Foundation Trust, London, UK

**Correspondence**: Ahmed ElSherbini

*Journal of Cardiothoracic Surgery 19(1):* A135

**Purpose:** Recurrent aortic arch obstruction following the Norwood procedure is a recognised complication in 9–37% of patients despite continuous advances in surgical technique. This study tracks the incidence of recurrent arch obstruction and its correlation to the surgical techniques following the Norwood procedure in a large cohort of patients.

**Methods:** A retrospective review of all Norwood procedures performed in a single institution between 1st January 2005 and 31st December 2021 was performed. Patients whose first definitive operation was the hybrid procedure or bilateral pulmonary artery banding were excluded. Analysis was performed in patients who survived to initial assessment (catheter or MRI). Patients were divided into two groups based on the surgical techniques. Recurrent aortic arch obstruction was defined as an increased distal arch velocity > 2.5 m/s on echocardiogram with diastolic tail and/or narrowing on cardiac MRI.

**Results:** 154 patients who had the Norwood procedure and survived the initial assessment prior to Hemi-Fontan or Glenn. Two distinctive surgical techniques for aortic arch repair were noted: Technique A in 112 patients (72.72%) using extensive ductus excision required transection of the descending aorta and re-anastomosis to the transverse arch before arch augmentation. Technique B in 42 patients (27.27%) was to completely excise the ductus arteriosus from the isthmus of the aortic arch, extend the incision in the descending aorta well past the ductus insertion site to avoid ductal contraction and recurrent coarctation and then peel off any ductal tissue or coarctation shelf from the posterior wall of the aortic isthmus before arch augmentation. Arch re-intervention was required in four patients (2.59%) (Technique A = 3, Technique B = 1).

**Conclusions:** Extremely low rates of re-intervention after the Norwood procedure can be achieved when the surgical technique focuses on adequate removal of ductal tissue regardless of surgical technique.

## A136 Elective delayed sternal closure strategy following pulmonary artery banding in complex congenital heart defects

### Amr Ashry^1^; Sathyan Gnanalingham^2^; Rachel Tan^2^; Ramesh Kutty^2^; Ram Dhannapuneni^2^; Rafael Guerrero^2^; Attilio A Lotto^2^

#### ^1^Assiut University Hospital, Assiut, Egypt; ^2^Alder Hey Children's Hospital, Liverpool, UK

**Correspondence**: Amr Ashry

*Journal of Cardiothoracic Surgery 19(1):* A136

**Objectives:** Perfect adjustment of Pulmonary Artery Banding (PAB) could be difficult, particularly in complex CHD, with the risk of redo operation to adjust the PAB. We started a strategy of delayed chest closure in order to readjust the PAB in the first 24–48 h postoperatively. The objective of our study was to evaluate the outcomes of elective delayed sternal closure following PAB with emphasis on reoperation rate.

**Methods:** Retrospective study for paediatric patients who underwent pulmonary artery banding via median sternotomy with delayed chest closure at our institution between 2016 and 2022. Outcomes were to assess postoperative complications, like reoperation rate, mortality, wound infection, ECMO, ICU and hospital stay.

**Results:** 110 PAB cases either isolated or associated with other procedures were identified. 52 patients (47.3%) had closed chest and 58 cases (the study group) had delayed chest closure for various reasons (52.7%). Median age and weight were 26 days (IQR 12.3–52.8) and 3.5 kg (IQR 3.2–4.1). There were 23 isolated PAB (39.7%), mainly for VSD management and 35 cases (60.3%) of PAB with other procedures, mostly in association with hypoplastic aortic arch (HAA) repair. Median ICU stay and postoperative hospital stay were seven days (IQR 5–13.8) and 17.5 days (IQR 10.4–40.2), respectively. 30-days hospital mortality occurred in two cases (3.4%). Debridement for sternal wound infection was done in two cases (3.4%). 27 cases (46.6%) required PAB adjustment before chest closure. Median PAB velocity at operation day and at discharge were 3.05 m/s (IQR 2.4–3.4) and 3.95 m/s (IQR 3.4–4.2), respectively. No patients needed late redo sternotomy to adjust the PAB. One patient (1.7%) needed intra-operative ECMO, while four cases (6.9%) required post-operative ECMO.

**Conclusions**: Delayed chest closure represents a successful strategy in complex CHD such as in single Ventricle or in association with HAA, reducing the need for redo sternotomy to adjust the PAB.

## A137 Micromechanical properties of animal and synthetically derived materials used in congenital cardiac surgery repairs

### Attilio A Lotto^1^; Amr Ashry^2^; Isobelle Harrison^3^; Celine Chikuhwa^3^; Riaz Akhtar^4^

#### ^1^Alder Hey Children Hospital, Liverpool, UK; ^2^Assiut University Hospital, Assiut, Egypt; ^3^School of Engineering, Liverpool, UK; ^4^Liverpool Centre for Cardiovascular Science, Liverpool, UK

**Correspondence**: Amr Ashry

*Journal of Cardiothoracic Surgery 19(1):* A137

**Background:** Repair of congenitally hypoplastic heart vessels relies on the use of heterologous patches to surgically increase vessels size. The mechanical mismatch between animal or synthetic derived materials and the native tissue may be responsible for distortion and turbulence of the pulse wave propagation and associated with the poor clinical outcomes and recurrent vessel stenosis.

**Methods:** Oscillatory nanoindentation was conducted with an ultra-low load indenter head utilizing a 100 µm flat punch indenter to characterize the mechanical properties of synthetic patch materials and xenograft patch materials. Ten indents were performed on each sample with three replicates per patch type. The synthetic patches that were utilized were Dacron (0.61 mm thickness), ePTFE (0.4 mm thickness), and ePTFE (1 mm thickness). For the xenograft patch materials, bovine pericardium (BP) samples of thickness 0.2, 0.3 and 0.5 mm and a bovine jugular vein (BJV) conduit were used. The data were compared with native ovine aorta properties.

**Results:** The Dacron graft exhibited a mean elastic modulus (E) of 2.37 ± 0.54 MPa in comparison to 5.84 ± 1.34 MPa, and 5.27 ± 1.78 MPa for ePTFE (0.4 mm) and ePTFE (1 mm), respectively. The biological patches were significantly lower in terms of stiffness with a mean E of 0.12 ± 0.06 MPa for BJV and 0.25 ± 0.046, 0.155 ± 0.077 and 0.165 ± 0.054 MPa for BP of 0.2, 0.3 and 0.5 mm thickness, respectively. Previous reports from our group have shown an E value for the ovine aorta ranging from 0.08 to 0.1 MPa.

**Conclusions**: Our results show that BJV conduit have the closest mechanical properties to the aorta followed by the BP patches. Hence, animal derived patches have a lower mechanical mismatch than synthetic grafts and may lead to better outcomes than synthetic grafts.

**Nursing and AHP Forum**

## A138 Is there a place for simulation in enhancing leadership skills in the clinical area? Views of trainee advanced nurse practitioners

### Maura Sale

#### Royal Papworth Hospital, Cambridge, UK

**Correspondence**: Maura Sale

*Journal of Cardiothoracic Surgery 19(1):* A138

**Background:** The changing face of the NHS is in part due to a growing, aging population, the existence of more long-term conditions and a reduction in junior doctor working hours. Supported by HEE (2017) an advanced level of nursing practice was implemented to support these shortfalls in healthcare delivery. Its pivotal workplace curriculums are established to support the education and development of this level of practice. To date, there is paucity of evidence to suggest simulation as a pedagogical approach supports education and development leadership skills at this level of nursing practice. More importantly if these skills are the transferable to clinical practice.

**Objectives:** To explore the benefits of simulation in enhancing trainee advanced nurse practitioner leadership skills and their transferability to clinical practice.

**Design:** Supporting a constructivist world view a qualitative research design informed by phenomenological methodology was used.

**Methods:** Trainee ANPs attended mandatory workplace simulation sessions focusing on leadership skills over a five-month period. Participants were recruited through purposive sampling from this group and invited to attend a virtual focus group two weeks after the final simulated session.

**Results:** Four themes were identified from the data: safety; motivation; acquisition of knowledge & confidence; and transferability of skills to clinical practice.

**Conclusions:** Trainee ANPs believed simulated practice provided a safe space for learning, gave them the knowledge and confidence they required to lead on patient care in addition to facilitating the transferability of skills to clinical practice. It is recommended simulation should be utilized as a pedagogical approach to support Trainee ANP education & development. Further research is needed using different methodological approaches to objectively quantify the benefits of simulated learning in trainee ANP workplace curriculums.

## A139 Nurses as inventors: the cardio-adjustable thoracic support (CATS) vest

### Rosalie Magboo^1^; Sandra Hutton^2^; Carlos Morais^3^; Melissa Rochon^4^

#### ^1^St Bartholomew's Hospital, London, UK; ^2^Oxford University Hospital, Oxford, UK; ^3^Guy's & St Thomas' NHS Foundation Trust, London, UK; ^4^Royal Brompton and Harefield Hospital, London, UK

**Correspondence**: Rosalie Magboo

*Journal of Cardiothoracic Surgery 19(1):* A139

**Objective:** A number of evidence suggest that chest support may play a significant role in reducing pulmonary complications and pain and may potentially reduce the risk of surgical site infection following cardiac surgery. The objective of this nurse-led quality improvement project was to design a comfortable, effective and user-friendly unisex chest support which was machine washable at high temperatures.

**Methods:** Between November 2019 to August 2022, nurses at four hospital sites in England applied the Plan-Do-Study-Act (PDSA) cycles to develop and design the CATS vest. Different prototypes were done using iterative tests of change. Prototypes 6–12, which incorporated 84 iterative small tests of change cycles, were trialled with cardiac patients. Different CATS vest sizes (XS-XXXL) with two depths (20 and 26 cm) were also tested. Feedback from staff and patients was obtained using a five-point Likert scales for agreement based on different elements. Adjustments to the vest design were made after learning was obtained from each cycle. The final PDSA cycle for Prototype 13 was tested in 64 patients at three centres.

**Results:** The final design included Velcro fastens with a ‘glove style’ for ease of fit, a pocket to hold telemetry or negative pressure battery pack, and was made of breathable supportive material which was machine washable at 70 degrees Celsius.

Clinical evaluation scores for the final prototype were above 4 out of 5 for domains including support, security and recommending to others. The result was similar when compared for both female and male as well as patients with BMI ≥ 30 and < 30.

**Conclusions:** A nurse-led unisex chest support was developed using PDSA cycles that may be useful to support patients during recovery period. Patent and design rights applications were accepted, pending approval. Future plans include clinical trial for the CATS vest and assessment of its impact on the prevention of cardiac surgery complications.

## A140 Living with marfan syndrome: preliminary results of the health-related quality of life and psychosocial effects of the aortovascular diagnosis

### Rosalie Magboo^1^; Aung Oo^1^; Gareth Owens^2^; Julie Sanders^1^

#### ^1^St Bartholomew's Hospital, London, UK; ^2^Aortic Dissection Awareness UK and Ireland

**Correspondence**: Rosalie Magboo

*Journal of Cardiothoracic Surgery 19(1):* A140

**Objective:** Marfan Syndrome (MFS) is a rare autosomal-dominant connective tissue disorder with serious and life-threatening cardiovascular manifestations. Despite the severity of this life-long condition, there is very little evidence on the health-related quality of life (HRQoL) and psychosocial effects of living with MFS. This study is one part of a mixed methods larger project aiming to identify the HRQoL and the psychosocial effects of the diagnosis of aortovascular manifestation in MFS patients and describe it in the UK population for the first time.

**Methods:** This single site qualitative exploratory study was designed to identify quality of life issues affected by having MFS from patients’ lived experience. Interviews, lasting up to one hour, were conducted using a semi-structured interview schedule. Each interview was audio-taped and transcribed verbatim. Data saturation assessment was done using the Guest et al. (1) method. Data analysis was undertaken using the Framework method, which follows a seven-stage process.

**Results:** Twenty interviews were undertaken, transcribed and analysed. Preliminary results revealed that the HRQoL and psychosocial effects of the aortovascular diagnosis include: loss of personal efficacy, social isolation, health anxiety, financial burden, feeling depressed, loss of self-esteem and worry in building a family. However, patients also expressed that the diagnosis led to disease acceptance, constructive engagement in coping with the condition and self-acceptance.

**Conclusions:** These results assist in better understanding the HRQoL and psychosocial factors that affect patients with MFS. This information can support the development of a patient care pathway that includes HRQoL and psychosocial aspects of care to provide a holistic and comprehensive service for this patient group.

Reference

Guest G, Namey E, Chen M. A simple method to assess and report thematic saturation in qualitative research. (2020) PLoS One. 15(5).

## A141 Day zero mobilisation: post thoracic surgery. Enhanced recovery after surgery, the aggregation of marginal gains

### Lydon Metson

#### James Cook University Hospital/South Tees Hospitals NHS Foundation Trust, Middlesbrough, UK

**Correspondence**: Lydon Metson

*Journal of Cardiothoracic Surgery 19(1):* A141

1. The intuitive benefits of early mobilisation are broadly accepted across medical professions, as a result, it is identified as a central component in both international ERAS guidelines and local ERAS protocols.

There are 45 enhanced recovery components covering topics related to preadmission, admission, intraoperative care and postoperative care. Small improvements in each of these components equate to significant improvements in patient outcomes overall. Many of the components are tailored to facilitate early mobilisation.

This service improvement initiative endeavours to illustrate that, a prioritised, physiotherapy led day zero mobilisation service can improve compliance, efficiency and effectiveness in relation to the earliest possible mobilisation of patients following Thoracic Surgery.

2. The initiative aims to mobilisie all patients admitted to HDU following Thoracic Surgery. Mobilisation standards were identified as sitting in chair on day of surgery, walking 10 m on day of surgery, first mobilisation < 6 h from return to HDU.

3. To date 67% of patients have been mobilised in < 6 h on day of surgery. 58% of these patients mobilised > 10 m. 93% of the 90 patients successfully mobilised in < 6 h sat out in their chair on day of surgery.

54% of those patients not mobilised was due to late return from theatre, despite extended physiotherapy working hours to capture patients returning from theatre.

4. ERAS principles emphasise the importance of early mobilisation. This project has demonstrated that a physiotherapy led, day zero mobilisation service facilitates the mobilisation of almost 70% of Thoracic Surgery patients admitted to HDU on the day of surgery. Any improvements in service provision plays an important role in the "aggregation of marginal gains" for patient outcomes. Further improvements could be made by amending day zero service provision to address the large number of patients not mobilised as they returned late from theatre.

## A142 Determining physiotherapy pathways and sternal precautions protocols for post cardiac surgery patients in the UK

### Alicia Page; Charlotte Milligan

#### Royal Papworth Hospital NHS Foundation Trust, Cambridge, UK

**Correspondence**: Charlotte Milligan

*Journal of Cardiothoracic Surgery 19(1):* A142

**Objective:** Coronary artery bypass graft (CABG) and cardiac valve repair/replacement surgery are among the most common surgeries. One aspect of cardiac surgical care that falls outside evidence-based practice is when a physiotherapist should review these patients, the frequency of review, and use of sternal precautions. We conducted a survey to investigate physiotherapist involvement with this patient group to identify cardiac surgery pathways and protocols at cardiac centres in the UK.

**Methods:** A mixed methods questionnaire was designed online, consisting of 10 questions capturing data on day one of physiotherapy review, frequency of review, if a screening tool is used and who within the physiotherapy team can use this tool. We also asked if sternal precautions were used. The questionnaire was circulated via Twitter.

**Results:** 17 responses from across the UK were received. 80% of physiotherapists reviewed patients on day one following cardiac surgery and 55% continued to review these once daily for their admission. 45% reported they use screening tool from day one post-surgery, most requiring a band five physiotherapist or above to use the screening tool. 100% of responses reported that other professionals took an active role with rehabilitation, with 64% specifically reporting that nursing staff would assist with mobilisation. 73% reported they used standard sternal precautions (no pushing, pulling, or lifting for 12 weeks), 9% using ‘Keep Your Move in the Tube’ (KYMITT) and 18% using no sternal precautions.

**Conclusions:** Most physiotherapists report using formal sternal precautions however there are two NHS Trusts who are starting to introduce KYMITT, which may change the frequency of physiotherapy reviews required.

This data has provided a snapshot of how physiotherapists screen and review patients following cardiac surgery in the UK. Further research is needed to establish an evidence base for physiotherapists to review and treat patients post sternotomy.

## A143 Informing evidence-based best practice in cardiac surgery care: a CONNECT and ctsnet collaboration

### Julie Sanders^1^; Rochelle Wynne^1^; Suzanne Fredericks^1^; Tara Bartley^2^; Jill Ley^2^

#### ^1^on behalf of CONNECT; ^2^on behalf of CTSNet

**Correspondence**: Julie Sanders

*Journal of Cardiothoracic Surgery 19(1):* A143

**Objectives:** The Objectives of CONNECT (The **C**ardiac surgery internati**ON**al **N**ursing and alli**E**d professional resear**C**h network) and CTSNet are aligned – to connect the global cardiothoracic community and to reduce the variability, disparity and inequity in patient access and outcomes. Thus, a collaboration to develop and deliver a webinar series aiming to present the best available evidence in key areas of cardiothoracic patient care was established.

**Methods:** A series of seven webinars has been devised considering core elements of nursing and allied professional cardiothoracic patient care. Webinars on sex-based differences, pain, surgical site infection, patient reported outcomes and using research in practice will be delivered by CONNECT members and preceded by an introductory webinar delivered by the CTSNet editors and close with a webinar detailing the work of CONNECT in future evidence-building. All webinars will be pre-recorded and will be released sequentially in early 2023. The series will conclude with a live webinar with an opportunity for questions to the CTSNet and CONNECT panel.

**Results:** We aim to present at SCTS 2023 excerpts from the series, including the key messages from each webinar, and the importance of evidence-based practice in nursing and allied professional cardiac surgery care.

**Conclusions:** Collaborations are necessary to deliver the ambition of improving patient outcomes and reducing inequality in cardiac surgical care globally. This CONNECT and CTSNet collaboration is a first step in providing a joint online resource of best evidence in key areas of nursing and allied professional cardiac surgery care.

## A144 Early discharge after temporary epicardial pacing wire removal: a retrospective cohort study

### Liril Jacob^1^; Vito Domenico Bruno^1^; Debbie Cross^2^

#### ^1^Bristol Heart Institute, Bristol, UK; ^2^University of the West of England, Bristol, UK

**Correspondence**: Liril Jacob

*Journal of Cardiothoracic Surgery 19(1):* A144

**Background:** Insertion of temporary epicardial pacing wires is a common procedure following cardiac surgery. Complications related to their removal, though rare, can be fatal. There are no nationally recognised guidelines on the removal of pacing wires or safe discharge thereafter.

**Aim:** Evaluate the safety of discharging stable cardiac surgery patients, who meet all other discharge criteria, within four to twenty-four hours after epicardial pacing wire removal.

**Methods:** A single-centre retrospective cohort study was conducted on all consecutive cardiac surgery patients who underwent temporary pacing wire insertion at a tertiary centre for cardiac surgery (n = 250). Patient records were retrospectively reviewed to extract and collate variables related to the procedure as well as acute and long-term adverse outcomes.

**Results:** No significant difference was observed for acute (p = 0.646) or long-term complications (p = 0.118) between patients discharged before 24 h from removal and those discharged later. Acute complications showed significantly greater incidence in cases with moderate/severe resistance to removal (p < 0.001). Patients with INR > 2 at removal showed significantly greater long-term complications (40.9% vs 16.2%, p = 0.02).

**Conclusions:** The study showed the practice of discharging patients within 24 h after pacing wire removal, if all other discharge criteria are met, is safe. High resistance and elevated INR (> 2) at the time of removal are independent predictors of acute and long-term complications. Such patients should be closely monitored after removal and might benefit from delayed discharge. Further research should be conducted to make the study’s results generalisable and to aid formulation of guidelines to standardise practice.

## A145 Introduction of physiotherapy review to assessment of lung transplant candidates

### Emma Matthews

#### Royal Papworth Hospital, Cambridge, UK

**Correspondence**: Emma Matthews

*Journal of Cardiothoracic Surgery 19(1):* A145

**Objectives:** Patients attending our centre for lung transplant assessment are routinely seen by multiple members of the MDT however physiotherapy review was only on an adhoc basis dependent on patient availability. In July–September 2019, only 4 out 15 assessment patients were reviewed by a physiotherapist. Therefore, an allocated physiotherapy slot was added to the assessment timetable. The Objectives of this review are to assess:

1. If the addition of an allocated timeslot increased the frequency of physiotherapy review
2. The role of the physiotherapist within lung transplant assessment
3. Average baseline score on SPPB at transplant assessment

**Methods:** In July 2021, an allocated physiotherapy timeslot was added to each lung transplant assessment timetable. A physiotherapy assessment protocol was agreed outlining important topics to be discussed, and following discussion with other transplant centres, the Short Physical Performance Battery (SPPB) was utilised to assess baseline frailty with a score range of 0–12 (categorising patients as minimally, mildly, moderately or severely frail).

**Results:** Between July 2021 and July 2022, 70 patients were admitted for 3-day lung transplant assessments. Of these 96% were reviewed by the physiotherapy team. Of the three patients not reviewed, two were not available at the time of physiotherapy review, whilst the 3rd was admitted under the care of another clinical team.

Education was provided on numerous topics outlined below (Table 1).

**Table 1:** Frequency of education topics covered in physiotherapy lung transplant assessment

Education topic	Number of patients (%)
Transplant Preparation	72 (100%)
Airway Clearance	9 (12.5%)
Exercise	56 (78%)
Breathlessness Management	21 (29%)
Oxygen	19 (26%)
Incontinence	2 (3%)
Cough Suppression	8 (11%)

SPPB were completed with 97% of patients. The mean score was 8.5, suggesting mild frailty. Those without an SPPB had been deemed too short of breath at rest to complete this assessment.

**Conclusions:** Allocation of a specific timeslot within the lung transplant assessment pathway significantly increased the frequency of physiotherapy review. Specialist Physiotherapists can offer a range of education as part of the transplant assessment process, particularly in relation to preparation for transplant and exercise.

## A146 Withdrawing physiotherapy review of routine cardiac surgery patients on post op day 2

### Emma Matthews; Florence Edwards

#### Royal Papworth Hospital, Cambridge, UK

**Correspondence**: Emma Matthews

*Journal of Cardiothoracic Surgery 19(1):* A146

**Objectives:** Historically, Cardiac Surgery patients have been screened for physiotherapy (PT) input on day 1 post operatively (POD1) and then seen each weekday of their admission (with additional input over weekends if indicated). Following increasing observation of excellent nursing care on POD2, alongside attachments limiting mobility progression, we trialled withdrawing PT review of routine POD2 patients. This allowed time to be spent with patients requiring more intensive PT input. Whether a patient is routine is determined by a screening process on critical care.

The Objectives of this review are to assess:

1. If withdrawal of POD2 PT review was associated with a delay in PT discharge beyond their medical fit date
2. Discharge destination of these patients

**Method:** Patients screened out on critical care who had transferred to the ward on POD1 did not receive PT input on POD2. Nursing staff continued their usual practice of assisting the patient out of bed, and mobilising to the bathroom as appropriate. PT then commenced regular reviews as per current practice from POD3 until discharge (excluding weekends unless clinical need indicated review). If the nursing staff had any mobility or respiratory concerns for these patients, they could verbally refer them to the physio team, who would then assess.

Data was collected for 10 weeks following the implementation of this project.

**Results:** 86 patients were screened out by PT and transferred to the surgical wards on POD1. Of these, 1 was referred for POD2 review. 3 (3.5%) were medically fit for discharge before they were safe for PT discharge. The remaining 83 patients were all discharged from PT on the same day as (28%) or at least 1 day before (68.5%, with a mean of 1.5 days) they were medically fit. 85 (98.8%) patients were discharged home, with 1 (1.2%) referred to their local hospital for medical needs.

**Conclusions:** For most patients, reducing PT review on POD2 did not adversely affect their length of stay or discharge destination.

## A147 Use of a novel patient orientated technology application to streamline wound surveillance after cardiac surgery

### Emma Jordan; John McShane; Zahra Jahangir; Ishtiaq Ahmed

#### University Hospitals Sussex, Brighton, UK

**Correspondence**: Emma Jordan

*Journal of Cardiothoracic Surgery 19(1):* A147

**Objectives:** This project aims to transform the way nurse-led surgical site infection (SSI) surveillance is delivered. The many innovative methods employed to deliver care during Covid has highlighted the importance and impact of visual data in the provision of care especially once patients are back in the community This project will enable SSI surveillance nursing teams to leverage visual data to facilitate surgical site infection detection.

**Methods:** A successful funding bid for a grant from the Burdett trust enabled this nurse delivered project to take place. Isla is a patient-centred progressive web application which enables the secure submission of visual data to the patient’s record. Both clinicians and patients can contribute to the patient’s record. Photos are recorded on the app and patients also submit follow up data and clinical review notes triggered by SMS reminders.

**Results:** Initial work on ensuring compliance with Data protection Impact Assessment (DPIA) and secure governance around cloud storage is now complete. Over the next six months data will be collected on post discharge wound surveillance including interventions necessary.

**Conclusions:** Leveraging visual data and digital innovation to provide quality care will result in a reduction of unnecessary follow ups due to detection of infection sooner and more efficiently and effectively report on SSI while empowering patients to contribute to their own patient record. This project aligns with the NHS Long Term Plan, particularly with regards to "making better use of data and digital technology".

## A148 Creating a national network for physiotherapist's working in thoracic surgery in the UK and Ireland

### Michelle Gibb^1^; Zoe Barrett-Brown^2^

#### ^1^University Hospitals of Leicester, Glenfield Hospital, UK; ^2^Royal Papworth Hospital NHS Foundation Trust, Cambridge, UK

**Correspondence**: Michelle Gibb; Zoe Barrett-Brown

*Journal of Cardiothoracic Surgery 19(1):* A148

**Introduction:** Physiotherapists are vital members of the thoracic surgery MDT and have an important role throughout the thoracic patient’s surgical journey. Within the physiotherapy profession specialist networks have been successful, allowing for expert and multicentre collaboration. Previously there has not been a specialist thoracic network for physiotherapists. Within the UK there are 45 Thoracic surgery units within the UK and Ireland (SCTS 2022, Hospital finder).

**Objectives:** We sought to contact each thoracic unit and gain members to join the network. The objectives of the thoracic network were:

1. To gain a Physiotherapist representative from each unit.
2. Use the network for benchmarking and peer support.

**Methods:** We reached out to trusts across the UK and Ireland and used emails and social media to find physiotherapists who wanted to join the network. Once we had established contact details, an initial meeting was arranged via Microsoft teams. Introductions were made and a central database created with each centres details and demographics. We agreed the meetings would run quarterly and each session should have a key theme for benching marking, service improvement and peer support purposes.

**Results:** The thoracic network currently have over 50 members from 27 units. Since the formation of the network, three virtual meetings have taken place. The key themes have been staffing and service provision, prehabilitation and ERAS. The meetings have been well attended with members presenting and sharing their work with peers in the group.

**Conclusions:** The formation of the thoracic surgery network for physiotherapists has been well received as a great platform for peer support and expert collaboration. We hope to gain representation from all thoracic surgery units across the UK.

## A149 An MSSA eradication re-audit within in house urgent cardiac surgery patients

### Katy Wood; Salima Rehman

#### Northern General Hospital, Sheffield, UK

**Correspondence**: Katy Wood

*Journal of Cardiothoracic Surgery 19(1):* A149

**Objectives:** To re-audit the compliance of prescribing and administration of MSSA decolonisation treatment for in house urgent cardiac surgery patients.

**Methods:** A retrospective notes audit was undertaken using a convenience sample of 50 in house urgent cardiac surgery patients.

**Results:** 76% of patients received a full 5-day course of chlorhexidine bodywash. 64% of patients received a full 5-day course of mupirocin nasal ointment.

**Conclusions:** Whilst compliance with decolonisation treatment had improved since the previous audit, compliance remains significantly less than the 100% standard. Multiple factors were identified both from a prescribing and administration aspect which contributed to the non-compliance with the standard. An action plan has been developed to address these factors to improve compliance with the MSSA decolonisation standard.

## A150 Feasibility and acceptability of home-based computerised cognitive training after cardiac surgery (FACCT study)

### Tracey Bowden^1^; Catherine S Hurt^1^; Julie Sanders^2^; Leanne M Aitken^1^

#### ^1^City, University of London, London, UK; ^2^St Bartholomew's Hospital, London, UK

**Correspondence**: Tracey Bowden

*Journal of Cardiothoracic Surgery 19(1):* A150

**Objectives:** Cognitive dysfunction occurs in up to 50% of patients after cardiac surgery. The aim of this study was to evaluate the feasibility and acceptability of a home-based computerised cognitive training programme in postoperative cardiac surgery patients.

**Methods:** This is a single-arm, non-blinded, feasibility and acceptability study on adult (> 18 years of age) patients admitted for first time elective cardiac surgery, registered on clinicaltrials.gov (NCT05298540). Participants were required to complete an 8-week cognitive training programme (40 sessions [20 min/day, 5 days/week]), commencing one week postoperatively, and administered using their own computers or tablets. The Montreal Cognitive Assessment (MoCA), a brief test of global cognition, was administered preoperatively and after the training programme. Feasibility outcomes included recruitment and retention rates and adherence to the programme. Acceptability was assessed by the Theoretical Framework of Acceptability Questionnaire (TFA-Q) which was administered post-programme.

**Results:** In total, 95 patients were screened, 51 (53.7%) were eligible and approached, and 31 (60.8%) consented to participate. Of these, 29 participants enrolled in the cognitive training programme, 7 (24.1%) were female, 20 (69.0%) were white British, and the mean baseline MoCA was 27/30. To date, all participants have completed the pre-operative assessments and 10 have completed the 8-week training programme. All data collection will be completed by December 2022. Feasibility and acceptability will be assessed and available to present.

**Conclusions:** This study exploring the feasibility and acceptability of a home-based computerised cognitive training programme has shown promise thus far. Such a programme could offer an inexpensive and safe method of improving postoperative cognitive function. Following the completion of this work a randomised controlled trial is planned.

## A151 Assessing current rehabilitation services in a complex cardiothoracic ITU environment in conjunction with developing an occupational therapy service

### Alix Shearer; Ewan Sharp

#### Golden Jubilee National Hospital, Glasgow, UK

**Correspondence**: Alix Shearer

*Journal of Cardiothoracic Surgery 19(1):* A151

**Objective**: To assess the current rehabilitation service in ITU using a standardised and validated outcome measure (Chelsea Physical Assessment Tool, CPAx). In conjunction with developing the role of Occupational Therapy (OT) in ITU and supporting earlier rehabilitation.

**Methods**: Over an eight-week period, all patients who remained in ITU on day four were scored using CPAx. Patients with a CPAx score of 25 or less were referred to OT, as well as those readmitted from the ward. CPAx scoring was completed 2–3 times a week to assess respiratory and functional elements. To ensure the CPAx remained standardised, OT assessed patients on a correlating scale for grip strength and cognition.

**Results**: 13 patients were included. Two main trends were established: those who immediately progressed (mean total length of stay (LOS) 12.4) and those with a delay in rehab progression (mean total LOS 18.75). Average CPAx scores for those in ITU for less than 10 days was higher (42) than patients admitted for greater than ten days (18). Eight regained independent functional usage of both upper limbs with OT and six were cognitively impaired, inhibiting their functional ability.

**Conclusions**: Patients with an ITU length of stay of > 10 days resulted in an increased total LOS, prolonged rehab or non-survival. All patients (bar two) with a LOS < 10 were rehabilitated quicker and discharged home. Those in receipt of OT by day 7 of admission went home. CPAx scores are higher when sitting balance is assessed within eight days.

## A152 Discontinuation of routine strict sternal precautions (SSP) post sternotomy in cardiac surgery patients

### Megan Gregory; Tracy Gee; Eleanor Douglas

#### Nottingham University Hospitals NHS Trust, Nottingham, UK

**Correspondence**: Megan Gregory; Tracy Gee

*Journal of Cardiothoracic Surgery 19(1):* A152

**Objectives:** Evaluate the impact of discontinuing routine SSP post sternotomy on length of stay (LOS) and number of physiotherapy (PT) interventions in cardiac surgery patients.

**Background:** SSP have a profound effect on a patient’s recovery. In the short term, independent mobilisation may be hindered and in the long term, reductions in upper limb range of movement can impair functional independence. Routine SSP appear to be based on historic practice as there is a paucity of evidence to support their use.

We discontinued routine SSP. For those deemed to be at low risk of sternal dehiscence the 'keep your move in your tube' concept of movement was implemented. For patients surgeons deemed as high risk there was an opt-in to continue use of SSP.

**Method:** Data on LOS and PT requirement was prospectively collected for two months prior to discontinuing routine use of SSP and for two months following a four-month implementation period of ‘keep your move in your tube’ and opt in for high-risk patients.

**Results:** There was an overall mean reduction in LOS in hospital, on Cardiac Intensive Care Unit (CICU) and the Cardiac High Dependency Unit (CHDU) and the ward. A reduction in PT interventions was also observed, the mean number of PT treatment days and number of sessions both reduced. No other significant changes in practice were implemented over the time period.

Table shows the mean for each measure pre and post change in practice. The final column shows the reduction for each measure post change in practice.

**Table:** 

	With sternal precautions	'Keep your move in your tube'	Difference
Mean LOS Hospital (Days)	10.2	8.5	1.7
Mean LOS CICU/CHDU (Days)	5.7	4.9	0.8
Mean LOS Ward (Days)	4.6	3.7	0.9
Mean Number of PT days (Days)	7	5.3	1.7
Mean Number of PT Treatments	9	7.4	1.6

**Conclusions:** LOS and number of PT interventions reduced when a patient’s function was not restricted by the use of SSP. This may indicate that SSP use delays patients’ independence. Further work is required to assess if the observed changes in LOS and PT intervention can be attributed to discontinuation of SSP, and if there is long-term impact, such as an increased incidence of sternal malunion.

## A153 Cardiac ERAS (enhanced recovery after surgery): the introduction and evaluation of a novel service on a cardio-thoracic ICU to improve patient care

### Myrna Scott; Young Kim; Allan Bravo; Eliseo Sampiano

#### St George's Healthcare NHS Trust, London, UK

**Correspondence**: Myrna Scott

*Journal of Cardiothoracic Surgery 19(1):* A153

## Objectives:

1. Introduce CERAS on a cardiac ICU after the second COVID wave to improve and enhance care of post operative patients through staff engagement, literature reviews, guideline development, audit of CERAS elements, education and patient engagement.
2. Work with the cardiac surgical pre op, peri op and post op MDTs to develop CERAS.
3. Develop audit tools to evaluate each element of CERAS and use the data collected to adapt, change or continue with the current CERAS guidelines.
4. To involve all staff on Cardiac ICU caring for cardiac surgical patients in this innovative and exciting project.

**Methods:** The 11 elements of CERAS are owned by a 15 strong team. To date each element has a complete or in progress literature review and guideline. Seven elements are fully audited using novel audit tools with data from August, n = 25 patients, and future months will be presented at conference.

**Results:** To date: development of an updated goal directed therapy guideline, introduction of Hemosphere monitors and Thopaz + drains, confirmation of the current pain management strategy, extubation targets as per CERAS recommendations with 75% patients extubated in less than six hours and more than 50% in less than four hours. Surgical Wound site surveillance, glycaemic control, delirium and pacing audits are completed and ready for analysis.

**Conclusions:** The CERAS project is exciting and dynamic, involving a dedicated team of staff and engaging the whole MDT. It has contributed to not only improving patient care through literature reviews, guideline development and dedicated audit, but by reinvigorating the team, whose first passion has always been cardiac surgery, but were diverted by the pandemic. The project demonstrates that audit and improvements to care and practice can continue, despite the pressures of a pandemic and current health service demands.

## A154 The impact of COVID and the relocation of the thoracic research team

### Helen Shackleford^1^; Salma Kadiri^2^; Christer Lacson^1^; Aya Osman^1^; Akshay Patel^1^; Hazem Fallouh^1^; Babu Naidu^3^

#### ^1^QE Hospital, Birmingham, UK; ^2^University Hospitals Birmingham, Birmingham, UK; ^3^University of Birmingham, Birmingham, UK

**Correspondence**: Helen Shackleford

*Journal of Cardiothoracic Surgery 19(1):* A154

**Objectives:** Re-establishing the research portfolio and team post Covid at a new hospital site.

**Methods:** During Covid, all our studies were paused and were informed of the relocation to a new site. The impact of the relocation affected our team, we lost five members. We have reflected on the collective experience of the research team and identified the main challenges.

**Results:** Due to no face-to-face contact; waiting lists were decimated. Contact with our patient and public involvement group and our follow up patients was limited, this was a loss of extra support which patients had benefited from. Although one trust, this was a new environment with different ways of working and lack of access to facilities e.g. use of rooms for consenting, meetings and standards of practices. Some studies did not fit into the new process.

**Conclusions:** With the development of a strategy plan, we have stopped over recruitment to studies and focus on maximising follow up with data collection. Individual goals planned, staff recruited, studies were re-opened, and new ways of working were formed such as home visits, virtual meetings, and online surveys. Despite the challenges, we have built rapport with other departments. As a team, we rose to the challenges.

## A155 Audit of GTN infusion use post CABG at royal brompton and harefield hospital

### Nisha Bhudia; Hayley Patel; Shahzad Raja

#### Royal Brompton and Harefield Hospital part of Guys and St Thomas' NHS Foundation Trust, London, UK

**Correspondence**: Nisha Bhudia

*Journal of Cardiothoracic Surgery 19(1):* A155

**Aims and Objectives:** Review the practice of using GTN infusions for 24 h post CABG and encourage uniform practice across two sites in the trust. Review the benefits and evidence for Glycerine Trinitrate (GTN) infusion for 24 h post Coronary Artery Bypass Graft (CABG) and standardise the practice across all sites.

**Methods:** Data for all patients who underwent CABG from 1st Jan 2022 to 28th Feb 2022 across two sites were reviewed. Information on GTN infusion obtained from ICCA and clinical data obtained from PATS/NICOR database. Criteria measured included: Number of patients that underwent CABG (including combined CABG/Valve surgery) at two sites; Number of patients that had GTN infusion prescribed; Dose and duration of GTN infusion.

**Results:** No evidence of benefit to patients for the use of GTN infusions for 24 h post CABG was found. 96% of the patients received GTN infusion at site A whereas only 16% of the patients received GTN infusion at site B immediately postoperatively. 88% of the patients received GTN infusion for less than 24 h with a dose range between 0.5 and 11 mls/h at site A, whereas 71% of patients received GTN infusion for less than 24 h with a dose range between 1-8mls/hour at Site B.

Ways to improve and encourage standardisation of practice include: Not to initiate GTN infusion for 24 h post CABG as a standard historic practice; prescribe GTN infusions only if clinically indicated; Remove prescriptions of GTN infusions from electronic order sets postoperatively; Achieve standardisation across all sites to ensure only evidence-based practices are undertaken to treat patients.

**Conclusions:** There is difference in practice across two sites within the same trust and standardisation of practice will be beneficial. The change in practice showed improvement and should be reaudited. There is a need to develop a local trust guideline. A national audit to compare practices across all cardiac centres may be beneficial.

## A156 Comparison of SPPB score as an indicator of functional outcome in cardiac transplant DCD vs DBD recipients

### Florence Edwards; Emma Matthews

#### Royal Papworth Hospital, Cambridge, UK

**Correspondence**: Florence Edwards

*Journal of Cardiothoracic Surgery 19(1):* A156

**Objectives:** To compare Short Physical Performance Battery (SPPB) scores at discharge from hospital in cardiac transplant recipients who received their organs via DCD (donation after circulatory death) or DBD (donation after brainstem death) donation.

**Methods:** This is a single-centre, retrospective, observational cohort study comparing SPPB outcomes of heart transplant patients who received hearts transplant from DCD versus DBD donors from May 2021 to May 2022.

Between May 2021 and May 2022 there were 25 DBD and 25 DCD heart transplants. Of these recipients, 18 DBD recipients and 17 DCD recipients completed an SPPB at point of discharge from hospital. The primary outcomes reviewed were average scores of individual components of the SPPB and total score. The SPPB score is measured out of 12, 4 points for each section.

**Results:** In DBD recipients Total SPPB score averaged at 9.16 out of 12. In DCD recipients Total SPPB score averaged at 10.29 out of 12.

Average scores of each component of the SPPB are displayed below:
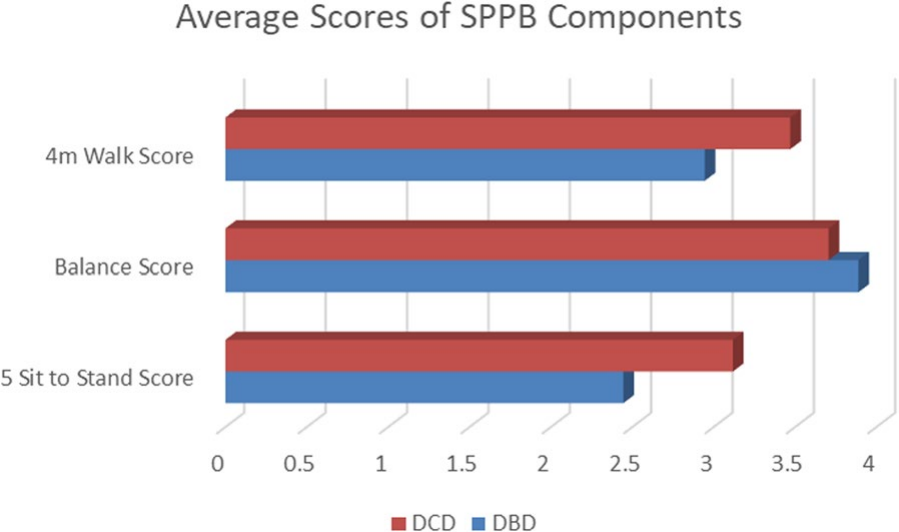


Classification of limitations based on SPPB score suggests a score of 7–9 relates to mild limitations and 10–12 suggests minimal limitations therefore the above scores compared between DCD and DBD recipient scores suggest minimal and mild limitations.

**Conclusions:** Being a recipient of a DCD donated heart transplant does not negatively impact functional outcomes post-cardiac transplant in comparison with DBD donation recipients.

## A157 Patient and public involvement (PPI) in research during and after COVID

### Christer Lacson; Aya Osman; Salma Kadiri; Helen Shackleford; Akshay Patel; Hazem Fallouh; Babu Naidu

#### University of Birmingham / University Hospitals Birmingham (UHB), UK

**Correspondence**: Christer Lacson

*Journal of Cardiothoracic Surgery 19(1):* A157

**Objectives:** To deliver an effective Patient and Public Involvement (PPI) group engagement in a non-NHS location and through virtual contact.

**Methods:** Our methods were to recruit new members throughout the COVID period by telephone contact; engage members and capture feedback through short online surveys and by post (for the members who does not have digital access); and early face-to-face meeting post-COVID outside the health sector.

**Results:** We have recruited 40 new members and eight members and carer broke social isolation and attended the face-to-face meeting. We were able to gain feedback for NIHR HTA study Fit4Surgery 2 which helped the proper design and aided successful funding. However, there was apprehension before the face-to-face meeting but it was preferred.

**Conclusions:** The strategy was successful. The mixture of both face-to-face and virtual is a good model for future PPI activity. Conducting the PPI meeting in a non-NHS location was positive for both the patient and institution.

## A158 Does acupuncture reduce anxiety levels preoperatively in patients awaiting urgent in-house cardiac surgery?

### Cristina Ruiz Segria; Enoch Akowuah; Joel Dunning; Robin Sunley; Rebecca Maier; Rui Fontes

#### James Cook University Hospital, Middlesbrough, UK

**Correspondence**: Cristina Ruiz Segria

*Journal of Cardiothoracic Surgery 19(1):* A158

Preoperative anxiety is common in patients undergoing surgery and it can potentially increase the risk of postoperative complications such as pain and prolong stay. Although a dedicated team reviews this group of patients daily, we have never evaluated or treated their anxiety.

Acupuncture is a non-pharmacological strategy that is widely used to reduce anxiety with a very low cost, however its use is limited in the hospital setting.

**Objectives:**

1. To evaluate if preoperatively anxiety exists and document the extent of the problem in patients awaiting inhouse urgent cardiac surgery.
2. To determine if mentioned anxiety can be reduced if a new service of acupuncture is introduced

**Methods:** Patients referred for inhouse urgent surgery were asked to complete a HADS score (Hospital Anxiety and Depression Score) on arrival, after seven days if still waiting for surgery and one the night before surgery.

Acupuncturist offered treatment after first HADS score was completed. This is based on six needles (four in the body and two in auricular points) left in situ for 20 min either seat in the hospital chair or bed.

**Results:** Early results showed a reduction on anxiety in 50% of the patients with a single treatment of acupuncture.

**Conclusions:** Acupuncture can reduce anxiety prior surgery, but further large-scale and rigorous studies with more treatments are needed to confirm their efficacy.

## A159 Inequality in the NHS should not be ignored

### Xiaohui Liu

#### University Hospital Southampton NHS Foundation Trust, Southampton, UK

**Correspondence**: Xiaohui Liu

*Journal of Cardiothoracic Surgery 19(1):* A159

**Objectives:** I will explore my personal experiences and review literatures of inequality in the workplace, and recommendations of how to overcome these obstacles.

**Methods:** Provide some data from literatures, different trusts and national institutions as well as some case studies regarding inequality, bullying and harassment in the workplace.

**Results:** Recommendations of how to build resilience to face the challenges within the modern NHS in the future and how to seek support when experiencing inequality in the workplace within the NHS. Inequality is not acceptable in NHS regardless of what your colour, background, your physical appearance, your ability and your band is etc.

**Conclusions:** We need to work together to build a better environment for everyone who works in the NHS.

## A160 How you can support staff wellbeing in the workplace

### Xiaohui Liu

#### University Hospital Southampton NHS Foundation Trust, Southampton, UK

**Correspondence**: Xiaohui Liu

*Journal of Cardiothoracic Surgery 19(1):* A160

**Objectives:** A lot of evidence suggests that improving staff wellbeing has better outcomes on patients care, increasing staff productivity and reducing sickness absence.

**Methods:** Provide some data from literatures review, different trusts and national institutions as well as some case studies regarding how to improve staff wellbeing. Explore the evidence of barriers to staff engagement, and how the leaders can facilitate and improve staff wellbeing.

**Results:** Leaders need to facilitate the wellbeing service tailored to each individual team member, and ensure their needs are met. The leaders also need to evaluate the service in order to overcome the berries.

**Conclusions:** Leaders need to adopt a strategic and needs-led approach facilitate their team member’s wellbeing. Leaders also need to balance their own work life balance to enable them to support their team members.

## A161 How to set up a nurse-led genetics clinic for patients with small aneurysms

### Una Ahearn

#### Liverpool Heart and Chest Hospital, Liverpool, UK

**Correspondence**: Una Ahearn

*Journal of Cardiothoracic Surgery 19(1):* A161

**Objectives:** There is increasing perception that Thoracic Aortic aneurysms have a genetic foundation in syndromic and non-syndromic diseases or are acquired sporadically as a chronic degenerative process. Various genes have been associated with Thoracic aortic aneuryms and dissection. Consequently, genetic testing can identify pathogenic mutations in specific genes that increase patients' risk of aortic aneurysm, aortic dissection and may inform on the timing of surgery to prevent an aortic catastrophe. The latest recommendation of the UK NHS National Genomic Test Directory for diagnostic genetic testing of a proband has reduced to a lower threshold of 3.8 cms. Subsequently, this has led to an increase in the number of patients eligible for genetic testing, coupled with the increase in incidental findings of thoracic aortic aneurysms from local lung cancer surveillance programmes, prompted interest in developing a nurse-led genetics clinic model for patients referred with small aneurysms.

**Methods:** A robust programme of nurse education and assessment was initiated. Patients referred with aneurysms measuring < 5.5 cms who fulfilled the testing criteria were counselled and offered genetic testing.

**Results:** 31% of patients reviewed fulfilled the criteria and were offered genetic testing. 29% accepted whilst 2% declined. All patients with a pathogenic variant were referred to a genetics Counsellor to initiate predictive genetic testing. All patients with variants of unknown significance were referred to a clinical geneticist. Our nurse-led Genetics clinic, with a face-to-face model opened six months ago and has received positive feedback.

**Conclusions:** Despite some initial challenges, the Nurse-led genetics clinic is popular with patients and a pragmatic solution to the increasing number of patients with aortic disease suitable to be offered genetic testing.

## A162 Service evaluation of a pilot prehabilitation programme in a North East tertiary thoracic surgical centre: freeman hospital

### Elisabeth Green^1^; Jackie Spensley^2^; K Ang^2^; Dharmendra Agrawal^2^

#### ^1^Newcastle Hospice, Newcastle, UK; ^2^Freeman Hospital, Newcastle, UK

**Correspondence**: K Ang

*Journal of Cardiothoracic Surgery 19(1):* A162

**Objectives:** We conducted a service evaluation of a pilot programme offering prehabilitation to patients with diagnosed or suspected lung cancer undergoing surgery at Freeman Hospital.

**Methods:** A pilot prehabilitation programme was offered to eligible patients during a 10-week period, whereby they were assessed for their physical, psychological and nutritional status before their surgery. Participating patients were then given an individualised prehabilitation programme over a minimal of 2 week period to improve their functional status. The programme included one or more of the following: exercise regimen, dietary input, psychosocial support, as well as alcohol and smoking cessation support if required. We evaluated service delivery and its impact on the patients.

**Results:** Forty patients were approached. Ten patients agreed to participate. Main reasons for patients declining prehabilitation were: logistical issues; extra cost incurred to attend additional prehabilitation sessions. Seven patients completed the minimal prescribed prehabilitation duration, two of which needed extra dietary support. Seven patients responded to the post-prehabilitation survey. Majority felt that prehabilitation was very useful in improving their exercise and performance status, and was helpful in their preparation for surgery.

**Conclusions:** Prehabilitation is feasible and beneficial for patients undergoing oncological lung resections. However, there are challenges for its delivery to the wider population.

## A163 Developing a physiotherapy enhanced recovery programme following pulmonary endarterectomy surgery

### Bryony Lewis; Nikki Gilbert; Ciara Smalley

#### Royal Papworth Hospital NHS Foundation Trust, Cambridge, UK

**Correspondence**: Bryony Lewis; Nikki Gilbert

*Journal of Cardiothoracic Surgery 19(1):* A163

**Objective:** Pulmonary endarterectomy (PEA) surgery is an intervention for patients with chronic thromboembolic pulmonary hypertension. Due to a combination of symptoms and a lack of education and support, many patients avoid exercise following surgery. Physiotherapy (PT) management in the UK has focused on functional mobility, and advice includes avoiding strenuous exercise until six-month review. Research internationally has suggested that exercise is safe and beneficial in the early period following PEA. Since January 2022, the PT team at a UK cardiothoracic centre developed an enhanced recovery programme to support appropriate patients to exercise following PEA.

**Method:** Following discussions with the multidisciplinary team, criteria were established to identify appropriate patients with good operative outcomes to engage in early post-op exercise. The programme includes guidelines for exercise for inpatients, and a pathway for appropriate patients to be assessed and enrolled for pulmonary rehabilitation (PR) following discharge. This pathway was developed taking into consideration the geographical spread of patients, with potential face to face or virtual attendance of PR.

**Results:** Since March 2022, six patients have been identified as appropriate and consented to attend PR following PEA surgery. All patients have completed, or are in the process of undertaking, a six-week course of twice weekly exercise and education sessions. Outcome measures for patients who have completed the programme have shown improvements including the incremental shuttle walk test and their CAMPHOR score. Patient feedback has been positive and there have been no adverse events.

**Conclusions:** Preliminary data has shown that supervised exercise in the early months following PEA surgery can be safe and effective for improving exercise capacity and health related quality of life. Priorities for future include expanding the inclusion criteria and number of patients who can safely engage in exercise post-PEA.

**Pat Magee Competition**

## A164 The role of CEPDs during TAVI in preventing stroke and mortality as well as their impact on acute kidney injury: a systematic review

### Amelia Websdale

#### University of Manchester, Manchester, UK

**Correspondence**: Amelia Websdale

*Journal of Cardiothoracic Surgery 19(1):* A164

**Introduction:** Severe aortic stenosis [AS] occurs when the aortic valve area becomes less than 0.8 cm^2^ (Smith et al., 2011). Routine resolution of AS is done by surgical aortic valve replacement [SAVR], however in high-risk surgical patients, transcatheter aortic valve implantation [TAV]) is used. Cerebral embolic protection devices [CEPDs] can be utilised during TAVI to prevent embolic material from traveling from the valve site to the brain, and therefore reducing stroke—the most common complication from TAVI.

**Objectives:**

1. Do CEPDs reduce the risk of stroke and mortality at in hospital and 30-day intervals?
2. Do CEPDs increase the incidence of acute kidney injury due to increased fluoroscopy time?

**Method:** Using a systematic search strategy, a total of 379,454 participants undergoing TAVI with or without CEPD usage were studied. Rates of stroke and mortality at in-hospital and 30-day post-TAVI intervals, as well as mean fluoroscopy time, and acute kidney injury incidence, were recorded and meta-analysed.

**Results:** CEPDs showed to be successful in decreasing stroke and mortality at both intervals, with p-values all < 0.05. Mean fluoroscopy time was not found to be significantly different between CEPD versus no CEPD groups and decreased acute kidney injury rates favoured use of CEPDs.

**Conclusions:** This systematic review provided evidence that CEPDs do significantly decrease stroke and mortality rates at both in-hospital and 30-day intervals. Average fluoroscopy time showed no significant difference between CEPD and no-CEPD cohorts, and AKI incidence was significantly lower in CEPD groups.

## A165 Extra-pleural pneumonectomy with diaphragmatic and pericardial reconstruction in the treatment of Doege-Potter syndrome

### Bhanu Wahi-Singh^1^; David Dorward^2^; Rocco Bilancia^3^; Malcolm Will^4^

#### ^1^Department of Cardiothoracic Surgery, The Royal Infirmary of Edinburgh, Edinburgh, Scotland; ^2^Department of Pathology, The Royal Infirmary of Edinburgh, Edinburgh, UK; ^3^Golden Jubilee National Hospital, Glasgow, UK; ^4^Royal Infirmary of Edinburgh, Edinburgh, UK

**Correspondence**: Bhanu Wahi-Singh

*Journal of Cardiothoracic Surgery 19(1):* A165

Doege-Potter Syndrome (DPS) is a rare paraneoplastic syndrome presenting as non-islet cell tumour hypoglycaemia, primarily in patients with Solitary Fibrous Tumours (SFTs). While non-surgical interventions may prove useful in hypoglycaemia management, the only definitive treatment is surgical resection. However, no DPS-causing SFT with involvement of the chest wall, pleura, diaphragm, and pericardium has been successfully resected. It remains a question whether DPS-causing diffuse, malignant SFTs can be successfully treated through Extra-Pleural Pneumonectomy (EPP).

A 74 y.o. female presented with nocturnal hypoglycaemia and blood glucose < 3 mmol/L, refractory to medical treatment. Past medical history included a 2019 SFT mass resection by R thoracotomy. CT Chest demonstrated large R lower zone mass and multiple pleural deposits. The patient thus underwent an EPP with complete diaphragmatic and pericardial reconstruction.

Postoperatively, the hypoglycaemia resolved. The patient was treated for Atrial Fibrillation and the mediastinum repositioned on one occasion. No unexpected inpatient post-EPP complications occurred. The patient was discharged 13 days post-operatively.

Pathology findings confirmed recurrent SFT with features similar to the 2019 tumour resection with no evidence of a de-differentiated or sarcomatoid component. There was a miliary pattern of spread with multiple deposits across the pleura and lung as well as involvement of the chest wall margin.
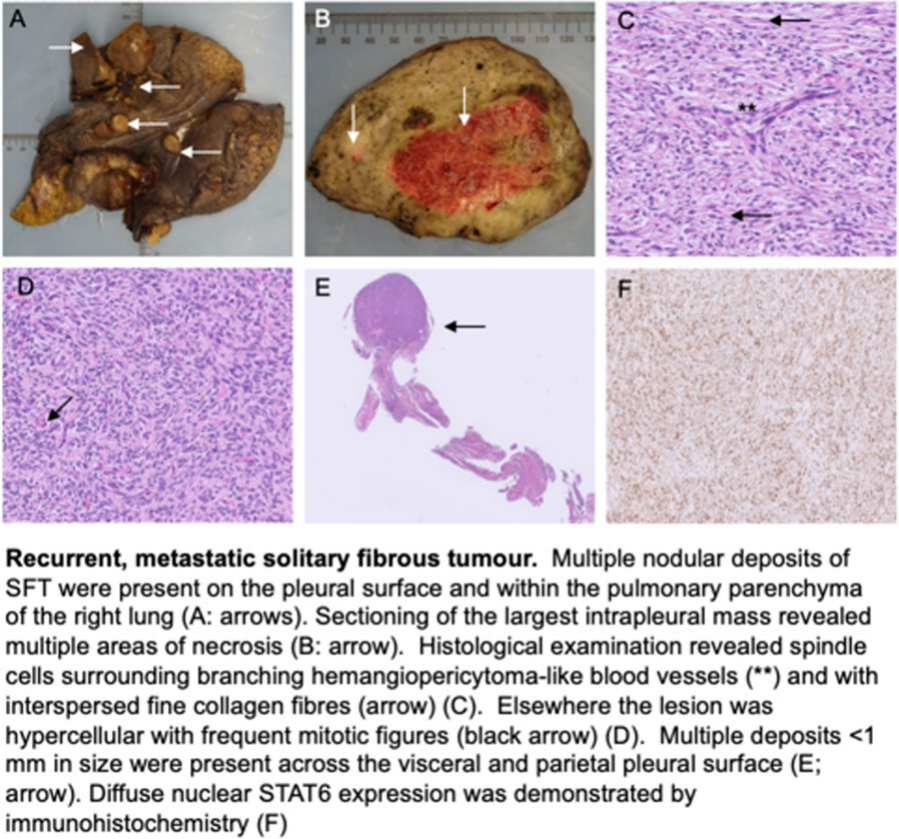


Due to high recurrence risk, the case was discussed at a lung cancer and sarcoma multidisciplinary team meeting where the patient was referred for initial oncological follow-up and repeat imaging of a previously identified T2 sclerotic lesion.

This case highlights the extremely rare use of EPP in the multimodality treatment of recurrent, disseminated SFT with refractory hypoglycaemia. In review of the medical literature, we identified only one similar case, published in 2004, in a patient with SFT but not DPS.

The patient gave their consent to publish their information in an Open access journal.

## A166 Outcomes of tricuspid annuloplasty in degenerative mitral valve surgery: a meta-analysis

### Samuel Burton^1^; Alexander Reynolds^2^; Amit Modi^3^; Sanjay Asopa^4^

#### ^1^Peninsula School of Medicine, Plymouth, UK; ^2^Swansea University Medical School; ^3^Southampton General Hospital; ^4^Derriford Hospital, Plymouth, UK

**Correspondence**: Samuel Burton

*Journal of Cardiothoracic Surgery 19(1):* A166

**Objectives:** This meta-analysis aimed to compare the clinical outcomes of patients receiving tricuspid valve annuloplasty (TVA) at the time of mitral valve repair or replacement for patients with degenerative mitral valvular disease.

**Methods:** Literature search was conducted with the terms (“mitral valve surgery” AND (“tricuspid valve intervention” OR “tricuspid surgery”)) in PubMed, Cochrane Library, and Google Scholar databases. Following PRISMA guidelines, suitable studies were identified and data was pooled for outcomes reported in three or more studies. Forest plotting was performed using Review Manager 5.3. Primary outcomes included long-term freedom from moderate or severe tricuspid regurgitation (TR), new pacemaker implantation, and rate of reoperation.

**Results:** PRISMA selection identified three retrospective studies and one randomised control trial. Subsequent data pooling of 2,217 patients demonstrated a significant difference in the rate of new pacemaker implantation, favouring mitral valve surgery (MVS) without TVA (odds ratio [OR], 4.46; 95% confidence interval [CI], 2.40 to 8.26; I2 = 19%; p =  < 0.00001). Meta-analysis of 1391 participants revealed no significant difference in long-term freedom from moderate or greater TR (OR, 0.34; CI 0.09 to 1.19; I2 = 86%; p = 0.09) with a mean follow-up period of 4.6 years. All studies that stated rates of reoperation reported no further surgery for recurrent TR.

**Conclusions:** Meta-analysis of clinical outcomes demonstrated that MVS with TVA has higher rates of new pacemaker implantation and no difference in freedom from long-term moderate or greater TR. Studies reported no reoperations for TR.

## A167 Perceval sutureless bioprosthesis versus perimount sutured bioprosthesis for aortic valve replacement in patients with aortic stenosis

### Sharan Kapadia^1^; Yousuf Salmasi^2^; Heba Mohamed^2^; Alicja Zientara^2^; Cesare Quarto^2^; George Asimakopoulos^2^

#### ^1^Imperial College School of Medicine, London, UK; ^2^Royal Brompton and Harefield NHS Foundation Trust, London, UK

**Correspondence**: Sharan Kapadia

*Journal of Cardiothoracic Surgery 19(1):* A167

**Objectives:** Rapid-deployment aortic valve replacement (RDAVR) is an emerging alternative to conventional AVR (cAVR) for aortic stenosis. Studies suggest that the rapid-deployment Perceval valve reduces operative times, may improve recovery, and has similar or superior haemodynamics compared to conventional valves. However, further evidence, particularly mid-long-term data, is required to inform guidelines.

**Methods:** This was a single-centre, retrospective cohort study comparing baseline, intra-operative, post-operative, and mid-long-term parameters between patients receiving the Perceval valve (*n* = 133) versus the conventional Perimount valve (*n* = 258) between 2014 and 2020. Data were extracted from a prospectively collected, nationally managed dataset.

**Results:** The Perceval patients were older (Perceval 74 [69–78] years; Perimount 71 [66–76] years, *P* < 0.001). The Perceval group had reduced cross-clamp (Perceval 62 [49–81] minutes; Perimount 79 [63–102] minutes, *P* < 0.001) and bypass times (Perceval 89 [74–114] minutes; Perimount 104 [84–137] minutes, *P* < 0.001). The Perceval group had less frequent post-operative acute kidney injury (AKI) (Perceval 0%; Perimount 4.1%, *P* = 0.017), but more frequent thrombocytopaenia (Perceval 5.3%; Perimount 1.6%, *P* = 0.015), and pacemaker implantation (Perceval 14%; Perimount 7.4%, *P* = 0.048). Haemodynamic performance was initially inferior in the Perceval group but equalised at 12 + months. The Perceval was associated with lower LVEF (relative to baseline) at 2 + years (*B*: − 12.2, 95% CI: [− 22, − 2.0], *P* = 0.02).

**Conclusions:** The Perceval facilitated shorter operations; this may carry post-operative benefits such as reduced AKI. Hence, the Perceval could benefit intermediate-high-risk, elderly patients with comorbidities (such as renal disease) requiring concomitant procedures, serving as a ‘bridge’ between cAVR and TAVR. However, pacemaker implantation, thrombocytopaenia, and potentially reduced mid-long-term LVEF (relative to baseline) may be drawbacks.

**Table:** 

	Perceval (*n* = 133)	Perimount (*n* = 258)	*P*-value
Cross-clamp time / minutes	62 (49–81)	79 (63–102)	< 0.001*
Bypass time / minutes	89 (74–114)	104 (84–137)	< 0.001*
Minimally invasive	32% (43)	5% (12)	< 0.001*
Thrombocytopaenia	5.3% (8)	1.6% (4)	0.015*
Acute kidney injury	0% (0)	4.1% (10)	0.017*
Pacemaker implantation	8.3% (11)	5.5% (13)	0.288*
Operative mortality	1.5% (2)	1.9% (5)	0.759
Mid-long-term mortality	2.3% (3)	5.8% (15)	0.112

## A168 Future landscape of cardiothoracic surgery: attitudes amongst attendees of the SCTS 8th annual student engagement day

### Javeria Tariq^1^; Farah Bhatti^2^; Karen Booth^3^

#### ^1^Leeds School of Medicine, Leeds, UK; ^2^Morriston Hospital, Swansea, UK; ^3^Freeman Hospital, Newcastle, UK

On behalf of the SCTS INSINC (medical student) Committee

**Correspondence**: Javeria Tariq

*Journal of Cardiothoracic Surgery 19(1):* A168

Understanding barriers in getting into the specialty of cardiothoracic surgery is an important goal to improve equality and diversity within leadership positions. We aim to demonstrate current perspectives of medical students who engage with the specialty at the 8th annual SCTS student engagement conference.

Approval for the study was sought through the SCTS INSINC committee and Leeds University. All students who registered to attend the SCTS student engagement conference in November 2021 were surveyed through electronic questionnaires. School students who participated were not surveyed. Of the 93 registered delegates, 86 were eligible to be surveyed and 60 completed pre-conference feedback. Data was exported from google forms using Microsoft excel.

Respondents were mostly medical students (57) 95%, the remaining students belonged to allied health programmes. From this cohort (35) 58% were in their first three years of study, (24) 40% were in their fourth and fifth year of study and (1) 2% were intercalating. 49% of students we interviewed stated they were likely to pursue a career in CTS compared to 42% who reported they were undecided and 7% who said they were unlikely. Most attendees were more interested in pursuing a career in cardiac surgery (92%) than thoracic surgery (8%.) Of those interested in cardiac surgery 60% were interested in acquired and 40% in congenital practice. Competition and a lack of training posts was the most reported barrier to the specialty 42% (25). This was followed by length of training and work life balance 15% (9). And 8% (5) reported being a woman as a barrier to getting into the specialty.

Barriers to the specialty are perceived to be gender, length of training, lack of training posts and the resultant competition. This provides valuable insight into the current attitudes of a highly motivated group of students who attended our student engagement day.

Barriers to the specialty as perceived by attendees of the SCTS 8th Annual Student Engagement Day.
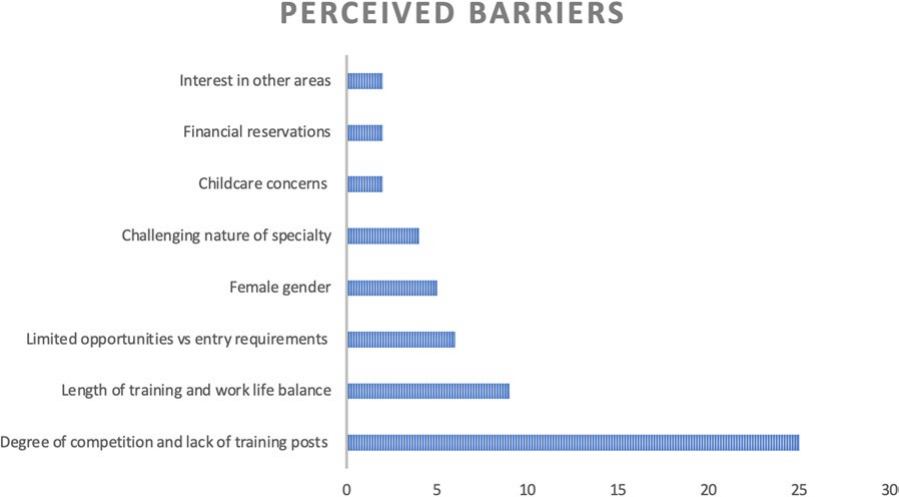


## A169 How should we manage stable coronary heart disease: a comparison of European, American, Japanese and Canadian guidelines on coronary revascularisation?

### Shubhi Gupta^1^; Swarna Yemparala^1^; Mahima S Bharadwaj^1^; Bilal Kirmani^2^

#### ^1^School of Medicine, University of Liverpool, Liverpool, UK; ^2^Department of Cardiothoracic Surgery, Liverpool Heart and Chest Hospital, Liverpool, UK

**Correspondence**: Shubhi Gupta

*Journal of Cardiothoracic Surgery 19(1):* A169

**Objectives:** Clinical guidelines form the framework for clinical decision-making; however, the quality of these guidelines has been subject to criticism. Furthermore, differences in interpretation between contemporaneous guidelines written from the same evidence base have been highlighted recently. This review aims to compare and critically appraise the evidence-base and methodological quality of four international guidelines for stable coronary artery disease (CAD).

**Methods:** A detailed search of PubMed, Scopus and Web of Science was performed from 1st January 2012 to 31st January 2022 by two independent review authors. English language guidelines concerning stable CAD were included. Information concerning author panel, class of recommendation, level of evidence and references was extracted. The Appraisal of Guidelines for Research & Evaluation Instrument (AGREE II) tool was used to assess the guidelines’ methodological quality.

**Results:** The European Society of Cardiology (ESC/EACTS 2019), American Heart Association (ACC/AHA/SCAI 2021), Canadian (2014) and Japanese Circulation Society (JCS 2022) guidelines were included for analysis. The author panels comprised of a higher ratio of cardiologists to cardiothoracic surgeons (ESC/EACTS 12:7, ACC/AHA/SCAI 12:7, Canadian 17:2, JCS 22:4). The aggregate AGREE II score for JCS was 65.3%, ESC/EACTS 68.6%, ACC/AHA/SCAI 68.6% and Canadian 67%. The number of references generating the guidelines’ evidence-bases differed (JCS 1401, ESC/EACTS 529, ACC/AHA/SCAI 1167 and Canadian 143). There was a variation in level of recommendation provided for PCI (IA-IIIA) vs CABG (IA-IIA) in multiple-vessel and left main stem CAD. In addition, the RCT to other study type ratio was also disparate (JCS 1:8, ESC/EACTS 4:9, ACC/AHA/SCAI 4:1 and Canadian 3:4).

**Conclusions:** Quality is consistent and moderately good across all guidelines. However, substantial variation in the interpretation and selection of best evidence amongst guidelines.

## A170 Analysis of surgical sites causing bleeding requiring re-exploration following cardiac surgery

### Natalia Hara; Andre Navarro; Monica Mittal; Alejandra Ceballos; Carlos Corredor; Alex Shipolini; Martin T Yates

#### St. Bartholomew's Hospital, London, UK

**Correspondence**: Natalia Hara

*Journal of Cardiothoracic Surgery 19(1):* A170

Cardiac surgery involves multiple suture lines, the use of systemic heparinisation and often cardiopulmonary bypass with an associated risk of post operative bleeding. Re-exploration is associated with prolonged intensive care unit stay, high rates of blood transfusion, renal impairment, and mortality. This contemporary series aims to identify the surgical sites causing postoperative bleeding.

Patients who underwent adult cardiac surgery followed by re-exploration for bleeding within the first 48 h of the initial operation at our institution from June 2020 to September 2022 were identified. The bleeding site of patients requiring resternotomy was recorded retrospectively from their electronic patient records. Patients with primary diagnosis of tamponade or cardiac arrest were excluded.

109 patients underwent re-exploration for bleeding within the first 48 h following cardiac surgery. No source of bleeding was identified in 36 (33%) of patients, therefore likely related to coagulopathy. A specific bleeding site was found in the 73 (67%) patients. Source of bleeding was 26 (24%) anastomosis/suture lines, 26 (24%) chest wall / soft tissue, 14 (13%) conduits, 5 (5%) cannulation site and 2 (2%) other.

Patients that undergo re-exploration for bleeding have higher rates of mortality and morbidity. Meticulous surgical technique, postoperative monitoring and implementation of systems such as the use of checklists intraoperatively may reduce the incidence of post operative bleeding. This study highlights the most common sites which must be reviewed prior to sternal closure.

## A171 Adequacy of surveillance imaging following lung cancer resection surgery: Are we doing enough?

### Aishah Mughal^1^; Ahmed El-Zeki^2^; Ahmed Habib^2^; Patrick Yiu^2^; Ahmed Oliemy^2^

#### ^1^University of Birmingham, Birmingham, UK; ^2^New Cross Hospital, Royal Wolverhampton NHS Foundation Trust, Wolverhampton, UK

**Correspondence**: Aishah Mughal

*Journal of Cardiothoracic Surgery 19(1):* A171

**Objectives:** Surgical resection with curative intent is offered to patients with localised lung cancer. Post-operative surveillance imaging is essential to detect cancer recurrence. Clinical guidelines by the American Society of Clinical Oncologists (ASCO) recommend all patients with curatively treated lung cancer should undergo surveillance imaging every six months for two years, preferably using computed tomography (CT) imaging with contrast. We aim to evaluate the adequacy of surveillance imaging amongst lung cancer patients following resection surgery, against ASCO guidelines.

**Method:** A clinical audit was performed on all patients undergoing curative lung resection surgery between 19/01/2019 and 22/12/2020. Data was collected from electronic health records including demographics, post-operative tumour staging and presence of surveillance imaging at 6-, 12-, 18- and 24-months post-resection. Data analysis was performed using SPSS.V29.

**Results:** 164 patients undergoing lung cancer resection with complete and available follow-up data were included in analysis. The median age of patients at operation was 74 years (IQR 68–80). Most patients had tumour staging of T1 (48.8%), N0 (68.3%), M0 (83.5%). Surveillance imaging was performed in 73.2%, 62.2%, 51.2% and 45.1% of patients at 6, 12, 18 and 24 months, respectively (Fig. 1). Chest X-ray was the most frequently utilised imaging modality, followed by CT with contrast and CT without contrast. Primary lung cancer recurrence was most frequently detected at 18-months post-resection (9.1%).

**Fig. 1:**
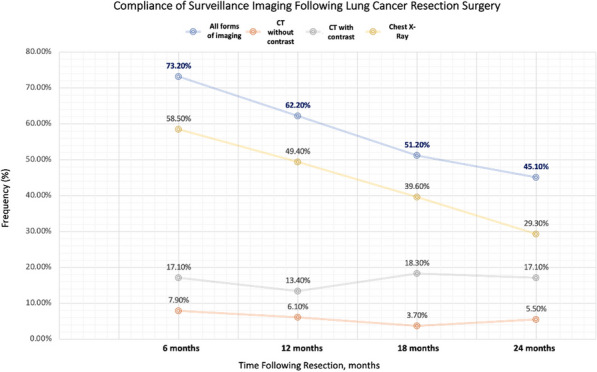
A clinical audit to evaluate the adequacy of surveillance imaging amongst lung cancer patients following resection surgery, against American Society of Clinical Oncologists (ASCO) guidelines

**Conclusions:** Surveillance imaging following resection is adequate within the first 6-months, however, improvement is needed to ensure patients receive timely imaging 24-months post-surgery. Future recommendations should involve implementing digital proforma to assist in prompt surveillance imaging for all patients. Emphasising the importance of using CT imaging (with contrast) amongst clinicians will also enable early detection of cancer recurrence.

## A172 Who deserves a second chance? Considerations and strategies when approaching high-risk lung retransplantations

### Koray N. Potel^1^; Rishav Aggarwal^2^; Rosemary F. Kelly^2^; Ilitch Diaz-Gutierrez^2^; Stephen J. Huddleston^2^

#### ^1^Queen's University Belfast, Belfast, UK; ^2^University of Minnesota, Minneapolis, USA

**Correspondence**: Koray N. Potel

*Journal of Cardiothoracic Surgery 19(1):* A172

**Background:** Mediastinal shift with compression of the transplanted lung is a known complication of single lung transplantation for chronic obstructive pulmonary disease (COPD). Long-term survival with redo bilateral lung transplant is possible in patients with mediastinal shift, although technical obstacles leading to higher early mortality must be overcome.

**Case Presentation:** A 49-year-old female with COPD underwent single right lung transplantation in 2012. Over time, she developed chronic lung allograft dysfunction and left lung hyperinflation which led to a profound mediastinal shift and severe hypoxia. After multidisciplinary evaluation, redo bilateral transplantation was recommended. Preoperative planning relied on CT scans which characterised the chest architecture with mediastinal displacement. Suitable lungs became available, and the patient underwent clamshell thoracotomy for bilateral lung transplantation using femoral arterial and venous cardiopulmonary bypass. Through bilateral anterolateral thoracotomies with transverse sternotomy, extensive adhesiolysis was performed, and the mediastinum was fully mobilised and repositioned in the middle of the thoracic cavity balancing the chest once more. Postoperatively, the mediastinum remained centered, and the patient recovered well.

**Conclusions:** This case highlights how mitigation of perioperative risks through thorough preoperative assessment, strategic surgical planning and experienced teamwork leads to successful outcomes even in a high-risk lung retransplantation population and offers patients with chronic lung allograft dysfunction a viable treatment option and a second chance for full recovery.

I confirm the patient gave explicit permission to publish their information and images in an open access journal.
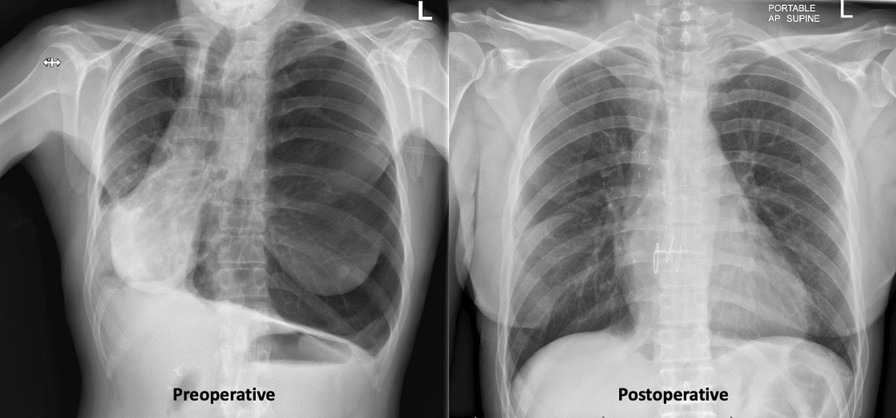


## A173 Altmetric vs bibliometric trends in cardiac surgery research: prospects for social media dissemination of key publications and role in training

### Alana Atkinson^1^; Rickesh Karsan^2^; Gwyn Beattie^2^

#### ^1^Queen's University Belfast, Belfast, UK; ^2^Department of Cardiothoracic Surgery, Royal Victoria Hospital, Belfast, UK

**Correspondence**: Alana Atkinson

*Journal of Cardiothoracic Surgery 19(1):* A173

**Objectives:** Research impact is largely determined by citations publications receive. Web-based platforms look to have changed how scientific research gains attention. We aimed to establish a relationship between web-based platforms and citations, and impact and force of research in cardiac surgery, whilst predicting the future role of social media in highlighting key research.

**Methods:** Cardiac and Surg* were searched in the Clarivate Analytics Web of Science database. The top 100 cited English language articles were identified and cross-referenced to their altmetric score (AS) and stratified by evidence level (based on Oxford Centre for Evidence-Based Medicine: Levels of Evidence, 2009). Altmetric data was broken down by relevant web platforms. Citation and altmetric data were analysed via regression and ANOVA. Forecast models were generated to predict trends in altmetric and citation data over the next 5 years (via IBM SPSS v23.0).

**Results:** Eligible articles totalled 307,286. The most cited article gained a total of 5,061 citation (leon et al.; 2010), with the highest altmetric score was 428 (Ford et al.; 2007). Breakdown of almetric scores showed, patents scored highest (mean 17.48 ± 4.5), with Twitter having the 2nd highest score (mean 10.74 ± 3). AS was found to be associated with citation score (r = 0.473; p =  < 0.001), whilst altmetric rate and citation rate showed a strong relationship (r = 0.549; p =  < 0.001). There was no relationship between evidence level and citations or AS. Forecast models over the next 5-years predicts altmetrics to rise significantly compared to citations p = 0.008.

**Conclusions:** Altmetric data appears to predict the potential future impact of research within cardiac surgery, with a projected rise in web-based platform use to disseminate research. The role of social media platforms is likely to see a greater role in training to highlight key research and guide future development in the speciality.

A graph to show the relationship between citation and altmetric scores by regression.
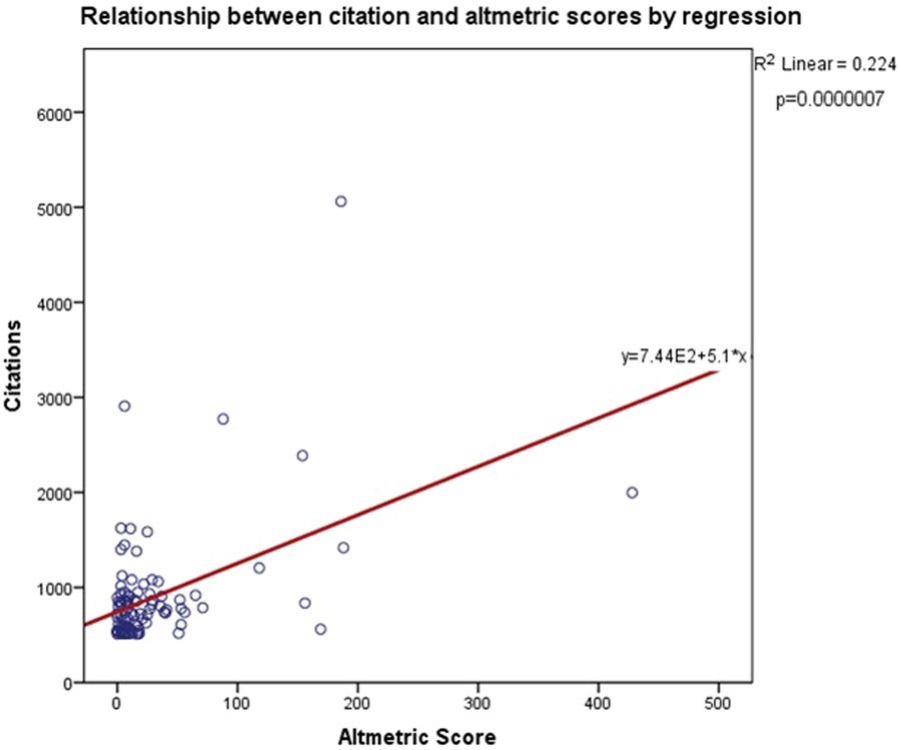


## A174 The impact of COVID-19 on UK medical students' perceptions of cardiothoracic surgery: a comparison of nationwide surveys in 2018 and 2022

### Sathyan Gnanalingham^1^; Raman Gnanalingham^2^; Devan D Limbachia^3^

#### ^1^University College London Medical School, London, UK; ^2^Oxford Medical School, Oxford, UK; ^3^University Hospital Birmingham, Birmingham, UK

**Correspondence**: Sathyan Gnanalingham

*Journal of Cardiothoracic Surgery 19(1):* A174

**Objectives:** COVID-19 had a widespread impact on medical student education in clinical specialties. We investigated how this pandemic has impacted UK medical students’ interest in a career in cardiothoracic surgery (CTS) and their perceptions towards the field.

**Methods:** We distributed the same online survey to all UK medical schools via social media outlets before COVID-19 in 2018 and after in 2022. The survey contained 14 different factors of interest and 7 outreach opportunities to evaluate the importance of different factors when considering a career in cardiothoracic surgery. Chi-Square test was used to analyse differences between 2018 and 2022.

**Results:** Overall, career intentions of survey respondents towards CTS did not change between 2018 and 2022. However, students’ perceptions towards CTS became more negative (p = 0.03). For example, in 2018, 37% of survey respondents had neutral or negative perceptions of CTS which increased to 59% in 2022.

**Conclusions:** Overall, the effects of COVID-19 did not have a significant impact on medical student interest in a career in CTS. However, negative perceptions associated with a career in CTS remain. A new era of remote, hybrid career events and medical student modules in CTS should continue to be explored to encourage greater interest.

No patients were used in this study. I can confirm that Participants consented to their data being used for research publication.

## A175 Interrupted aortic arch type A: an adolescent's late presentation

### Shubhi Gupta^1^; Amr Ashry^2^; Attilio A. Lotto^2^

#### ^1^University of Liverpool, Liverpool, UK; ^2^Department of Paediatric Cardiac Surgery, Alder Hey Children Hospital, Liverpool, United Kingdom, Liverpool, UK

**Correspondence**: Shubhi Gupta

*Journal of Cardiothoracic Surgery 19(1):* A175

**Background:** Interruption of the aortic arch (IAA) is a rare congenital malformation defined by an interruption of the lumen and anatomical continuity between ascending and descending aorta. IAA can be classified into three types, depending on the site of disruption; type A defined as the interruption distal to the left subclavian artery (LSCA) origin. Usually diagnosed within a few hours or days of birth, anatomical surgical repair is performed with Deep Hypothermic Circulatory Arrest (DHCA). Cases reported through adolescence into adulthood are rare and surgical management more complex as anatomical repair not feasible.

**Clinical picture and presentation:** A 16-year-old girl, previously fit and well, presented with a 3-month history of headaches and systemic hypertension.

**Investigations:** Echocardiography showed the presence of type A IAA with descending aorta measuring 11–12 mm. A chest computerized tomography scan further delineated the anatomy of the arch and extensive collateralisation, with enlarged intercostal arteries. The case was discussed at our regional multidisciplinary meeting and an indication for surgical correction was agreed.

**Operative and Postoperative Course:** The patient underwent an elective surgical repair via median sternotomy by a size 14 mm extra anatomic bypass Dacron graft from ascending to descending aorta, with deep hypothermia (24 degrees C) and two arterial cannulae, one in the aorta and an additional femoral arterial cannula to support distal circulation to protect the spine. Patient was extubated the next day, stepped down from ICU 2 days later and discharged eight days postoperatively with lifelong aspirin.

**Conclusions:** Although selection of the appropriate surgical technique is challenging and standard surgery has potential for spine hypoperfusion with consequent paraplegia, the approach used with ascending-to-descending aorta extra anatomical bypass with hypothermia provides a safe alternative in cases of Interrupted Aortic Arch with late presentation.

## A176 Does increased exposure to cardiothoracic surgery increase medical students' interest in the specialty? A systematic review

### Najeeba Lallmahomed^1^; Sarah Alwan^2^

#### ^1^St. Thomas' Hospital, London, UK; ^2^Swansea University, Swansea, UK

**Correspondence**: Najeeba Lallmahomed

*Journal of Cardiothoracic Surgery 19(1):* A176

**Objectives:** The aim of this study is to evaluate whether increased exposure to cardiothoracic surgery increases medical students’ interest in the specialty. We also describe other factors contributing to increased interest as identified in the literature.

**Methods:** A comprehensive search was conducted on the PubMed database. The keywords included "cardiothoracic surgery medical students". Articles up to July 26, 2022, were included in this study. The studies were analysed for their risk of bias and the results were tabulated.

**Results:** Five articles were included in the study, with a total number of 2,736 respondents identified. The percentage of exposed students who were interested in the specialty ranged from 29.6 to 40.5%, versus 23.4% to 59.5% in non-exposed students.

**Conclusions:**

**Table:** 

Study (First author, year)	Number of students	Results	Statistical significance	Risk of bias
Dost 2022 [6]	1675	40.5% of exposed students interested 59.5% of non-exposed students interested	No p values computed	Possible self-selection bias
Miller 2021 [7]	303 (132 women)	58% of women exposed 18% of women interested	No direct comparison in interest between exposed and non-exposed students	(Women-focused study)
Coyan 2020 [8]	126	60% of exposed students 13% of students interested	No direct comparison in interest between exposed and non-exposed students	Low response rate (22%) may have caused selection bias
Gasparini 2019 [9]	162	29.6% of exposed students interested 23.4% of non-exposed students interested	p = 0.4255 No significant difference	Selection bias
Foote 2017 [10]	470 (372 women)	Low interest in female students (30%) Low exposure in female students	No direct comparison in interest between exposed and non-exposed students	Selection bias (Women-focused study, men as a control group)


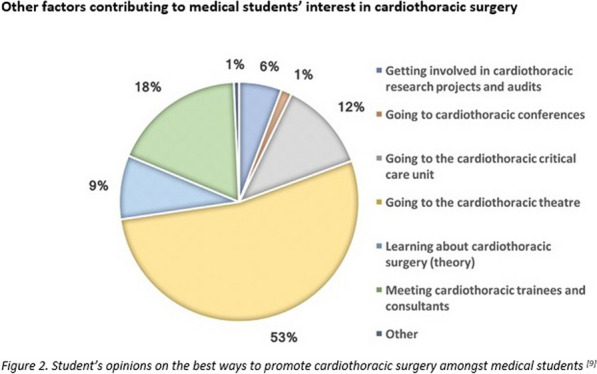


More prospective studies need to be carried out to evaluate the direct effect of exposure to cardiothoracic surgery on medical students’ career choices. Other factors contributing to increased interest included mentorship programmes, electives, highly interactive simulations and portfolio-building workshops.

## A177 Trends in QRS characteristics during follow-up of children and adults with tetralogy of fallot

### Chloe Knight^1^; John Stickley^2^; Paul Clift^3^; Vinay Bhole^2^; Nigel Drury^2^

#### ^1^Univeristy of Birmingham, Birmingham, UK; ^2^Birmingham Children's Hospital, Birmingham, UK; ^3^Queen Elizabeth Hospital Birmingham, Birmingham, UK

**Correspondence**: Chloe Knight

*Journal of Cardiothoracic Surgery 19(1):* A177

**Objectives:** Increasing QRS duration (QRSd) over time is an independent risk factor for sudden cardiac death in patients with tetralogy of Fallot (TOF), however recent studies have identified extent of QRS fragmentation (fQRS) to be superior for mortality prediction. This project aims to explore cohort level trends in long-term follow-up of patients with TOF and compare trends in patients with a transannular patch (TAP) at complete repair.

**Methods:** All available electronic ECGs recorded between 2012 and 2019 in patients with usual TOF repaired at Birmingham Children’s Hospital since 1988 were included. QRSd was computed digitally using the Glasgow Interpretive Algorithm and each lead was individually magnified and assessed for fQRS.

**Results:** 2,382 ECGs were included from 530 patients with a median age of 14 years (5.1–23.0) at last ECG. 398 (75.1%) had TAP at repair. 113 (21.3%) had undergone pulmonary valve replacement, at a median age of 20.1 (16.3–24.0) years. Pre-PVR, patients with a TAP showed a faster rate of increase in both QRSd and number of leads with fQRS at 15–25 years post-repair. Within one-year post-PVR, there was an average decrease in QRSd of 9 ms, but no change in leads with fQRS.

**Conclusions:** Patients with a TAP and chronic pulmonary regurgitation show an accelerated rate of increase in both QRS characteristics at 15–25 years after complete repair, similar to the average age at PVR (Fig. 1).

**Fig. 1:**
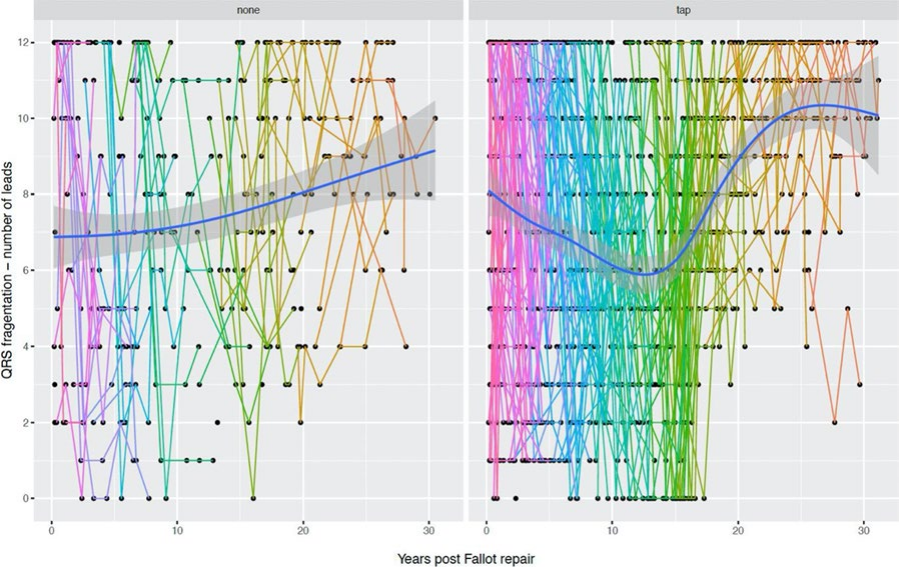
LOESS plot showing trend of total number of leads with fQRS over time since complete repair for patients who did not require a transannular patch at complete repair (n = 103, left) compared to those who required a TAP (n = 398, right)

## A178 Early palliation of paediatric patients with tetralogy of fallot in Malaysia: What is the role of transcatheter stenting and cardiac surgery?

### Aishah Mughal^1^; Ernest Ng^2^; Kan Chan^2^; Laura Mazalan^2^

#### ^1^University of Birmingham, Birmingham, UK; ^2^Serdang Hospital, Selangor, Malaysia, Selangor, Malaysia

**Correspondence**: Aishah Mughal

*Journal of Cardiothoracic Surgery 19(1):* A178

**Objectives:** Patients with Tetralogy of Fallot (ToF) can be palliated using cardiac surgery (ToF repair or Modified Blalock-Taussig (MBT) shunt) or transcatheter stenting (right ventricular outflow tract (RVOT) stenting or patent ductus arteriosus (PDA) stenting). There is a paucity of data comparing outcomes following these interventions which are representative of the Malaysian population. This study aims to investigate the types of treatment modalities used to provide early palliation in patients with ToF in Malaysia.

**Methods:** A retrospective observational study was performed on children diagnosed with ToF (21/05/2020–10/05/2022) at Serdang Hospital, Malaysia. Data was collected from electronic healthcare records. Statistical analysis was performed using SPSS.27.

**Results:** Forty-seven ToF patients were identified during the study period. Of these, 55.3% were female and extra-cardiac anomalies were reported in 27.7% of patients. Mean oxygen saturation prior to intervention was 81.9%. 57.4% of patients underwent ToF repair, 23.4% received MBT shunt, 12.8% received PDA stenting and 6.4% underwent RVOT stenting. MBT shunting and ToF repair was associated with a significant increase in mean oxygenation saturation following surgery (p < 0.05). ToF repair and PDA stenting had the shortest hospital admission length, however, RVOT stenting was associated with the highest proportion of post-procedural complications. Only one case of procedure-related mortality was observed across the cohort and 97.8% were alive at the time of follow-up.

**Conclusions:** Most patients with ToF are commonly treated using ToF surgical repair than transcatheter stenting. Although clinical outcomes following cardiac surgery appears to be superior to transcatheter stenting, further research is required across a larger sample size to confidently compare clinical outcomes between these palliative interventions.

**Thoracic Benign**

## A179 Current predictors of outcome in surgically managed patients with parapneumonic pleural empyema

### Michael Schweigert^1^; Ahmed Hamdouna^1^; Attila Dubecz^2^; Ana Beatriz Almeida^1^; Helmut Witzigmann^3^; Hubert J. Stein^2^

#### ^1^University Hospital Schleswig–Holstein; ^2^Klinikum Nuremberg; ^3^Klinikum Dresden-Friedrichstadt

**Correspondence**: Ahmed Hamdouna

*Journal of Cardiothoracic Surgery 19(1):* A179

**Objectives:** Parapneumonic pleural empyema is a critical condition with substantial morbidity and mortality. Surgical intervention is often complicated by multimorbidity, old age, frailty and advanced septic disease.

**Methods:** In a retrospective study from a prospective database the outcome of surgically managed patients with parapneumonic pleural empyema at four German tertiary referral hospitals was analyzed. Study period was 2006 – 2021.

**Results:** There were 571 patients (female 176, male 395). Mean age was 60.57 years. Multimorbidity (mean Charlson Score 2.48) with predominately cardiac conditions (222/571), diabetes mellitus (131/571), neurological disorders (100/571) and COPD (100/571) was common. VATS, open decortication and window thoracostomy were carried out in 437, 120 and 14 cases, respectively. ICU admission (325/571), mean ICU days (11.6), mean LOS (25.3 days) and in-hospital-mortality (47/571) underline the severity of the condition. Sepsis (OR 9.92; 95% CI: 4.99–19.72; p < 0.01), respiratory failure (OR 21.47; 95% CI: 10.26–44,98; p < 0.01), acute renal failure (OR 9.54; 95% CI: 4.79–18.99; p < 0.01), ICU stay > 1 day (OR 13.17; 95% CI: 5.49–31.61; p < 0.01) and Charlson Score ≥ 3 (OR 5.34; 95% CI: 2.71–10.55; p < 0.01) had higher odds for fatal outcome. Multimorbidity (Charlson Score ≥ 3) was also associated with significantly higher odds for sepsis and ICU admission. In contrast, advanced age (≥ 80 years) was not associated with higher mortality (11/83 vs. 36/488; p = 0.08).

**Conclusions:** Delayed referral for surgery with already advanced septic disease as well as pre-existing multimorbidity are the main reasons for fatal outcome. The most promising option for improving results is to avoid the occurrence of pulmonary sepsis by means of timely surgical intervention.

## A180 Endobronchial lung volume reduction: be prepared to have a "second look"

### Michelle Lee; Al Rehan Dhanji; Marco Nardini; Periklis Perikleous; Ralitsa Baranowski; David Waller

#### Barts Thorax Centre, St Bartholomew's Hospital, London, UK

**Correspondence**: Michelle Lee

*Journal of Cardiothoracic Surgery 19(1):* A180

**Objectives:** Endobronchial lung volume reduction (EBLVR) is an effective treatment in patients with severe emphysema but revisional (redo) bronchoscopy may be necessary. We aimed to identify more about the indications and outcomes of these procedures.

**Methods:** We prospectively collected follow-up clinical data including the indication and outcome of redo bronchoscopy in a cohort study of 72 consecutive patients undergoing EBLVR. We compared the characteristics of those requiring redo bronchoscopy with the remainder and looked for predictive factors of a successful outcome.

**Results:** Redo bronchoscopy was required in 29 of 72 (40%) patients at a median interval of 5(0–47) months The indications were: loss of initial benefit in 16 (55%); no initial benefit in five (17%); symptomatic intolerance (including haemoptysis) in six (21%) and secondary infection two (7%). The findings at redo bronchoscopy included: valve blockage in 55% (nine due to mucus, seven due to granulation), valve expectoration six (21%), leakage four (14%) and colonisation one (3%). In two (7%) there were no specific findings. The need for redo bronchoscopy was associated with lower airways obstruction in more elderly patients.

In 70% (19/27) there was both subjective and objective (lobar collapse) improvement. Of the remaining eight patients, six patients are followed up to date. Three had no subjective improvement despite radiological lobar collapse assessment and in three even redo valve insertion could not achieve collapse (Table 1).

**Table 1:** Pre-operative patient demographics

Median (range)	No redo bronchoscopy	Redo bronchoscopy	p-value
Number	43 (25M:18F)	29 (16M:13F)	0.8031
Age (Year)	63 (43–81)	72 (54–82)	**0.0499**
BMI (kg/m^2^)	22.1 (13–37)	24.6 (15.8–34.4)	0.1614
LUL:LLL	24:10	8:7	0.2422
RUL:RLL	7:2	9:5	0.4925
Upper:Lower	31:12	17:12	0.2342
FEV1 (%predicted)	30 (13.8–63)	35.3 (17–62)	**0.0110**
DLCO (%predicted)	36.9 (10.7–84)	38 (19–61)	0.3144
RV (%predicted)	200 (43.2–350.6)	211 (130–289)	0.1316

**Conclusions:** The need for revisional bronchoscopy after initial EBLVR is frequently required but unpredictable. Patients need to be properly informed and kept under surgical follow up for change in symptoms. Redo bronchoscopy is justified and beneficial but is most useful in those who have lost an initial improvement.

## A181 Is open surgery the optimal approach for the treatment of descending necrotising mediastinitis in the minimally invasive era?

### Savvas Lampridis; Marie Becker; Nabih Berjaoui; Ricard Simo; Andrea Bille

#### Guy's Hospital, London, UK

**Correspondence**: Savvas Lampridis

*Journal of Cardiothoracic Surgery 19(1):* A181

**Objectives:** To evaluate the postoperative outcomes of open surgery for the treatment of descending necrotising mediastinitis.

**Methods:** We retrospectively reviewed consecutive adult patients who underwent open surgery for descending necrotising mediastinitis. Preoperative planning was based on contrast-enhanced computed tomography of the neck and thorax. In general, widespread necrotising process extending the length of the posterior mediastinum was approached via a posterolateral thoracotomy, whereas disease confined in the superior mediastinum was managed through a cervical incision. All patients received empiric antibiotic treatment that was subsequently adjusted according to drug susceptibility tests.

**Results:** From September 2015 to July 2022, 17 patients (13 men) with a mean ± SD age of 49.3 ± 17.6 years underwent surgery for descending necrotising mediastinitis secondary to deep neck space infection. Of those, 14 (82.4%) patients underwent thoracotomy and three (17.6%) underwent cervicotomy. Four (23.5%) patients had associated empyema and underwent additional debridement and lung decortication at the same stage. All postoperative admissions to the Intensive Care Unit (n = 12) were planned, with a median length of stay of 12 days (IQR, 4–34 days). Grade III or IV complications as per the Clavien-Dindo classification were recorded in 17.6% of the patients (one reoperation for cervical exploration and two bronchoscopies under general anaesthesia for atelectasis). The median length of hospital stay was 22.5 days (IQR, 14–38 days). There were no 30-day hospital readmissions, and only one (5.9%) patient died within 90 days after surgery.

**Conclusions:** Open surgery for descending necrotising mediastinitis is associated with favourable postoperative outcomes, while it can provide improved access compared to minimally invasive approaches.

## A182 Pathogenesis of empyema thoracis and the path to decortication: a 6-year perspective from an irish tertiary hospital cardiothoracic surgery unit

### Gráinne Keehan; Jack Whooley; Alan Soo

#### Galway University Hospital, Galway, Ireland

**Correspondence**: Gráinne Keehan

*Journal of Cardiothoracic Surgery 19(1):* A182

**Objectives:** Pleural empyema remains a significant healthcare burden associated with substantial morbidity and mortality. The aim of this study was to explore the clinical characteristics, microbiological profile and aetiology of empyema thoracis in patients referred for surgical intervention; and to determine whether any changes have been seen in these features since the onset of the Covid-19 pandemic.

**Methods:** This single centre retrospective observational study was conducted between June 2016 and June 2022. Patients with empyema were identified through a search performed on the hospital’s electronic clinical information system.

**Results:** Patients eligible for inclusion *(n* = *46)* were primarily male *(80.43%),* active smokers *(54.35%)* with a mean age of *58.17 years (SD 13.99).* A reduction in the number of patients presenting with pleural empyema requiring decortication around or after the onset of Covid-19 was noted, when compared to the previous 3-year period (*n* = *16 vs. n* = *30*), with a reduction in mean-time from initial diagnosis to referral to a cardiothoracic centre also seen *(16.31 vs. 21.68 days).* Patients undergoing decortication after June 2019 tended to be at earlier stages of disease progression when compared to the previous 3-year period (25% stage III disease vs. 56.67% stage III disease). The majority of cases occurred in the context of a para-pneumonic effusion (67.39%). Less common causes included post-traumatic (21.73%), post-operatively (2.17%), or as a consequence of oesophageal perforation (2.17%), retropharyngeal abscess (2.17%) or a subphrenic abscess (4.35%). Microbiological diagnosis was obtained in 34.78%.

**Conclusions:** This study provides an interesting insight into the current clinical characteristics, microbiology and aetiology of empyema. Early recognition and triage down appropriate pathways is central to patient outcomes in empyema. Our study suggests that Covid-19 pandemic may be associated with earlier recognition of this complex disease.

## A183 Rib fractures management in the elderly population: a single centre series

### Ee Phui Kew^1^; Ian Hunt^2^

#### ^1^Guys and St Thomas NHS Trust, London, UK; ^2^St George's Hospital London, London, UK

**Correspondence**: Ee Phui Kew

*Journal of Cardiothoracic Surgery 19(1):* A183

**Objectives:** Rib fracture is associated with significant morbidities and mortality, especially in the geriatric population. Current literature suggests that rib fixation for flail chest reduced mortality, intensive care stay and incidence of pneumonia. However, the indication for surgery in the elderly population is not clear. We aim report the outcomes of rib fractures in patients above the age at 65 in our centre.

**Methods:** A single centre, five-year retrospective study was conducted. It included all trauma patients with rib fractures above the age of 65 years-old, admitted between 1st Jan 2017 and 31st Dec 2021. Demographic data such as sex, age, injury severity score (ISS), probability of survival score (PS) and number of rib fractures were recorded. Primary outcome was inpatient mortality. Secondary outcomes were length of hospital stay (LOS) and length of critical care stay (LOSCC).

**Results:** A total of 875 patients was included, with 45.4% female, mean age of 80.2 years old, mean number of rib fractures of 4.57, mean ISS of 21.2, and mean PS of 84.0%. The overall inpatient mortality was 16.6% (n = 145). The mean LOS was 18 days and LOSCC was two days. 2.1% (n = 18) patients underwent surgical rib fixation, and three of them were aged 80 and above. When the surgical group was compared with the non-surgical group, there was no significant statistical difference in the ISS, PS, LOSCC and LOS. The inpatient mortality was higher in the non-surgical group, with 16.9% compared to 0% in the surgical group, but there was no statistical significance (p = 0.056). The main differences between the two groups were the lower mean age (72.1 vs 80.4; p < 0.001) and higher number of rib fractures (6.5 vs 4.5; p = 0.003) in the surgical group.

**Conclusions:** Rib fixation confers good outcome in selected geriatric population. There is a trend towards lower mortality in the surgical group. We should consider rib fixation in elderly patients who have good functional status and low co-morbidities.

## A184 Thoracoscopic first rib resection for thoracic outlet syndrome: a single surgeon experience

### Jean-Luc Duval; Tenisha Joseph; Dominic Howard; Francesco Di Chiara

#### John Radcliffe Hospital, Oxford, UK

**Correspondence**: Jean-Luc Duval

*Journal of Cardiothoracic Surgery 19(1):* A184

**Objectives:** Thoracoscopic first rib resection (FRR) has several advantages and is an emerging option for minimally invasive surgical management of thoracic outlet syndrome (TOS), however only three centres in the UK currently offer this procedure. A single surgeon at our institution introduced the thoracoscopic FRR in 2019. This paper seeks to review the first three years outcomes of thoracoscopic FRR and demonstrate safety and efficacy of this technique.

**Methods:** Retrospective analysis of case notes of all patients undergoing thoracoscopic FRR between 2019 and 2022.

**Results:** Thirteen patients (male = 6, median age = 36) underwent thoracoscopic FRR between August 2019 and September 2022 after discussion at Vascular MDT. The indication for all cases was venous TOS and three patients had undergone previous supraclavicular FRR. The median length of stay was three days with no significant inpatient complications. Freedom from ongoing symptoms was reported by 76% (n = 10) of patients at latest follow-up. There was one case of delayed bleeding post-discharge requiring chest drain insertion and return to theatre for washout of haemothorax.

**Conclusions:** Thoracoscopic first rib resection appears to be a safe and effective minimally invasive surgical management option for venous TOS.

## A185 Chest wall reconstruction: 8-year experience at major trauma centre

### Avishek Samaddar; Rana Mehdi; Qingzi Guo; Andrew Chrisp; Kajan Mahendran; Shilajit Ghosh; Udo Abah; Lakshmi Srinivasan

#### University Hospitals of North Midlands NHS Foundation Trust, Stoke on Trent, UK

**Correspondence**: Avishek Samaddar

*Journal of Cardiothoracic Surgery 19(1):* A185

**Objective:** The NICE guidelines for management of traumatic rib fractures date back to 2010. Although the evidence base is improving, there is still heterogeneity in practices, particularly between trauma and non-trauma centres. Hence, the indications for rib fixation are not firmly established. We present our experience from a high-volume Major Trauma Centre.

**Methods:** Data from all consecutive chest trauma patients undergoing reconstruction over past eight years was collected from prospectively filled database, excluding delayed repairs over one month, isolated sternal fractures and redo operations for ensuring uniformity in patient presentations. Statistical analysis was done in R statistical software (packages GLM and CARRoT).

**Results:**

**Table:** 

	All chest trauma (245)	Isolated chest trauma (87)	Poly-trauma (158)	p value
Overall Complications (%)	18.8%	13.8%	21.5%	0.19
LRTI (%)	4.9%	2.3%	6.3%	0.22
Tracheostomy (%)	13.9%	8.0%	17.1%	0.07
Empyema (%)	0.4%	0.0%	0.6%	1
Return to theatre (%)	2.4%	1.1%	3.2%	0.43
Post-operative Ventilation (%)	31%	18.4%	38%	0.002
Median Hospital stay (IQR	7 (4–16)	5 (3–8)	9 (2–23)	
Median ITU stay (IQR)	1 (0–6)	0 (0–0)	2 (0–9.75)	
In-hospital Mortality	11 (4.4%)	2 (2.3%)	9 (5.7%)	

On multi-variate analysis age, preoperative ventilation, head injury, sternal injury and number of ribs fractured showed to be statistically significant predictors for mortality and post-operative complications.

**Conclusions:** Chest wall reconstruction can be successfully performed in high-volume major trauma centres across all age groups. Post-operative results are better in the isolated chest trauma group. Age, pre-operative ventilation, head injury, number of ribs fractured/fixed, and associated requirement of sternal fixation are important predictors of outcome.

## A186 Erector spinae plane block in thoracic surgery: a single centre short-term experience

### Anchal Jain^1^; Avishek Samaddar^2^; Shilajit Ghosh^2^; Juneenath Karattuparambil^2^; Lakshmi Srinivasan^2^

#### ^1^Addenbrooke's Hospital, Cambridge University NHS Trust, Cambridge, UK; ^2^University Hospitals of North Midlands NHS Foundation Trust, Stoke on Trent, UK

**Correspondence**: Avishek Samaddar

*Journal of Cardiothoracic Surgery 19(1):* A186

**Objective:** Erector spinae plane block is a simple and effective form of regional anaesthesia with expanding application. Post-operative pain management is the cornerstone to successful recovery in thoracic surgical patients. Recent studies have shown its effectiveness in thoracic surgery. We present our short-term experience of usage of depot and catheter ESB in VATS and open thoracotomy cases.

**Methods:** A total of 44 consecutive patients undergoing a thoracic surgical procedure in whom some form of Erector Spinae Plane Block was given were followed up to assess their post-operative recovery. Records were kept regarding the type of surgery, VATS or open thoracotomies. Post-operative pain scores in the recovery room and up to 48 h were recorded along with the mobility and functional status. Records were also kept for any adverse events. Statistical analysis was done in R statistical software (packages GLM and CARRoT).

**Results:**

**Table:** 

	All patients with erector spinae block (44)	Erector spinae depot (18)	Erector spinae catheter (26)
VATS/Open Thoracotomy %	26 (59%) /16 (36.3%)	8 (44.4%) /8 (44.4%)	18 (69.2%) /8 (30.8%)
Median Pain Score 1 Hour	3	2	3
Median Pain Score 4 Hours	1	1	1
Median Pain Score 12 Hours	1	1	1
Median Pain Score 24 Hours	0	0	0
Median Pain Score 48 Hours	0	0	0
Median Post-operative Day when sitting	1	1	1
Median Post-operative Day when discharged from physiotherapy	2	2	3
Opioid Requirement Post-operative Day 1 (%)	37 (84%)	12 (66.6%)	25 (96%)

There was a single mortality. Although patient-controlled analgesia was used in 28 patients, the overall requirement was considerably low compared to patients where no ESB was used.

**Conclusions:** Erector Spinae Plane Block can be used as a safe and effective form of pain management in both VATS and open thoracic surgeries. Enhanced recovery with early mobility and significant pain relief was achieved. However, larger studies with higher patient populations are required to compare between depots and catheters.

## A187 Lung volume reduction surgery: a micro-costing analysis from a national tertiary referral centre

### Kathryn Mulryan^1^; Jan Sorensen^2^; David Waller^3^; Karen Redmond^4^

#### ^1^Mater Misericordiae University Hospital, Dublin, Ireland; ^2^Healthcare Outcome Research Centre, RCSI University of Medicine and Health Sciences, Dublin, Ireland; ^3^Department of Cardiothoracic Surgery, St Bartholomew's Hospital, London, UK; ^4^National Thoracic and Transplant Centre, Mater Misericordiae University Hospital, Dublin, Ireland

**Correspondence**: Kathryn Mulryan

*Journal of Cardiothoracic Surgery 19(1):* A187

**Objectives:** Lung volume reduction surgery (LVRS) is a clinically effective palliation procedure for patients with chronic obstructive pulmonary disease (COPD). LVRS has recently been commissioned by the NHS England. In this study, a costing model was developed to analyse cost and resource implications of different LVRS procedures.

**Methods:** Three pathways were defined by their surgical procedures: bronchoscopic endobronchial valve insertion (EBV-LVRS), video assisted (VATS-LVRS), and robotic assisted LVRS (RATS-LVRS). The costing model considered use of hospital resources from the LVRS decision until 30-days after hospital admission. The model was calibrated with data obtained from an observational study, electronic health records, and expert opinion.

**Results:** VATS-LVRS was associated with the lowest cost at €12,896 per patient. This compares to the costs of EBV-LVRS at €15,598 per patient and €13,305 per patient for RATS-LVRS. This study presents a comprehensive framework for the analysis of hospital-related resource use and costs for the three surgical modalities.

**Conclusions:** In the future, service commissioning agencies, hospital management and physicians can use this framework to determine their modifiable resource use (composition of surgical teams, use of staff and consumables, planned length of stay, and revision rates for EBV-LVRS) and to assess the potential cost implications of changes in these parameters.

## A188 COVID pandemic drives introduction of regional cryo-biopsy service for the diagnosis of interstitial lung disease

### Salah Al-Haddi^1^; Faisal Jawad^2^; Richard Steyn^2^; Ehab Bishay^2^; Maninder Kalkat^2^; Hazem Fallouh^2^; Vanessa Rogers^2^; Ashvini Menon^2^; Babu Naidu^2^

#### ^1^University of Birmingham / University Hospitals Birmingham (UHB), Queen Elizabeth Hospital, UK; ^2^Queen Elizabeth Hospital, Birmingham, UK

**Correspondence**: Salah Al-Haddi

*Journal of Cardiothoracic Surgery 19(1):* A188

**Background:**

- Thoracic surgery services took a heavy hit during the pandemic. Prioritisation of services meant that surgery was being offered solely for emergency and cancer patients. Those with ‘benign’ lung disease were seemed low priority as in patient beds became very limited and their care has undoubtedly been harmed.
- Bronchoscopic cryo-lung biopsy is a technique which is performed as a day case and is associated with comparable diagnostic yields but reduced morbidity and length of stay.

**Objectives:**

- To set up a regional Cryo-lung biopsy service.
- To compare it with or own historical data for VATS lung biopsy as well as international standards for cryo-biopsy.

**Methods:**

- Inclusion criteria: FEV1 & TLCO > 35%, MDT approved target.
- Standardising four biopsy samples for every patient.
- A Chest X-ray usually done on recovery to assess for pneumothorax.

**Results and Discussion:**

- 17 cases
- Average Age 62, DLCO 49–91% and FEV1 51–115%.
- Average theatre time 20 min (15–31 min).
- 13/17 patients were discharged same day, four required post-op admission; one developed an exacerbation of their ILD, one who had a small pneumothorax managed conservatively and two patients that required chest drain insertion for pneumothorax.
- In each case four biopsies were taken from at least two lobes. The size of sample varied from 3 to 8 mm.
- There were no other surgical complications or significant bleeding.
- 94% diagnostic accuracy on par with 83% in meta-analysis study.
- 15% of Cryo-cases developed moderate-severe pneumothorax comparable to 12% in Meta- analysis study.
- Median length of stay was 0.4 days in comparison to two days for VATS lung biopsy (local audit).

**Conclusions:** Cryo-lung biopsy so far seems to be a safe and viable alternative to lung biopsy and will help facilitate with ILD diagnosis in a resource strapped NHS.

## A189 Modelling the healthcare system benefit of enhanced recovery & day of surgery admission in thoracic surgery

### Rebecca Koh; Claudia Pama; Giuseppe Aresu; Adam Peryt; Piergiorgio Solli; Aman Coonar

#### Royal Papworth Hospital, Cambridge, UK

**Correspondence**: Rebecca Koh

*Journal of Cardiothoracic Surgery 19(1):* A189
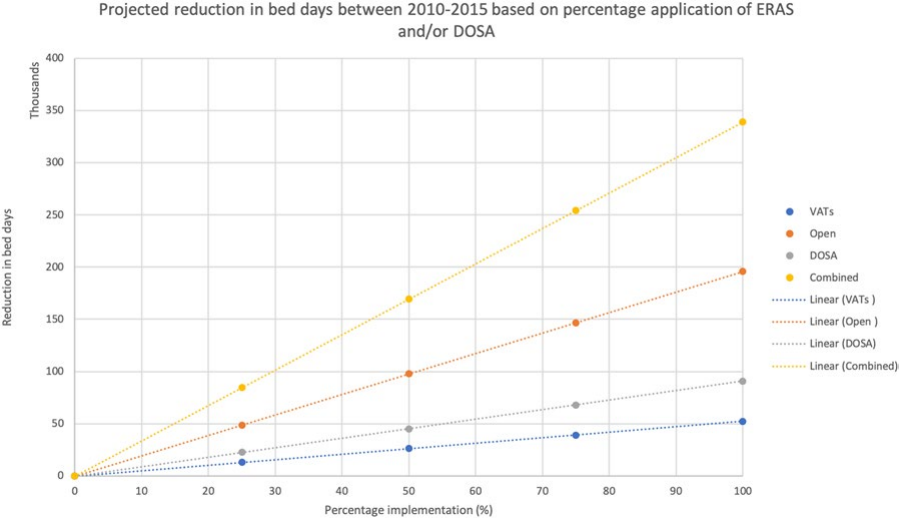


This study aims to model potential savings in hospital occupancy and money if Enhanced Recovery After Surgery (ERAS) and Day of Surgery Admission (DOSA) were more widely implemented in SCTS Thoracic Surgery units.

**Methods:**

1. A literature review was performed to estimate the reduced length of stay (LOS) arising from ERAS in thoracic surgery. We assumed that DOSA produced a one-day reduction in hospital stay/elective procedure. Using historical data we estimated the rate of elective procedures to be 73.6%.
2. In the 2010–2015 SCTS Thoracic Surgery Report there were 51,856 VATS and 71,470 open procedures (total 123,326). We modelled the possible benefits based on that report, and considered different levels of ERAS and DOSA performance.

**Results:**

1. A literature review found relevant papers which estimated the reduction in LOS arising from ERAS in thoracic surgery as 1.3–3.3 days (weighted mean 2.3). The reduction in LOS for VATS is associated with a reduction in LOS of 1 day compared to open surgery which is 2.7 days.
2. At a 75% performance rate (v. 0%) for ERAS after thoracic surgery we estimated a LOS reduction of 39,281 days for VATS procedures and 146,870 days for open procedures. At a performance rate for DOSA in elective thoracic surgery we estimated a LOS reduction of 68,076 days. The total reduction in LOS was 254,227 days.

Using the 2018 NHS reported cost of £406/day for ward care we estimated a total financial saving of £103,216,455 based on a 75% performance rate of both ERAS and DOSA.

Different levels of performance produced different LOS reduction as shown in the graph.

**Conclusions:** DOSA and EAS are variably implemented in thoracic surgery. The NHS aims for at least 75% adherence. If this could have been achieved in the evaluation period the potential hospital occupancy savings could have been at least 254,227 days and the corresponding financial saving at least £103,216,455.

## A190 COPD community referral to specialist assessment: a service evaluation for handheld spirometry

### Eliana Peus; Paul Bontoft; Hannah Burke; Tom Havelock; Edwin Woo; Aiman Alzetani

#### University Hospital Southampton, Southampton, UK

**Correspondence**: Eliana Peus

*Journal of Cardiothoracic Surgery 19(1):* A190

**Objectives:** The Targeted Lung Health Check (TLHC) screening program has been launched in centres around UK. At one of those centres just under 10% of patients were radiologically detected with moderate to severe emphysema. Pulmonary function tests (PFT) are essential for clinical diagnosis of COPD. This study aims to evaluate the use of a point of care-based test for lung function to see if it is a practical, reliable and economical way to help with prioritising referrals to formal PFTs and thereafter specialist respiratory services for more advanced management including Lung Volume Reduction.

**Methods:** Participants from a local TLHC program detected with moderate to severe emphysema on low dose CT scans, with no diagnosis of COPD, were recruited to take part in PFT with the handheld spirometry device at their existing PFT appointment. Results of the handheld spirometer were compared with hospital spirometer.

**Results:** From the start of the TLHC, in January 2020 until September 2022, a total of 6262 low dose CT scans have been performed on current or previous smokers aged 55 to 74. Moderate or severe emphysema was detected on 556 patients. These had COPD specialist referrals, 267 of which have had no previous COPD diagnosis. The average waiting time between a scan until the referral is 7.7 months. Out of 60 patients attending the PFT 14 were suitable for this handheld spirometry. Of these, seven patients had an FEV1/FVC ratio < 0.7 and five had an FEV1/FVC ratio < 0.6 which is classified as moderate COPD according to the GOLD criteria.

**Conclusions:** The study identifies the handheld spirometers as a simple and practical device that can aid the diagnoses of asymptomatic COPD patients. These devices have the potential to reduce pressure on the referral system by streamlining which patients need to be seen sooner and have more formal PFTs. This may allow for earlier clinical diagnosis and more specialist interventions leading to better outcomes.

## A191 Service evaluation of post operative management of deep vein thrombosis (DVT) and pulmonary embolism (PE) post thoracic surgery

### Muhsin Zaiq^1^; Aiman Alzetani^2^; Bradley Yee^2^; Martin Chamberlain^2^; Hannad Ahmed^2^

#### ^1^University of Southampton, Southampton, UK; ^2^University Hospital Southampton, Southampton, UK

**Correspondence**: Muhsin Zaiq

*Journal of Cardiothoracic Surgery 19(1):* A191

**Aims:** The aim of this service evaluation is to see the incidence of DVT/PE in patients post thoracic surgery and its management beyond discharge for up to six months at a regional cardiothoracic surgery department.

**Methods:** Data was collected on all adult patients admitted for thoracic surgery who subsequently developed DVT/PE post-surgery from 2017. Patients with existing DVT/PE and/or patients on long term anti-coagulation at the time of their surgery were excluded. Data collected included patient demographics, co-morbidities, date of surgery, outcomes of surgery, date of discharge/death, presence of complications, any cases of readmission, perioperative DVT/PE prophylaxis, and any evidence of DVT/PE up to 6 months from the procedure date.

**Results:** Between Jan 2017- Aug 2017 there were 581 cardiothoracic procedures carried out. Out of these 7(1.20%) patients presented with either DVT, PE or both. There were two cases with DVT and four with PE. One patient had both DVT & PE. There was three males and four females. One patient died of a PE. The average time interval from operation to DVT/PE was 69.43 days with 3.00 days being the shortest and 157.00 days being the longest. None of these patients have had extended pharmacological prophylaxis post procedure but were provided with TED stockings for up to four weeks. The patients all underwent major thoracic procedures such as (Flexible Bronchoscopy, VATS Mediastinoscopy and Rigid Bronchoscopy).

**Conclusions:** This service evaluation demonstrates the prevalence of DVT/PE post thoracic surgery. Although there was extended mechanical prophylaxis (TED Stockings) post discharge there was no evidence of a pharmaceutical one. This highlights the need to create some guidelines that identify high risk patients who would benefit from extended prophylaxis as has been done in other surgical specialties in the UK. More research with a bigger patient group over a longer period is required to develop such guidelines/protocols.

## A192 Establishing an expert opinion framework for lung volume reduction in ireland: a Delphi

### Kathryn Mulryan^1^; Jan Sorensen^2^; Karen Redmond^3^

#### ^1^Mater Misericordiae University Hospital, Dublin, Ireland; ^2^Health Research Outcomes Centre, Royal College of Surgeons in Ireland, Dublin, Ireland; ^3^National Thoracic and Transplant Centre, Mater Misericordiae University Hospital, Dublin, Ireland

**Correspondence**: Kathryn Mulryan

*Journal of Cardiothoracic Surgery 19(1):* A192

**Objectives:** Lung volume reduction (LVR) is an effective treatment offered to patients with emphysema. An approach to agreeing and implementing an LVR referral framework in an Irish context is required. A Delphi process facilitates consensus development using expert opinion. The aim of this study was to develop recommendations for LVR referral guidelines in an Irish context based on current practice and evidence.

**Methods:** In accordance with Guidance on Conducting and Reporting Delphi Studies, a Delphi study was performed. 33 statements informed from review of research literature were identified and presented to participants. Evaluation of the statements was performed by an expert panel using a Likert scale. ≥ 70% agreement was defined as consensus and items with a consensus rating < 70% were revised. Questionnaires were distributed to 18 experts with a response rate of 78% (n = 14) and a second-round response-rate of 50%.

**Results:** The expert panel consisted of representatives from respiratory medicine, cardiothoracic surgery and allied-health professionals with expertise in COPD care. Of the initial 33 statements in five dimensions, there were consensus regarding 31 statements.

**Conclusions:** The 31 statements agreed through this study clarify a coherent direction to develop an LVR framework in Ireland. The Delphi process is a useful tool to reach consensus among multi-disciplinary experts.

## A193 Enhanced recovery in thymectomy for myasthenia gravis: How far can we go?

### Rhona Taberham; Ramanjit Kaur; Dionisios Stavroulias

#### Oxford University Hospitals, Oxford, UK

**Correspondence**: Rhona Taberham

*Journal of Cardiothoracic Surgery 19(1):* A193

**Objectives:** Minimally invasive thymectomy is a well-established treatment option for myasthenia gravis (MG). Historically, these patients were routinely admitted to intensive care (ICU) post-operatively. At our institution, we changed this practice in 2018 and evaluated its safety.

**Methods:** We retrospectively reviewed 107 patients undergoing VATS thymectomy for MG between November 2013 and November 2022. We compared patients sent to ICU (pre-2018) against those who were not. Outcomes including development of myasthenic crisis, need for ICU and length of stay were reviewed.

**Results:** 57 patients were admitted to ICU and 50 were not. Baseline characteristics were similar in the two groups. Of those sent directly to the ward, no patient required admission to ICU. No patient suffered a myasthenic crisis. Length of stay was significantly shorter in the ward group (2.08 vs. 2.58 days, p = 0.0395). 30% (15/50) of ward patients were discharged on the first post-operative day and this rose to 74% (37/50) by the end of the second day.

**Conclusions:** We have shown it is safe to avoid the use of an ICU bed post-operatively in VATS thymectomy for MG. Most patients can be discharged home on the first or second post-operative day. Should we consider day-case thymectomy?

## A194 Is pulmonary hypertension a contraindication to proceeding with lung volume reduction surgery (LVRS)? A pilot study

### Jose Alvarez Gallesio; Abdullah Alshammari; Olotuntobi Rotimi; Aurelien Gueroult; Hema Chavan; Chiara Proli; Aina Pons; Simon Jordan; Sofina Begum

#### Royal Brompton, London, UK

**Correspondence**: Jose Alvarez Gallesio

*Journal of Cardiothoracic Surgery 19(1):* A194

**Objectives:** Historically, pulmonary hypertension was a contraindication for LVRS. This paper aims to present the outcomes for a single institution LVRS modified protocoled pathway, which included selective patients with preoperative pulmonary hypertension.

**Methods:** Data extracted retrospectively and a follow-up evaluation prospectively for every patient who underwent LVRS from February–October 2022 following the modified LVRS protocol. Patients with a preoperative echocardiogram with high pulmonary artery pressure, normal size, and right ventricle function were also included under the new protocol. An echocardiogram was performed after a month to compare the outcomes. Preoperative and postoperative lung function, quality of life (CAT score), morbidity, and mortality were analysed.

**Results:** Of 25 patients who underwent LVRS, eight had preoperative pulmonary hypertension. In the pulmonary hypertension group, the mean systolic pulmonary artery pressure decreased from 38 (36–45) to 24.2 (28–35) mmHg after surgery. The postoperative morbidity was similar between the two groups, and the overall sixty-day mortality rate was 0. CAT score improved following LVRS in both groups.

**Conclusions:** LVRS could be safe in selective patients with preoperative pulmonary artery hypertension. A standard detailed perioperative protocol is mandatory to proceed in high-risk LVRS patients.

## A195 Can perioperative fluids improve clinical outcomes in patients following thoracic surgery? A single-centre propensity matched retrospective study

### Aishah Mughal^1^; Elisha Warner^2^; Ahmed El-Zeki^2^; Ahmed Oliemy^2^; Patrick Yiu^2^; Ahmed Habib^2^

#### ^1^University of Birmingham, Birmingham, UK; ^2^New Cross Hospital, Royal Wolverhampton NHS Foundation Trust, Wolverhampton, UK

**Correspondence**: Aishah Mughal

*Journal of Cardiothoracic Surgery 19(1):* A195

**Objectives:** The optimal approach for fluid management continues to remain a subject of controversy amongst surgeons. A restrictive fluid regime can predispose to acute kidney injury (AKI) requiring haemofiltration and ICU admission. There is limited data regarding the benefits of perioperative fluids in minimising these adverse events. We aim to evaluate outcomes of patients receiving perioperative fluids following thoracic surgery.

**Method:** A retrospective observational study was performed on 250 patients undergoing thoracic surgery (2/03/2022–31/08/2022). Propensity matching (age, sex, and anatomical lung resection) identified an intervention group of patients who received perioperative fluids (POF) and a comparator group that did not (non-POF). Clinical outcomes were investigated including AKI, haemofiltration and in-hospital mortality. Statistical analysis was performed using SPSS.V29.

**Results: **125 patients were identified in both POF and non-POF groups. The median age at operation was 73 (IQR 68–77) years. Most patients had a performance status of 0–1 (88.7 vs 88.4% for POF and non-POF groups, respectively). Four cases (3.2%) of stage 3 AKI were recorded in the POF group. Of these, all 4 cases received post-operative fluids with zero compliance to receiving pre-operative fluid. 1 patient required haemofiltration who only received post-operative fluids. Seven cases of in-hospital mortality were observed in patients receiving perioperative fluids, none were attributed to AKI. There were no statistically significant differences in clinical outcomes between POF and non-POF groups (Table 1).

**Table 1:** A propensity matched retrospective observational study to compare and evaluate outcomes of patients receiving perioperative fluids following thoracic surgery

Clinical outcomes	Perioperative fluids (POF) Group n = 125	Non-perioperative fluids (Non-POF) Group, n = 125	p-value
Post-Operative Acute Kidney Injury (Stage 3)	4 (3.2%)	5 (4.0%)	0.823
Requirement of Haemofiltration	1 (0.8%)	2 (1.6%)	0.809
Intensive Care Unit (ICU) Admission	7 (5.6%)	11 (8.8%)	0.232
Length of Hospital Admission, days [median (IQR)]	3 (2–7)	3 (2–7)	0.327
In hospital mortality	3 (2.4%)	4 (3.2%)	0.558

**Conclusions:** Whilst an improvement in clinical outcomes was observed following administration of POF, this did not reach statistical significance. Improvement is required to optimise compliance in patients requiring pre- and post-operative fluids. Further studies are required to ascertain the effectiveness of perioperative fluids in minimising adverse outcomes following thoracic surgery.

## A196 Do rib fractures leading to non-union elude usual indication criteria for rib fixation at time of acute presentation?

### Aly Pathan; Waldemar Bartosik; Vasileios Kouritas; Bartlomiej Szafron; Aleksander Mani; Jakub Kadlec

#### Norfolk and Norwich University Hospital, Norwich, UK

**Correspondence**: Aly Pathan

*Journal of Cardiothoracic Surgery 19(1):* A196

**Objectives:** Review of our unit experience with rib fixation for rib fractures since its implementation with focus on patients with rib fracture non-union.

**Methods:** Retrospective review of rib fixation with MatrixRibTM System titanium plates between September 2016 and September 2022. Patient records were reviewed, and descriptive statistics was used for analysis. Non-union was defined as non-healing fracture on CT scan in symptomatic patients. Retrospective review at time of presentation was performed to clarify if these patients should have had surgical fixation as per Bemelman guidelines.

**Results:** Thirty-six patients had rib fixation. Of these, surgery was performed for acute injury in 28 (77.7%) patients (12 flail segment, 15 for multiple fractures and one haemothorax) and 8 (22.2%) for non-union. Mean number of rib fractures for early surgery was seven versus 3.5 for non-union fixation. The average time from injury to fixation was 4.0 days (range 1–13) for acute fracture and 354 days (range 138–802) for non-union. None of the patients with non-union would have met criteria for acute fixation as per Bemelman guidelines—all had undisplaced lateral fractures of ribs 7 to 10, with 9th rib most affected. There was no difference in gender or age from acute fracture patients. Seven of eight patients (87.5%) had significant pain improvement. On follow up, seroma, screw migration, broken plate and displacement of metalwork were noted.

**Conclusions:** From our limited experience non-union is more common amongst patients with lateral fractures of caudal ribs, especially the 9th rib. None of these patients would have met current criteria for fixation at time of trauma.

## A197 Sympathetic surgery for hyperhidrosis and facial flushing: A literature review on sympaticotomy, ramicotomy and clipping

### Mostin Hu; Guiseppe Aresu; Adam Peryt; Piergiorgio Solli; Aman Coonar

#### Royal Papworth Hospital / University of Cambridge, Cambridge, UK

**Correspondence**: Mostin Hu

*Journal of Cardiothoracic Surgery 19(1):* A197

**Objectives:** To conduct a contemporary literature review of sympathectomy to determine the optimal surgical method to reduce complications and achieve treatment success.

The first report of surgery on the upper sympathetic chain for the treatment of severe facial hyperhidrosis was in 1920 (1). Today, sympathetic surgery is an accepted treatment for facial flushing, craniofacial and upper extremity (palmar, axillary) hyperhidrosis following failure of medical interventions (2). Post-operative complications of compensatory hyperhidrosis (CH), Horner’s syndrome, and treatment failure are common (3), with some studies reporting moderate-severe CH in > 90% of patients (4,5). The 2011 STS consensus guidelines (6) do not suggest a preferred method of interruption (transection or clipping) or structure to interrupt (sympathetic chain, pre/postganglionic fibres, rami communicantes).

**Methods:** Medline and EMBASE were reviewed for thoracic sympathetic surgery. Information on target sympathetic level, treatment failure, % patients with CH and other post-operative complications was collected.

**Results:** 25 papers were identified: 8 comparative studies and 17 single method reports. Ramicotomy (transection of rami communicantes, leaving sympathetic chain intact) and approaches involving lower segments of the chain was associated with lower rates of CH compared to methods disrupting the sympathetic chain (4,5,7–10). Highly-selective approaches involving the transection of only postganglionic sympathetic fibres have achieved good preliminary results with none-mild CH and near 100% treatment success.
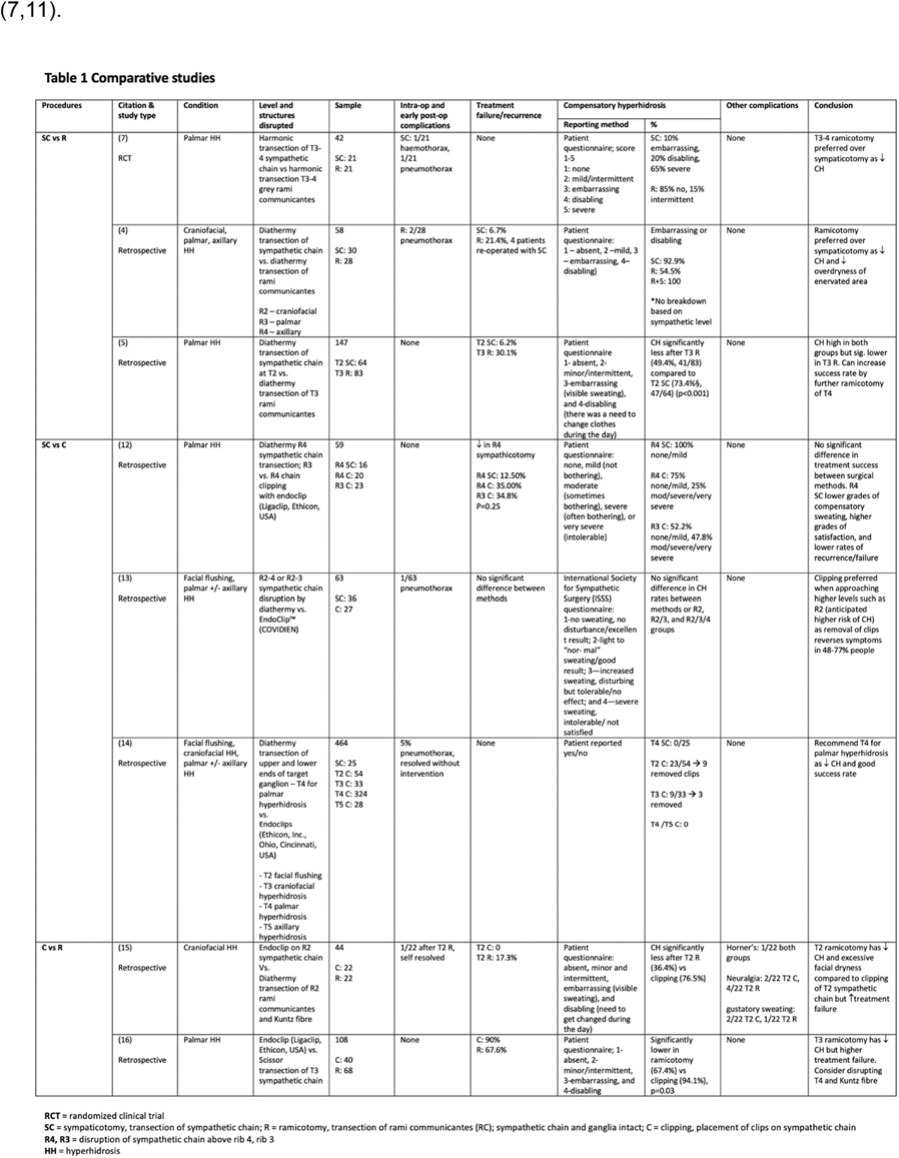


**Conclusions:** Thoracic sympathetic surgery is evolving with further study required to determine the optimal surgical method. A standardized follow-up period, inclusion of both subjective and objective measurement tools for CH (ex: Hyperhidrosis Disease Severity Scale (HDSS)), and the creation of a registry to permit data-sharing for future studies may improve outcomes.

## A198 VATS Nuss Bar procedure: our experience

### Soumik Pal; Srushti Bhat; Shyamsunder Kolvekar

#### St Bartholomew's Hospital, Barts Heart Centre, London, UK

**Correspondence**: Soumik Pal

*Journal of Cardiothoracic Surgery 19(1):* A198

**Background:** Pectus excavatum (PE) is a common congenital anterior chest wall deformity characterized by a sunken appearance. The incidence rate is approximately 0.1% with a male-to-female ratio of 4:1. It is associated with connective tissue disorders, neuromuscular diseases, and some genetic conditions.

In 1987, Dr Nuss and his colleagues invented the minimally invasive repair of pectus excavatum, also known as the Nuss procedure. This is a two-staged procedure that resulted in lesser hospital stays, smaller incisions, and early mobilization. Applying this procedure to video-assisted thoracoscopic surgery (VATS), we present our experience on insertion as well as removal of the Nuss bar in patients with PE.

**Methods:** This is a single-centre, retrospective study. We operated on a total of 177 patients between 2016 and 2021 (5 years). All the procedures were performed by a single consultant cardiac surgeon using biotech stainless steel bars.

**Results:** A total of 86 bar insertions and 91 bar removals were performed. The mean age range of patients was 16–43 years and the average hospital stay was 2–5 days (Table 1).

**Table 1:** Number of patients with bar insertion (48.6%) vs removal (51.4%)

Age (years)	Total	Male	Female	Bar insertions	Bar removals
< 18	43	42	1	41	2
18–29	99	94	5	37	62
30–44	28	25	3	8	20
> 45	7	6	1	0	7

Postoperative complications for bar insertions included pleural effusion (2.2%), pneumothorax (1.1%), bleeding (0.5%), bar dislodgement (2.2%), pericardial effusion (1.1%) and wound infection (4.5%).

**Conclusions:** VATS-Nuss procedure can be safely performed in teenage and adult patients resulting in shorter hospital stays and reduced post-operative complications.

## A199 Surgery for Empyema, a longitudinal study (SEALS)

### Jordan Green; Daniel Field; Azar Hussain; Mahmoud Loubani; Syed Qadri

#### Castle Hill Hospital/Hull University Teaching Hospitals NHS Trust, Hull, UK

**Correspondence**: Jordan Green

*Journal of Cardiothoracic Surgery 19(1):* A199

**Objectives:** There are several surgical management options available for thoracic empyema, mainly drainage or decortication, yet there is a paucity of robust evidence comparing either intervention thus far. This study aimed to determine the clinical effectiveness of isolated surgical drainage of empyema when directly compared to decortication (with drainage).

**Methods:** All patients undergoing surgery for empyema at 13 thoracic centres across the UK and Ireland between 2018 and 2019 were retrospectively audited. Patients were grouped into 'drainage' or 'decortication'. Primary outcomes were 30-day and one-year mortality. Secondary outcome measures were incidence of post-operative complications, empyema recurrence, rate of re-intervention, and post-operative length of stay. Kaplan–Meier survival analysis and multi-level regression techniques were used for analysis.

**Results:** 763 patients were included (drainage: 47.6%, *n* = 363; decortication: 52.4%, *n* = 400). Notably, only 30.8% of patients were staged pre-operatively (*n* = 235). Decortication was not associated with a significantly greater adjusted odds of mortality or survival between groups (*p* = 0.065). Decortication was associated with a higher rate of post-operative complications, 33.0% (*n* = 132) versus 24.2% (*n* = 88) [OR 1.92, 95% CI 1.19–3.10, *p* = 0.008]. Specifically, there were significantly higher rates of persistent air leak, 9.0% (*n* = 36) versus 3.3% (*n* = 12) [OR 3.12, 95% CI 1.47–6.62, *p* = 0.003). Empyema recurrence, rates of re-intervention, and post-operative length of stay were not associated with significant increases in odds between groups (*p* > 0.05).

**Conclusions:** Isolated surgical drainage of empyema has been demonstrated to be as clinically effective as decortication. Moreover, decortication is associated with greater odds of post-operative complications, notably air leak, which has significant clinical and cost implications. Future prospective studies are needed to examine these findings further.

## A200 Cryobiopsy in interstitial lung disease: a single-centre experience

### Devan Limbachia; Mostafa Ahmed; Luis Hernandez

#### University Hospital Coventry and Warwickshire, Coventry, UK

**Correspondence**: Mostafa Ahmed

*Journal of Cardiothoracic Surgery 19(1):* A200

**Introduction:** Cryobiopsy was initially used in our unit to biopsy COVID patients. After observing its safety and enhanced recovery, we utilised it in patients with Interstitial Lung Disease (ILD). 2022 European Respiratory Society guidelines suggest Cryobiopsy is a safer alternative to surgical lung biopsy in ILD.

**Objectives:** This audit aimed to identify the safety of Cryobiopsy in the diagnosis of ILD.

**Methods:** Patients undergoing Cryobiopsy between January 2021 and August 2022 were identified at a single UK thoracic centre. Prospective data collection with retrospective analysis was performed. Pre-operative factors, length of stay (LOS), intraoperative factors, diagnostic yield, post-operative factors and 30-day mortality was extracted.

**Results:** 18 patients underwent Cryobiopsy. Mean age was 57. All were discussed at ILD MDT. 100% had pre-operative CT imaging. 94% had preoperative dyspnoea with modal ASA 3. No patients had intraoperative complications. 100% of specimens were deemed high quality. 94% had full diagnostic yield, of which 61% were ILD and 17% were malignant.

Average LOS was two days. 17% had post-operative respiratory tract infection. One patient had post-operative pneumothorax, conservatively managed. There was no 30-day mortality.

**Conclusions:** Cryobiopsy is a safe alternative to surgical lung biopsy in the diagnosis of ILD, with 94% diagnostic yield at our centre.

## A201 The impact of a national programme which facilitated protected beds and theatre access for thoracic surgery

### Mistura Kareem; Orlaith Kavanagh; Karen Redmond; Rachel Brown; Donna Eaton

#### Mater Misericordiae University Hospital, Dublin, Ireland

**Correspondence**: Mistura Kareem

*Journal of Cardiothoracic Surgery 19(1):* A201

**Objectives:** In October 2020 the Health Service Executive introduced a programme to facilitate patient access to surgical services. We carried out a retrospective review of our activity to evaluate the impact of this initiative on our service.

**Methods:** We retrospectively analysed the thoracic surgical data prior to the introduction of this programme, pre-covid, 2019 and compared this to 2021.

**Results:** With the introduction of the programme the annual activity increased from 359 to 718 surgical procedures. The volume of urgent referrals managed in the department increased from 132 to 197 patients. The median waiting time for urgent inpatient referral to surgery reduced from five days to two days (mean 6 to 2.7 days). This resulted in a total of 591 national bed days saved and associated cost savings of E502350. In addition, the lung cancer waiting times from referral to surgery reduced from a median of 14 days to nine days (mean 18.2 to 10.3 days).

**Conclusions:** As with most thoracic surgery units our productivity is usually limited by access to beds and theatre. With the help of the national pathway implemented by the health service executive we have been able to achieve a two fold increase in surgical caseload whilst significantly reducing both inpatient and outpatient waiting times for surgery.

## A202 Predictors of short-term mortality in patients with isolated, uncomplicated rib fractures

### Huzaifa Ahmad; Pavlos Papoulidis; Sridhar Rathinam; Apostolos Nakas; Edward Caruana

#### Department of Thoracic Surgery, Glenfield Hospital, Leicester, UK

**Correspondence**: Huzaifa Ahmad

*Journal of Cardiothoracic Surgery 19(1):* A202

**Background:** Traumatic rib fractures are associated with significant early morbidity and mortality. We sought to identify patient and in-hospital predictors of short-term mortality in patients with isolated rib fractures without intrathoracic injury.

**Methods:** Clinical coding data was obtained for all patients admitted to a single UK-based teaching hospital between January 2018 and December 2020 with traumatic rib fractures. Patients with pneumothorax, haemothorax and other bony (including sternal) injury were excluded. Stepwise multiple regression analysis was performed in StataSE v17 for the covariates of age, gender, frailty (defined as a Clinical Frailty Score as five or more), Acute Kidney Injury (AKI), pneumonia (defined clinically) and delirium.

**Results:** 1032 patients were admitted over the study period, of whom 602 (57% male, age 73 ± 17 years) were retained for this analysis. 30-day mortality was 10%. Univariate regression analysis identified age, frailty, AKI, pneumonia and delirium as being associated with 30-day survival. Significance was only retained for AKI [OR 2.53 (95% CI 1.13 to 5.68), p = 0.024] and pneumonia [OR 2.42 (1.16 to 5.01), p = 0.018] in the multivariate model.

**Conclusions:** Our data demonstrates that AKI and pneumonia are independent risk factors for short-term mortality after uncomplicated rib fracture patients.

## A203 The impact of nighttime, weekend and winter admission oon short-term outcomes in rib fracture patients

### Huzaifa Ahmad; Pavlos Papoulidis; Sridhar Rathinam; Apostolos Nakas; Edward Caruana

#### Department of Thoracic Surgery, Glenfield Hospital, Leicester, UK

**Correspondence**: Huzaifa Ahmad

*Journal of Cardiothoracic Surgery 19(1):* A203

**Background:** There is evidence of worse clinical outcomes for patients admitted at night, during the weekend, or in winter across multiple care areas. We sought to investigate these phenomena in the traumatic rib fracture population at a single NHS teaching hospital in the United Kingdom.

**Methods:** Clinical coding data was obtained for all patients admitted to a single UK-based teaching hospital between January 2018 and December 2020 with traumatic rib fractures. Patients with other major bony (including sternal) injury were excluded. Chi square and t tests were applied in StataSE v17 to check for association between [1] length of hospital stay (LoS) and [2] 30-day mortality, and [1] night time (22:00 to 06:00), [2] weekend (Saturday and Sunday) and [3] winter (December 1 to February 28/29) admission.

**Results:** 777 patients (59% male, age 71 ± 18 years) were included in the analysis. Patients admitted during night time (vs day time, p = 0.15 and p = 0.26) and at weekends (vs weekdays, p = 0.75 and p = 0.53) had similar LoS and early-mortality. Patients admitted during the winter months (vs other seasons) had similar LoS (p = 0.80) but higher short-term mortality (13.4% [28/209] vs 7.2% [41/568], p = 0.007).

**Conclusions**: Our data demonstrates important differences in seasonal mortality in our local rib fracture cohort. There is no evidence of a ‘weekend effect’ or variation in outcomes according to time of admission to hospital.

## A204 Does pre-operative symptom duration predict outcomes in empyema?

### Oliver Pumphrey

#### Nottingham City Hospital, Nottingham, UK

**Correspondence**: Oliver Pumphrey

*Journal of Cardiothoracic Surgery 19(1):* A204

**Background:** Para-pneumonic empyema presents with non-specific symptoms. Previous studies have shown a relationship between duration of pre-operative symptoms and outcomes of surgery, including operative approach and length of stay.

**Methods:** We examined our series of patients over a five-year period admitted with empyema from a respiratory source. Pre-operative symptom duration, length of stay, operative approach and complications were recorded.

**Results:** We operated on 285 patients with para-pneumonic empyema between January 2016 to September 2021. The median duration of symptoms was 10 days prior to admission to hospital. There was no correlation between duration of symptoms and length of post-operative stay (r2 = − 0.06). Similarly, we found no correlation between duration of symptoms and operative approach, drain duration or complications at first post-operative review.

**Conclusions:** In our series, there was no relationship we could identify between the pre-operative duration of symptoms and post-operative outcomes. We need to be mindful when trying to predict the clinical course of patients based on the duration of their symptoms.

**Thoracic Oncology**

## A205 Does hyperthermic povidone-iodine lavage increase the apoptotic rate of residual cancer cells in patients with malignant pleural mesothelioma?

### Andrea Bille^1^; Hiral Jhala^2^; Leanne Ashrafian^3^; Leanne Allison^4^; Matthew Russell^4^; Roland Fleck^4^; Daisuke Nonaka^3^

#### ^1^Golden Jubilee National Hospital, Glasgow, UK; ^2^Kings College Hospital, London, UK; ^3^Guy's Hospital; ^4^King's Centre for Ultrastructural Imaging, King's College London

**Correspondence**: Hiral Jhala

*Journal of Cardiothoracic Surgery 19(1):* A205

**Objectives:** Malignant pleural mesothelioma is an incurable, late presenting primary cancer, conferring a survival of 8–14 months. Different intrapleural treatments have been tested as part of a multimodality approach to treat a select group of patients with limited disease, increasing survival. Recently, povidone-iodine has been shown to induce apoptosis in microscopic tumour cells in vitro, with no reported complications. This is the first in vivo study assessing the apoptotic rate caused by intraoperative hyperthermic betadine lavage using routine immunohistochemistry combined with transmission electronic microscopy (TEM).

**Methods:** We included surgically fit patients aged > 18, undergoing minimally invasive video assisted thoracoscopic surgical (VATS) pleural biopsy between December 2016–February 2018, for confirmed or presumed pleural malignancy. Parietal pleural biopsies were obtained at 7.5, 15 and 30 min after hyperthermic betadine lavage, and compared to pre-lavage biopsy samples, for apoptotic changes. Viable tumour samples underwent histological, immunohistochemical and ultrastructural analysis, and TEM for apoptotic features.

**Results:** N = 6. Median age was 76 years. Median overall survival was 26.7 months. There was no statistical impact on survival of laterality of disease (left vs right). Immunohistochemical analysis showed no significant difference in the expressions of apoptotic index markers pre and post betadine treatment. TEM analysis showed no discernible effect on morphological features of apoptosis with betadine treatment (Fig. 1). We identified no side effects post betadine lavage.

**Fig. 1:**
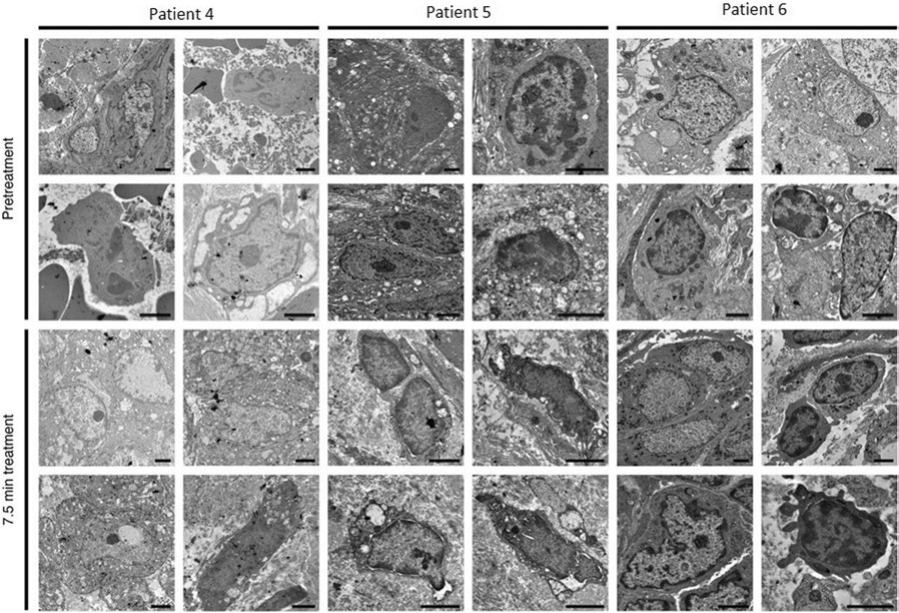
TEM images of pleural biopsy of viable mesothelioma before and after treatment with hyperthermic povidone-iodine solution. 4 images are shown for each condition. The images illustrate the range of morphologies observed, but should not be taken as representative of specific proportions. Bars, 2um. TEM, transmission electron microscopy

**Conclusions:** Though hyperthermic betadine lavage is a safe antiseptic solution with no toxicity when performed intraoperatively, it confers no effect on apoptotic rate or necrosis. It is therefore unlikely that hyperthermic betadine lavage will have an impact on reducing the microscopic residual disease after pleurectomy decortication and enhancing survival.

## A206 Under pressure: clinical impact of injury to the contralateral lung during lung cancer resections and actions to prevent it

### Charlotte Holmes^1^; Benjamin Waterhouse^2^; Jonathan Ferguson^2^; Andrew McDonald^2^

#### ^1^Northern General Hospital, Sheffield, UK; ^2^James Cook University Hospital, Middlesbrough, UK

**Correspondence**: Charlotte Holmes

*Journal of Cardiothoracic Surgery 19(1):* A206

**Objectives:** Contralateral lung injury following lobectomy has a significant impact on mortality and morbidity. Current Enhanced recovery (ERAS) protocols are designed to protect the ipsilateral lung. Our aim was to review incidence and impact of contralateral lung injury following lobectomy and actions to prevent it.

**Methods:** All patients undergoing lung cancer resection from 2015 to 2020 were identified using a thoracic database. The database was used to identify patients with lung injury and radiological imaging was reviewed to confirm contralateral lung changes. Pneumonectomies, patients prior to the 2015 introduced ERAS pathway and patients without contralateral radiological changes were excluded. Medical notes were used to collect data including: ventilation volumes and pressures, length of stay, ITU admissions and mortality.

**Results:** 29 patients were identified. Average age; 65 years. 59% were female. 45% of cases were VATS, 41% open, and 7% robotic. One lung ventilation (OLV) volume and pressures were documented in 21% of patients. Average length of stay was 23 days (range 7–74 days). 48% of patients were escalated to ITU. Of these patients 64% were reintubated. Of those intubated 88% required a tracheostomy and prolonged respiratory wean. 13.8% of patients required inotropic support and 13.8% required renal replacement therapy. 13.8% of patients died as inpatients. Outcomes for patients by side of initial radiological change were worse for those with bilateral changes followed by those with contralateral changes at first presentation in all endpoints.

**Conclusions:** Contralateral lung injury significantly increases morbidity and mortality. The pathophysiology differs to ipsilateral injury; with worse outcome. WHO checklist now updated with OLV protection strategy, reinflation on ipsilateral lung and maximum peak pressure of 30 cmH2O, a “pressure pause” for patients exceeding 30 cmH2O was introduced and early use of oral steroid introduced for acute lung injury.

## A207 Open thoracotomy vs VATS vs RATS approach for segmentectomy: a systematic review and Bayesian network meta-analysis

### Jeevan Francis^1^; Diana Meirinho Domingues^1^; Jeremy Chan^2^

#### ^1^Royal Infirmary of Edinburgh, Edinburgh, UK; ^2^Morriston Hospital, Swansea, UK

**Correspondence**: Diana Meirinho Domingues

*Journal of Cardiothoracic Surgery 19(1):* A207

**Objectives:** Recent trials suggest that more conservative resections such as segmentectomy are non-inferior to more radical approaches. Most segmentectomy surgeries can be safely performed using video-assisted thoracoscopic surgery (VATS). The clinical benefits of robotic-assisted thoracoscopic surgery (RATS) remain unclear. We aimed to perform a systematic review evaluating the outcome of open thoracotomy, VATS, and RATS for segmentectomy.

**Methods:** A systematic database search was conducted of original articles exploring the outcome of open vs VATS vs RATS segmentectomy in PubMed, EMBASE and SCOPUS. Primary and secondary outcomes were 30-day mortality, hospital readmission, air leak, and post-operative pneumonia respectively. Network meta-analysis was performed on the results using a Bayesian framework.

**Results:** Eleven studies were included with a total patient sample size of 7280. There are no significant differences between the three approaches for 30-day mortality, hospital readmission, air leak, and post-operative pneumonia. The VATS approach ranked first in all primary and secondary outcomes, followed by open, and RATS techniques.

**Conclusions**: There are no significant differences between the three approaches. VATS appears to be a safe alternative to open techniques for segmentectomy. The learning curve of RATS remains an important issue prior to wider adoption in the community. Further randomised control studies are required to compare the short and long terms results of VATS and RATS approaches.

## A208 Incidence of acute kidney injury following major lung resection: a single center study

### Benjamin Omoregbee; Hind Elhassan; Tanveer Khan; Salman Arif; Vasileios Tentzeris

#### Castle Hill Hospital/Hull University Teaching Hospital, Hull, UK

**Correspondence**: Hind Elhassan

*Journal of Cardiothoracic Surgery 19(1):* A208

Table 1 initial study patients’ characteristics and results
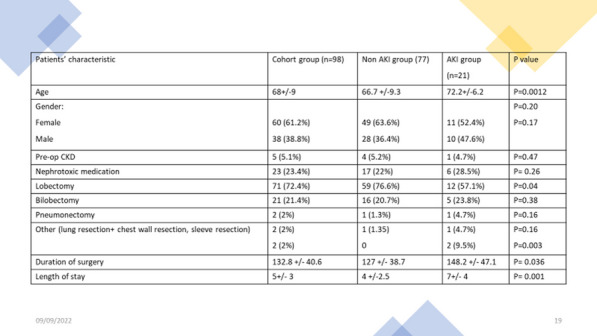


**Objective:**

To determine the incidence of Acute Kidney Injury (AKI) following major lung resection surgery.

**Methods:**

- All patients who underwent major lung resections from December 2021–June 2022 were included in this study.
- Demographics, length of surgery, preoperative and postoperative creatinine levels, and length of hospital stay were collected.
- P-value < 0.05 was deemed significant.
- AKI was staged using the Kidney Disease Improving Global Outcomes Criteria (KDIGO).

**Results:**

- 98 patients were included in the study: 60 (61%) males, 38 (39%) Females.
- Mean age was 68 years (± 9). No AKI: 68 ± 9, AKI: 72.2 ± 6.2 (p = 0.012).
- Case distribution: Lobectomy 71 (72.4%), Bilobectomy 21 (21.4%), Pneumonectomy 2 (2%), Other (lung resection + chest wall resection) 2 (2%), sleeve resection 2 (2%).
- 42.8% (9) of patients were 1st, 28.6% (6) were 2nd, 9.5% (2) were 3rd, 9.5% (2) were 4th, 4.8% (1) were 5th, 4.8% (1) were 6th on the Operating list.
- Incidence of AKI was 21.4% (21) within 48 h of surgery.
- KDIJO staging: AKI I: 16(76%), AKI II: 4 (19%), AKI III: 1 (5%).

**Conclusions:**

- In this study, 1 in 5 patients developed postoperative AKI. Most of these were Stage 1 AKI.
- AKI occurred more in patients who were first and second on the operating list who are likely to have fasted longest.
- Patients who developed AKI, spent 2 days longer in the hospital compared to patients without AKI and this was statistically significant.

## A209 Incidence of oncogenic driver mutations in surgically resected non small cell lung cancer

### Bejoy Philip; Mauin Uddin; Michael Shacklcoth

#### Liverpool Heart and Chest Hospital, Liverpool, UK

**Correspondence**: Mauin Uddin

*Journal of Cardiothoracic Surgery 19(1):* A209

**Background:** Epidermal growth factor receptor (EGFR) mutation is more common in the Asian population with a prevalence of 38% compared to 14% in European patients. Also, Kirsten RAt Sarcoma (KRAS) mutations occur in 20–40% of lung adenocarcinomas. Higher in Western vs Asian populations (26% vs. 11%).

Recently tyrosine kinase inhibitors (TKIs) are licensed for treating EGFR mutation positive lung cancer patients. Sotorasib is a new drug active against KRAS mutations.

**Objectives:** To identify the incidence of EGFR and KRAS mutation patients in our surgical cohort of UK patients.

Compare the occurrence of EGFR mutation in our female patients compared to the occurrence mentioned in literature.

Analyse the number of patients with EGFR and KRAS mutations who died during the study period.

**Patient selection:** All patients who underwent surgical resection for lung cancer and consented for genetic mutation study from July 2016 to December 2018.

**Methods:** Data gathered from online database of patients whose genetic mutation studies were done. Data was collected data of 279 patients, who underwent surgical resection of lung cancer in the study period.

Some patients were excluded as their samples failed to pass quality control, inadequate sample or there was no malignancy seen in the specimen. Finally, data of 217 patients were suitable for this study.

**Results:** KRAS mutation was seen in 34 patients, which is 28.8%.

EGFR mutation was seen in 12 cases, which is 10.1%.

Five patients with adenocarcinoma and EGFR mutation were females, which is 41%.

KRAS mutation was seen in 34 patients, which is 28.8%.

EGFR mutation was seen in 12 cases, which is 10.1%.

Five patients with adenocarcinoma and EGFR mutation were females, which is 41%.

**Conclusions:** EGFR mutation was only 10.1% as compared to 14.1% expected in a European population.

KRAS mutation was 28.8% as expected for the population.

Out of 12 patients with adenocarcinoma who had EGFR mutation, 5 were female patients, 41%.

Four EGFR mutation patients were non smokers.

Figure shows the various types of lung cancers seen in the patient cohort analysed and their percentages.
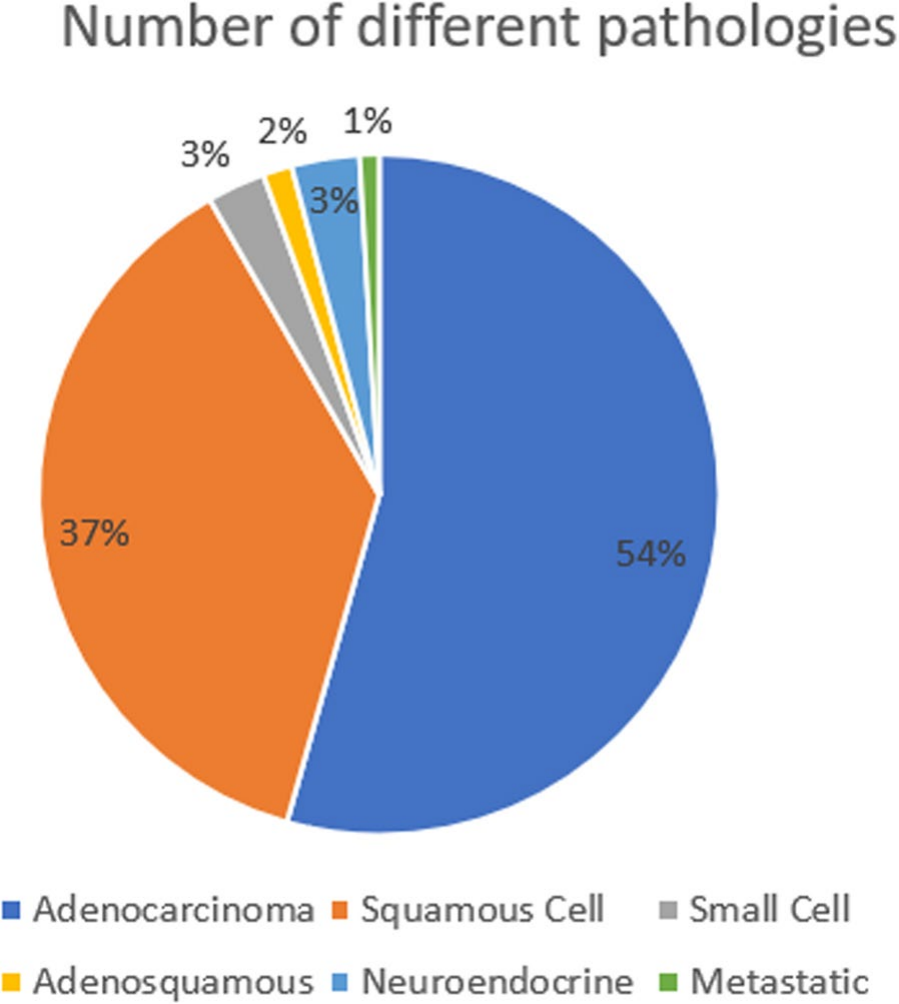


## A210 Intraoperative use of A biodegradable polymeric sealant reduces the incidence of air leak and duration of chest drains after anatomic lung resection

### Savvas Lampridis^1^; Iulia Bujoreanu^2^; Andrea Bille^1^

#### ^1^Guy's Hospital, London, UK; ^2^St Thomas' Hospital, London, UK

**Correspondence**: Andrea Bille

*Journal of Cardiothoracic Surgery 19(1):* A210

**Objectives:** To evaluate the safety and effectiveness of a biodegradable polymeric sealant (Progel, BD, Franklin Lakes, NJ, USA) for intraoperative closure of air leaks that may occur during anatomic lung resection.

**Methods:** We conducted a retrospective comparative cohort study of consecutive adult patients who underwent lobectomy or segmentectomy for lung cancer between January and September 2022. Patients were divided in two groups according to whether Progel was used or not. In general, Progel was applied to the remaining lung, including fissures, staple lines, and raw pulmonary surface where conventional methods of air-leak closure were ineffective or impractical. Postoperative air leaks were recorded with a digital chest drainage system (Thopaz+, Medela, Baar, Switzerland).

**Results:** In total, 75 patients were included in the study: 36 in the sealant group and 39 in the control group. There were no significant differences between the groups in terms of demographic characteristics, comorbidities, cancer stage, neoadjuvant therapy, completeness of lung fissures, or surgical approach and procedure. Absence of air leak on the first postoperative day was recorded for 26 (72.2%) patients in the sealant group compared to 14 (35.9%) patients in the control group (*P* = 0.002). The median duration of chest drains was significantly shorter in the sealant group at 2.5 days (IQR, 2–5 days) compared to four days (IQR, 3–7 days) in the control group (*P* = 0.005). The median length of hospital stay was five days (IQR, 2–7 days) in the sealant group versus six days (IQR, 4–9 days) in the control group (*P* = 0.029). There were no significant differences in morbidity, mortality, or readmissions between the groups. Progel was not associated with any adverse events.

**Conclusions:** Intraoperative use of Progel significantly decreased the incidence and duration of postoperative air leak following anatomic lung resection, which led to a significant reduction in the length of hospitalisation.

## A211 An absorbable haemostatic powder with polysaccharide particles is safe and effective for control of bleeding during lung resection and lymphadenectomy

### Savvas Lampridis^1^; Iulia Bujoreanu^2^; Andrea Bille^1^

#### ^1^Guy's Hospital, London, UK; ^2^St Thomas' Hospital, London, UK

**Correspondence**: Savvas Lampridis

*Journal of Cardiothoracic Surgery 19(1):* A211

**Objectives:** To evaluate the safety and effectiveness of a novel absorbable haemostatic powder that consists of microporous polysaccharide particles (Arista, BD, Franklin Lakes, NJ, USA), for control of bleeding that may occur when performing anatomic lung resection with complete lymphadenectomy.

**Methods:** We conducted a retrospective comparative cohort study of consecutive adult patients with lung cancer who underwent lobectomy or segmentectomy with complete lymphadenectomy. Cases were included if a haemostatic agent was used to control arterial and/or venous bleeding when conventional methods of control were ineffective or impractical. The haemostats used were either Arista or an absorbable layered haemostat prepared by oxidation of regenerated cellulose (Surgicel Fibrillar, Ethicon, Raritan, NJ, USA).

**Results:** The study included 120 patients in the Arista group and 104 in the control group. There were no significant differences between the groups in terms of demographics, comorbidities, cancer stage, or surgical approach and procedure. No patients in the Arista group underwent reoperation for bleeding compared to 2 patients in the control group (*P* = 0.214). There were no differences in transfusion of blood products or median estimated blood loss between the groups (70 [IQR, 65–100] ml in the Arista group vs 70 [IQR, 60–105] ml in the control group; *P* = 0.219). Use of Arista was associated with significantly less median drain output in the first 24 h after surgery (175 [IQR, 100–265] ml vs 220 [IQR, 100–375] ml; *P* = 0.011). The median duration of chest drains was 2 (IQR, 1–4) days in the Arista group compared to 3 (IQR, 2–4) days in the control group (*P* = 0.041). There were no significant differences in air-leak duration, morbidity, mortality, or readmissions between the groups. Arista was not associated with any adverse events.

**Conclusions:** Arista was safe and effective for control of bleeding when performing anatomic lung resection with complete lymphadenectomy.

## A212 An evaluation of patient satisfaction with the introduction of electromagnetic navigational bronchoscopy into clinical practice

### Yasmin Sheikey^1^; Juliet Thomas^1^; Laura White^1^; Richard Finley^2^; Connor Byrne^1^; Jackie Chandler^2^; Martin Chamberlain^1^; Edwin Woo^1^; Lukacs Veres^1^; Aiman Alzetani^1^

#### ^1^University Hospital Southampton, Southampton, UK; ^2^Wessex Academic Health Science Network, Southampton, UK

**Correspondence**: Yasmin Sheikey

*Journal of Cardiothoracic Surgery 19(1):* A212

**Objectives:** ENB is a device that generates a 3D geometric approximation of the tracheobronchial tree of the patients' airways through the use of CT scans to enable clinicians to obtain samples from lung lesions that are not accessible through traditional bronchoscopy or CT guided biopsies. This service has been introduced recently at a thoracic surgery regional centre. We have surveyed the patients for their experience and satisfaction with this service.

**Method:** Data was collected from all the patients who were referred for ENB using Somerset and other hospital databases, this included demographics, referral pathway, pathological findings, any complications from procedure and length of hospital stay. Patients were asked post procedure on their experience and overall satisfaction.

**Results:** In total 33 patients underwent ENB, of these 18 were female and 15 were male with the ages ranging from 54 to 88 years. The first patient was put on a cancer pathway in 2019 and the most recent was in 2022. There was no complications and all patients were discharged on the same day. In total 12 patients responded to the survey which was conducted between October 2022 to November 2022. All results were anonymised and have been presented in the image below:

The initial pathological diagnosis for the patients has been listed below:

Carcinoid tumour: 5

Malignant neoplasm: 10

Adenocarcinoma: 6

Non-malignant: 12

**Conclusions:** Overall ENB is a safe procedure with a high diagnostic yield, minimal complications and a short hospital stay. The majority of patients were satisfied with the service and the level of information and care provided by clinical staff. It was difficult to make a comparison between ENB and CT/Bronchoscopy biopsy with this group of patients. ENB is a useful diagnostic tool and should be considered within lung cancer MDT when more traditional methods are not suitable.

## A213 Wedge resection for T1 vs T2 non-small cell primary lung cancer outcomes

### Andrew Jones; Thomas Combellack; Jeremy Chan

#### University Hospital Wales, Cardiff, UK

**Correspondence**: Andrew Jones

*Journal of Cardiothoracic Surgery 19(1):* A213

Recent evidence suggests that wedge resections should be considered for early-stage non-small cell lung cancer. We aim to compare survival in patients undergoing non-anatomical wedge resection for T1 and T2 primary non-small cell lung cancer. We have conducted a retrospective, single centre study, using PATS data and online patient records to identify our patient cohort. We identified 30 consecutive patients (15 T1 and 15 T2) who underwent intended wedge resection for primary lung cancer between December 2014 and April 2019. Exclusion criteria included small cell carcinoma, clinical nodal disease and all those who underwent frozen section and completion lobectomy. Data were analysed using a Kaplan–Meier curve.

After appropriate censorship, the data shows that patients under-going wedge resection for T1 had a statistically better overall survival than for T2 (P = 0.013) however the analysis also revealed no statistical difference between the 2 groups for disease-free survival (P = 0.371) (see Table 1).

It is unclear why our cohort of T2 patients had a higher mortality with equivocal recurrence rates. A large proportion of the T2 group had tumours under 3 cm but had a form of pleural invasion, upstaging them to T2. This could suggest that pleural invasion is a significant prognostic indicator in this patient cohort in terms of survival however this did not affect disease-free survival/recurrence.
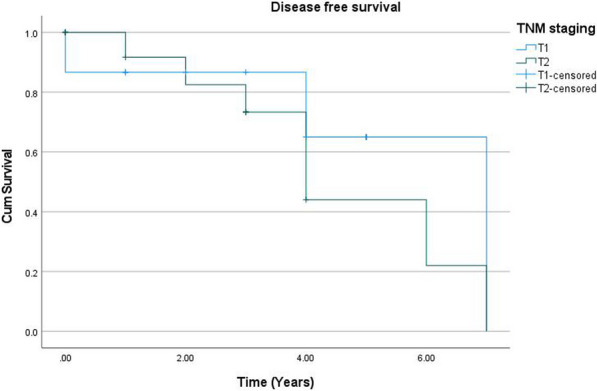


## A214 Postoperative outcomes based on surgical access for stage I/II non-small cell lung cancer resection

### Qingzi Guo; Andrew Chrisp; Amber Ahmed-Issap; Rana Mehdi; Avishek Samaddar; Kajan Mahendran; Lakshmi Srinivasan; Shilajit Ghosh; Udo Abah

#### University Hospitals of North Midlands NHS Foundation Trust, Stoke-On-Trent, UK

**Correspondence**: Qingzi Guo; Andrew Chrisp

*Journal of Cardiothoracic Surgery 19(1):* A214

**Objectives:** To compare postoperative outcomes and short-term pain levels following anatomical lung resections for stage I or II NSCLC based on surgical access.

**Methods:** All patients who underwent segmentectomy or lobectomy for stage I or II NSCLC between July 2018 and June 2021 were included. Exclusion criteria are: complex resections (e.g. sleeve/bi-lobectomy), ICU stay > 1 day, and incomplete record of pain scores. Patients were divided into five cohorts based upon operative approach: uniportal vats, multiportal vats, muscle-sparing thoracotomy, mini-thoracotomy, and classical posterolateral thoracotomy (division of latisimus dorsi). Postoperative outcomes including complication rates, length of stay, and highest reported pain score on day 1&2 postoperatively were analysed.

**Results:** 281 patients were included. The uniportal cohort had the highest proportion of pain-free patients on day 1&2 postoperatively (37%, 56% respectively), and the lowest mean pain scores (1.84, 1.35 respectively). Highest pain scores were recorded in the mini-thoracotomy cohort (3.2, 2.19), with intermediate findings in other cohorts (multiportal: 2.6, 2.14; muscle-sparing: 2.7. 2.12; posterolateral: 2.74, 1.84), p value < 0.05. Overall complication rate and mean length of stay was also found lowest in the uniportal cohort.

**Conclusions:** Uniportal vats approach is linked to reduced pain scores immediately following surgery and a lower rate of postoperative complications, warranting a further prospective study.
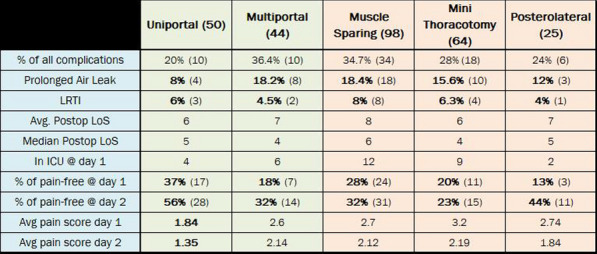


## A215 Passing on the knowledge: Are the old crafts being lost in the new wave of technology and what does this mean for trainees?

### Ben Shanahan; Cristina Viola; David Waller

#### St Bartholomew's Hospital, London, UK

**Correspondence**: Ben Shanahan

*Journal of Cardiothoracic Surgery 19(1):* A215

**Background:** Thoracic surgical training must remain in step with new developments but a core knowledge of traditional procedures may be lost. An overview of thoracic practice may inform training requirements.

**Methods:** We analysed the case mix in prospectively collected data of a 25-year consultant practice, including operations performed by over 40 trainees.

**Results:** Open procedures have dropped from 94% (n = 105) in 1997 to 24% (n = 12) by 2022 (p < 0.01) whilst VATS increased from 6% (n = 6) to 54% (n = 50) by 2016 (p < 0.01). RATS now accounts for 72% of cases, and has facilitated a sharp increase in segmentectomy, from 15% (n = 5) to 50% (n = 25) between 2017 and 2022 (p < 0.01). Although the number of extended resections (pneumonectomy, sleeve, chest wall resection) have significantly declined they still account for 20% (n = 10) of cases in 2022 compared to 37% (n = 41) of cases in 1997 (p < 0.01).

**Conclusions:** Lung cancer resection is now performed at an earlier stage taking less lung tissue via smaller incisions. The focus on minimally invasive surgery must not result in unfamiliarity with traditional techniques as the demand for them may rise again with the advent of surgery post induction treatment. Modern thoracic training should include competency in these procedures possibly requiring trainee-directed placements (Fig. 1).

**Fig. 1:**
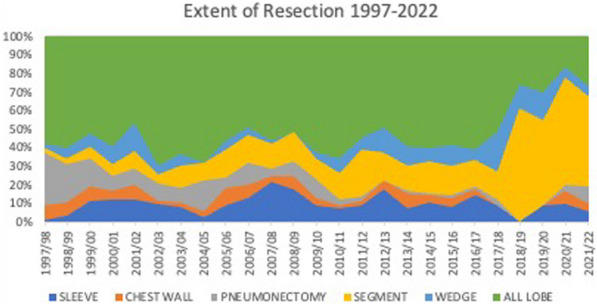
Extent of resection 1997–2022

## A216 VE/VCO2 slope in CPET is independently associated with postoperative outcomes of lung resections

### Amr Rushwan; Polyvios Drosos; Javeria Tariq; Nilanjan Chaudhuri; Joshil Lodhia; Richard Milton; Konstantinos Papagiannopoulos; Peter Tcherveniakov; Elaine Teh; Alessandro Brunelli

#### St James's University Hospital – Leeds Teaching Hospitals NHS Trust, Leeds, UK

**Correspondence**: Amr Rushwan

*Journal of Cardiothoracic Surgery 19(1):* A216

**Objectives:** To assess the association between VE/VCO2 slope and postoperative mortality of lung resections for cancer.

**Methods:** All patients who were submitted to lung resection for cancer from 2014 until 2022 and had a complete CPET with VE/VCO2 slope measurement were included. CPET was performed in patients with reduced pulmonary function or cardiovascular comorbidity. A logistic regression analysis was performed to assess the independent association between VE/VCO2 slope > 40 and 90-day mortality.

**Results:** 552 of 2303 patients, who underwent lung resection for cancer, had a CPET with VE/VCO2 slope. Of those, 374 were lobectomies, 81 segmentectomies, 55 pneumonectomies and 42 wedge resections. The total cardiopulmonary morbidity was 32%, in-hospital/30-day mortality 6.9% and 90-day mortality 8.9%. 138 patients (25%) had a slope > 40. These patients were older (72% vs. 69.6%, p = 0.012), had lower DLCO (57.3% vs. 68%, p < 0.001), lower BMI (25.4% vs. 27.0%, p = 0.001) and lower VO2max (14.9% vs. 17.0%, p < 0.001) than those with a slope < 40. They had more frequently IHD (17% vs. 10%, p = 0.028). No differences were found in sex distribution, FEV1 values, or incidence of CVD, CKD and DM between the groups. The cardiopulmonary morbidity amongst patients with a slope > 40 was 40% vs. 29% in those with lower slope (p = 0.019). The incidence of respiratory complications was 31.4% vs. 23.6% (p = 0.07). The in-hospital/30-day mortality was 12% vs. 5.1% (p = 0.004) and the 90-day mortality was 15% vs. 6.7% (p = 0.002). The 90-day mortality of elderly patients with slope > 40 was 21% vs. 7.8% (p = 0.001). The 90-day mortality in the high slope group was 15.5% in lobectomies, 33.3% in pneumonectomies, 13.3% in segmentectomies and 6.2% in wedge resections (p = 0.38). A logistic regression analysis showed that VE/VCO2 slope retained a significant association with 90-day mortality after adjusting for variables.

**Conclusions:** VE/VCO2 slope is strongly associated with morbidity and mortality.

## A217 Patterns of recurrence in resected N2 non-small cell lung cancer: Where to focus follow-up and therapy

### Kieran McManus; Jamal Khan

#### Royal Victoria Hospital, Belfast, UK

**Correspondence**: Kieran McManus

*Journal of Cardiothoracic Surgery 19(1):* A217

**Objectives:** To determine the commonest sites of recurrence after anatomical resection for NSCLC with N2 disease to guide follow-up and therapy.

**Methods:** Retrospective analysis of a regional thoracic database of patients with N2 disease following anatomical resection for NSCLC between 2017 and 2021. Patients with previous lung cancer therapy were excluded.

**Results:** Of 609 anatomical resections, 59 (9.7%) had N2 disease (27 preoperatively staged as N0, 9 staged N1 and 23 known N2). 25 had pre-resection mediastinoscopy or EBUS and 34 PET staging only.

Overall, five-year survival was 35.9% and was better in those whose N2 disease was found incidentally at surgery (preop N0 50.8%, known N2 12.9%).

33 patients had adjuvant chemotherapy, 22 completing a full course of four cycles. The commonest reasons for failing to complete therapy were surgical complications or postoperative death 4, unfit 5, patient preference 5, COVID 6, early relapse 5, toxicity 5. 23 proceeded to second line chemotherapy or immunotherapy following recurrence with median survival 32.4 months vs 15 months without SACT.

44 (74.6%) patients had recurrence. Of these, the site of first recurrence was brain 25%, mediastinum 22.7%, lung 11.4%, bone 9.1%, pleura 4.5% and abdomen 2.3%. Eight (18.2%) presented with multiple sites of recurrence (3 intrathoracic, 5 extrathoracic). Further sites of recurrence were subsequently found in the mediastinum 21, lung 17, brain 12, bone 11 abdomen 9, and subcutaneous 1.

Single site metastasis was found in the mediastinum in 10 and brain in 11. But 4 at each of these sites subsequently developed disseminated disease.

**Conclusions:** The high incidence of mediastinal and brain recurrence suggests that prophylactic local therapies ought to improve survival. That this has not been supported by randomised controlled trials indicates that disease is likely to be disseminated at the time of surgery and it can be difficult to deliver the required multimodality therapies following surgery.

## A218 Project murray a trial to study the effectiveness of smoking cessation in the surgical pathway before major lung surgery: the journey so far

### Salma Kadiri^1^; Christer Lacson^2^; Helen Shackleford^2^; Aya Osman^2^; Hazem Fallouh^2^; Ehab Bishay^2^; Maninder Kalkat^2^; Ashvini Menon^2^; Vanessa Rogers^2^; Babu Naidu^1^

#### ^1^University of Birmingham / University Hospitals Birmingham (UHB), Birmingham, UK; ^2^QE Hospital, Birmingham, UK

**Correspondence**: Salma Kadiri

*Journal of Cardiothoracic Surgery 19(1):* A218

**Objectives:** 15% patients undergoing major thoracic surgery develop PPC. Smoking is the biggest risk factor. One in four patients continue to smoke before surgery. NICE recommends all patients stop, however. Uptake is poor in the community (< 5%). There are no specific applications to help patients stop smoking before thoracic surgery. The aim is to assess various aspects of the trial design and management at five hospitals.

**Methods:** Patients will be recruited into either intervention or usual care arm. Three elements to the intervention arm are behavioural Support, Pharmacotherapy and a web-based application. Patients completed questionnaires to assess NRT usage, nicotine dependency, mood, physical symptoms health resource usage and QOL. Some patients and staff have been interviewed to assess feasibility of trial and app.

**Results:** Only 34 patients recruited due to COVID, all patients recruited on average 16 days after being seen in initial surgery clinic. Full data will be available at the end of recruitment date early 2023. Interviews have been analysed using thematic analysis. Training of staff has received positive feedback. Extra support has been appreciated by patients.

**Conclusions:** The process measures, trial pathways and questionnaires have been satisfactory. With ongoing recruitment, the target of 120 patients can be achieved.

## A219 Is thoracotomy in danger of becoming a lost skill?

### Daniel Jones; Miles Buller; Joshil Lodhia; Elaine Teh; Richard Milton; Nilanjan Chaudhuri; Kostas Papagiannopoulos; Alex Brunelli; Peter Tcherveniakov

#### St James's University Hospital – Leeds Teaching Hospitals NHS Trust, Leeds, UK

**Correspondence**: Daniel Jones

*Journal of Cardiothoracic Surgery 19(1):* A219

**Objectives:** In this study we address whether the rise in minimally invasive procedures may render the thoracotomy a lost skill. Minimally invasive procedures are associated with lower complication rates, shorter hospital stays and are less expensive than their open counterparts [1–3]. Consequently, ‘keyhole’ techniques have become the predominant method of surgical access for a number of procedures across specialities [3,4]. Trainee exposure to thoracotomy has anecdotally decreased. We aim to analyse the impact of minimally invasive techniques on thoracotomy experience in trainees.

**Methods and Results:** Between 2017 and 2021, 1,294 patients underwent elective lung resection in a UK teaching hospital. 288 (22.3%) procedures were open, and 79 (27%) of these were completed between six trainees. The mean number of thoracotomies performed by a trainee in this 5-year period was 2.6 per year. In comparison, between 2012 and 2016 a thoracic trainee now recently appointed as a consultant, completed 128 thoracotomies, a mean of 25.6 per year. Levene’s test for variance was p = 0.064, and independent samples T-test was p = 0.038 (p < 0.05).

**Conclusions:** This highlights a deficit in thoracotomy experience in current thoracic surgery trainees. Thoracotomy is an essential skill; adhesions, intra-operative bleeding and increased tumour size are known risk factors for VATS-open conversion [5]. Robotic access is associated with low conversion rates. [6,7]. However, in an emergency a trainee must be competent in gaining open access whilst the consultant scrubs-in.

Outside of elective work, though data regarding incidence is poor, resuscitative thoracotomy is an important part of ATLS management [8]. Steps to improve thoracotomy training opportunities may include simulation and cadaveric based training. A 2019 study by Melmer et. al. suggested a competency based system for ensuring trainees are proficient in open cholecystectomy and appendectomy, a similar approach could be used here [9].

## A220 Lymph node dissection in lung cancer surgery: a comparison between robot-assisted vs video-assisted vs open approach

### Patrick Deniz Hurley; Fabbri Giulia; Savvas Lampridis; Andrea Bille

#### Guys and St Thomas NHS Trust, London, UK

**Correspondence**: Patrick Deniz Hurley

*Journal of Cardiothoracic Surgery 19(1):* A220

**Objectives:** TNM staging is the most important prognosticator for non-small cell lung cancer (NSCLC) patients. Staging has significant implications for the treatment modality of these patients. Lymph node dissection in robot-assisted thoracoscopic (RATS) surgery remains an area of ongoing evaluation. In this study, we aim to compare lymph node dissection in RATS, video-assisted thoracoscopic (VATS) and open lung resection.

**Methods:** We retrospectively compiled a database of 844 patients from 31/7/2015 to 8/7/2022 who underwent either a wedge resection, segmentectomy, lobectomy or pneumonectomy. We analysed the database according to lymph node dissection. The database was divided into RATS (n = 375), VATS (n = 342) and open (n = 127) procedures.

**Results:** The mean number of lymph nodes harvested overall with RATS was 6.1 ± 1.5 nodes, with VATS approach it was 5.53 ± 1.8 nodes, and with open procedures, it was 6.04 ± 1.6 nodes. The mean number of N1 stations harvested was 2.66 ± 0.8 with RATS, 2.36 ± 0.9 with VATS and 2.4 ± 0.7 with open technique. RATS approach showed statistically more lymph node dissection compared to VATS (p = 0.002) and similar level of dissection with open approach (p = 0.541). Open approach also showed more lymph node dissection when compared to VATS approach (p = 0.004).

Out of the 375 RATS procedures, 26 (6.4%) patients undergoing a RATS procedure were upstaged from N0/N1 staging to N2. N0/N1 to N2 upstaging was reported in 28 of 342 (8.2%) patients undergoing a VATS procedure and 13 of 127 (9.5%) undergoing an open procedure. The majority of upstaging was seen in N0 to N2 disease 19 of 375 (5%) for RATS and 23 of 342 6.7% for VATS with open procedure showing more frequent N1 to N2 upstaging, 7 of 12 (5.5%).

**Conclusions:** We conclude that in RATS procedures there is a higher rate of lymph node dissection compared to VATS procedures. There is similar N1/N2 lymph node harvesting with open procedures and RATS procedures.

## A221 Anatomical lung resection in patients without a pre-operative tissue diagnosis and an evaluation of the utility of frozen section

### Patrick Deniz Hurley^1^; Mylene Sait-Rosenberg^2^; Luke Holland^1^; Smith Alex^1^; Muslim Mustaev^1^; John Pilling^1^

#### ^1^Guys and St Thomas NHS Trust, London, UK; ^2^King’s College London, London, UK

**Correspondence**: Patrick Deniz Hurley

*Journal of Cardiothoracic Surgery 19(1):* A221

**Objectives:** In patients with a lung nodule that is concerning for malignancy, tissue diagnosis can be key to deciding if a patient warrants anatomical lung resection. When a pre-operative tissue diagnosis has not been obtained, an intraoperative frozen section can sometimes be used to determine preliminary histopathology. Patients without favourable anatomy may have to undergo lung resection for both diagnosis and treatment. It is feasible in our current era of modern medicine that frozen section analysis is of lesser utility, and this study aims to investigate the contemporary value of frozen section in patients without pre-operative tissue diagnosis.

**Methods:** Two cohorts of patients undergoing lung resection at a single centre were identified retrospectively. The first underwent pulmonary resection in the absence of preoperative or intraoperative (FS) tissue diagnosis, and identified how often they had cancer on final histological workup. The second cohort underwent an intraoperative FS to obtain a preliminary diagnosis, and we identified how often lung resection was abandoned due to the result.

**Results:** In our first cohort from January 2015 to October 2019, 71 patients without a pre-operative diagnosis or frozen section underwent pulmonary lobectomy as a combined diagnostic and therapeutic procedure. 68 (95.7%) were found to have malignant disease, most commonly NSCLC (n = 58), and the remaining 3 (4.3%) benign diagnoses were granulomatous inflammation.

In our second cohort from January 2019 to June 2022, 50 patients had a frozen section, of whom 6 (12%) did not require lobectomy as a result of the preliminary histological report.

**Conclusions:** In patients who cannot undergo pre- or intraoperative diagnosis, proceeding to anatomical lung resection is appropriate as a large majority will have a cancer diagnosed on formal histological analysis. However, our study supports the continued use of frozen section, as it will prevent 12% of patients undergoing non-prognostic resections.

## A222 Use of an innovative platform for post-surgery monitoring of thoracic surgical patients following hospital discharge

### Aaina Mittal; Matthew Fung; Nadir Syed; Fiona Devine; Rogelio Ferreol; Safia Malik; Carol Tan

#### St Georges Hospital, London, UK

**Correspondence**: Aaina Mittal

*Journal of Cardiothoracic Surgery 19(1):* A222

**Objectives:** Patient reported outcomes play a vital role in monitoring patients undergoing thoracic surgery and evaluating their care. Care4Today Monitor (C4TM) is an innovative platform based on the principle that there is a "micromoment" of 1.6 s for questions to be most reliably answered. It is a mobile application which enables patients to communicate with healthcare professionals (HCPs) by capturing patient feedback digitally using their smartphone after hospital discharge. We report results from the first UK experience of C4TM in thoracic surgery.

**Methods:** All patients undergoing thoracic surgery at a specialist tertiary referral centre were eligible for inclusion. They were provided with information on the purpose, installation and usage of C4TM. Data collected from the app was evaluated.

**Results:** Over a 7-month period 66 patients installed C4TM, and 47 have completed the 4-week monitoring period. 2322 (71.9%) out of a total of 3228 questions asked have been answered. There were 540/2322 (23%) notifications generated requiring HCP attention with most occurring within the first week. The most common notifications generated (summarised in table) were related to pain (n = 232), wound problems (n = 139) and breathlessness (n = 57). 19% reported requiring readmission/medical attention due to an unforeseen event since their discharge postoperatively. The overall score for how much patients felt this application helped with follow-up of their surgery and recovery was 6.8/10.

**Conclusions:** C4TM is an effective adjunct for connecting thoracic surgical patients with HCPs after hospital discharge, with positive user experience reviews as well as packaging and providing valuable information in a convenient way for the HCP to make clinical decisions.

**Table:** 

Commonest alerts	Total number	Level of notification
Patient has abdominal or shoulder pain	99	Moderate
Patient has moderate pain (VAS 6 to 8)	70	High
Patient has chest pain	49	Moderate
Patient has severe pain (VAS 9 to 10)	14	Very high
Wound problem (overall)	139	
Patient has gaping wound	4	High
Breathlessness (overall)	57	
Patient feels shorter of breath than normal	6	Very high

## A223 Video-assisted thoracoscopic surgery (VATS) and multimodal anaesthetic approach in non-intubated patients

### Maria Rita Maccaroni; Ahmed Shazly; Youssef Abouelela

#### Essex Cardiothoracic Centre, Basildon, Basildon University Hospital, UK

**Correspondence**: Youssef Abouelela

*Journal of Cardiothoracic Surgery 19(1):* A223

**Background:** Video-assisted thoracoscopic surgery (VATS) has revolutionised the approach towards minimally invasive thoracic surgery. It is equally important to adopt an anaesthetic approach that further reduce invasiveness. Currently, there is no correlating clear anaesthetic guideline and therefore these specific recommendations are based on a combination of literature sources and clinical practice. Fundamentally this approach allows for non-intubated spontaneous breathing via a laryngeal mask (LMA/I-Gel) with a multimodal analgesic pain management.

**Recommendations:** These recommendations are for minimally invasive procedures such as pleural biopsy, indwelling pleural catheter insertion, bullectomy or small wedge resection. This guidance is for short, selected procedures discussed and agreed with the surgical team. Using a LMA or I-Gel instead of double lumen endotracheal tube or bronchial blocker, has many advantages including reduced airway trauma from direct laryngoscopy and requirement of no muscle paralysis. The use of total intravenous anaesthesia (TIVA) allows for target-controlled infusions with short-acting drugs (such as remifentanil and propofol) and the depth of anaesthesia is monitored with continuous bispectral index (BIS) monitoring. The aim is to reduce perioperative opioid consumption and instead use a more novel approach with single paravertebral block or erector spinae block pre-emptively. The analgesic approach is completed with intravenous administration of Paracetamol 1 g before knife to skin, Ketorolac 30 mg and Magnesium sulphate 2 g before the end of surgery for best post-operative outcomes.

**Conclusions:** Implementation of a multimodal approach in minimally invasive thoracic procedures is now the gold standard. These recommendations are associated with improved clinical outcome, reduced pain and complications as well as overall enhance recovery with shorter hospital stay and better patient satisfaction.

## A224 The impact of COVID-19 on lung resection training

### Mohamed Sherif; Joshil Lodhia; Richard Milton; Kostas Papagiannopoulos; Alex Brunelli; Nilanjan Chaudhuri; Elaine Teh; Peter Tcherveniakov

#### St James Hospital, Leeds, UK

**Correspondence**: Mohamed Sherif

*Journal of Cardiothoracic Surgery 19(1):* A224

**Objectives:** This study aims to evaluate the impact of COVID on major lung resection training in our thoracic surgery unit.

**Method:** A retrospective study of major lung resections was performed in our thoracic centre between April 2018 to April 2022. Data on the operation performed and the primary operator was collected from the electronic patient system. Data were analysed using IBM SPSS software version 27. Pre-COVID time was identified in this study from April 2018 to April 2020, and COVID time was from April 2020 to 2022.

**Results:** 771 lung resection cases were performed between April 2018 to April 2022 at our centre. The analysis of the cases is summarised in Table 1.

**Table 1:** Annual case volume summary and analysis by grade of surgeon

Year	Cases performed by consultants	Cases performed by registrars	Total cases	% of cases performed by registrars
2018–2019	207	75	282	27%
2019–2020	191	102	293	35%
2020–2021	165	68	233	29%
2021–2022	208	96	304	32%

The average number of lung resection cases performed by trainees before COVID-19 time was similar to post-COVID time, with 89 and 82 respectively (p > 0.05). On average, trainees in our unit performed around a third of cases pre and during COVID time. No significant reduction in lung resections was performed in our centre pre and post-COVID time (P > 0.05).

**Conclusions:** COVID-19 had no significant impact on lung resection training in our centre. The unit maintained a similar average number of lung resections, and trainees performed around a third of cases despite the pressure of COVID-19.

We believe lung cancer surgery had fewer chances of cancellation, and our trainees were not deployed to other departments during COVID-19. Hence our unit maintained a high volume of patients and training opportunities. Although these results are encouraging, the full impact of the pandemic on training is unclear. Our results suggest that training in lung resection was preserved by prioritising lung cancer work. However, this was achieved by sacrificing exposure to several benign conditions.

## A225 Single centre retrospective analysis of lung cancer recurrence following surgical resection

### Rana Mehdi; Amber Ahmed-Issap; Qingzi Guo; Avishek Samaddar; Lakshmi Srinivasan; Kajan Mahendran; Shilajit Ghosh; Udo Abah

#### Royal Stoke University Hospital, Stoke, UK

**Correspondence**: Rana Mehdi

*Journal of Cardiothoracic Surgery 19(1):* A225

Surgical resection offers the best chance of long-term asurvival in patients with early stage cancer; approximately 30–55% of patients with resectable non-small cell lung cancer recur within 5 years. Pathological indicators for recurrence are not well understood; factors including tumour size, rate of tumour growth and pathological staging have demonstrated association with recurrence rates. We performed a retrospective analysis of a single centre’s experience to determine clinicopathological markers predictive of local and/or distant recurrence.

We analysed all consecutive lung cancer resections at our institution from 01/01/2012 to 31/10/2019. Patients with synchronous primaries or metastatic disease at presentation were excluded. Univariate analysis was conducted to identify predictors of recurrence.

1244 patients were identified. 451(36.3%) suffered from recurrence. 263(58.3%) had locoregional recurrence, 78(17.3%) distant recurrence and 110(24.4%) were found to have both locoregional and distant recurrence. Recurrence rates per cell type were; adenocarcinoma 39.9%, squamous cell carcinoma 34.4%, carcinoid tumours 10%, small cell 55% and large cell neuroendocrine tumours 71.4%. As expected, increasing stage was predictive of recurrence; 1b: OR 2.0 (1.39–2.91), 2a: OR 2.29 (1.47–3.58), 2b: OR 3.71 (2.37–5.85), 3a: OR 3.15 (2.03–4.86), 3b: OR 3.66 (1.07–13.03), as was positive resection margin, pleural or lymphovascular invasion (Table 1). Poorly differentiated tumours had significantly higher risk of recurrence when compared to tumours with moderate differentiation: OR 1.53 (1.16–2.02) and well differentiated tumours significantly lower risk of recurrence: OR 0.44 (0.20–0.87).

**Table 1:**
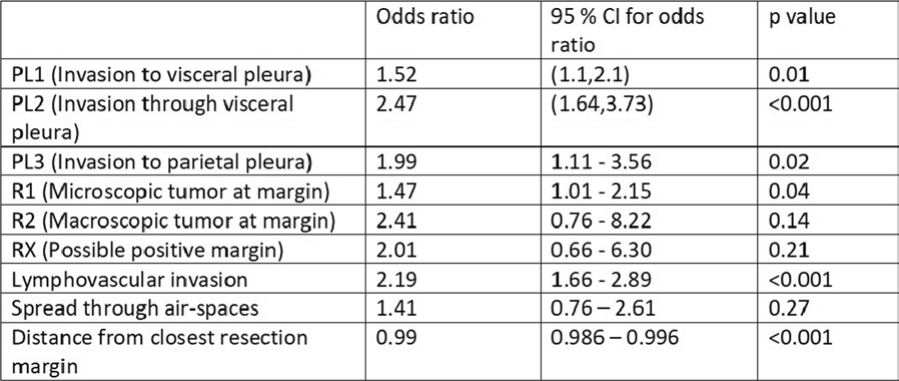
Likelihood of recurrence based on pathological markers

As well as increasing stage, pleural invasion, lymphovascular invasion and distance from resection margin were found to be strong predictors of recurrence in keeping with published literature. This data supports the proposition that both pleural and lymphovascular invasion are indicators for adjuvant therapy.

## A226 Extended resections for thymic tumours with pleural involvement

### Karishma Chandarana; Helen Weaver; Marinos Koulouroudias; Apostolos Nakas

#### Department of Thoracic Surgery, Glenfield Hospital, Leicester, UK

**Correspondence**: Karishma Chandarana

*Journal of Cardiothoracic Surgery 19(1):* A226

**Objectives:** ESMO guidelines advise surgical resection of thymomas if they are felt to be completely resectable upfront; classically this relates to Masaoka-Koga Stage I–III. To date, there is limited literature regarding resecting Masaoka-Koga Stage IV tumours (with pleural involvement).

We report a single centre series of patients with Masaoka-Koga Stage IV thymic tumours undergoing extended surgical resections.

**Methods:** A hospital operative database identified all cases of thymoma resections from May 2012 to August 2022. 86 cases of thymoma resections were identified during this time period. Ten patients (11.6%) underwent extended resections for Masaoka-Koga Stage IV thymoma.

**Results:** 90% of our cohort were female with a median age of 45 (range 35–63). 30% had a preoperative diagnosis of myasthenia gravis. Two patients underwent extended pleurectomy pneumonectomy (EPP) and eight patients extended pleurectomy decortication (EPD). 70% of patients had neoadjuvant chemotherapy and 80% had adjuvant radiotherapy. 60% of patients had recurrence, median DFI of 16 months (range 4–40). Survival was 90% at one year and 83% at 5 years.

**Conclusions:** ESMO guidelines state that resectable Masaoka-Koga Stage IV thymic tumours should undergo upfront surgery and receive postoperative radiotherapy. Our recurrence rate over this period was similar to that quoted in the literature 71% (34–100%). Although not all patients have completed 10 years follow up, overall mortality rate is currently 20% compared to that quoted in the literature, 42% (26–58%).

This series supports the use of extended resections in selected patients. As the operative procedure required is not performed by the majority of thoracic surgeons, we recommend that these cases are referred to dedicated centres with specialised multidisciplinary teams.

## A227 Impact of Sars-Cov2 pandemic on clinical and pathological staging in patients who underwent lung cancer resection in a high-volume thoracic surgery center

### Giulia Fabbri; Federico Femia; Savvas Lampridis; Stephanie F Fraser; Andrea Bille

#### Guy's Hospital, London, UK

**Correspondence**: Andrea Bille

*Journal of Cardiothoracic Surgery 19(1):* A227

**Objectives:** To evaluate the impact of Sars-Cov2 pandemic on clinical and pathological staging in patients underwent lung cancer resection in a high-volume thoracic surgery center.

**Methods:** We conducted an historic cohort study of consecutive patients who underwent anatomical lung resection for non-small-cell lung cancer (NSCLC) in a high-volume thoracic surgery center between January 2019 and July 2022, and we divided them in 6 groups according to the pandemic waves’ timeline. We analyzed clinical stage at presentation, pathological stage after surgery and incidence of upstaging in every group.

**Results:** We collected 483 patients, 112 in the pre-pandemic group, 67 in the 1st wave, 75 in the 2nd wave, 80 in the 3rd wave, 69 in the 4th wave and 80 in the 5th wave group. The distribution in percentage of different clinical and pathological stages in each wave’s population is shown in Fig. 1.

**Fig. 1:**
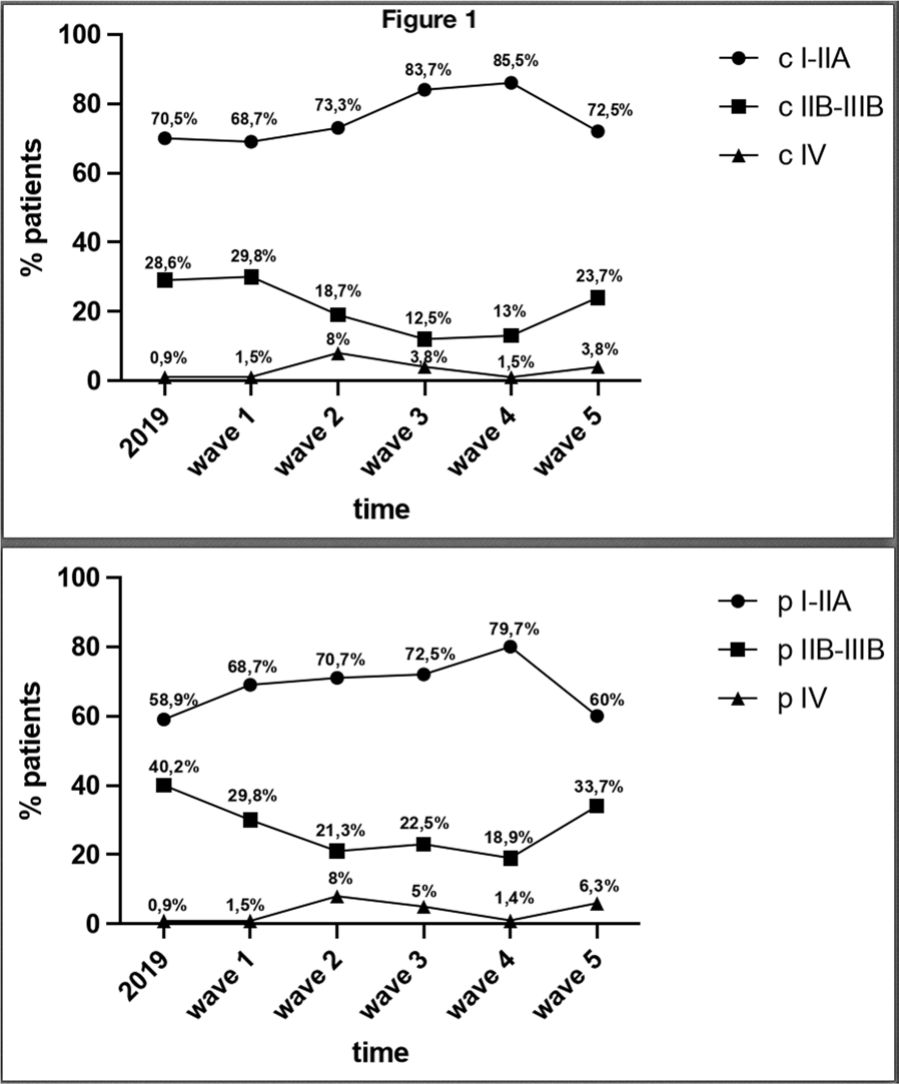
Distribution in percentage of different clinical and pathological stages in each wave’s population

The spike in patients with metastatic disease observed in the 2nd wave group was probably due to late presentation in clinic. There were no statistically significant differences in clinical and pathological stage rates between the groups along all the time span considered. The upstaging rate was 37,5% (n = 42), 40,3% (n = 27), 28% (n = 21), 25% (n = 20), 37,7% (n = 26), and 33,8% (n = 27) in the pre-pandemic, 1st, 2nd, 3rd, 4th and 5th wave group, respectively (p = 0.277).

**Conclusions:** In our center there were no significant differences in the distribution of clinical and pathological stages of NSCLC treated with surgery throughout the COVID pandemic period. Furthermore, upstaging rate didn’t differ, suggesting a stability in the diagnostic-therapeutic timeline for our patients. Lung cancer treatment pathway was prioritized in our national health system. Our center didn't experience reduction or delays in surgical activity, because patients were operated on in private facilities dedicated to elective admission. Multidisciplinary meetings (MDTs) were held every week virtually.

## A228 Strategies to improve diagnostic yield of electromagnetic navigational bronchoscopy (ENB): single-centre experience?

### Hanan Hemead; Hazem Fallouh; Vanessa Rogers; Ehab Bishay; Maninder Kalkat; Richard Steyn; Babu Naidu; Ashvini Menon

#### Queen Elizabeth Hospital, Birmingham, UK

**Correspondence**: Hanan Hemead

*Journal of Cardiothoracic Surgery 19(1):* A228

**Objectives:** Electromagnetic navigational bronchoscopy (ENB) has been increasingly utilized recently for diagnosis of peripheral nodules which are not amenable for conventional bronchoscopy. Ongoing technical refinement and growing experience improve the diagnostic yield of ENB.

**Methods:** Retrospective analysis of ENB procedures between November 2021 to October 2022 was performed.

**Results:** Twenty patients (n = 22) underwent ENB. The median age was 68 years (44–84 years). Most lesions were in the right upper lobe (n = 9, 45%), followed by left upper lobe (n = 6). The average size of lesions was 24.9 ± 10 mm ranging from 9 to 48 mm. Confirmed diagnosis of malignancy was established in 10 lesions (45%). Cervical mediastinoscopy was combined with ENB in four patients with suspected N2 disease. No adverse events were experienced. Fluoroscopy and selection of cases with positive bronchus sign (n = 7) improved the diagnostic yield of ENB.

**Conclusions:** ENB is a feasible and less invasive diagnostic procedure. Fluoroscopy, selection of lesions with positive bronchus sign and utilization of scans with good overlap can increase diagnostic yield. The impact of these factors will be analysed and added by the time of the conference.

## A229 Impact of mobile lung cancer screening (HANSE Trial in Northern Germany) on the caseload of a thoracic surgery unit

### Ana Beatriz Almeida; Michael Schweigert; Ahmed Hamdouna; Sabine Bohnet; Daniel Drömann

#### University Hospital Schleswig–Holstein, Luebeck, Germany

**Correspondence**: Ana Beatriz Almeida

*Journal of Cardiothoracic Surgery 19(1):* A229

**Objectives:** The HANSE Trial is a prospective study on mobile lung cancer screening at three centres in Northern Germany. One and a half year after its start we preliminary analysed the impact on the caseload of one of the participating thoracic surgery units.

**Methods:** Outcome, tumour stage, operative management and characteristics of all patients undergoing anatomical lung resection were analysed. Study period was April 2021 to October 2022.

**Results:** A total of 1764 patients underwent mobile lung cancer screening at our centre after enrolment into the HANSE Trial. In the same period of time 102 anatomical lung resections for lung cancer were carried out in curative intent. Out of these 102, 18 were participants of the HANSE Trial. There were no significant differences in age (p = 0.65), FEV one in percentage (p = 0.10) or FEV 1 in litres (p = 0,92) between the two groups. HANSE Trial mobile screening patients underwent lobectomy, bilobectomy and pneumonectomy in 15, one and two cases, respectively. HANSE Trial mobile screening patients had significantly higher odds for diagnosis in early pT1 stage compared to regular patients (OR 2.78; 95% CI: 0.99–7.88; p = 0.05). Moreover, non-screening patients had higher odds for the need of induction therapy (15/84 vs. 1/18; OR 3.70; 95% CI: 0.46–29.96). There was no mortality and no tumour recurrence among HANSE Trial screening patients during the study period.

**Conclusions:** Lung cancer screening with mobile trucks scanning those most at risk from lung cancer (current and ex-smokers) contributes significantly to the caseload of affiliated thoracic surgery units. Screening patients tend to be diagnosed in earlier tumour stages with excellent chance for curative operative treatment.

## A230 Pneumonectomy for N2 disease in non-small cell lung cancer

### Marcus Taylor; Kandadai Rammohan; Eustace Fontaine; Vijay Joshi; Felice Granato

#### Wythenshawe Hospital, Manchester, UK

**Correspondence**: Marcus Taylor

*Journal of Cardiothoracic Surgery 19(1):* A230

**Objectives:** Whilst multimodality treatment for stage III lung cancer is now established, the role of pneumonectomy.

in patients with N2 disease remains contentious. The aim of this study was to review short and long-term outcomes for patients with N2 disease undergoing pneumonectomy for non-small cell lung cancer.

**Methods:** All patients undergoing pneumonectomy for primary lung cancer with pathologically confirmed N2 disease between 2012 and 2018. Primary outcomes were 90-day mortality and overall survival. The log-rank test and Cox regression were used to analyse the impact of variables on overall survival.

**Results:** In total, 26 patients were included. The median post-operative length of stay was 6 days (interquartile range [IQR] 4–9 days) and 90-day mortality was 0%. Mean age was 64.5 years (± 10.7) and 34.6% (n = 9) of patients underwent right pneumonectomy. The median number of mediastinal lymph node stations sampled was 4 (IQR 4–5). The median number of positive mediastinal lymph node stations was one (IQR 1–2) and 26.9% (n = 7) of patients had multi-station N2 disease. Median follow-up time was 16 months (IQR 8–46 months) and estimated median overall survival was 15 months (95% confidence intervals [CI] 9–21 months). Undergoing right pneumonectomy was not associated with reduced overall survival (p = 0.940). However, the presence of multi-station N2 disease was associated with significantly reduced overall survival (p = 0.046). For this small subgroup of patients, estimated median overall survival was six months (95% CI 3–9 months).

**Conclusions:** This study has shown limited long-term survival for patients undergoing pneumonectomy for N2 disease. Whilst laterality of pneumonectomy did not affect outcomes, the presence of multi-station N2 disease was associated with extremely poor overall survival.

## A231 Predicting low sodium in patients undergoing lung resection for primary lung cancer

### Marcus Taylor; Kandadai Rammohan; Eustace Fontaine; Felice Granato; Vijay Joshi; Annabel Sharkey; Somshekar Ganti

#### Wythenshawe Hospital, Manchester, UK

**Correspondence**: Marcus Taylor

*Journal of Cardiothoracic Surgery 19(1):* A231

**Objectives:** Low sodium is the most common electrolyte abnormality for patients with lung cancer and may be associated with worse long-term outcomes. The aim of this study was to identify factors associated with low pre-operative sodium levels.

**Methods:** All consecutive patients undergoing lung resection for primary lung cancer during 2019 in a single UK centre were included. Sodium levels immediately prior to surgery were recorded. Patients with sodium < 135 mmol/L were considered to have low sodium, as defined by NICE. Patients with missing sodium values were excluded. Multivariable logistic regression analysis was used to identify factors associated with low pre-operative sodium levels.

**Results:** Overall, 422 patients were included, and 90-day mortality was 1.9% (n = 8). The mean age was 69.0 years (± 8.7) and 43.4% (n = 183) of patients were male. The median pre-operative sodium value was 140 (range 128–149, interquartile range 138–141). In total, 8.1% (n = 34) of patients had a low pre-operative sodium level. After multivariable analysis, age, male sex and smoking were not independently associated with low pre-operative sodium. However, lower body mass index (hazard ratio [HR] 0.883, 95% confidence intervals [CI] 0.808–0.965, p = 0.006), lower haemoglobin (HR 0.961, 95% CI 0.936–0.986, p = 0.003) and advanced cancer stage (HR 2.594, 95% CI 1.183–5.685, p = 0.017) were all independently associated with low pre-operative sodium.

**Conclusions:** Measures of physiological reserve and advanced malignancy emerged in this study as associated with low pre-operative sodium, which has been linked with poor long-term outcomes after lung cancer surgery. Further work should consider ways of addressing modifiable risk factors.

## A232 Outcomes after sublobar resection for early-stage lung cancer

### Sara Ugolini; Marcus Taylor; Kandadai Rammohan; Eustace Fontaine; Vijay Joshi; Felice Granato

#### Wythenshawe Hospital, Manchester, UK

**Correspondence**: Sara Ugolini

*Journal of Cardiothoracic Surgery 19(1):* A232

**Objectives:** Many contemporary studies advocate sublobar anatomical resection for early-stage lung cancer. The aim of this study was to compare outcomes for patients undergoing anatomical and non-anatomical sublobar resections for early-stage lung cancer.

**Methods:** All consecutive patients undergoing sublobar resection (wedge resection or segmentectomy) for stage T1a/b N0 primary non-small cell lung cancer between 2010 and 2020 in a single UK centre were included. Primary outcomes were 90-day mortality, post-operative length of stay (PLOS) and overall survival. Univariable analyses were undertaken to compare outcomes between groups.

**Results:** 116 patients were included in the study. The mean age was 69.7 years (± 7.8) and 41.4% (n = 48) were male. 56.9% (n = 66) underwent segmentectomy and 43.1% (n = 50) underwent lobectomy. Overall, 90-day mortality was 0% and median PLOS was six days (interquartile range 4–8 days). Despite a significantly higher mean number of mediastinal lymph node stations sampled in the segmentectomy group (3.4 vs 2.1, p = 0.002), the rate of post-operative nodal upstaging was not significantly different between groups (segmentectomy 1.5% [n = 1] vs wedge 4.0% [n = 4], p = 0.404). Margin to tumour ratio (MTR) was available for 97 patients. The incidence of MTR ≥ 1 was not significantly different between groups (segmentectomy 52.0% [n = 26] vs wedge 63.8% [n = 30], p = 0.239). Median follow-up time was 47 months. Overall survival was significantly reduced for the group undergoing wedge resection (log-rank analysis p < 0.001).

**Conclusions:** Despite similar pre, peri and post-operative characteristics, anatomical segmentectomy was associated with longer overall survival when compared to wedge resection for patients with T1a/b N0 non-small cell lung cancer.

## A233 Short and long-term outcomes after lung resection for stage III non-small cell lung cancer

### Marcus Taylor; Kandadai Rammohan; Eustace Fontaine; Vijay Joshi; Felice Granato

#### Wythenshawe Hospital, Manchester, UK

**Correspondence**: Marcus Taylor

*Journal of Cardiothoracic Surgery 19(1):* A233

**Objectives:** Multimodal therapy with chemoradiotherapy and surgery is recognised as an important treatment option for the management of stage III lung cancer. There is limited information available on outcomes for patients undergoing thoracic resection who are post-operatively classified as stage III. The objective of this study was to review short and long-term outcomes in this cohort.

**Methods:** All patients who underwent lung resection in a single UK centre between 2012 and 2019 and were post-operatively classified as having stage III lung cancer (as per the 8th edition of the TNM staging guidelines) were included. Primary endpoints were 90-day mortality, one-year mortality and overall survival. Cox regression analysis was used to evaluate the impact of variables on overall survival.

**Results:** In total, 629 patients were included. Overall, 90-day and one-year mortality were 4.6% (n = 29) and 22.7% (n = 143), respectively. Patients were classified as stage IIIa (77.3%, n = 486), IIIb (21.8%, n = 137) and IIIc (1.0%, n = 6). Mortality for stage IIIa and IIIb/c patients was 3.9% (n = 19) and 7.0% (n = 10) at 90-days (p = 0.122) and was 20.8% (n = 101) and 29.4% (n = 42) at 1-year (p = 0.031). Median follow-up time was 31 months and estimated median overall survival was 34 months (95% confidence intervals [CI] 28–40 months). After Cox multivariable regression analysis, stage III subgroup (IIIa vs IIIb/c) was not associated with significantly reduced overall survival. Both anaemia (hazard ratio [HR] 1.355, 95% confidence intervals [CI] 1.099–1.671, p = 0.004) and lower body mass index (HR 0.974, 95% CI 0.953–0.994, p = 0.012) were associated with significantly reduced overall survival.

**Conclusions:** Although patients with stage IIIb/c lung cancer had significantly higher mortality at one year in comparison to stage IIIa patients, in this cohort stage IIIb/c lung cancer was not associated with significantly reduced overall survival compared to stage IIIa after multivariable adjustment.

## A234 The impact of sodium levels on post-operative length of stay

### Marcus Taylor; Kandadai Rammohan; Eustace Fontaine; Felice Granato; Vijay Joshi; Annabel Sharkey; Somshekar Ganti

#### Wythenshawe Hospital, Manchester, UK

**Correspondence**: Marcus Taylor

*Journal of Cardiothoracic Surgery 19(1):* A234

**Objectives:** Low sodium is the most common electrolyte abnormality for patients with lung cancer. The aim of this study was to determine whether low sodium levels prior to lung resection or a drop in sodium after lung resection impact on post-operative length of stay (PLOS).

**Methods:** All consecutive patients undergoing lung resection during 2019 in a single UK centre were included. Sodium levels immediately prior to surgery and immediately after surgery were recorded and expressed as continuous variables. The difference between immediate pre- and post-operative sodium levels was also measured. Low sodium was also considered as a dichotomous variable, using a definition of < 135 mmol/L, as defined by NICE. Patients with missing sodium values were excluded. Primary outcomes were 90-day mortality and PLOS. Linear regression was used to determine the effect of sodium on PLOS.

**Results:** A total of 552 patients were included. Overall, 90-day mortality was 1.8% (n = 10). The median PLOS was five days (interquartile range 3–8 days, range 1–73 days). The median pre-operative sodium value was 140 (range 128–149) and the median change in sodium from before and after surgery was − 4 (range − 17 to + 6). When considered as continuous variables, neither lower pre-operative sodium nor larger drop in sodium before and after surgery were associated with significantly longer PLOS. When considered as a dichotomous variable, low pre-operative sodium was also not associated with significantly longer PLOS.

**Conclusions:** These results suggest that low or falling sodium levels before and after lung resection do not impact on PLOS.

## A235 A systematic review of the prognostic role of serum sodium levels in patients undergoing surgical resection of non-small cell lung cancer

### Asmita Singhania; Marcus Taylor; Annabel Sharkey; Somshekar Ganti

#### Wythenshawe Hospital, Manchester, UK

**Correspondence**: Asmita Singhania

*Journal of Cardiothoracic Surgery 19(1):* A235

**Objectives:** Low serum sodium concentration has been implicated as a negative prognostic marker for several malignancies. Its role in the context of non-small cell lung cancer (NSCLC) has not been well explored. The aim of this study was to review the literature on the role of sodium levels in resected non-small cell lung cancer (NCSLC).

**Methods:** A systematic review of PubMed and Cochrane library databases was performed using terms related to NSCLC and lung resection and sodium. Case reports and non-English language studies were excluded.

**Results:** The search strategy yielded 13 results, of which 10 were excluded for lack of relevance and one was not written in English. No relevant Cochrane library reviews were identified. Two studies were included in the final review comprising 1690 patients. Kobayashi et al. undertook a single-centre study of 386 patients undergoing surgery between 2000 and 2009 with a median follow-up time of 41 months. Hyponatraemia was defined as sodium < 139 mEq/L and was associated with significantly reduced overall survival on multivariable analysis. Li et al. undertook a single-centre study of 1304 patients undergoing surgery between 2007 and 2014 with a median follow-up time of 41 months. Hyponatraemia was defined as sodium < 141.9 mEq/L and was also associated with significantly reduced overall survival on univariable analysis in patients without an anion gap (n = 671) but not those with a low (n = 221) or high (n = 412) anion gap.

**Conclusions:** Despite a limited number of studies, there is a small amount of evidence to suggest that low sodium levels are associated with poor prognosis in patients undergoing resection for NSCLC. Additional large-scale studies are required to explore this further.

## A236 The role of sodium in predicting long-term outcomes after lung resection

### Marcus Taylor; Kandadai Rammohan; Eustace Fontaine; Felice Granato; Vijay Joshi; Annabel Sharkey; Somshekar Ganti

#### Wythenshawe Hospital, Manchester, UK

**Correspondence**: Marcus Taylor

*Journal of Cardiothoracic Surgery 19(1):* A236

**Objectives:** Low sodium is the most common electrolyte abnormality for patients with lung cancer. However, the prognostic significance of low sodium in patients undergoing lung resection is unknown. The aim of this study was to review the impact of sodium levels on outcomes after lung resection.

**Methods:** All consecutive patients undergoing lung resection during 2019 in a single UK centre were included. Sodium levels immediately prior to surgery and immediately after surgery were recorded and expressed as continuous variables. The difference between immediate pre- and post-operative sodium levels was also measured. Patients with missing sodium values were excluded. Primary outcomes were 90-day mortality and overall survival. Multivariable Cox regression analysis was used to identify factors associated with reduced overall survival.

**Results:** A total of 552 patients were included. Overall, 90-day mortality was 1.8% (n = 10). The median pre-operative and post-operative sodium values were 140 (range 128–149) and 136 (range 125–144), respectively. On univariable analysis, lower pre-operative sodium was associated with reduced overall survival (hazard ratio [HR] 0.904, 95% confidence intervals [CI] 0.825–0.991, p = 0.031). Post-operative sodium levels and change in sodium level were not associated with reduced overall survival on univariable analysis. Following adjustment for age, gender, body mass index and the presence of malignant disease, lower pre-operative sodium remained independently associated with reduced overall survival (HR 0.880, 95% CI 0.799–0.968, p = 0.008).

**Conclusions:** These results suggest that whilst a post-operative drop in sodium levels does not affect outcomes, patients with lower pre-operative sodium levels may experience reduced overall survival after lung resection.

## A237 The significance of margin to tumour ratio in sublobar resections for non-small cell lung cancer

### Sara Ugolini; Marcus Taylor; Kandadai Rammohan; Eustace Fontaine; Vijay Joshi; Felice Granato

#### Wythenshawe Hospital, Manchester, UK

**Correspondence**: Sara Ugolini

*Journal of Cardiothoracic Surgery 19(1):* A237

**Objectives:** Some studies investigating the significance of margin to tumour ratio (MTR) have found MTR ≥ 1 is associated with improved prognosis for patients undergoing sublobar resection for stage I non-small cell lung cancer. The aim of this study was to analyse the prognostic value of MTR in our own cohort of patients.

**Methods:** All consecutive patients undergoing sublobar resection (wedge resection or segmentectomy) for stage I non-small cell lung cancer (staged as per TNM 8) between 2010 and 2020 in a single UK centre were included. Patients with missing tumour diameter or stapled margin were excluded. Multivariable Cox regression analysis was performed to assess the impact of MTR ≥ 1 on overall survival.

**Results:** 127 patients were included in the study. The mean age was 71.2 years (± 7.7) and 47.2% (n = 60) were male. 48.0% (n = 61) underwent segmentectomy and 52.0% (n = 66) underwent sublobar wedge resection. 85.0% (n = 108) of patients underwent surgery via thoracotomy. Overall, 90-day mortality was 0.8% (n = 1). Median MTR was 0.71 (IQR 0.31–1.25 mm) and 42.5% (n = 54) of patients had MTR ≥ 1. Median follow-up time was 47 months. After multivariable analysis including age, anaemia, laterality, body mass index and procedure type, MTR < 1 was independently associated with reduced overall survival (hazard ratio [HR] 1.985, 95% confidence intervals [CI] 1.114–3.539, p = 0.020). This effect was retained in the segmentectomy subgroup (HR 3.195, 95% CI 1.068–9.556, p = 0.038), but not in the wedge subgroup (HR 1.462, 95% CI 0.712–3.003, p = 0.301).

**Conclusions:** In patients with stage I non-small cell lung cancer undergoing anatomical segmentectomy, MTR < 1 was independently associated with reduced overall survival. Intra-operative consideration of resection margins should be a key area of focus for thoracic surgeons performing sublobar resection.

## A238 Short-term outcomes of robotic versus uniportal video-assisted approach in anatomical lung resection for lung cancer: a single centre experience

### Ahmed El-Zeki; Hannah Jesani; Ahmed Habib; Patrick Yiu; Ahmed Oliemy

#### Royal Wolverhampton NHS Trust, Wolverhampton, UK

**Correspondence**: Ahmed El-Zeki

*Journal of Cardiothoracic Surgery 19(1):* A238

**Table:** 

	RATS (n = 96)	uVATS (n = 62)	p-value
Segmentectomy	18	5	0.069
Operative time (hours)	4.52 (± 1.24)	3.54 (± 0.88)	0.000
Number of lymph node stations	4 (1–8)	4 (1–7)	0.798
30 days mortality	1	2	0.326
Hospital stay (Days)	3 (1–42)	4 (1–31)	0.233
Drain duration (Days)	2	2	0.895
Need for assisted ventilation	2	3	0.334
Pneumonia treated with antibiotics	14	7	0.552
Surgical re-exploration	1	4	0.058
Air Leak > 7 days	21	18	0.308

**Objectives:** Robotic assisted thoracic surgery (RATS) is becoming increasingly popular in the UK since 2017. Uniportal video assisted thoracic surgery (uVATS) is currently the least invasive approach for lung cancer resection. Comparing the outcome of both approaches will help to answer the ongoing questions about their safety and efficacy.

**Methods:** Primary lung cancer patients treated with curative intent anatomical lung resection via RATS or uVATS in our institution from October 2019 to September 2022 were included. Early post operative outcome was analysed retrospectively and compared between both approaches.

**Results:** A total of 158 patients met the inclusion criteria, 96 of them had RATS and 62 had uVATS anatomical lung resection. There were no significant differences in the pre-operative criteria. The mean operative time was significantly longer in RATS (P = 0.000), there was a trend towards more segmentectomies performed with RATS (P = 0.069). Both groups were similar in number of lymph node stations dissected, 30 days mortality, hospital stay, drain duration and complication rates. There was a trend towards higher rate of surgical re-exploration in the uVATS group (P = 0.058).** Table-1.**

**Conclusions:** Looking at the short-term outcome, both RATS and uVATS are safe and feasible for curative anatomical lung cancer resection, the approach choice did not affect the overall patients’ outcome. RATS technology helped performing more complex anatomical segmentectomy procedures, RATS was also associated with lower surgical re-exploration rate.

## A239 Improving enhanced recovery after surgery (ERAS) pathway compliance for high-risk thoracic surgery patients

### Ben Cornwell; Genevieve O'Farrell; Matt Molyneux

#### Bristol Royal Infirmary, Bristol, UK

**Correspondence**: Ben Cornwell

*Journal of Cardiothoracic Surgery 19(1):* A239

The ERAS guidelines for thoracic surgery advocates standardised evidence-based care to improve patient outcomes. Our Trust thoracic surgery ERAS pathway includes a standard post-operative analgesia prescription to support regional techniques and minimise opiate prescribing. At present we do not know how well standard pathways are adhered to. This project aims to assess and improve compliance with ERAS pathways.

A retrospective audit was conducted. Patient records for all those undergoing video-assisted thoracic surgery (VATS) during a 5-month period in 2022 were reviewed. Analgesic prescribing on post-operative days 0, 1 and 2 was assessed for compliance against our post-operative analgesia pathway for VATS surgery.

Seventy-seven patients underwent VATS during the audit period. In patients cared for in a ward-based setting there was 85% compliance with ERAS post-operative analgesia, however compliance in those cared for in a high dependency unit (HDU) setting was 63%.

A large proportion of higher risk patients do not receive ERAS analgesia bundles, rather than this being related to patient risk profile we suggest that this is due to barriers in post-operative prescribing and lack of staff education. To improve prescribing, we have introduced an electronic VATS post-operative analgesia prescription bundle and staff education sessions.

## A240 Predictors of mid-term survival after anatomical resection for primary lung cancer

### Bartlomiej Szafron; Haisam Saad; Obada Alqudah; Waldemar Bartosik; Vasileios Kouritas; Aleksander Mani; Jakub Kadlec

#### Norfolk and Norwich University Hospital, Norwich, UK

**Correspondence**: Bartlomiej Szafron

*Journal of Cardiothoracic Surgery 19(1):* A240

**Objectives:** The aim of the study was to establish risk factors affecting mid-term survival after surgery for primary lung cancer. Special attention was paid to histological variables contributing to final pathological staging.

**Methods:** Records of 221 patients operated on between Jan 2020 and Aug 2021 were reviewed retrospectively. Cox regression and Kaplan–Meier curves were applied accordingly for survival analysis. Clinical variables included age, gender, American Society of Anaesthesiologists (ASA) classification grade, type of surgical approach, operated body side, number of resected segments, sub-lobar resection, pneumonectomy. Histological variables included type of cancer, whole tumour size, size of the invasive component, nodal spread, completeness of resection, pleural breaching and vascular invasion.

**Results:** Mean observation time was 649 days. After Cox regression analysis three variables were retained in the model: ASA grade (Exp(b) 3.51, 95% CI: 1.40–8.84, p = 0.01), invasive component size (Exp(b) 1.03, 95% CI: 1.02–1.05, p < 0.0001), incomplete resection (Exp(b) 3.43, 95% CI: 1.01–11.63, p = 0.05). Kaplan–Meier curves analysis confirmed significant difference in mean survival time for ASA higher than 2, incomplete resection and invasive tumour size bigger than 10 mm. Out of other components of pathological staging, not included in the model, also nodal spread and vascular invasion proved significant in Kaplan–Meier analysis. After case–control matching for ASA grade, incomplete resection, vascular invasion and nodal spread, invasive component size bigger than 45 mm was established as a significant risk factor.

**Conclusions:** ASA grade is a strong clinical predictor of mid-term survival. Incomplete resection and size of the invasive component are significant histological predictors. Significance of invasive component size and its critical value may vary depending on ASA grade and absence of incomplete resection, nodal spread and vascular invasion (Fig. 1).

**Fig. 1:**
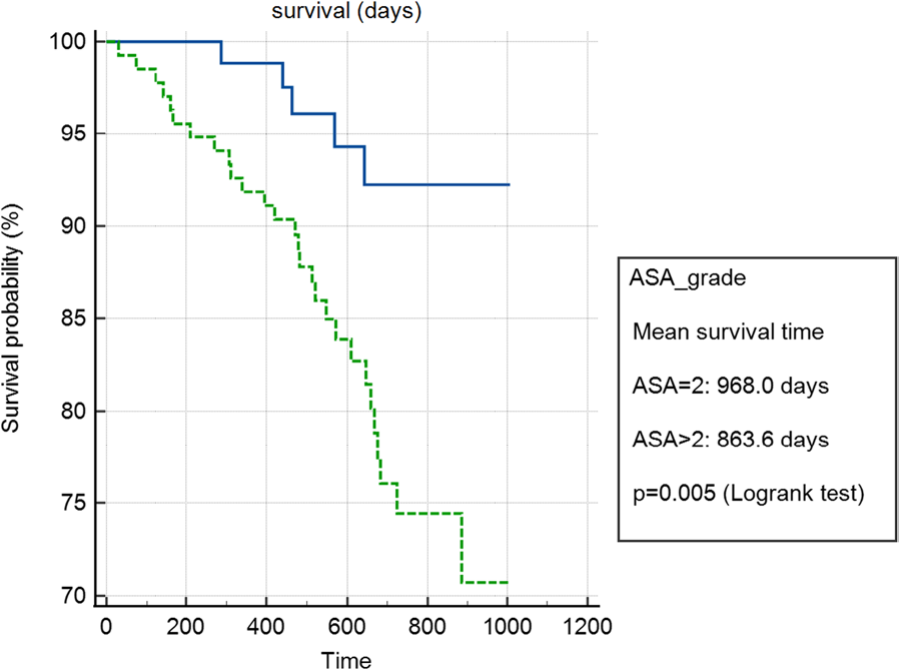
Kaplan–Meier curves for different ASA grades

## A241 Short-term outcome of robotic anatomical sub-lobar lung resection in comparison with VATS, a single institution experience

### Obada Alqudah; Owais Shah; Haisam Saad; Nicole Asemota; Aly Pathan; Bartlomiej Szafron; Aleksander Mani; Jakub Kadlec; Waldemar Bartosik; Vasileios Kouritas

#### Norfolk and Norwich University Hospital, Norwich, UK

**Correspondence**: Obada Alqudah

*Journal of Cardiothoracic Surgery 19(1):* A241

**Objectives:** Robotic-Assisted Thoracoscopic Surgery (RATS) is only recently utilized in anatomical lung resections. Consequently, its utilization in sublobar lung resections is often avoided despite recent findings, that sublobar anatomical lung resections offer better survival than lobectomies.

Aim of the study was to compare the outcomes of RATS versus Video-Assisted thoracoscopic surgery (VATS) sublobar lung resections.

**Methods:** Within two years, patients who underwent RATS and VATS sublobar anatomical lung resections were retrospectively investigated. Data analyzed, apart from the demographics, included multiple variables compared between the two groups.

**Results:** Overall, 45 RATS and 38 VATS patients were analyzed. The mean age was 69.6 ± 8.2 years and 44 (53%) were females. The age, gender, PS, LFTs and the type and staging of lesions resected were similar between the two groups. Duration of procedure, upstages and R1 resections were similar in both groups. Nevertheless, complex segmentectomies were performed more via RATS than via VATS (36.1% vs 9.6%, p = 0.001). Respiratory complications were lower in RATS group (17.8% vs 36.8%, p = 0.043) and so was the need for intravenous antibiotics (13.3% vs 36.8%, p = 0.012). for RATS patients, length of stay (LOS) was shorter (3 (1–11) vs 4 (2–25), p = 0.018) and escalation to critical care was less frequent (0% vs 10.5%, p = 0.026). Although in-hospital/30-day mortality was similar, late deaths were observed more in VATS goup patients (2.2% vs 15.8%, p = 0.033).

**Conclusions:** Utilization of RATS approach for sublobar anatomical lung resections is not inferior to VATS, even more complex sublobar resections were performed via RATS in this study which showed superior outcomes. RATS could be one of the preferable approaches for managing patients who require sublobar anatomical lung resections, especially the complex ones.

## A242 Advanced RATS hybrid approach for pancoast tumour

### Obada Alqudah; Haisam Saad; Joana Fuentes-Warr; Nicole Asemota; Aly Pathan; Jonathon Francis; Elynda Bailon; Lydia Rhodes; Vasileios Kouritas

#### Norfolk and Norwich University Hospital, Norwich, UK

**Correspondence**: Obada Alqudah

*Journal of Cardiothoracic Surgery 19(1):* A242

**Objectives:** Robotic-Assisted Thoracoscopic Surgery (RATS) is a new technology and its acceptance in complex cases is very limited.

We herein present the case of a posterior Pancoast treated with RATS resection combined with limited posterior thoracotomy.

**Methods:** A 40-year-old male patient presented with a 4-month history of right upper chest pain radiating to his right arm.

Investigations demonstrated a 5.7 × 4.3 cm right upper lobe apico-posterior mass invading the chest wall posteriorly at the level of 2nd–4th ribs, suggesting Pancoast tumour, with a radiological stage of T3N0M0 (Fig. 1A). Attempts to obtain a preoperative diagnosis failed.

**Fig. 1:**
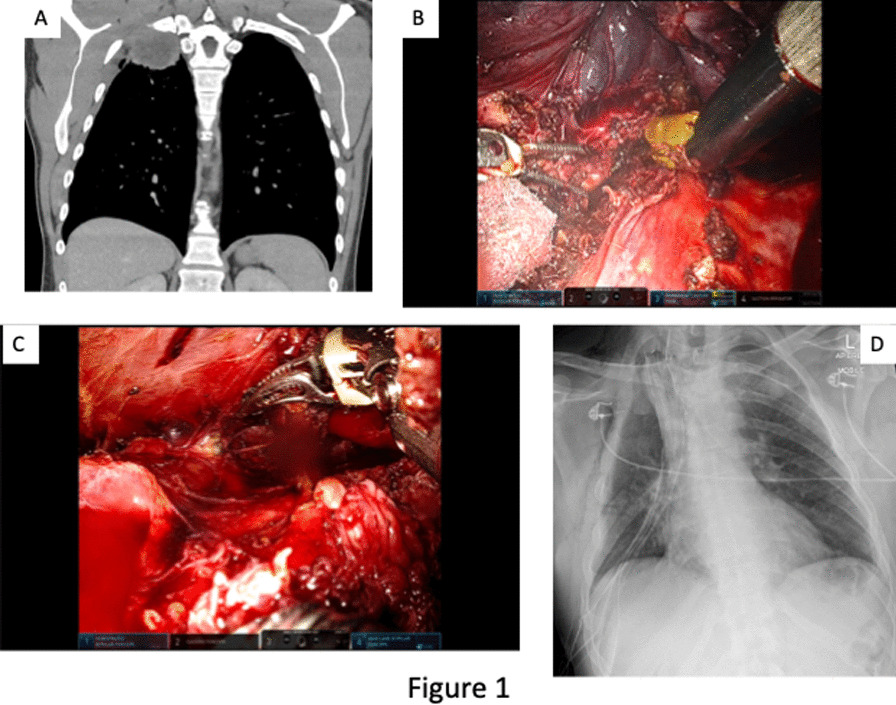
**A** Right upper lobe tumour invading the chest wall, **B** Anterior chest wall dissection from outside towards the apex, where Brachial plexus was preserved, **C** Posterior chest wall dissection and disseconnection of ribheads with transverse processes, **D** Post operative Chest Xray after resection

**Results:** A RATS resection was attempted with 4 ports and an assisting port to facilitate rib resection.

A standard upper lobectomy was performed.

The chest wall resection was accomplished with ‘‘Kerrison-Rongeur’’ via the assistant port, starting from the anterior aspect of the ribs. Dissection progressed outside the chest wall towards the apex, where the brachial plexus was identified and preserved (Fig. 1B). Posteriorly dissection continued under the scapula towards the spine. The rib heads with the transverse processes were disconnected with diathermy (Fig. 1C).

At this point a high limited posterior thoracotomy was performed to ensure that R0 resection is achieved and remove the specimen.

Postoperative recovery was uneventful and the patient was discharged on day 5 (Fig. 1D). The final pathology was squamous cell lung cancer pT3N0V1PL3R0. He received adjuvant chemotherapy and radiotherapy. Six months post-surgery he is well and without relapse of the cancer.

**Conclusions:** RATS can be utilized to successfully treat complex locally advanced lung cancer cases, which include extensive chest wall resection, as shown in the present Pancoast case. Avoidance of a prolonged extensive thoracotomy in such cases can provide quicker recovery.

The patient gave the permission to publish this information and images in an open access journal.

## A243 Bronchectomy in the management of patients with bronchial carcinoid tumour

### Nerielle Fundano; Rory Beattie; Alastair Graham

#### Royal Victoria Hospital, Belfast, UK

**Correspondence**: Nerielle Fundano

*Journal of Cardiothoracic Surgery 19(1):* A243

**Objectives:** Bronchectomy is a bronchoplastic technique which maximises the preservation of normal lung parenchyma. The purpose of this study is to highlight the safety and efficacy of bronchectomy in the management of patients with endobronchial carcinoid tumours.

**Methods:** The electronic database from a single institution was retrospectively reviewed from 2007 to 2022 for all patients who underwent bronchectomy without lung resection. In each case bronchoscopy was performed in theatre prior to bronchectomy via thoracotomy. Intra-operative frozen sections were performed to ensure clear resection margins.

**Results:** A total of six patients were identified over a 25-year period. Four had left sided lesions (two left main bronchi and two lobar bronchi) with the remainder having tumours in the bronchus intermedius. Median age at time of operation was 39 and median length of stay was six days. The only significant post operative complication was one patient who needed bronchoscopic dilatation. Histopathology confirmed typical carcinoid in five patients and atypical carcinoid in one patient. Median follow up was 5.8 years during which there was no endobronchial or lung parenchymal recurrence. One patient with pathological n1 staging developed isolated nodal recurrence which was successfully resected.

**Conclusions:** We have demonstrated that bronchectomy is a safe and effective technique allowing both complete resection and complete preservation of lung parenchyma when used in carefully selected patients.
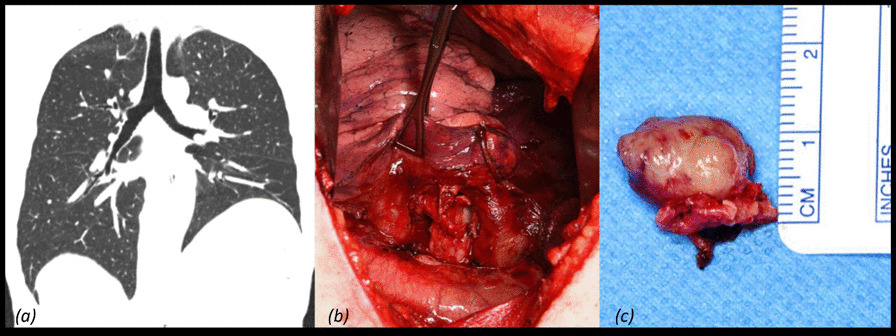


(a) CT chest of patient with typical carcinoid tumour in the left main bronchus, (b) bronchectomy in the same patient, (c) resected tumour.

## A244 What are the predictors of postoperative increase in dyspnoea after lung cancer surgery?

### Saffana K Algaeed^1^; Veena Surendrakumar^1^; Rajnikant L Metha^2^; Hazem Fallouh^1^; Ehab Bishay^1^; Vanessa Rogers^1^; Ashvini Menon^1^; Richard Steyn^1^; Maninder Kalkat^1^; Babu Naidu^1^

#### ^1^University Hospitals Birmingham (UHB), Birmingham, UK; ^2^University of Birmingham, Birmingham, UK

**Correspondence**: Saffana K Algaeed

*Journal of Cardiothoracic Surgery 19(1):* A244

**Objectives:** Many patients did not experience any recovery in their breathing after lung cancer surgery. This study aims to investigate the predictors of postoperative dyspnoea increase and the changes in QoL indices over five months following lung cancer surgery (Table 1).

**Table 1:** Multivariable regression analysis of dyspnoea increase at five months postoperatively

Predictor	Odds ratio	Std. Err	p-value	95% CI	
*Model 1*					
Baseline dyspnoea	1.05	0.004	0.00	1.05	1.1
Current Smoking	2.3	0.7	0.006	1.2	4.4
Smoking: never	0.5	0.13	0.01	0.3	0.8
PPODLCO	0.98	0.005	0.02	0.97	0.99
*Model 2*					
Baseline dyspnoea	1.05	0.004	0.00	1.05	1.1
Baseline fatigue	1.01	0.005	0.002	1.01	1.03
LOS > 5 days	1.6	0.3	0.01	1.1	2.4

PPO %DLCO, predicted postoperative diffusing capacity of carbon monoxide; LOS, length of hospital stay. P-value < 0.05 is considered statistically significant.

**Methods:** This is a prospective longitudinal, single-centre study that recruited patients with lung cancer surgery. EORTC QLQ-C30 and LC13 were introduced at baseline, 6 weeks, and 5 months post-surgery. Multivariate logistic regression analysis was used to investigate the predictors of postoperative increase in the dyspnoea domain five months post-surgery.

**Results:** A total of 1275 patients were recruited, and 906 screened patients were included in this study. EORTC QLQ-C30 dyspnoea scores at baseline, 6 weeks, and 5 months post-surgery were (20.4 ± 25.4), (41.9 ± 29.6), and (39.2 ± 29.4), respectively.

In the multivariable analysis, baseline dyspnoea and fatigue, smoking status and quit time, predicted postoperative diffusing capacity of carbon monoxide (ppoDLCO), and length of hospital stay were associated with the increase of dyspnoea after surgery (p < 0.05).

**Conclusions:** In conclusion, QoL is essential for decision-making and patient selection before treatments. Therefore, utilising them in clinics is highly encouraged.

## A245 Stromal Ki67 has a predictive value in determining outcomes following extended pleurectomy/decortication for malignant pleural mesothelioma

### Kudzayi Kutywayo^1^; Dean Fennell^2^; Apostolos Nakas^1^

#### ^1^Glenfield Hospital, Leicester, UK; ^2^University Hospital of Leicester NHS Trust, Leicester, UK

**Correspondence**: Kudzayi Kutywayo

*Journal of Cardiothoracic Surgery 19(1):* A245

**Objectives:** Malignant pleural mesothelioma has a low survival rate. Multimodality treatment involves surgery (pleurectomy/decortication), chemotherapy or immunotherapy.

Several factors that determine prognosis in MPM include: histologic subtype, sex, age, TNM stage, performance score (PS), weight loss, and several peripheral blood values.

It remains uncertain how patients with similar stage and histologic subtype have a wide variance in outcome. Ki67 is a proliferation marker which has shown prognostic value in other malignancies. We endeavoured to see if this would be the case in malignant pleural mesothelioma.

**Methods:** As part of an ongoing study, pleural tissue from 50 malignant pleural mesothelioma consecutive patients undergoing extended pleurectomy/decortication was collected during surgery. Tissue cores were stained in tissue microarray panels. Multiplex immunofluorescence was performed containing Ki67, pancytokeratin and DAPI.

**Results:** The median age was 70.50, M:F = (3.8:1). Histology: epithelioid 89.58%, biphasic 10.42. 91.67% had microscopic residual tumor (R1). Median PFS and OS were 162.50 and 387.50 days respectively. PFS and OS had moderate correlation (0.68). Survival analysis of high Ki67 expression vs low Ki67 expression showed a significant difference when stromal proliferation ratio was used as the proliferation index (p = 0.002), Surprisingly, this difference was not seen when tumor proliferation ratio was used (p = 0.3).

**Conclusions:** Stromal Ki67 was shown to have strong predictive value in patients who underwent pleurectomy/decortication. This can help in future patient selection as it identifies patients most likely to benefit from surgery.

## A246 Weathering the storm: the lack of impact of the COVID-19 pandemic on an established enhanced recovery protocol

### Charlotte Holmes; Laura Socci; Jagan Rao; John Edwards; Sara Tenconi; David Hopkinson; Roxanna Zakeri

#### Northern General Hospital, Sheffield, UK

**Correspondence**: Charlotte Holmes

*Journal of Cardiothoracic Surgery 19(1):* A246

**Objectives:** Our Enhanced recovery (ERAS) protocol was established in 2014. The impact was reduced length of stay (LOS), morbidity and mortality. The protocol includes preoperative nutrition, smoking cessation, day of surgery admission (DOSA), maintaining normothermia, protocolised analgesia and protocolised drain management. Our aim was to assess the impact of the COVID-19 pandemic on an established ERAS programme.

**Methods:** 50 consecutive patients undergoing lung cancer resection were identified between December 2020 and February 2021. Medical notes were used to collect data. Outcomes included adherence to the ERAS protocol, LOS, postoperative complications, and mortality. Outcomes are compared with a 2014 cohort of 50 patients post ERAS implementation.

**Results:** There was no significant difference in patient demographics. 93% (vs 22%) were referred for smoking cessation. 94% (vs 28%) of patients received carbohydrate drinks. 72% (vs 68%) of patients were DOSA. Mean LOS and Median LOS was comparable; six days and four days respectively for both groups. 82% (vs 88%) patients were normothermic. 100% had paravertebral catheters and intercostal blocks (vs 88%). 98% had oxycodone or codeine; 4% required a PCA (vs 94% requiring a PCA). 98% (vs 76%) had drains removed as per protocol. Postoperative complications were comparable: air leak (14% vs 10%), chest infection (28% vs 28%), AF (8% vs 8%), ITU admission (4% vs 4%) and readmission (6% vs 10%). Inpatient mortality was 2% in both groups.

**Conclusions:** The data period is nine months into the pandemic which would have allowed time for the team to learn, adapt and deliver education. However, there has been increased adherence to the ERAS protocol over time, as it has become embedded in clinical practice. This is shown by the lack of impact on adherence to the ERAS protocol and patient outcomes during the COVID-19 pandemic.

## A247 Evaluation of relationship between early mobilisation and length of hospital stay in a thoracic patient population: a single centre experience

### Andrew Chrisp; Qingzi Guo; Lakshmi Srinivasan; Kajan Mahendran; Udo Abah

#### Royal Stoke, Stoke on Trent, UK

**Correspondence**: Andrew Chrisp

*Journal of Cardiothoracic Surgery 19(1):* A247

**Objectives:** Early mobilisation after surgery aims to improve operative outcomes by counteracting the surgical stress response, increasing early lung expansion, and decreasing patient-reported pain scores. Our goal was to assess the rate of same-day mobilisation in a single centre with no specific early mobilisation programme in place, and how this affected length of stay.

**Methods:** We undertook opportunistic data collection of post-surgical thoracic patients in both intensive care and a ward-based setting to ascertain time from surgical closure to first mobilisation, over a 3-month period. The patients were sorted into three groups based on time to mobilise and the mean length of stay was calculated.

**Results:** We found an increase in mean length of stay (days) between patients mobilising within 12 h (2.7), between 13 and 23 h (4.6) and after 24 h (6.1). 40% of ward-based patients were mobilised on the day of surgery as compared to 0% of the intensive care patients, with average length of stay being 140% shorter for ward-based patients.

**Conclusions:** We explore possible reasons for this correlation including post-surgical pain, location of discharge following recovery, and degree of lung volume reduction. Finally, we discuss modifiable barriers to early mobilisation and suggest ways that these can be overcome by a structured mobilisation programme, with a particular focus on intensive care patients for whom the length of stay was longest.

## A248 Outcomes of robotic anatomical lung resections in high-risk patients in comparison with vats and thoracotomy

### Nicole Asemota; Owais Shah; Santosh Thandayuthapani; Jenny Advincula; Bartlomiej Szafron; Aleksander Mani; Jakub Kadlec; Waldemar Bartosik; Michael Irvine; Vasileios Kouritas

#### Norfolk and Norwich University Hospital, Norwich, UK

**Correspondence**: Nicole Asemota

*Journal of Cardiothoracic Surgery 19(1):* A248

**Objectives:** Robotic-assisted thoracoscopic surgery (RATS) is a relative new approach. Patient selection is often restricted to low-stage, low risk patients, despite the known benefits of RATS. We reviewed high risk cases, discussed at our local high-risk MDT, comparing the outcomes of those who underwent RATS to VATS and open surgery (OS).

**Methods:** Retrospective analysis of high-risk patients who underwent anatomical lung resection from May 2019–September 2022. Pneumonectomy patients were excluded. Demographics, preoperative data and postoperative outcomes were analysed.

**Results:** Overall, 89 eligible patients were divided into three groups: RATS (n = 22), VATS (n = 45) and OS (n = 22). The mean age was 69.88 ± 9.8 years, with 45 (61.8%) males. Baseline characteristics were similar amongst groups. VE/VCO2 was higher (p = 0.037) and the Anaerobic Threshold (AT) lower in the RATS group (p = 0.031). More patients with higher stage tumours (Stage 2 or 3) underwent RATS or OS (p = 0.016).

Complications, re-admissions, upstages and returns to theatre were similar amongst groups. Length of stay was less with RATS (median 4 vs 6 vs 7, p = 0.002) and so were the unplanned critical care admissions in comparison to VATS and OS respectively (0% vs 13.3% vs 18.2%, p = 0.042).

Overall deaths were less in the RATS group (0 vs 9% vs 5.6%, p = 0.034) but long-term survival was similar (log-rank 1.988, p = 0.370, Fig. 1). Overall death rates showed correlation with surgical approach (p = 0.033). Regression analysis demonstrated surgical approach (RATS to OS) as a factor of death prediction (p = 0.039).

**Conclusions:** Anatomical lung resection using RATS in high-risk patients is equally as safe as with VATS and OS and better in variable outcomes. RATS should not be excluded for managing surgically high-risk patients, especially as surgical approach may be a factor in death prediction.

## A249 A single-centre experience: comparing subxiphoid versus intercostal uniportal video-assisted thoracoscopic pulmonary lobectomy for primary lung cancer

### Jacie Jiaqi Law^1^; Karen Chien Lin Soh^2^; Pranav Kumar Santhosh^2^; Jeremy Chan^3^; Giuseppe Aresu^4^

#### ^1^Royal Victoria Hospital, Belfast, UK; ^2^University of Cambridge School of Clinical Medicine, Addenbrooke’s Hospital, Cambridge, United Kingdom, Cambridge, UK; ^3^Bristol Heart Institute, Bristol, UK; ^4^Royal Papworth Hospital, Cambridge, UK

**Correspondence**: Jacie Jiaqi Law

*Journal of Cardiothoracic Surgery 19(1):* A249

**Objectives:** Subxiphoid uniportal video-assisted thoracoscopic (SUVAT) lobectomy is a novel approach aimed to enhance recovery by reducing incisional pain after traditional intercostal uniportal video-assisted thoracoscopic (IUVAT) approach. However, evidence on SVAT remains scattered, especially in Europe and Americas. We aim to share our institutional experience with SUVAT lobectomy in the management of primary lung cancer and compare perioperative outcomes with IUVAT lobectomy to assess its safety and feasibility.

**Methods:** Retrospective analysis of prospectively collected data identified 57 patients and 132 patients undergoing SUVAT and IUVAT lobectomy respectively. Data on patient characteristics and perioperative outcomes are presented as mean ± standard deviation. Comparison of continuous and categorical variables between groups was performed with Pearson’s Chi-suqared test, Fisher’s exact test and Wilcoxon rank sum test. Statistical significance was considered at P < 0.05.

**Results:** Patient characteristics such as age (69.84 ± 9.69, p = 0.39), BMI (26.34 ± 4.42, p = 0.07), pulmonary function (FEV1/FVC 62.65 ± 15.24%, p = 0.86 and TLCO 75.19 ± 22.13%, p = 0.68) and ASA grade were similar between two groups. There was no difference in operating times (209 ± 69 min vs 199 ± 54 min, p = 0.42) but a higher rate of conversion to thoracotomy was observed in the IUVAT group (1.8% vs 11%, p = 0.04). Post-operatively, the SUVAT group experienced lower postoperative day 1 drainage volume (96 ± 118 ml vs 153 ± 154 ml, p < 0.001), shorter duration of chest drain use (3 ± 4.6 days vs 5 ± 4.6, p = 0.043) and reduced hospital length of stay (5 ± 6.4 vs 6 ± 14.3 days, p = 0.04). There was also no difference in R0 resection (p = 0.30), reoperation within 30 days (p = 0.56) and disease recurrence (p = 0.91).

**Conclusions:** Our experience demonstrates that SUVAT lobectomy for primary lung cancer is safe and promising with similar perioperative outcomes and oncological resection results when compared to IUVAT lobectomy.
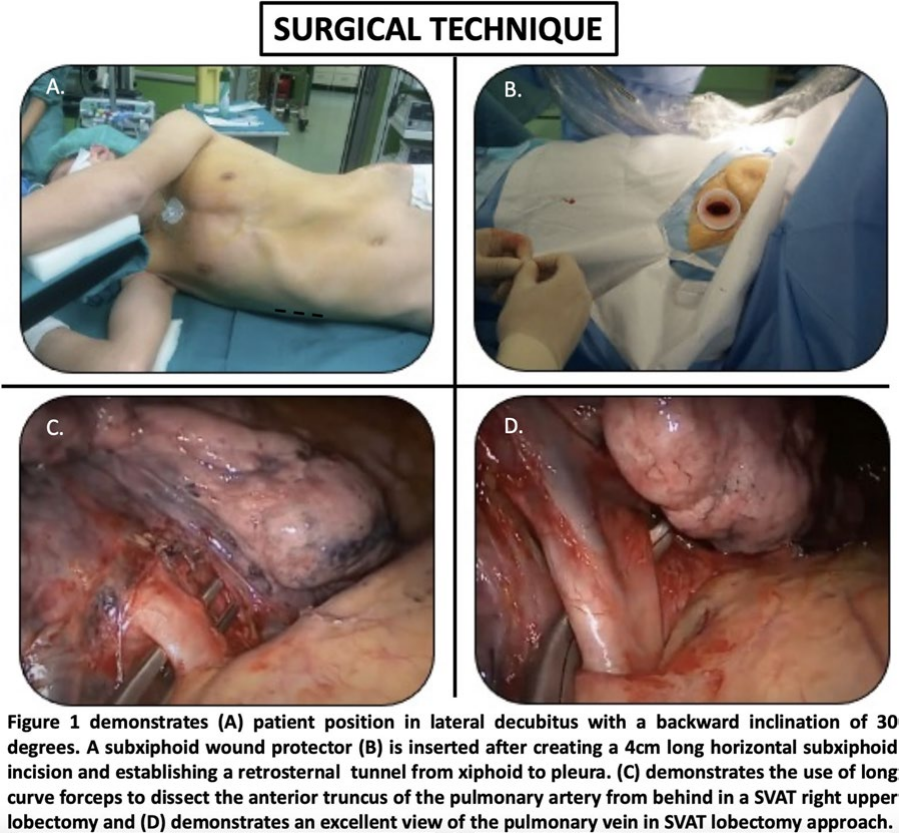


## A250 Chest wall resection and reconstruction for Ewing's Sarcoma: a single-centre experience

### Akshay Patel^1^; Alina-Maria Budacan^1^; Edward Ackling^2^; Haitham Khalil^2^; Sumathi Vaiyapuri^3^; Michael Parry^4^; Maninder Kalkat^1^

#### ^1^Department of Thoracic Surgery, University Hospitals Birmingham, Birmingham, UK; ^2^Department of Oncoplastic and Reconstructive Surgery, University Hospitals Birmingham, Birmingham, UK; ^3^Department of Histopathology, Royal Orthopaedic Hospital, Birmingham, UK; ^4^Department of Orthopaedic Surgery, Royal Orthopaedic Hospital, Birmingham, UK

**Correspondence**: Alina-Maria Budacan

*Journal of Cardiothoracic Surgery 19(1):* A250

**Objectives:** Ewing’s Sarcoma (ES) is a rare, soft tissue tumour with an incidence of 1 per million per year, and of these 20% will present as a chest wall primary. Extended follow-up studies have shown 60% survival at 5 years with the majority of ongoing mortality-risk attributable to disease recurrence and sequelae of treatment. We sought to investigate the factors related to overall and disease-specific survival following surgical resection in this group of patients.

**Methods:** We retrospectively interrogated our chest wall reconstruction data, from 2012 to 2022, for cases of ES. We included demographics, presenting symptoms, neoadjuvant therapy, operative variables (laterality, site, volume resected, tissue resected, number of ribs), type of reconstruction (prosthesis and/or flap), vital status, length of hospital stay and date of death/recurrence if applicable. Data was analysed using R Studio (R v4.0.3 system).

**Results:** Fourteen patients with ES were identified, 10/14 were male (71.4%). Median age at time of resection was 24.5 years (range 17–50 years), mean BMI was 22.4 ± 2.2, median ECOG status was 0, median ASA grade was 1. All patients had neo-adjuvant treatment either VIDE or VDC/IE chemotherapy (8/14 cases), VIDE chemotherapy + RT (3/14 cases) or Proton beam therapy + chemotherapy (3/14 cases). In 7/14 cases additional skeletal or visceral organs were removed either alone or in combination: diaphragm (3/14), lung (4/14) or vertebrae (2/14). Thirteen patients had clear margins following resection and 9/14 patients had > 75% necrosis of tumour tissue post neo-adjuvant treatment. 9/14 patients were alive at the time of writing with a mean follow-up of 1540 days (Fig. 1) and two had recurrence.

**Fig. 1:**
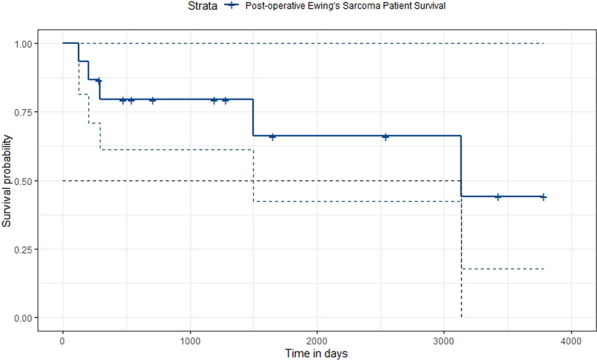
Overall survival in the cohort

**Conclusions:** Multimodal treatment is an effective approach to managing ES of the chest wall. A close collaboration between oncologists, thoracic, plastic and spinal surgeons is essential to achieve satisfactory long-term outcomes.

## A251 Survival after pulmonary metastasectomy for colo-rectal cancer

### Umair Aslam; James Jones; Sam Poon; Yasir Ahmed; Sobaran Sharma; Bilal Habshi; Ira Goldsmith

#### Morriston Hospital, Morriston, Swansea, UK

**Correspondence**: Umair Aslam

*Journal of Cardiothoracic Surgery 19(1):* A251

**Introduction:** The median five-year survival after pulmonary metastasectomy for colorectal carcinoma (CRC) ranges between 40–50%. The aim of our study was to determine whether adjuvant chemotherapy improves 3 and 5-year survival of patients after pulmonary metastasectomy.

**Methods:** We analysed our prospectively collected data of 83 patients [mean age 69.7 (95% CI 67.7–71.8) years and 72% male] between January 2016 and March 2022, who underwent pulmonary metastasectomy for CTC. 20% (16) had bilateral disease and 13.2% (12) a second new disease. Predictors of overall survival (OS) and disease-free survival (DFS) were determined.

**Results:** There were no hospital deaths or deaths at 30 and 90 days. Univariate analysis indicated that diameter of largest metastasis ≤ 2 cm were associated with higher OS (*p* = 0.02) and DFS (*p* = 0.02). 3 and 5-year OS was 79% (CI 75–83) and 45% (CI 42–48) respectively, and DFS 83% (CI 80–86) and 50% (CI 46–54). 86% of patients received adjuvant chemotherapy. There were no differences in OS at 3-years, 83% (CI 80–86) vs 80% (CI 82–84), *p* = 0.17, or 5-years 48% (CI 44–52) vs 40% (CI 42–46), *p* = 0.19 who received vs did not receive chemotherapy nor in the DFS at 3-years 80% (CI 77–83) vs 76% (72–80), *p* = 0.35, or 5-years 55% (CI:51–59) vs 49% (CI:47–52), *p* = 0.29.

**Conclusions**: Our study suggests that there was no difference in the OS or DFS at three and five years with or without adjuvant chemotherapy following pulmonary metastasectomy for CRC.

## A252 A machine learning approach to predicting prolonged length of stay after primary lung cancer resection in an enhanced recovery after surgery programme

### Lauren Kari Dixon^1^; Vito Domenico Bruno^1^; David Messenger^2^; Luke Rogers^2^; Neil Rasburn^2^; Natasha Joshi^2^; Timothy Batchelor^2^

#### ^1^Bristol Heart Institute, Bristol, UK; ^2^University Hospitals Bristol and Weston, Bristol, UK

**Correspondence**: Lauren Kari Dixon

*Journal of Cardiothoracic Surgery 19(1):* A252

**Objective:** Enhanced Recovery After Surgery (ERAS) protocols in Thoracic Surgery are designed to improve post-operative recovery including reducing length of hospital stay (LOS). Prolonged LOS is associated with increased morbidity. We aimed to design predictive models of prolonged LOS after primary lung cancer resection using machine learning (ML) methodology.

**Methods:** Prospective data was collected for 1537 consecutive patients who underwent lung cancer resection and followed the ERAS programme in our Thoracic Surgery Centre. The median LOS was five days; 725 (47%) had a LOS < 5 days, while the remaining 812 (53%) of patients had a prolonged LOS (≥ 5 days). A training/testing ratio of 70/30 and resampling methods were used, and cross-validation was conducted.

**Results:** Prolonged LOS patients were older (p < 0.001), more frequently female (p = 0.002), had an open, rather than VATS, approach (p < 0.001), more frequently had pneumonectomy (p < 0.01), had higher ASA (p < 0.001) and lower pre-operative haemoglobin (< 0.001). The predictive abilities of the ML models were as follows: logistic regression: Area under the Curve (AUC) = 0.76, accuracy = 0.72; Generalized additive model: AUC = 0.73, Accuracy = 0.69; Naïve Bayes AUC = 0.68, Accuracy 0.65.

**Conclusions:** Developing a reliable predictive model for LOS following primary lung cancer resection proved to be difficult, but several preoperative characteristics have a significant impact on the probability of prolonged LOS. Larger studies are needed to investigate the possibility of developing a reliable predictive model that would help to improve identification of patients at high risk of prolonged LOS and guide service planning.

## A253 Surgery for small cell lung cancer: single centre experience

### Abed Elfattah Atieh; Hesham Ahmad; Abdelrahman Elsayed; Mohammad Hawari

#### Thoracic Surgery Department, Nottingham University Hospitals NHS Trust, Nottingham, UK

**Correspondence**: Abed Elfattah Atieh

*Journal of Cardiothoracic Surgery 19(1):* A253

**Introduction:** Surgery has very little role in management of small cell lung cancer. NICE has recommended that surgery can be considered in people with early-stage small cell lung cancer T1-2a, N0, M0. We aim to review our practice and assess the outcome of surgery in this group of patients.

**Methods:** A retrospective review of prospectively collected data between February 2010 and August 2022, which included all patients who underwent lung cancer resection with a final histopathological diagnosis that included SCLC was done. Data was analysed using SPSS to assess outcomes and factors associated with better survival.

**Results:** 51 patients were identified, 31 males and 20 females with mean age of 72 years. All were current/ex-smokers. Mean FEV1 was 73.1% and DLCO 61.6%. 32 patients had stage I disease with T1-T2a, N0, M0 and 19 had T2b-T4, N0-N2, M0. 21 patients had small cell cancer only on their histopathology. 30 had small cell cancer associated with another non-small cell type. One-year survival for the whole group was 87.5%, two-year survival was 66.7% and 5-year survival was 36.5%. One-year survival for stage I versus other stages was 96.6% and 72.6%, 2-year survival was 81.6% and 42.3% and 5-year survival was 42.8% and 23.5%, respectively (*p* = 0.02). 37 patients had lobectomies, 14 had sub-lobar resections including segmentectomies and wedge resections. There was no difference in survival between males and females, or type of resection or nodal status. There was a significantly better 1-year-survival (89.6% versus 84% respectively) and 5-year-survival (47.5% versus 18% respectively) in patients with mixed small and non-small cell cancer compared to pure small cell cancer (p = 0.016). All patients had adjuvant chemotherapy and were referred for consideration of prophylactic brain radiotherapy.

**Conclusions:** Patients with Stage I Small Cell Lung Cancer (T1-T2a, N0M0) should be considered for lung cancer resection as they would benefit from longer-term survival.

## A254 Role of surgery in stage IV lung cancer with oligo-metastatic brain disease thoracic surgery department, nottingham university hospitals NHS Trust, UK

### Abed Elfattah Atieh; Hesham Ahmad; Abdelrahman Elsayed; Mohammad Hawari

#### Thoracic Surgery Department, Nottingham University Hospitals NHS Trust, Nottingham, UK

**Correspondence**: Abed Elfattah Atieh

*Journal of Cardiothoracic Surgery 19(1):* A254

**Background:** Outcomes for stage IV lung cancer is very poor with reported median survival of 2–11 months. In the UK, 1-year survival for stage IV lung cancer is 19.3% and 5-year survival is 2.9%. Patients with oligo-metastatic brain disease who are suitable for curative intention treatment of the brain metastasis are discussed in the lung multi-disciplinary team meeting and are considered for radical treatment of their lung cancer, as these patients might survive longer as per different studies. Our aim is to review our practice and outcomes in this group of patients.

**Methods:** Data for patients who underwent curative lung resections in our centre following brain surgery or Stereotactic Radio-Surgery (SRS) to their brain metastases between January 2011 and March 2022 was reviewed retrospectively from a prospectively collected database and outcomes were analysed using SPSS.

**Results:** 16 patients met the inclusion criteria. There were nine males and seven females with a mean age of 65 years. All patients were smokers/ex-smokers. Nine patients had Adenocarcinoma and seven had squamous cell carcinoma. For their brain metastases, five had craniotomy and resection and seven had SRS treatment and four had both modalities. For their lung cancer, 14 patients had Video Assisted Thoracic Surgical (VATS) lung resections and two had thoracotomies including one sleeve lobectomy. The mean hospital stay was six days. All patients were discharged home. Overall, 1- and 2-year survival were 92.3%, 5-year survival was 55.4%. There was no difference in survival when comparing sex, T stage or N stage.

**Conclusions:** Patients with operable lung cancer in the chest who have Oligo-metastatic brain disease should be discussed in a brain MDT for consideration of curative treatment to their metastases. These patients can undergo lung resection following that. From our study, their survival was significantly higher when compared to other stage IV lung cancer patients.

## A255 Long term outcomes of pneumonectomy for lung cancer in a tertiary referral centre: a 20-year experience

### Ramanish Ravishankar^1^; Azar Hussain^2^; Syed Qadri^2^

#### ^1^Hull University Teaching Hospitals, Castle Hill Hospital, Hull, UK; ^2^Castle Hill Hospital, Hull, UK

**Correspondence**: Ramanish Ravishankar

*Journal of Cardiothoracic Surgery 19(1):* A255

**Introduction:** The incidence of pneumonectomy for lung cancer in the UK is continuing to decline in the era of minimally-invasive thoracic surgery totalling approximately 3.5% of lung cancer resections annually. Literature is lacking for long-term survival of pneumonectomies. This study serves as an update to our previous results. Between 1998 and 2008, 233 patients underwent pneumonectomy compared to between 2009 and 2018 with 104 patients.

**Methods:** From January 1998 until December 2018, 337 patients underwent pneumonectomy. Data was retrospectively analysed for age, gender, laterality, histology and time period. The primary endpoint was mortality.

**Results:** Operative mortality was 3.2% overall which was lower than the national average of 5.8% and Thoracoscore of 8%. In the last five years, there were no in-hospital, operative or 30-day mortality. During this period, 90-day mortality was 10%. Left-sided pneumonectomies had significantly better overall survival (2.68 vs. 2.03 years; p = 0.0038), squamous cell carcinoma (3.23 vs. 1.55 years; p = 0.0001) as well as those aged less than 70 (2.48 vs. 1.92 years; p = 0.049). There was no significant difference in survival between gender (p = 0.37). Intervention from 1998 to 2008 had significantly greater survival compared to the latter ten years (2.53 vs 2.21 years; p = 0.032). The Cox model (Fig. 1) shows that laterality, age, histology and time period remain significant with multivariate testing.

**Fig. 1:**
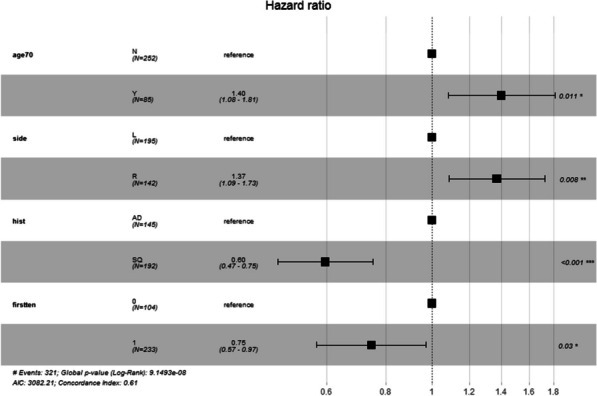
Cox model showing significant factors contributing to survival

**Discussion:** Our updated retrospective study has built on our previous results by reinforcing the operative success of pneumonectomies. The incidence of pneumonectomies is likely to decrease with the potential deployment of nation-wide lung cancer screening in the UK due to earlier detection. However, with an overall improvement in survival and managing risk factors, we have shown that the pneumonectomy still is a safe and effective strategy when required.

## A256 Incidence of EGFR mutations amongst resected non-small cell lung cancer patients: a review from a single surgical centre

### Abed Elfattah Atieh; Abdelrahman Elsayed; Hesham Ahmad; Mohammad Hawari

#### Thoracic Surgery Department, Nottingham University Hospitals NHS Trust, Nottingham, UK

**Correspondence**: Abed Elfattah Atieh

*Journal of Cardiothoracic Surgery 19(1):* A256

**Background:** In January 2022, NICE has allowed Osimertinib (Tagrisso) as a possible adjuvant treatment after complete tumour resection in adults with stage IB to IIIA Non-Small-Cell Lung Cancer whose tumours have Epidermal Growth Factor Receptor (EGFR) exon 19 deletions or exon 21 (L858R) substitution mutations. We aim to review our database and identify all biomarkers prevalence in patients who had surgery with emphasis on EGFR mutations, who would be potential candidates for this novel treatment.

**Methods:** A retrospective study was done using our prospectively collected database and included all patients who underwent lung cancer resections between May 2021 and April 2022. Molecular data was collected and analysed according to type of cancer, gender and smoking status.

**Results:** 317 patients underwent lung cancer resection during this period, 56.5% were females, aged between 30 and 90. The histological subtypes were 178 (56.2%) adenocarcinoma, 89 (28.1%) squamous cell carcinoma, three Adeno-squamous carcinoma, 5 large cell neuroendocrine cancer, 27 carcinoid tumour and 15 others. 85.2% were smokers (85.2%). 21 patients (12%) of those tested for EGFR mutations had it, including 15 in exon 21, three in exon 19, 2 in exon 20 and one G719Xaa and S768I mutations. Nine patients were in stages IB-IIIA, eight of which met NICE recommendations for adjuvant treatment.

7/200 patients (3.5%) had ALK mutations, 129/273 patients (47.3%) had > 1% PDL-1 expression, out of which 45 (16.5%) had > 50% PDL-1 expression. 4/201 (2%) had BRAF mutation, 8/198 (4%) had ROS-1 mutation. EGFR mutations were only detected in Adenocarcinoma patients, 47.6% were females and 7 were non-smokers (33.3%). The most commonly detected EGFR mutation was exon 21 (L858R) substitution (71.4%).

**Conclusions:** The prevalence of EGFR mutation was 12%, these were all adenocarcinoma. Non-Smokers with EGFR mutations are more likely to develop lung cancer. 4% of all EGFR tested patients will be candidates for adjuvant treatment with Osimertinib.

**Transplant and Failure**

## A257 In-situ recovery of the DCD donor heart shows equivalent survival to conventional donor hearts

### John Louca^1^; Marco Öchsner^2^; Ashish Shah^3^; Francisco González-Vilchez^4^; Mario Royo-Villanova^5^; Deane Smith^6^; Filip Rega^7^; Vincent Tchana-Sato^8^; Marian Urban^9^; Stephen Large^10^

#### ^1^Cambridge University, Cambridge, UK; ^2^Cambridge University; ^3^Vanderbilt Heart Transplant Unit; ^4^Spanish Registry on Heart Transplantation; ^5^Hospital Universitario Virgen de la Arrixaca; ^6^NYU, Langone Health; ^7^Departmenst of Cardiac Surgery and Cardiology, The University Hospital Leuven; ^8^Department of Cardiovascular Surgery, CHU Liege; ^9^Department of Cardiothoracic Surgery, University of Nebraska Medical Centre Omaha; ^10^Royal Papworth Hospital, Cambridge, UK

**Correspondence**: John Louca

*Journal of Cardiothoracic Surgery 19(1):* A257

**Objectives:** Heart transplantation is an effective treatment offering the best recovery in both quality and quantity of life in those affected by refractory, severe heart failure. However, transplantation is limited by donor organ availability. The reintroduction of heart donation after the circulatory determination of death (*DCD*) in 2014 offered an uplift in transplant activity by 30%. Thoraco-abdominal normothermic regional perfusion (taNRP) enables in-situ reperfusion of the DCD heart. The objective of this paper is to assess the clinical outcomes of DCD donor hearts recovered and transplanted from donors undergoing taNRP.

**Methods:** Outcomes included functional warm ischaemic time, use of mechanical support immediately following transplantation, perioperative and long-term actuarial survival and incidence of acute rejection requiring treatment. 157 taNRP DCD heart transplants have been included from 15 major transplant centres worldwide including the UK, Spain, the USA and Belgium. 673 Donation after the neurological determination of death (DBD) heart transplantations from the same centres were used as a comparison group for survival.

**Results:** taNRP resulted in a 23% increase in transplantation activity. Survival was similar in the taNRP group when compared to DBD. 30-day survival was 96·8% (n = 157), 1-year survival was 93·2% (n = 82) and 5-year survival was 84·3% (n = 19).

**Conclusions:** taNRP provides a significant boost to heart transplantation activity. The survival rates of taNRP are not inferior to DBD. The similar survival may in part be related to a short warm ischaemic time or through a possible selection bias of younger donors, this being an uncontrolled observational study. Therefore, taNRP offers an effective method of organ preservation and procurement. This early success of.

## A258 A single centre prospective study comparing the Sherpa-Pak cardiac transport system with conventional icebox preservation for heart transplantation

### Bhuvaneswari Krishnamoorthy^1^; William Critchley^2^; Joanne Hasan^3^; Shishir Kore^3^; Nnamdi Nwaejike^3^; Vipin Mehta^3^; Steven Shaw^3^; Paul Callan^3^; James Barnard^3^; Rajamiyer Venkateswaran^3^

#### ^1^Manchester Foundation Trust and The University of Salford and The University of Manchester, Manchester, UK; ^2^University of Leeds, Leeds, UK; ^3^Manchester Foundation Trust, Manchester, UK

**Correspondence**: Bhuvaneswari Krishnamoorthy

*Journal of Cardiothoracic Surgery 19(1):* A258

**Introduction: I**ce storage can cause tissue injury whereas higher storage temperatures may cause ischemic injury. Paragonix Sherpa-Pak system addresses these issues by providing consistent cooling through its proprietary cool safe technology which maintains donor heart temperatures between 4 and 8 °C.

**Methods:** Between April 2018 and March 2022 included 74 patients (20 patients transported with Sherpa-Pak system and 54 using the conventional icebox storage). The aim of this study was to compare patient outcomes including Postoperative complications, length of Cardiothoracic Critical Care Unit (CTCCU) stay, total hospital stay, incidence of severe Primary Graft Dysfunction (PGD) requiring Extra-corporal Membrane Oxygenation (ECMO and rate of mortality.

**Results:** No statistically significant differences in demographic data or risk profile. The most important finding from this study was the reduction in the requirement for the use of ECMO following transplant utilising the Sherpa-Pak system (35.2% vs. 10.0%, p = 0.04). There were also significant reductions in respiratory complications using the Sherpa-Pak system (61.1% vs 10.0%, p = 0.0001) and infectious complications (57.4% vs. 10.0%, p = 0.0002).There was no statistically significant difference in CTCCU length of stay between the Sherpa-Pak and Icebox groups(14.1 ± 12.6 vs 19.3 ± 16.0, p = 0.19); or total length of hospital stay (30.6 ± 16.6 vs 35.6 ± 20.4, p = 0.32). The incidence of Intra-aortic balloon pump post-surgery was slightly high in the icebox group (15% vs 27.8%, p = 0.36). Thirty-day mortality (0% vs 5.6%, p = 0.56) and survival analysed to July 2022 did not differ between the groups (p = 0.26) (Fig. 1). Total mean cost of transplantation per patient was lower in the Sherpa-Pak group (£39,532.60) compared to the Icebox group (£53,489.91), however, this difference was not statistically significant in this study (p = 0.19).

**Conclusions:** Sherpa-Pak cardiac transport system reduces the requirement for ECMO support following heart transplant.

## A259 Impact of 3-h donor heart ischaemic time on outcomes after heart transplant: a 7-year single-centre retrospective study

### Fadi Al-Zubaidi; Ahmed Mohamed Abdel Shafi; Daniel Sitaranjan; Pradeep Kaul

#### Royal Papworth Hospital NHS Foundation Trust, Cambridge, UK

**Correspondence**: Fadi Al-Zubaidi

*Journal of Cardiothoracic Surgery 19(1):* A259

**Objectives:** Compare outcomes in heart transplant recipients with total ischaemic time of 180 min to those with ischaemic times greater than 180 min.

**Methods:** We collected data on 218 patients undergoing heart transplants between April 2014 and February 2021 at our institution. Only recipients of hearts from brainstem dead donors were included. Patients were grouped by ischaemic times into > 3 h (n = 79) and < 3 h (n = 139). We compared donor characteristics, intraoperative variables and postoperative outcomes. Our primary outcome was all-cause mortality (ACM). Secondary outcomes were primary allograft dysfunction, length of ITU stay and need for post-operative mechanical circulatory support (MCS). We planned multivariable regression models to assess the association between ischaemic time and postoperative outcomes. Variables differing with a p-value of < 0.25 were included as covariates. We then split the > 3 h group further into half-hour periods and conducted log rank survival analyses to assess post-transplant mortality. Kaplan–Meier curves were generated.

**Results**: Univariable comparisons demonstrated greater use of MCS and associated complications in recipients with total ischaemic time of > 3 h. Multivariable analyses demonstrated that ischaemic time > 3 h was not an independent predictor of ACM (HR:0.87; 95% CI:0.47–1.61), primary allograft dysfunction (OR:0.62; 95% CI:0.30–1.27) or prolonged ITU stay (OR:1.74; 95% CI: 0.88–3.42). It was a predictor of need for MCS post-transplant (OR:3.47; 95% CI:1.38–8.72). Log rank survival statistic was not significant (p = 0.814); Kaplan–Meier curves demonstrated comparable long-term survival (Fig. 1).

**Fig. 1:**
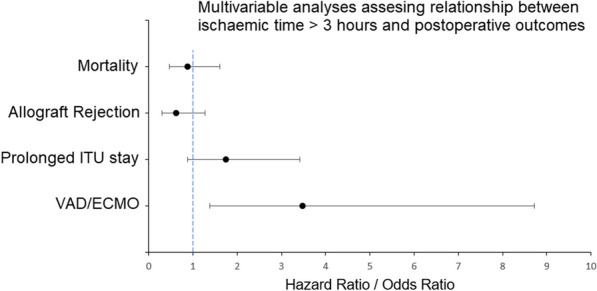
Forest plot demonstrating the multivariable association of prolonged ischaemic time with mortality, prolonged ITU stay, takeback to theatre and post-operative VAD/ECMO

**Conclusions:** Total cardiac allograft ischaemic times of > 3 h do not predict increased mortality, or prolonged ITU admission. Long-term follow up demonstrates comparable survival. Predicted ischaemic times > 3 h alone shall not be considered a barrier to heart transplantation.

## A260 Left ventricular assist devices for destination therapy in advanced heart failure patients: a systematic review of clinical effectiveness

### Sophie E Beese^1^; Tuba Saygın Avşar^2^; Malcolm Price^1^; Louise J Jackson^1^; Chidubem Okeke Ogwulu^1^; Pelham Barton^1^; Hoong Sern Lim^3^; David Quinn^3^; David J Moore^1^

#### ^1^University of Birmingham, Birmingham, UK; ^2^University College London, London, UK; ^3^University Hospital Birmingham NHS Foundation Trust, Birmingham, UK

**Correspondence**: Sophie E Beese

*Journal of Cardiothoracic Surgery 19(1):* A260

**Objectives:** Left ventricular assist devices (LVADs) can be used as ‘destination therapy’ (DT) for advanced heart failure patients who are ineligible for transplant. However, this is not commissioned by the UK NHS. This systematic review aimed to summarise the available evidence on the effectiveness of LVADs compared to medical management (MM) or other LVADs in these advanced heart failure patients.

**Methods:** Studies with > 50 adults implanted with any LVAD for DT were included. Cochrane CENTRAL, MEDLINE, Embase and WHO Clinical trials were searched for primary studies until January 2022 and relevant registry reports were sought. Data extraction and risk of bias assessment were done by one reviewer and checked by a second. The analysis focused on HeartMate 3 (HM3) as the only available device in the UK. A narrative synthesis was undertaken and a network meta-analysis (NMA) was considered to compare HM3 with MM.

**Results:** 134 studies (five RCTs, one non-randomised controlled trial, reports from five registries, 86 observational studies, five ongoing RCTs, 32 systematic reviews) were included. Only one trial compared HM3 to another device (HeartMate II–MOMENTUM 3 trial). Patient survival has improved with the evolution of LVADs such that 24-month survival with HM3 is 76.7% and occurrence of major events has reduced. Use of an NMA to obtain a relative effect of HM3 to MM was limited due to heterogeneity between studies, however the relative risk of death at 24-months was 0.25 (95% CI 0.13–0.47).

**Conclusions:** Whilst HM3 is considered clinically effective in DT patients, there is no published evidence directly comparing it to the only alternative therapy MM, and methodically sound indirect comparison is largely not possible. Ongoing studies (e.g. SweVAD randomised trial of HM3 vs MM) may address this evidence gap and inform more precisely analysis of the cost-effectiveness of LVAD as DT and thereby commissioning decisions.

Funding: UK NIHR-HTA programme: NIHR128996

## A261 Cost-effectiveness of left ventricular assist devices as destination therapy: an economic modelling study

### Tuba Saygin Avsar^1^; Louise Jackson^2^; Pelham Barton^2^; Sophie Beese^2^; Lim Sern^2^; David Quinn^2^; Malcolm Price^2^; David Moore^2^

#### ^1^University College London, London, UK; ^2^University of Birmingham, Birmingham, UK

**Correspondence**: Louise Jackson

*Journal of Cardiothoracic Surgery 19(1):* A261

**Objectives:** In the UK, Left Ventricular Assist Devices (LVADs) are not commissioned by the NHS for advanced heart failure patients who are ineligible for heart transplantation (destination therapy). This study aimed to estimate the cost-effectiveness of LVADs as destination therapy in the UK compared to optimal medical therapy (OMM).

**Methods:** A cost-utility analysis from an NHS perspective was conducted, using a novel Markov model with a lifetime horizon and monthly cycles. The model development was informed by systematic reviews and guidance from clinicians, patients and commissioners. The costs were in UK £ at 2019 prices, and a discount rate of 3.5% was employed. The analysis was repeated incorporating the probability of transition to heart transplant (HT). Exploratory sub-group analyses estimated the impact of severity of heart failure on cost-effectiveness, using INTERMACS profiles. Uncertainty was measured in sensitivity analyses.

**Results:** LVAD produced an additional 3.67 (95% CI 3.19–4.19) QALYs at an incremental cost of £152,329 (95% CI £125,665–£181,812) compared to OMM. Thus, the incremental cost-effectiveness ratio per QALY (ICER) was £54,748. The probability of cost-effectiveness was 0% at a threshold of £30,000/QALY, reaching 100% at £80,000. The ICER remained above £30,000 when severity weighting was applied on QALYs as per NICE guidelines if a small proportion of LVAD patients became eligible for a heart transplant and in sub-group analyses based on INTERMACS profiles. Varying outpatient costs for medical management had a significant impact on results.

**Conclusions:** In contrast to two recent UK studies, this study found that LVADs are not cost-effective as destination therapy in the UK at a threshold of £30,000/QALY. Robust data on ongoing costs for OMM are needed.

Funding: UK NIHR-HTA programme: NIHR128996

## A262 Development of a Prognostic score to aid decision making in organ utilisation for UK lung transplantation

### Gillian Hardman^1^; Rachel Hogg^1^; Rushton Sally^1^; Karen Booth^2^; Andrew J Fisher^3^; John H Dark^3^

#### ^1^NHS Blood and Transplant, Bristol, UK; ^2^Freeman Hospital, Newcastle Upon Tyne, UK; ^3^Newcastle University, Newcastle Upon Tyne, UK

**Correspondence**: Gillian Hardman

*Journal of Cardiothoracic Surgery 19(1):* A262

**Objectives:** The aim was to develop an objective scoring system to aid selection of donor organ and recipient, to improve lung utilisation in UK transplantation.

**Methods:** First time, adult lung-only recipients between 1 January 2010 and 31 December 2019 were randomly divided into model development (70%) and model validation (30%) cohorts. Variables selected a priori were introduced in a stepwise elimination into multivariable logistic regression models for the prediction of grade 3 PGD within the first 72 h post-transplant (PGD3) and death at 1-year. Risk groups were defined (lower-, moderate- and higher-risk). The incidence of PGD3 was compared using chi square tests and unadjusted 90-day, 1-year and 5-year survival rates were compared using Kaplan–Meier survival analysis and log-rank tests.

**Results:** Multivariable logistic regression analysis was performed on a complete case cohort of 1490 transplants. Six factors were identified for the prediction of PGD3 (donor past smoking, donor type, donor age, recipient disease, recipient ethnicity and recipient BMI), with five factors identified for the prediction of death at 1-year (recipient ethnicity, registration BMI, registration creatinine, registration prednisolone dose and recipient diabetes). Donor PaO_2_/FiO_2_ ratio was included based on clinical relevance. The additive UKLRI is shown in Fig. 1.

**Fig. 1:**
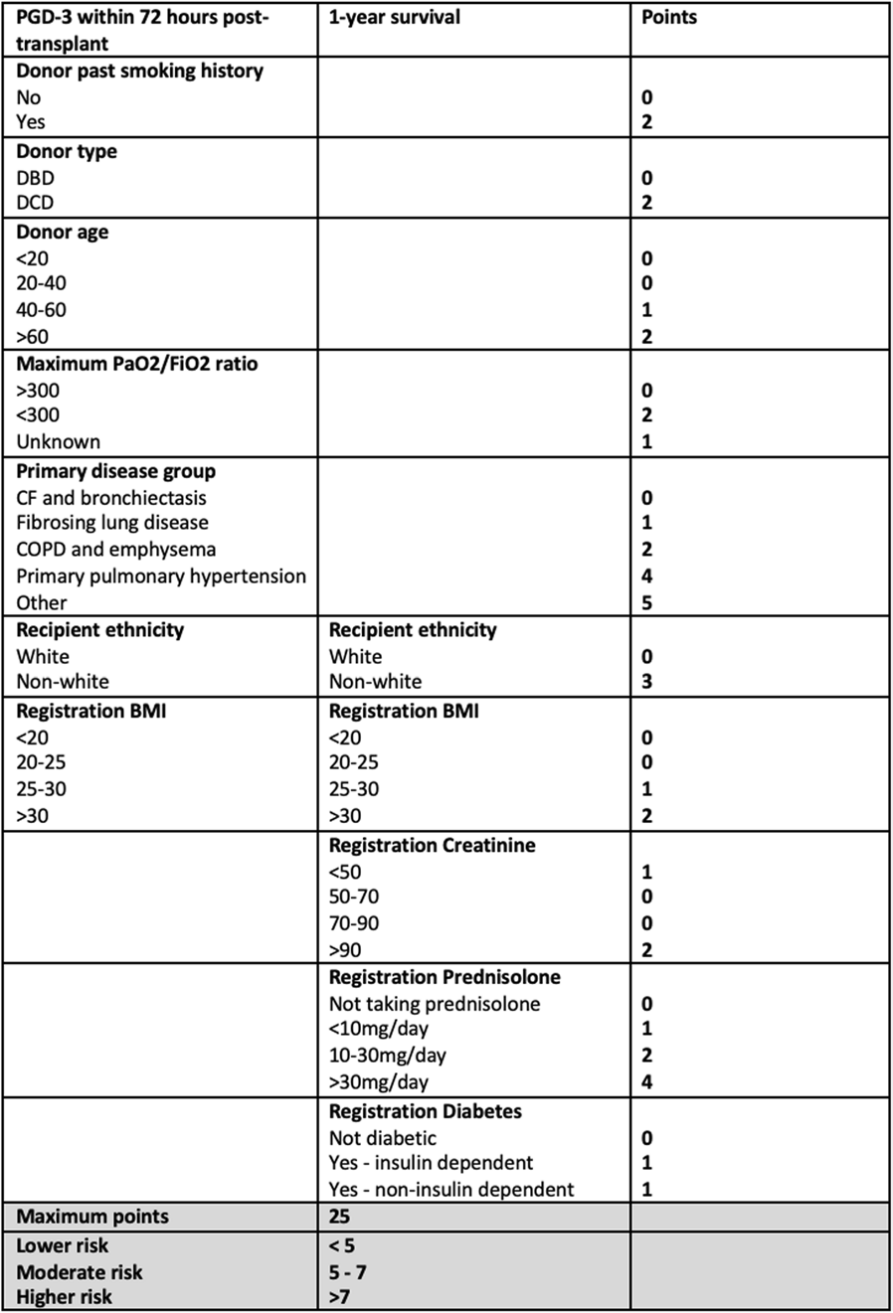
Factors included in the additive UK Lung risk index (UKLRI) score, maximum points awarded and risk stratification

There was a significant difference in incidence of PGD3 (lower risk 16%, moderate risk 22%, higher risk 40% p value < 0.0001) and survival, at all time points, for different UKLRI risk groups.

**Conclusions: **The UKLRI is a simple additive tool stratifying combinations of donor and recipient to three risk groups. *Higher risk* is not prohibitive to utilisation but may facilitate improved allocation and guide perioperative management. Routine adoption has potential to significantly increase lung transplant activity.

